# Effective synthesis of bicyclodienes via palladium-catalyzed asymmetric allylic alkylation and ruthenium-catalyzed cycloisomerization

**DOI:** 10.3906/kim-2004-81

**Published:** 2020-12-16

**Authors:** Nizam HAVARE

**Affiliations:** 1 Department of Chemistry, School of Humanities & Sciences, Stanford University, Stanford, California 94305-5580 United States

**Keywords:** Synthetic method development, asymmetric catalysis, *Tsuji-Trost* -reaction, palladium-catalyzed asymmetric allylic alkylation, ruthenium-catalyzed cycloisomerization

## Abstract

[n.3.0]Bicycles (n = 3–6) can be synthesized using palladium-catalyzed asymmetric allylic alkylation followed by ruthenium-catalyzed cycloisomerization. New types of triarylphosphino-1,2-diaminooxazoline ligands show the same high levels of enantioselectivity observed with Trost ligand when employed in Pd-catalyzed allylic alkylation reactions. The enyne products of these allylic alkylation reactions were further elaborated using a Ru-catalyzed redox isomerization process, for which a mechanism is proposed.

## 1. Introduction

The development of novel and effective cyclization methods constitutes a continuing challenge in organic synthesis, as five- and six-membered cycles are essential structural units in pharmaceuticals, agrochemicals, and other biologically active molecules [1]. Transition-metal carbenes play a fundamental role as reactive intermediates in the synthesis of these pharmaceuticals and/or agrochemicals [2,3]. Since the initial report by Trost et. al., metal-catalyzed enyne cycloisomerization [4] has become an important tool for synthesizing complex molecules [5–8] from simple building blocks while maintaining good selectivity [9] and perfect atom economy [10]. Several inter- and intramolecular reaction types have been developed including: alkyne addition to allylic alcohols [11–14], intramolecular addition of alkyne to alcohol [15,16], Alder-ene reaction [17–19] and its application to natural product synthesis [20–29], and allene-alkene addition [30,31] using common Ru-complexes: [CpRu(CH3CN)3] PF6, [CpRu(PPh3)2Cl], [IndRu(PPh3)2Cl], [CpRu(cod)Cl], and chiral [C19H23N2O2RuS] [32–35]. The utility of ruthenium catalysis has been studied in intramolecular cycloisomerizations, providing an efficient, atom-economical method to access an array of 1,3- or 1,4-dienes [31] and other types of bicyclic compounds [36,37]. The synthesis of *cis* and/or *trans* 2-alkylbicyclo[n.3.0]-diene compounds starting from the derivatives of allene-2-yl, cycloalk-2-enyl dimethyl malonate has also been reported in good to excellent yield using palladium [38–45].

Palladium-catalyzed asymmetric allylic alkylation (AAA), also known as the Tsuji-Trost reaction, is one of the most efficient methods for forming allylic C-C, C-O, C-N, C-S, and C-P bonds. It tolerates a broad range of olefins that contain a leaving group in the allylic position, employs mild reaction conditions, and forms the desired products in very high enantioselectivities and yields [6,46,47]. The Trost-Group has had a long-standing interest in this area, as well as in the area of TM-catalyzed AAA, and the aim was to combine these two general methods into a unified approach to synthesize bicyclodienes.

**Figure 1:**
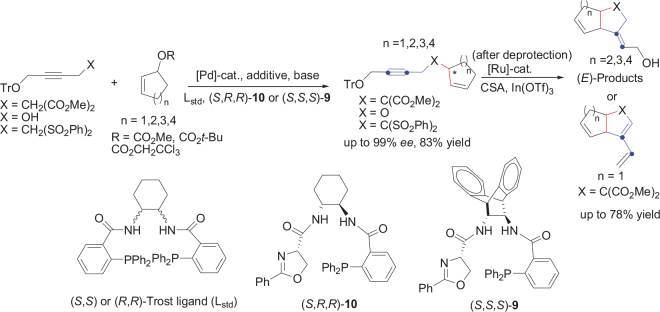
New synthetic method using Pd-catalyzed AAA and Ru-catalyzed cycloisomerization.

A useful new application of these two general methods is reported here for the synthesis of 2-alkyl-bicyclo[n.3.0]-diene compounds. Here n = 3–6. Starting from malonate protected by trityl group and cycloalk-2-enyl carbonyl derivatives, enyne compounds can be synthesized with high enantioselectivities and yields. These compounds were then deprotected using BiCl3 and treated with [IndRu(PPh3)2Cl] 1 [48] for cyclized end products (Figure 1).

## 2. Results

Malonate 2a [49] was tested as a nucleophile with cyclopent-2-enyl benzoate 3a as an electrophile under different reaction conditions to obtain high enantioselectivity and yield of allylated malonate 4 (Table 1). Pd2(dba)3∙CHCl _3_ catalyzed the asymmetric allylic reaction in the presence of ( *S* , *S* )-Trost-ligand (L _std_ ) in THF in very good yield and very low enantioselectivity (entry 1, Table 1).

**Table 1:** Optimization of palladium-catalyzed asymmetric allylic alkylation (Pd-catalyzed AAA).

Entry	Cat.	-R	L	Base	Additive	Solvent	Temp.	Yield (%)	ee (%)
1	Pd _2_ (dba) _3_ CHCl _3_	-Boc	L _std_ (S,S)	NaH	-	THF	r.t.	70	16
2	[η ^3^ -C _3_ H _5_ PdCl] _2_	-Bz	L _std_ (R,R)	NaH	THAB	CH _2_ Cl _2_	r.t.	71	-49
3	[η ^3^ -C _3_ H _5_ PdCl] _2_	-Bz	L _std_ (R,R)	NaH/DBU	-	CH _2_ Cl _2_	-20 → r.t.	70	-55
4	Pd _2_ (dba) _3_ CHCl _3_	-Boc	L _std_ (S,S)	DBU	-	THF	r.t.	50	40
5	[η ^3^ -C _3_ H _5_ PdCl] _2_	-Bz	L _std_ (R,R)	NaH/DBU	-	CH _2_ Cl _2_	-78 → r.t.	82	-67
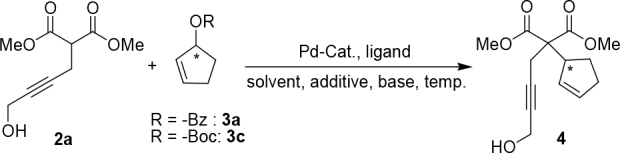									

When the reaction was carried out in dichloromethane (DCM) with tetrahexylammonium bromide (THAB) as an additive, the enantioselectivity increased to 49% *ee* (entry 2, Table 1). It is assumed that THAB plays a role as a phase transfer catalyst to increase the solubility of sodium malonate salt [46,47,49]. When the reaction was carried out in DCM in the presence of sodium hydride (60% in mineral oil) and 1,8-diazabicyclo[5.4.0]undec-7-en (DBU) starting at –20 °C and slowly warming to r.t. overnight, the enantioselectivity increased again to 55% *ee* (entry 3, Table 1). Using only DBU as a base in THF without any additive decreased the enantioselectivity of the reaction to 40% *ee* (entry 4, Table 1). Switching the solvent from DCM to THF and/or the higher temperature compared to entry 3 may be reasons for the lower enantioselectivity. The highest enantioselectivity was obtained in DCM by addition of the sodium hydride and DBU at –78 °C and allowing the reaction mixture to slowly warm to r.t. (entry 5, Table 1).

It is well known that the three dimensional direction and/or steric hindrance of the Trost-ligand in situ play distinct roles in determining the enantioselectivity of asymmetric allylic alkylation, but the angle (ϴ) between *π* -allylpalladium intermediate and the bonding post of the coordinated ligand is also significant [50]. Therefore, the design of Trost-ligand with different bonding will help to understand the asymmetric allylic alkylation for a broad range of substrate [50]. After low *ee* using standard Trost-ligand (L _std_ ) in Table 1 was obtained, it was decided to synthesize new ligands with a different bonding post.

**Figure 2:**
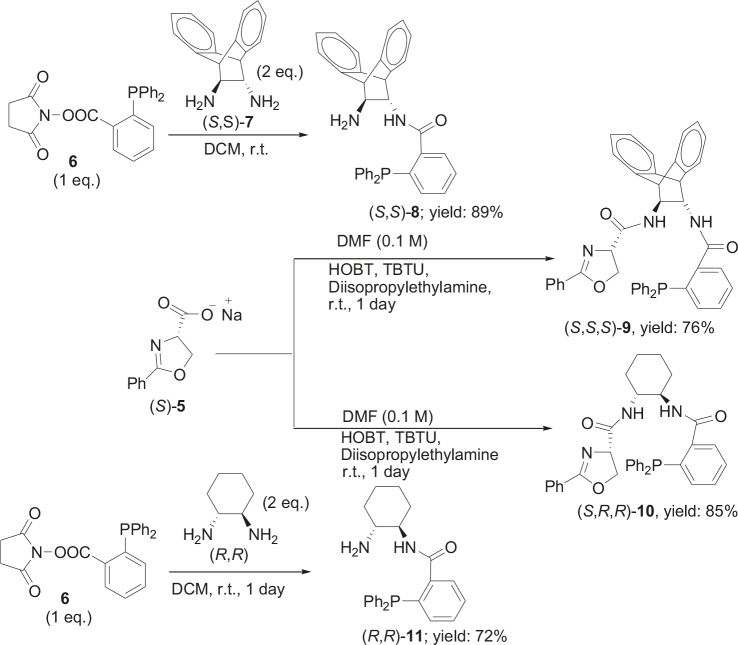
Synthesis of new ligands (S,S,S)-9 and (S,R,R)-10.

These novel ligands are synthesized according to the established synthetic strategy starting from the fragment 6 [51] (1 eq.),which can be synthesized from commercially available2-(diphenylphosphino)benzoic acid and N-hydroxysuccinimide (NHS) in the presence of N, N′-Diisopropylcarbodiimide (DIC) in DCM (Figure 2). (S)-5was synthesized according to established protocols by Pfaltz et. al. starting from benzamide and L-serine methyl ester in three steps in 63% overall yield. Fragment 6 was then reacted with either commercially available (R,R)-1,2-diamino-cyclohexane (2 eq.), or (S,S)-7(2 eq.) to form (R,R)-11, or (S,S)-8[52,53] and then coupled with (S)-5 using the TBTU and HOBT in the presence of Hünig’s base to form (S,R,R)-10 and (S,S,S)-9 in good yields (Figure 2) [54,55].

**Figure 3:**
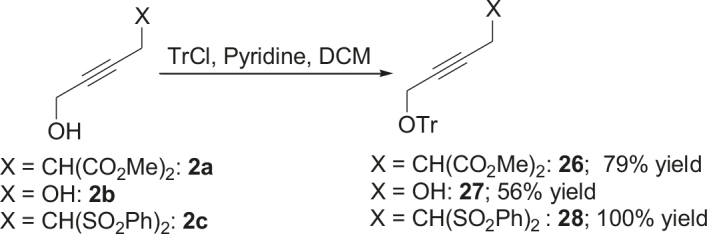
Protection of the hydroxyl group.

It is assumed that the free hydroxyl group in 2a may have been partially responsible for the moderate *ee* observed for 4, so going forward, it was decided to protect the hydroxyl group with it’s trityl ether [56,57]. The yields of the protected alcohols are moderate to excellent depending on the propargylic alcohol derivatives (Figure 3). The protected substrates were then reexamined using the optimized conditions from Table 1. Substrates in Figure 3 were then tested in the conditions of Table 1 (Table 2).

After the protection of the alcohol with a trityl group, very high enantioselectivities using the reaction conditions in Table 2 and Table 3 could be obtained. Two types of reactions were tested with ligands L _std_ , ( *S,S,S* )-9 and ( *S,R,R* )-10. First, the formation of C-C bond quarter centers of carbon atom (Table 2), then the formation of C-O bond by the use of 4- (trityloxy) but-2-yn-1-ol as nucleophilic source (Table 3) has been tested. The new ligands ( *S* , *S* , *S* )-9 and ( *S* , *R* , *R* )-10 also showed very high enantioselectivities and yields in the optimized reaction conditions (see entries 2,4,10, and 14,Table 2 and entry 4, Table 3). However, the enantioselectivities of the allylic alkylation decreased, if different types of nucleophiles were used under the same reaction conditions (entries 5–8, Table 2). It is assumed that the malonate carbonyl group (entries 1–4, Table 2) or sulfonyl group (entries 13–14, Table 2) may coordinate to the active catalyst in situ, giving rise to higher enantioselectivities.

**Table 2:** Substrate scope with trityl protected propargylic alcohol derivatives.

Entry	Substrate(nucleophile)	Substrate (electrophile)	Product	Reaction condition	Yield(%)a	ee(%)b
1	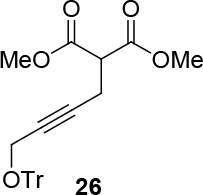	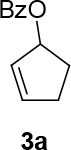	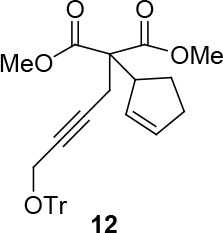	[η ^3^ -C _3_ H _5_ PdCl] _2_ (2.5%), PPh3 (30%), NaH (1.3 eq.), THF (0.2 M)	70	-
2				[η ^3^ -C _3_ H _5_ PdCl] _2_ (2.5%), (S,R,R)-10(7.5%),NaH/DBU (2/2 eq.),THAB (1 eq.),DCM (0.25 M), r.t., 1 day	82	99
3				[η ^3^ -C _3_ H _5_ PdCl] _2_ (2.5%), L _std_ (R,R) (7.5%), NaH/DBU (2/2 eq.),THAB (1 eq.),DCM (0.25 M), -78°C → r.t., 1 day	73	98
4				[η ^3^ -C _3_ H _5_ PdCl] _2_ (2.5%) (S,S,S)-9(7.5%), NaH/DBU (2/2 eq.), THAB (1 eq.), DCM (0.25 M), r.t., 1 day	81	99
5		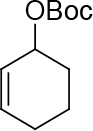	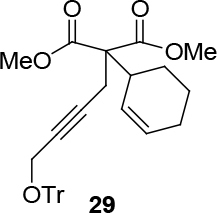	[η ^3^ -C _3_ H _5_ PdCl] _2_ (2.5%), PPh3 (30%), NaH (2 eq.) DCM (0.1 M), r.t., 1 day	90	-
6				[η ^3^ -C _3_ H _5_ PdCl] _2_ (2.5%), L _std_ (R,R), (7.5%), NaH/DBU (2/2 eq.), THAB, DCM (0.21 M), r.t., 1 day	60	91
7		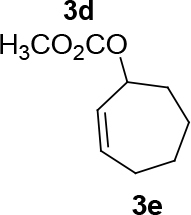	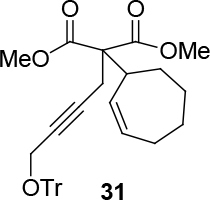	[η ^3^ -C _3_ H _5_ PdCl] _2_ (2.5%), PPh3 (30%), DCM, Δ, 1 day.	66	-
8				[η ^3^ -C _3_ H _5_ PdCl] _2_ (2.5%), L _std_ (R,R), NaH/DBU, THAB, DCM (0.25 M), -78°C → r.t., 1 day	51	99c
9		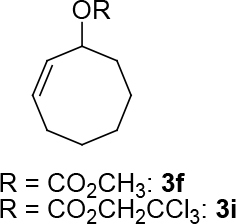	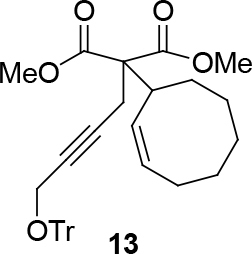	R = CO2CH3;[η ^3^ -C _3_ H _5_ PdCl] _2_ (2.5%), PPh3 (30%), DBU (3 eq.), THF (0.25 M), Δ, 3 days.	37	-
10				R = CO2CH2CCl3;[η ^3^ -C _3_ H _5_ PdCl] _2_ (2.5%) (S,R,R)-8 (7.5%), NaH/DBU (2/2 eq.), THAB, DCM (0.1 M), r.t., 1 day	75	93
11				R = CO2CH3; Pd2(dba)3CHCl _3_ (2.5%), L _std_ (S,S) (7.5%), DBU (2 eq.), THF (0.25 M), r.t., 1day	37	71
12				R = CO2CH3; [η ^3^ -C _3_ H _5_ PdCl] _2_ (2.5 %), L _std_ (R,R) (7.5%), NaH/DBU (2/2 eq.), THAB, DCM (0.25 M), r.t., 1 day	40	99
13	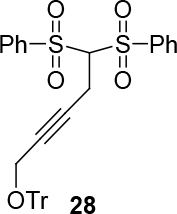	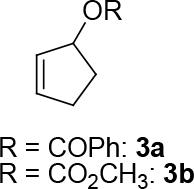	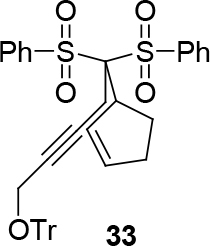	R = CO2Me; Pd2(dba)3CHCl _3_ , PPh3 (30%), NaH (1.3 eq.), THF (0.3 M), r.t., 1 day.	66	-
14				R = COPh; [η ^3^ -C _3_ H _5_ PdCl] _2_ (2.5%), (S,R,R)-10 (7.5%), NaH (1.3 eq.), THF (0.25 M), r.t., 1 day	74	70

aIsolated yield. bThe ee was determined by chiral HPLC except for entry 8. cThe ee was determined by chiral GC after deprotection.

Unfortunately, the novel synthesized ligand has brought low enantioselectivity in the formation of C-O bonds (Table 3). However, the yields varied from moderate to very good. One side trityl-protected but-2-yne-1,4-ol was tested as a nucleophilic source in the presence of ( *S,R,R* )-10. Probably the nucleophilic source also plays a crucial role in maintaining a high enantioselectivity. Why the product is obtained as a racemic mixture in the presence of chial( *S,R,R* )-10 ligand, when 2,2,2-trichloroethyl cyclohex-2-enyl carbonate is used as the electrophile source, is not clear (entry 2, Table 3). When the seven-membered ring is inserted, the *ee* increases up to 57% under the same reaction condition (entry 4, Table 3).

**Table 3:** The formation of C-O bonds with the use of (S,R,R)-10 ligands.

1	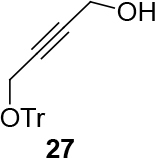	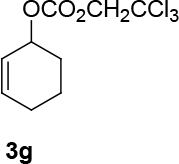	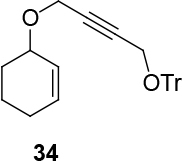	Pd2(dba)3CHCl _3_ (2.5%); dppp (7.5 %), NaH (1.3 eq.), THF (0.3 M), r.t., 1 day.	86	-
2				[η ^3^ -C _3_ H _5_ PdCl] _2_ (2.5%), (S,R,R)-10(7.5%), NaH/DBU (2/2 eq.), THAB, DCM (0.25 M), r.t., 1 day.	82	0
3		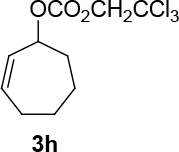	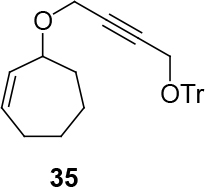	Pd2(dba)3CHCl _3_ (2.5%), dppp (7.5 %), NaH (1.3 eq.), THF (0.3 M), 1 day.	49	-
4				[η ^3^ -C _3_ H _5_ PdCl] _2_ (2.5%), (S,R,R)-10(7.5%), NaH/DBU (2/2 eq.), THAB, DCM (0.25 M), r.t., 1 day.	67	57

**Figure 4:**
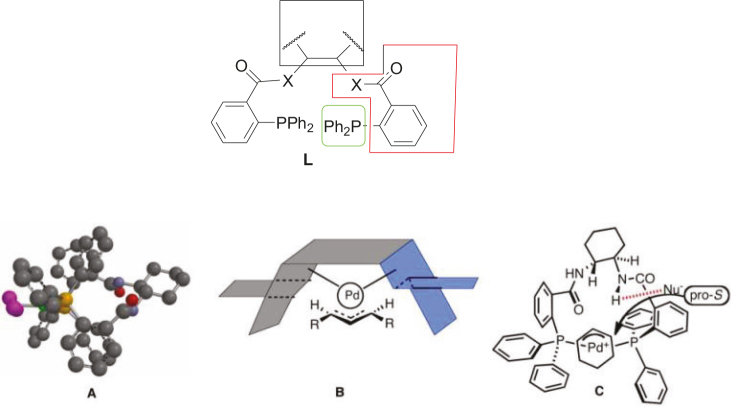
L: General representation of the Trost-ligand(s). A: Crystal structure of ligand Lstd. B: 3D proposed model for active catalyst π-allylpalladium complex in situ. C: 3D proposed model for π-cyclohexenyl palladium complex in situ.

However, the steric effect of the substrates was not studied in detail in the literature. Only the steric effect of the ligands on the substrates is studied in detail. The reasons for the high *ee* in Table 2 have been previously calculated theoretically in detail for the Trost-ligand L _std_ in the transition state of the Pd-catalyst [58–61]. The crystal structure, a 3D calculated model of the active catalyst of *π* -allyl palladium complex in situ, is shown in Figure 4 [58–61]. The ligand L consists mainly of three important parts: a linker (red), a chiral scaffold (black), and a binding post (green). According to Trost et. al., the linkers play a crucial role in nucleophile attacking the *π* -cyclohexenyl palladium complex. This step occurs in palladium catalytic cycles asymmetrically (Structure C, Figure 4). To demonstrate that the linkers (red) are very important in the enantioselectivity of the products, Trost et. al. synthesized a new chiral ligand without any additional group on binding post and tested it in the Pd-catalyzed AAA [58].

**Figure 5:**
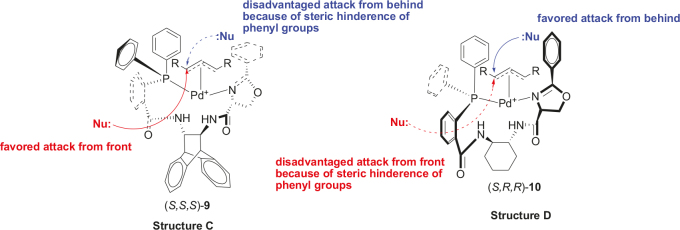
Proposed attacks of nucleophile (Nu:) in transition states of the catalysts using (S,S,S)-9 (Structure C) and using (S,R,R)-10 (Structure D) for the symmetrical π-allyl electrophile (R-CHCHCH-R+).

Based on the theoretical calculations of ligand L _std_ (Structure B) and other similar bisoxazoline ligands [62], the structures in Figure 5 were proposed that suggest transition states of the two catalysts Pd-( *S,S,S* )-9 and Pd-( *S,R,R* )-10. It is assumed that the phenyl group of the amide group and the phenyl group of the oxaziline group sterically hinder the nucleophilic attack from one side and this leads to high enantioselectivity in the reaction. Here, symmetrical electrophilic (R-CHCHCH-R+) was only used to understand better the steric hindrance of the ligands ( *S,S,S* )-9 and ( *S,R,R* )-10 to nucleophiles (Figure 5). It is still unclear whether the trityl group will act as an additional steric hindrance in substrates to increase the *ees* of the products. To determine whether the trityl group plays a special role, one has to test with another protective group for the same substrates in the same reaction conditions. Further detailed DFT calculations using complicated electrophile and nucleophile for the transition states of ligands ( *S,S,S* )-9 and ( *S,R,R* )-10 will be published in a separate manuscript.

Because a free hydroxyl group is required for the subsequent ruthenium-catalyzed redox cycloisomerization, a series of the *ee* -pure products 12 to 13 were deprotected with catalytic BiCl3 in DCM [63,64]. The corresponding deprotected malonates were isolated in good yield. Those compounds (Table 4) were then tested in cycloisomerization reactions using two types of Ru-catalysts.

**Table 4:** Removing the trityl group using BiCl3.

Entry	Substrate	Product(ee-pure)	Time	Yield (%)
1	12	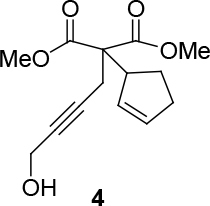	15 min	77
2	29	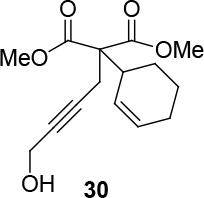	1 h	75
3	31	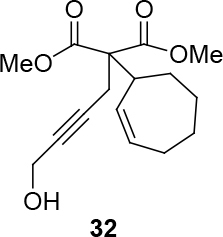	1 h	70
4	13	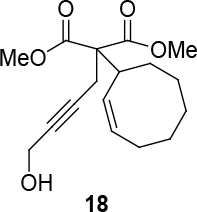	1 h	69

**Figure 6:**
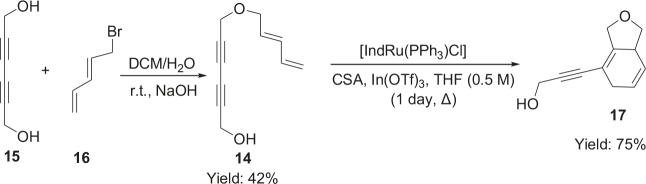
Testing for cycloisomerization with different type of substrates.

Different types of substrates for redox cycloisomerisation were tested as well. With 6-(( *E* )-penta-2,4-dienyloxy)hexa-2,4-diyn-1-ol 14 using [IndRu(PPh3)2Cl], CSA, and In(OTf)3 in THF, which was synthesized starting from commercially available compounds 15 and 16 (Figure 6). Compound 17 was obtained as a cyclization product in 75% yield. Here, [IndRu(PPh3)2Cl] 1 catalyzes a Diels-Alder type reaction (Figure 6).

In Table 5, enantiomerically pure and racematic substrates were used. The yield of the products in Table 5 were compared using racemic substrate and enantiomerically pure substrates. The same yields were obtained for all used substrates. The products, where the enantiomerically pure substrates were used, were not checked for enantiomeric purity.

**Table 5:** Redox cycloisomerization using [IndRu(PPh3)2Cl] to form bicycles.

Entry	Substrates	Product	Yield (%)
1	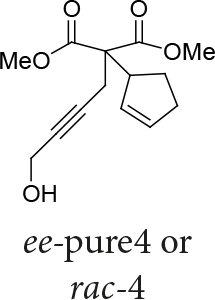	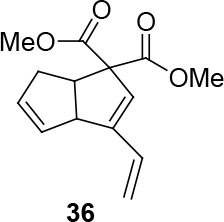	78
2	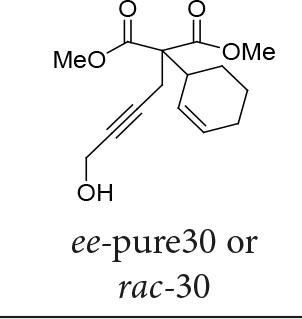	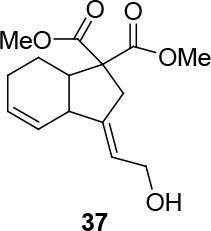	63
3	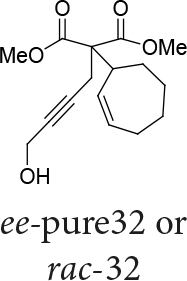	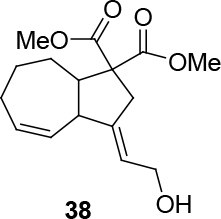	70
4	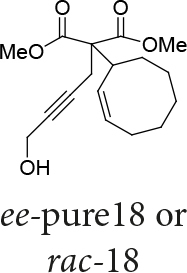	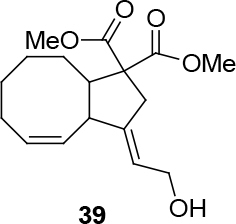	50

Two different catalysts in Table 5 were tested for cycloisomerization. [CpRu(CH3CN)3]PF6 showed no chemoselectivity, and resulted in the formation of 6 different unidentified products by LC-MS! However, [IndRu(PPh3)2Cl] 1 catalyzed the reaction in the presence of camphorsulfonic acid (CSA) and In(OTf)3 in THF (n = 2,3,4) or acetone (n = 1). The elimination of water if n=1 (entry 1, Table 5) was obtained. It is assumed that acetone, which is used as a solvent only for entry 1 (Table 5) may play a role as a solvent in the elimination of water. However, in the reactions carried out in THF, the corresponding alcohols were obtained (entries 2–4, Table 5). A series of racemic compounds *rac* **-** 4, 18, 30, and 32 were first tested and the results were compared with the series of the enantiomerically enriched compounds 4, 18, 30, and 32. All of the compounds gave the same products with the same yields (Table 5).

**Figure 7:**
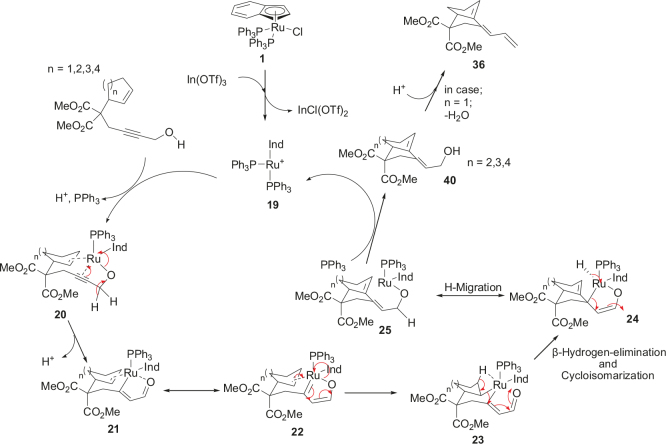
Proposed reaction mechanism for redox cycloisomerization.

It is proposed that the catalytic cycle occurs by H-migration followed by cycloaddition to form the bicycle products shown in Table 5 (Figure 7). The first step in the catalytic cycle is to form the active catalyst 19 from the catalyst 1 in situ using In(OTf)3, which abstracts chloride ion.

It is assumed that the reaction mechanism proceeds as follows; first, In(OTf)3 reacts with chlorine to form the active catalyst 19. The coordination of the enyne starting material results in the elimination of triphenyl phosphine and a proton to form 20. The triple bond reacts afterwards to double bond to form Intermediate 21, which has resonance with 22 (Figure 7).

The Intermediates 21 and 22 should be armchair shape conformation and prefer the formation of metalacyclohexane 23. Through *β* -hydrogen elimination and cycloisomerization, intermediate 23 reacts to form intermediate 24. After that, the hydrogen migration between intermediates 24 and 25 occurs. It is assumed that the Ru-H bond is one of the key intermediates to different types of bicycle compounds. Intermediate 24 can be formed by a second H-migration to carbon atom. The intermediates 24 and 25 can be formed by H-migration. In the following step, the catalyst detaches from the intermediate 25 by reductive elimination to form the corresponding products (entries 2–4, Table5) and regenerate the active catalyst 19. The mechanism was proposed using another Ru-catalyzed cycloisomerization reaction, which is published in ref. [18,19,21,22,34,35, and 48]. No additional experiments were carried out to detect the intermediates.

## 3. Conclusion

In summary, a novel method was developed successfully to synthesize asymmetrically a series of [n.3.0] bicyclic molecules (n = 3–6) bicyclodienes of various ring sizes in three steps starting from dimethyl 2-(4-(trityloxy)but-2-ynyl) malonate 3a[65] or symmetrically in two steps starting from dimethyl 2-(4-hydroxybut-2-ynyl)malonate 2a via palladium-catalyzed allylic alkylation and Ru-catalyzed cycloisomerization. For that, two new ligands (S,S,S)-9 and (S,R,R)-10 were developed and tested for the palladium-catalyzed asymmetric allylic alkylation (Figure 6). These ligands were used in the asymmetric cycloalkylation of trityl protected propargylic alcohols and resulted in excellent ee and up to very good yields (Table 2). [n.3.0] bicyclic molecules (n = 3–6) in Table 5 were then synthesized from the synthesized ee-pure or racemic propargylic compounds in Table 4 using the catalyst [IndRu(PPh3)2Cl]. The supplementary material is available free of charge on the website of Turkish Journal of Chemistry Turkish Journal of Chemistry (2020). Effective Synthesis of Bicyclodienes Via Palladium-Catalyzed Asymmetric Allylic Alkylation and Ruthenium-Catalyzed Cycloisomerization [online]. Website: u2374 [accessed 10th october 2020].

## 4. Experimental section

### 4.1. General Information

NMR spectra were obtained using a 400 spectrometer (1H at 300, 400, or 500 MHz, and 13C at 101 MHz). Chemical shifts for 1H NMR spectra are reported in parts per million (ppm) from tetramethylsilane with the solvent resonance as the internal standard (CDCl3: δ 7.26 ppm). Chemical shifts for 13C NMR spectra are reported in parts per million (ppm) from tetramethylsilane with the solvent as the internal standard (CDCl3: δ 77.0 ppm). IR spectra were obtained as liquid films with a spectrometer. GC−MS was obtained using electron ionization. HRMS was obtained with an LCMS-IT-TOF mass spectrometer. Unless stated otherwise, commercial reagents were used without further purification. All reagents were weighed and handled in air at room temperature.

Dimethyl 2-(4-hydroxybut-2-ynyl)malonate; compound 2a, Table 1):

K2CO3 anhydrous (5.56 g, 40.26 mmol, 3 eq.) was charged in a dried flask, and dried with a flame in a high vacuum for 2 min. Then 60 ml dry acetone was added under argon atmosphere. Dimethylmalonate (1.77 g, 13.42 mmol, 1 eq.) were dissolved in 20 mL dry acetone and added to a suspension of potassium carbonate under argon. The suspension was stirred for 20 min. under argon at room temperature. 1,4-Dihydroxy-2-butyne (2 g, 13.42 mmol, 1 eq.) and 20 mL acetone were added to the mixture slowly. The reaction mixture was stirred vigorously under argon atmosphere at room temperature for 2 days. After 2 days, the reaction mixture was filtered off and the solvent was removed under reduced pressure. The product was isolated by column chromatography (eluent: PE:EA = 2:1 and then pure EA). A 68% yield of colorless oil (1.826 g) was isolated. 1H-NMR (300 MHz, CHLOROFORM- *d* ) d ppm 2.84 (dt, J = 7.69, 2.14 Hz, 2 H) 3.60 (t, J = 7.69 Hz, 1 H) 3.77 (bs, 6 H) 4.23 (t, J = 1.95 Hz, 2 H). The NMR-data correspond to the published data (see supporting info of ref.[49]).

**4.2. General procedure 1 for the synthesis of cycloalkyl-2-enyl benzoate compound:** 3a [66–69] Cycloalkene (2.5 eq.) was placed in an oven dry flask with reflux condenser. CuBr (0.18 % eq.) was added to cycloalkene. Mixture was washed with argon. Mixture was heated neat under argon and reflux condenser (in the case of cyclopentene to 45–50 °C, in the case of cyclohexene to 80 °C, in the case of cycloheptene to 90 °C, and in the case of cis-cyclooctene to 95 °C). BzOOt-Bu (1 eq.) was added neat over 24 h using a syringe pump via syringe to the mixture drop by drop at those temperatures. After a while, the reaction has the color of ocean blue. The product was isolated by silica gel chromatography by directly adding the neat reaction mixture to silica gel. (Eluent for all: PE/EtOAc: 10/1). The isolated products correspond to the published NMR-data [66–69].

**4.3. General procedure 2 for the synthesis of racemic compounds:rac-12, rac-29, rac-31, rac-13, rac-33, rac-34 and rac-35 (entries 1,5,7,9, and 13, Table 2 and entries 1, 3 Table 3):** Flask A: An oven dried microwave flask was charged with Pd-cat. (Pd2(dba)3CHCl _3_ or [η ^3^ -C _3_ H _5_ PdCl] _2_ ; for the use of Pd-cat.; see Table 2, reaction conditions) (2.5 mol% eq.) and ligand: triphenyl phosphine (30 mol % eq.) or dppp (7.5 mol% eq.). These were washed with argon. The mixture was dissolved in one third mL of THF or DCM (for the solvents see the table). The reaction mixture was stirred for 30 min. in room temperature. Substrate (electrophile) (1 eq.) was added in one third / mL of THF or DCM to the reaction mixture. This reaction mixture was stirred for 30 min. at room temperature under argon. Flask B: In a dry microwave flask charged with NaH (for the required equivalent see table; 60% in oil). This was washed immediately with argon. Substrate (nucleophile) (1 eq.) was added in one third / mL of THF or DCM (for the solvent see table) to sodium hydride, slowly under argon flow to reach the concentration in table. The reaction mixture was stirred for 10 min. more at room temperature. The contents of flask A were injected via syringe to the reaction mixture. The whole reaction mixture was heated or stirred at room temperature for one day (for the conditions see Table 2). The reaction mixture was washed with water, extracted three times with DCM and dried over magnesium sulfate. The solvent was evaporated under reduced vacuum. The product was isolated by silica gel chromatography.

**4.4. General procedure 3 for the synthesis of ee-pure compounds 12, 29, 31, 13, 31, 33, 34, 35 (entries 2,3,4,6,8,10,11,12, and 14, Table 2 and entries 2,4, Table 3):** Flask A: Pd-cat. Pd2(dba)3CHCl _3_ or [η ^3^ -C _3_ H _5_ PdCl] _2_ ; for the used Pd-cat.; see Table 2; 2.5 % eq. and the corresponding ligand (7.5 mol% eq.) was dissolved in one third / mL of solvent. The reaction mixture was stirred for 30 min. at room temperature. Electrophile (1 eq., see table 2) in one third / mL of solvent was added to the reaction mixture. This reaction mixture was stirred for 20 min. at room temperature under argon. Flask B: Flame dried flask was charged with NaH (for eq. see table; 60% in mineral oil) and with THAB (1 eq., in table if necessary). The flask was washed under argon flow. Nucleophile substrate (1 eq.) in one third of solvent was added to NaH slowly under an open flow of argon gas, where the generation of hydrogen gas was observed. After addition, the reaction was stirred for a further 5 min. DBU (2 eq.) was added to this mixture (if necessary, see Table 2). The reaction mixture was stirred for 10 min. at room temperature. The contents of flask A were injected via syringe to the reaction mixture. The whole reaction mixture was stirred under the described conditions (see table) for one day. The reaction mixture was washed with water, extracted three times with DCM and dried over magnesium sulfate. The solvent was evaporated under reduced vacuum. The product was isolated by column chromatography (eluent: Hexane: EtOAc = 4:1).

**4.5. General procedure 4 for the synthesis of 26–28 (Figure 3):** An oven dried flask was charged with propargyl alcohol (2a, 2b, or 2c) (1 eq.), in case of but-2-yne-1,4-diol (2b) was charged with 3 eq.), 4-Dimethylaminopyridine (DMAP) (20% eq.) and trityl chloride (2 eq.). Those were washed under argon. The required amount of a half ml of DCM, was added to the mixture, and subsequently pyridine (2 eq.) in the other half ml of DCM, was added to reaction mixture. The reaction mixture was stirred for a day at room temperature (Eluent: Hexane: EA: 100:30). The rest was filtered. The liquid was evaporated. The products were isolated by silica gel chromatography (eluents: hexane: EtOAc = 100:30). The isolated products correspond to the published NMR-data in ref.[56,57,63].

**4.6. General procedure 5 for the deprotection of alcohol group from trityl group (Table 4):** Starting material (1 eq.) was dissolved in acetonitrile, so that the solution concentration could be 1 M. The solution was washed under argon. BiCl3 (5 mol%) was subsequently added in one portion to the solution. The solution was stirred as much as necessary (for stirring time see table). More stirring in r.t or addition of more than 5 mol% BiCl3 showed the decomposition of the products under this condition. The products were directly purified and isolated by silica gel chromatography (eluent for all: PE/EtOAc: 100:30). For yield of the products see Table 2. For the procedure with other compounds see ref. [64].

**4.7. General procedure 6 for Ru-catalyzed cycloisomerization: synthesis of 36-39 (Table 5):** [IndRu(PPh3)2Cl] 1 (0.01172 mmol, 3 mol% eq.) and camphorsulfonic acid (CSA) (0.01955, 5 mol%) were added to an oven dried flask. The flask was washed under argon. Starting material (4, 18, 30 and 32) (0.391 mmol, 1 eq.) in 0.5 mL THF (in case n = 1, 0.5 mL acetone was used as solvent) was added to the solid mixture. The reaction mixture was stirred for 5 min. In(OTf)3 (0.01172 mmol, 3 mol) in 0.5 mL was subsequently added to the reaction mixture. The reaction mixture was heated in the case of THF at 63 °C and in the case of acetone at 54 °C for one day. The product was directly isolated by silica gel chromatography (eluent for all: PE/EtOAc: 10:4).

**4.8. General procedure 7 synthesis of the new ligands (S,S,S)-9 and (S,R,R)-10:** An oven dried microwave flask was charged with (S)-5 (1 eq.), one of the compounds: (R,R)-11 (1.04 eq.) or (S,S)-7 (1.04 eq.) and the coupling reagents: 1-Hydroxybenzotriazol (HOBT, 1.12 eq.) and O-(Benzotriazol-1-yl)-N,N,N’,N’-tetramethyluronium-tetrafluoroborate (TBTU, 1.04 eq.). The flask was washed with argon. Diisopropylethylamine (3 eq.) was added to the reaction mixture, subsequently DMF was added under argon, until the concentration of the solution was 0.1 M. The reaction mixture was stirred for a day at room temperature. The reaction was washed with 20 mL saturated NH4Cl solution and extracted three times with DCM. The organic phase was dried over Mg2SO4. The solvent was evaporated. The products were isolated by silica gel chromatography (eluent: EtOAc/Hexane: 2/1). The rest of the viscous oil after the chromatography was recrystallized over Et2O and then pentane was added drop by drop until observation of full precipitation (recrystallization at room temperature) to obtain the white solid (S,R,R)-10 in 85% yield or pale yellow solid (S,S,S)-9in 76% yield.

**4.9. General procedure 8 for the synthesis of the fragments (S,S)-8 and (R,R)-11:** An oven dry flask was charged with compound 6 (879 mg, 2.179 mmol, 1 eq.). The flask was washed under argon. DCM (25 mL) is added to the mixture. Then, (R,R)-1,2-diaminocyclohexane or (S,S)-1,2-trans-antracene diamine ((S,S)-7) (1.03 g, 4.359 mmol, 2 eq.), which are dissolved in 10 mL DCM, were added drop wise using syringe pump over 5 h. The reaction mixture was stirred under argon for a day. A total of 25 milliliters of water was added to the solution. The organic phase was extracted three times with each 10 mL DCM. The solvent was evaporated. The product was isolated by silica gel chromatography (eluent: CH2Cl2/MeOH: 5/1).

**4.10. General procedure 9: for the racemate of the products: (4-(cycloalkyl-2-enyloxy)but-2-ynyloxy)-triphenyl methane: Compounds rac-34 and rac-35 (entries 1,3, table 3):** Flask A: Pd2(dba)3CHCl _3_ (0.05 mmol, 2.5 mol% eq.) and 1,3-bis(diphenylphosphino)propane (dppp) (0.0375 mmol, 7.5 mol%) were charged in an oven dry microwave flask. The flask was washed with argon, 0.7 ml THF was added and stirred for 30 min at room temperature. 2,2,2-trichloroethyl cycloalk-2-enyl carbonate (0.5 mmol, 1 eq.) was added in 0.5 ml to the reaction mixture. The reaction mixture was stirred for 30 min. Flask B: An oven dry microwave flask was charged with NaH (0.65 mmol, 1.3 eq.) and 4-(trityloxy)but-2-yn-1-ol (0.5 mmol, 1 eq.). The flask was washed subsequently with argon and 0.5 mmol THF was added to the mixture (hydrogen gas generation). The mixture was stirred for 10 min. in r.t. The contents of flask A were injected into the flask B via syringe. The whole reaction mixture was stirred for a day in r.t. Five milliliters of water were added to the reaction mixture. The organic phase was extracted three times with DCM. The solvent was dried over Mg2SO4 and filtered. The solvent was evaporated. The product was isolated by silica gel chromatography (Eluent: PE/EtOAc = 10/3).

**4.11. General procedure 10 for the enantioselective synthesis of the products: Compounds 34 and 35 (entries 2,4, Table 3):** (4-(cycloalkyl-2-enyloxy)-but-2-ynyloxy)-triphenyl methane: Flask A: [η ^3^ -C _3_ H _5_ PdCl] _2_ (0.006225 mmol, 2.5 mol% eq.) and ligands (S,S,S)-9 or (S,R,R)-10 (0.01867, 7.5 mol%). The flask was washed with argon and 0.25 ml DCM was added and stirred for 30 min at room temperature. 2,2,2-trichloroethyl cycloalkyl-2-enyl carbonate (0.25 mmol, 1 eq.) was added in 0.25 ml to the reaction mixture. The reaction mixture was stirred for 30 min. Flask B: An oven dry microwave flask was charged with NaH (0.50 mmol, 2 eq.), 4-(trityloxy)but-2-yn-1-ol[57] (0.25 mmol, 1 eq.) and THAB (0.25 mmol, 1 eq.) was added. The flask was washed subsequently with argon and 0.25 mmol DCM was added to the mixture. DBU (0.50 mmol, 150 μL, 2 eq.) was added (hydrogen gas generation!). The mixture was stirred for 10 min in r.t. The contents of flask A were injected into flask B via syringe. The whole Five milliliters of water were added to the reaction mixture. The organic phase was extracted three times with DCM. The solvent was dried over Mg2SO4 and filtered. The solvent was evaporated. The product was isolated by silica gel chromatography. (Eluent: PE/EtOAc = 10/3).

**4.12 General procedure 11 for the synthesis of 2,2,2-trichloroethyl cycloalkyl-2-enyl carbonate:** compounds 3g, 3h Cycloalk-2-enol (1 eq.) and 2,2,2-trichloroethyl chloroformate (1.1 eq.) were dissolved in two of thirds ml in DCM, which is needed for the 0.5 M concentration. The solution was washed with argon. The solution was cooled to 0 °C using an ice bath. Pyridine (6 eq.) was dissolved in one third of ml of DCM. The solution was slowly added via syringe. The reaction was left under stirring, warming to room temperature for one day. The reaction was quenched using saturated CuSO4. The organic phase was extracted three times with DCM and one time with Et2O. The organic phase was dried over Mg2SO4 and filtered. The solvent was evaporated. The product was isolated using silica gel chromatography (eluent: PE/EtOAc = 10/1).

**4.13. General procedure 12 for the synthesis of cyclopent-2-enyl methyl carbonate (3b), cyclohept-2-enyl methyl carbonate (3e), cyclooct-2-enyl methyl carbonate (3f) (Table 2):** Cycloalk-2-enol (1 eq.) was added to an oven dry flask. Dry DCM was added, so that the whole solution could be concentrated to 0.33 M. The whole solution was washed with argon. Dry pyridine (6 eq.) was added to the solution via syringe. The solution was cooled to 0 °C with an ice bath. Methyl chloroformate (2.5 eq.) was added slowly to the solution via syringe. The reaction mixture was allowed, under stirring, to warm to room temperature over 1 day. The reaction mixture was washed twice with HCl (1 M, 2.5 eq.). The organic phase was washed with saturated NaHCO3. The organic phase was dried over Mg2SO4 and filtered. The solvent was evaporated. The isolated products correspond to the published NMR-data.

*cyclopent-2-enyl benzoate: compound 3a:* colorless liquid. Yield: 70%: It was synthesized according to the published procedure [66–69]: the spectroscopy data correspond to the data in ref. [66–69].

*cyclopent-2-enyl methyl carbonate (3b), (Z)-cyclohept-2-enyl methyl carbonate (3e), (Z)-cyclooct-2-enyl methyl carbonate (3f):* colorless liquids. It was synthesized in three steps starting from cycloheptene to form cyclohept-2enyl benzoate (see: general procedure 6), hydrolysis with KOH/MeOH [70] (heating for 1 day at 63 °C) concentration of the solution, and protection of alcohol using methyl chloroformate according to the general procedure 11.

*tert-butyl cyclohex-2-enyl carbonate: compound 3d:* colorless liquid. It was synthesized in three steps starting from cyclohexene to form cyclohex-2enyl benzoate (see: general procedure 6), hydrolysis with KOH/MeOH[70] (heating 1 day at 63°C) and protection of alcohol using Boc2 according to the published procedure in ref. [70].

*2,2,2-trichloroethyl cyclohex-2-enyl carbonate (3g) (2,2,2-trichloroethyl (Z)-cyclohept-2-enyl carbonate (3h):* colorless liquids. It was synthesized in three steps starting from cyclohexene and *cis* -cycloheptene to form cyclohex-2-enyl benzoate and ( *Z* )-cyclohept-2-enyl benzoate (see: general procedure 6), hydrolysis with KOH/MeOH [70] and protection of alcohol using 2,2,2-trichloroethyl chloroformate according to the general procedure 11.

*2,2,2-trichloroethyl cycloalkyl-2-enyl carbonate: compounds 3g, 3h:*

It was synthesized in three steps starting from cyclohexene or cycloheptene to form cyclohex-2-enyl benzoate or cyclohept-2-enyl benzoate (see: general procedure 6), hydrolysis with KOH/MeOH [70] (heating overnight at 63 °C) and protection of alcohol using 2,2,2-trichloroethyl chloroformate according to the general procedure 11.

*2,2,2-trichloroethyl cyclohexyl-2-enyl carbonate: compound 3g:* colorless liquid.

1H NMR (400 MHz, CHLOROFORM- *d* ) δ ppm 1.58 - 1.71 (m, 1 H) 1.72 - 1.95 (m, 3 H) 1.95 - 2.19 (m, 3 H) 4.77 (s, 2 H) 5.19 (br. s., 1 H) 5.63 - 6.14 (m, 2 H).

FT-IR (thin film, cm-1) = 3397, 3035, 2949, 2871, 2835, 1753, 1652, 1437, 1379, 1246, 1169, 1098, 1049, 1003, 966, 912, 811, 784, 726, 570.

*2,2,2-trichloroethyl cycloheptyl-2-enyl carbonate: compound 3h:* colorless liquid.

1H NMR (300 MHz, CHLOROFORM- *d* ) δ ppm 1.30 - 1.52 (m, 1 H) 1.61 - 1.87 (m, 3 H) 1.91 - 2.37 (m, 4 H) 4.70 - 4.84 (m, 2 H) 5.15 - 5.48 (m, 1 H) 5.63 - 6.14 (m, 2 H).

FT-IR (thin film, cm-1) = 3506, 3031, 3000, 2858, 1748, 1655, 1568, 1446, 1380, 1323, 1128, 1095, 1065, 923, 895, 821, 785, 730, 570.

*Dimethyl 2-(cyclopent-2-enyl)-2-(4-hydroxybut-2-ynyl) malonate: compound 4:* colorless liquid.1H NMR (400 MHz, CHLOROFORM- *d* ) δ ppm 1.66 - 1.81 (m, 2 H) 1.96 - 2.16 (m, 2 H) 2.29 (br. s., 2 H) 2.85 (br. s., 2 H) 3.61 (m, 1 H) 3.71 (s, 3 H) 3.74 (s, 3 H) 4.21 (br. s., 2 H) 5.68 - 5.86 (m, 2 H).13C NMR (101 MHz, CHLOROFORM- *d* ) δ ppm 23.76, 25.45, 29.96, 31.97, 49.15, 51.48, 52.68, 52.87, 60.53, 81.56, 131.13, 133.06, 170.62, 170.82. FT-IR (thin film, cm-1) = 3442, 2954, 1732, 1436, 1267, 1079, 916, 731. LRMS (ESI) *m* / *z* : calcd for C14H18NaO5 [M+Na]+ 289.1053; found 289.10.

( *S* )-5 (Figure 2): white solid: yield: 63% (over three steps): the spectroscopy data correspond to the established data in ref. [54,55].

6 (Figure 2): pale yellow solid: yield: 98%; the spectroscopy data correspond to the published data in ref.[51–53].

*(S,S)-8:* pale yellow solid; yield: 89%; M.P. 195-197 °C. = +61.67 ( *c* = 0.26, CHCl _3_ ); 1H NMR (400 MHz, CHLOROFORM- *d* ) δ ppm 3.57 - 3.86 (m, 1 H) 4.04 - 4.27 (m, 2 H) 5.74 (d, *J* =5.87 Hz, 1 H) 6.87 (dd, *J* =6.94, 4.40 Hz, 1 H) 7.01 - 7.42 (m, 23 H) 7.47 (dd, *J* =6.85, 3.13 Hz, 1 H). 13C NMR (101 MHz, CHLOROFORM- *d* 6) δ ppm 48.69, 51.32, 60.43, 6056, 124.76, 124.89, 125.64, 126.50, 126.63, 126.89, 127.02, 128.18, 128.21, 128.88, 128.94, 129.00, 129.06, 129.24, 129.30, 130.56, 133.96, 134.15, 134.31, 135.91, 136.07, 136.91, 136.99, 138.68, 139.31, 140.49, 140.86, 141.06, 142.45, 169.54. 31P NMR (162 MHz, CHLOROFORM- *d* ) δ ppm -10.29. FT-IR (thin film, cm-1): 3409, 3284, 3050, 2950, 1652, 1583, 1502, 1484, 1371, 1293, 1265, 1138, 1090, 1026, 997, 908, 635, 560. HRMS (ESI) m/z: calcd for C35H29N2NaOP [M+Na]+ 547.1915; found 547.1939.

*(S,S,S)-9 (* Figure 2 *):* pale yellow solid; Yield: 76%; M.P.: 95-96°C, = +137.49 ( *c* = 0.355, CHCl _3_ ). 1H NMR (400 MHz, BENZENE- *d* 6) δ ppm 3.77 (t, *J* =9.29 Hz, 1 H) 3.88 - 4.02 (m, 1 H) 4.10 (br. s., 1 H) 4.21 - 4.39 (m, 1 H) 4.61 - 4.74 (m, 1 H) 4.88 (br. s., 1 H) 6.63 (d, *J* =9.00 Hz, 1 H) 6.74 (q, *J* =6.97 Hz, 2 H) 6.85 (d, *J* =4.11 Hz, 3 H) 6.89 - 6.94 (m, 1 H) 6.95 - 7.20 (m, 13 H) 7.29 - 7.48 (m, 6 H) 7.51 (br. s., 2 H) 7.91 (d, *J* =6.65 Hz, 2 H). 13C NMR (101 MHz, BENZENE- *d* 6) δ ppm 34.98, 38.09, 48.98, 49.80, 54.41, 58.41, 68.46, 68.54, 124.34, 124.98, 125.38, 126.11, 126.27, 126.55, 126.70, 127.38, 127.44, 127.68, 127.93, 128.09, 128.16, 128.22, 128.28, 128.38, 128.44, 128.48, 129.60, 131.65, 133.57, 133.77, 133.86, 134.07, 134.56, 137.01, 137.23, 138.23, 138.34, 138.90, 139.18, 139.31, 140.00, 140.96, 141.38, 142.21, 142.49, 161.58, 164.76, 168.47, 170.98. 31P NMR (162 MHz, BENZENE- *d* 6) δ ppm -10.13. FT-IR (thin film, cm-1) 3297, 3051, 2951, 1654, 1581, 1520, 1465, 1454, 1360, 1291, 1265, 1155, 1091, 1026, 746, 697, 638, 559. HRMS (ESI) *m* / *z* : calcd for C45H36N3NaO3P [M+Na]+ 720.2392; found 720.2384.

(S,R,R)-10 (Figure 2): white solid: yield: 85%; M.P. 80-81°C; = -45.05 ( *c* = 0.25, CHCl _3_ ). 1H NMR (400 MHz, BENZENE- *d* 6) δ ppm 0.84 - 1.04 (m, 3 H) 1.11 (t, *J* =6.94 Hz, 1 H) 1.32 (br. s., 2 H) 1.79 (d, *J* =11.15 Hz, 1 H) 1.99 (d, *J* =9.98 Hz, 1 H) 3.63 - 3.95 (m, 2 H) 4.05 - 4.19 (m, 1 H) 4.58 - 4.68 (m, 2 H) 6.46 (d, *J* =7.83 Hz, 1 H) 6.79 - 6.92 (m, 2 H) 7.05 (d, *J* =2.35 Hz, 10 H) 7.28 (d, *J* =5.28 Hz, 2 H) 7.36 (t, *J* =6.94 Hz, 2 H) 7.44 (d, *J* =7.43 Hz, 1 H) 7.54 - 7.63 (m, 1 H) 8.08 - 8.19 (m, 2 H). 13C NMR (101 MHz, BENZENE- *d* 6) δ ppm 15.24, 24.65, 24.80, 30.48, 31.89, 35.12, 38.11, 53.42, 53.52, 65.49, 69.58, 69.91, 127.44, 127.54, 127.68, 127.93, 128.05, 128.24, 128.31, 128.63, 129.44, 131.23, 133.76, 133.88, 133.96, 134.08, 136.77, 137.00, 138.58, 138.71, 141.51, 141.76, 162.12 165.28, 168.50, 171.62. 31P NMR (162 MHz, BENZENE- *d* 6) δ ppm -9.44. FT-IR (thin film, cm-1) = 3298, 3054, 2934, 2857, 1643, 1526, 1478, 1450, 1324, 1264, 1207, 1146, 1088, 1026, 967, 929, 854, 853, 779, 741, 696. HRMS (ESI) *m* / *z* : calcd for C35H34N3NaO3P [M+Na]+ 598.2236; found 598.2258.

(R,R)-11 (Figure 2): white solid: yield: 72% the spectroscopy and -rotation data correspond to the published data in ref. [51–53].

*Dimethyl 2-(cyclopent-2-enyl)-2-(4-(trityloxy)but-2-ynyl)malonate: compound* 12 *:* white viscous liquid.

1H NMR (400 MHz, CHLOROFORM- *d* ) δ ppm 1.67 - 1.86 (m, 1 H) 1.98 - 2.19 (m, 1 H) 2.31 (t, *J* =7.04 Hz, 1 H) 2.87 (s, 2 H) 3.67 (t, 2 H) 3.71 (s, 3 H) 3.74 (s, 3 H) 5.64 - 5.92 (t, *J* = 7 Hz, 2 H) 6.93 - 7.63 (m, 15 H). 13C NMR (101 MHz, CHLOROFORM- *d* ) δ ppm 23.51, 25.24, 29.71, 31.74, 48.95, 52.39, 52.56, 60.32, 79.55, 80.97, 87.42, 127.11-128.55 (15C), 131.10, 132.67, 143.58 (3 C) 170.43, 170.58. IR (thin film, cm-1) = 3473, 3058, 3000, 2958, 2952, 2854, 2245, 1960, 1734, 1596, 1491, 1447, 1368, 1261, 1223, 1156, 1055, 911,802, 764, 706, 632, 583. HRMS (ESI) *m* / *z* : calcd for C33H32NaO5 [M+Na]+ 531.2147; found 531.2161.

*Dimethyl 2-((Z)-cyclooct-2-enyl)-2-(4-(trityloxy)but-2-ynyl) malonate: compound* 13 *:* colorless viscous liquid.

1H NMR (300 MHz, CHLOROFORM- *d* ) δ ppm 1.33 - 1.74 (m, 8 H) 1.84 - 2.40 (m, 2 H) 2.53 (s, 2 H) 3.69 (s, 3 H) 3.71 (br. s, 1 H) 3.77 (s, 3 H) 5.39 - 5.88 (m, 2 H) 7.13 - 7.54 (m, 15 H).

13C-NMR (101 MHz, CHLOROFORM- *d* ) δ ppm 15.06, 23.46, 26.01, 26.57, 29.02, 33.52, 35.20, 52.05, 53.61, 54.84, 84.07, 87.59, 127.34, 127.50, 128.14, 128.83, 130.31, 143.77, 172.67. FT-IR (thin film, cm-1) 3485, 3025, 2930, 2859, 1747, 1653, 1619, 1437, 1380, 1261, 1154, 1097, 1022, 937, 849, 809, 757, 718, 568.

*6-((E)-penta-2,4-dienyloxy)hexa-2,4-diyn-1-ol: compound* 14 *:* yellow liquid.

1H NMR (300 MHz, CHLOROFORM- *d* ) *δ* ppm 4.11 (d, *J* =6.35 Hz, 2 H) 4.24 (s, 2 H) 4.36 (d, *J* =5.86 Hz, 2 H) 5.03 - 5.34 (m, 2 H) 5.64 - 5.83 (m, 1 H) 6.13 - 6.50 (m, 2 H). FT-IR (thin film, cm-1) = 3405, 2920, 2851, 1723, 1688, 1462, 1364, 1263, 1048, 903, 782, 731.

*3-(1,3,3a,6-tetrahydroisobenzofuran-7-yl)prop-2-yn-1-ol: compound* 17 *:* pale yellow solid.

1H NMR (400 MHz, CHLOROFORM- *d* ) δ ppm 1.78 (br. s., 1 H) 2.71 - 2.96 (m, 2 H) 3.21 (br. s., 1 H) 3.27 - 3.35 (m, 1 H) 4.24 (t, *J* =7.24 Hz, 1 H) 4.41 (br. s., 2 H) 4.50 (br. s., 2 H) 5.53 - 5.97 (m, 2 H). 13C (101 MHz, CHLOROFORM- *d* ) δ ppm 31.41, 40.96, 51.84, 69.85, 72.32, 83.92, 90.75, 108.71, 122.53, 126.79, 146.57.

FT-IR (thin film, cm-1) = 3399, 3031, 2924, 2857, 1721, 1425, 1362, 1319, 1269, 1238, 1157, 1098, 1070, 1023, 977, 939, 785, 748, 699, 610.

*Dimethyl 2-((Z)-cyclooct-2-enyl)-2-(4-hydroxybut-2-ynyl)-malonate: compound* 18 *:* pale yellow liquid.

1H NMR (500 MHz, CHLOROFORM- *d* ) δ ppm 1.28 - 1.74 (m, 8H) 1.86 - 2.35 (m, 2H) 2.80 (d, *J* =7.81 Hz, 2 H) 3.58 (t, *J* =7.57 Hz, 1 H) 3.73 (br. s., 1 H) 3.74 (s, 6 H) 4.64 (br. s., 2 H) 5.41 - 5.73 (m, 2 H).

13C-NMR (101 MHz, CHLOROFORM- *d* ) δ ppm 14.39, 19.00, 23.41, 26.54, 28.96, 35.13, 50.89, 53.14, 55.76, 60.76, 82.18, 83.67, 130.05, 130.46, 154.34, 168.51. IR (thin film, cm-1) 3485, 3025, 2930, 2859, 1747, 1658, 1619, 1437, 1380, 1261, 1154, 1097, 1022, 937, 809, 718, 757, 568.

*(5,5-bis(phenylsulfonyl)pent-2-ynyloxy)triphenylmethane: compound* 28 *:* white solid.

It was synthesized in three steps starting from commercially available bis(phenylthio)methane first to form bis(phenylsulfonyl)methane using *m* -CPBA according to the published procedure in ref. [70]. The Bis(phenylthio)methane was treated with 4-bromobut-2-yn-1-ol [71] to form 5,5-bis(phenylsulfonyl)pent-2-yn-1-ol according to the same procedure: dimethyl 2-(4-hydroxybut-2-ynyl)malonate. Titel compound was then synthesized according to general procedure 2. 1H NMR (400 MHz, CHLOROFORM- *d* ) δ ppm 3.14 (d, *J* =4.89 Hz, 2 H) 3.48 (br. s., 2 H) 4.27 - 4.78 (t, *J* = 6.01, 1 H) 7.18 - 7.35 (m, 10 H) 7.36 - 7.46 (m, 5 H) 7.53 (t, *J* =7.43 Hz, 4 H) 7.57 - 7.67 (m, 2 H) 8.00 (d, *J* =7.63 Hz, 4 H). 13C NMR (101 MHz, CHLOROFORM- *d* ) δ ppm 17.48, 53.26, 77.23, 77.55, 77.87, 78.71, 81.01, 82.12, 87.67, 127.58, 128.29, 128.86, 129.43, 130.04, 135.15, 138.12, 143.61.

*Dimethyl 2-(cyclohex-2-enyl)-2-(4-(trityloxy)but-2-ynyl)-malo-nate: compound* 29 *:* white viscous liquid.

1H NMR (300 MHz, CHLOROFORM- *d* ) δ ppm 1.45 - 1.67 (m, 1 H) 1.82 (dd, *J* =10.01, 5.37 Hz, 3 H) 1.96 (br. s., 2 H) 2.77 - 2.99 (m, 2 H) 3.14 (m, 1 H) 3.72 (s, 3 H) 3.76 (s, 3 H) 4.21 (s, 2 H) 5.60 - 5.81 (m, 2 H). 13C NMR (101 MHz, CHLOROFORM- *d* ) δ ppm 22.51, 23.08, 25.09, 24.50, 29.96, 39.17, 51.50, 52.64, 52.84, 60.77, 81.52, 81.64, 127.57, 129.40, 170.41, 170.65. FT-IR (thin film, cm-1) = 3411, 2924, 1734, 1447, 1261, 1052, 706. HRMS (ESI) *m* / *z* : calcd for C34H34NaO5 [M+Na]+ 545.2304; found 545.2280.

*Dimethyl 2-(cyclohex-2-enyl)-2-(4-hydroxybut-2-ynyl)malonate: compound* 30 *:* colorless liquid.

1H NMR (300 MHz, CHLOROFORM- *d* ) δ ppm 1.45 - 1.65 (m, 4 H) 1.77-1.88 (m, 2 H) 1.96 (br. s., 1 H) 2.81 - 2.99 (m, 2 H) 3.08-3.16 (m, 1 H) 3.73 (s, 3 H), 3.76 (s, 3H) 4.21 (s, 2 H) 5.60 - 5.81 (m, 2 H).13C NMR (101 MHz, CHLOROFORM- *d* ) 22.51, 23.08, 25.09, 24.50, 29.96, 39.17, 51.50, 52.64, 52.84, 60.77, 81.52, 81.64, 127.57, 129.40, 170.41, 170.65. FT-IR (thin film, cm-1) = 3411, 2924, 1734, 1447, 1261, 1052, 706. HRMS (ESI) *m* / *z* : calcd for C15H20NaO5 [M+Na]+ 303.1208; found 303.1209.

*Dimethyl 2-((Z)-cyclohept-2-enyl)-2-(4-(trityloxy)but-2-ynyl)-malonate: compound* 31 *:* pale yellow viscous liquid.

1H NMR (400 MHz, CHLOROFORM- *d* ) δ ppm 0.85 (br. s., 2 H) 1.49 - 2.24 (m, 4 H) 2.91 (br. s., 2 H) 3.25 (br. s., 1 H) 3.54 - 3.83 (m, 6 H) 5.55 - 6.09 (m, 2 H) 6.84 - 7.96 (m, 15 H).13C-NMR (101 MHz, CHLOROFORM- *d* ) δ ppm 24.04, 26.32, 28.22, 30.00, 31.77, 43.30, 53.75, 61.03, 79.90, 81.34, 87.59, 127.35, 128.15, 128.82, 133.00, 143.86, 170.73, 170.79. IR (thin film, cm-1) = 3398, 3024, 2923, 2853, 1734, 1596, 1491, 1447, 1369, 1274, 1223, 1156, 1053, 975, 899, 802, 746, 706, 631. HRMS (ESI) *m* / *z* : calcd for C35H36NaO5 [M+Na]+ 559.2460; found 559.2486.

*Dimethyl 2-((Z)-cyclohept-2-enyl)-2-(4-hydroxybut-2-ynyl)-malonate: compound* 32 *:* colorless liquid.

1H NMR (500 MHz, CHLOROFORM- *d* ) δ ppm 1.48 - 1.87 (m, 4 H) 1.99 - 2.10 (m, 2 H) 2.11 - 2.22 (m, 2 H) 2.89 (s, 2 H) 3.18 (d, *J* =6.35 Hz, 1 H) 3.72 - 3.78 (br. s., 6 H) 4.22 (s, 2 H) 5.61 - 5.95 (m, 2 H). 13C NMR (101 MHz, CHLOROFORM- *d* ) δ ppm 24.10, 26.21, 28.14, 31.65, 43.36, 51.25, 52.86, 61.05, 81.15, 81.81, 132.45, 132.77, 170.72, 170.79.FT-IR (thin film, cm-1) = 3476, 3025, 2924, 2851, 1733, 1436, 1309, 1277, 1223, 1144, 1070, 1044, 1019. HRMS (ESI) *m* / *z* : calcd for C16H22NaO5 [M+Na]+ 317.1365; found 317.1372.

*(5-(cyclopent-2-enyl)-5,5-bis(phenylsulfonyl)pent-2-ynyloxy)-triphenylmethane:compound 33:* white solid.

1H NMR (400 MHz, CHLOROFORM- *d* ) δ ppm 2.06 - 2.43 (m, 4 H) 3.23 (m, 2 H) 3.64 (br. s., 2 H) 3.94 - 4.27 (m, 1 H) 5.77 (d, *J* =13.30 Hz, 2 H) 7.19 - 7.36 (m, 10 H) 7.38 - 7.48 (m, 5 H) 7.52 (q, *J* =7.30 Hz, 4 H) 7.58 - 7.69 (m, 2 H) 8.09 - 8.24 (m, 4 H). 13C-NMR (101 MHz, CHLOROFORM- *d* ) δ ppm 23.64, 25.28, 32.09, 48.02, 53.68, 78.20, 83.05, 87.79, 92.30, 127.52, 128.25, 128.68, 128.83, 123.07, 123.14, 133.27, 134.84, 138.28, 138.38, 143.69. FT-IR (thin film, cm-1) = 3385, 3061, 2924, 2854, 2254, 1718, 1583, 1447, 1383, 1368, 1145, 1075, 1056, 977, 909, 732, 730, 631, 591, 572.

*(4-(cyclohex-2-enyloxy)but-2-ynyloxy)triphenylmethane: compound* 34 *:* colorless liquid.

1H NMR (400 MHz, CHLOROFORM- *d* ) δ ppm 1.56 - 2.37 (m, 6 H) 3.74 - 3.94 (m, 2 H) 4.66 - 4.89 (m, 2 H) 5.18 (d, *J* =1.96 Hz, 1 H) 5.66 - 6.12 (m, 2 H) 7.08 - 7.44 (m, 15 H). 13C (101 MHz, CHLOROFORM- *d* ) δ ppm 18.49, 24.81, 28.16, 53.03, 55.49, 72.41, 78.86, 84.12, 87.52, 124.71, 127.19, 127.94, 128.59, 133.57, 143.34, 154.27.

*(4-((Z)-cyclohept-2-enyloxy)but-2-ynyloxy)triphenylmethane compound* 35 *:* white viscous liquid.

1H NMR (400 MHz, CHLOROFORM- *d* ) δ ppm 1.48 - 1.82 (m, 4 H) 1.85 - 2.31 (m, 4 H) 3.82 (br. s., 2 H) 4.75 (br. s., 2 H) 5.14 - 5.40 (m, 1 H) 5.57 - 5.96 (m, 2 H) 7.02 - 7.61 (m, 15 H).13C NMR (101 MHz, CHLOROFORM- *d* ) δ ppm 26.31, 26.45, 28.40, 32.70, 53.02, 55.58, 78.77, 84.19, 87.52, 127.20, 127.94, 128.58, 132.05, 132.64, 143.32, 154.13. FT-IR (thin film, cm-1) = 3414, 3058, 3031, 2927, 2859, 1746, 1597, 1490, 1447, 1377, 1322, 1258, 1156, 1057, 967, 964, 899, 789, 764, 705, 632.

*Dimethyl 6,6a-dihydro-3-vinylpentalene-1,1(3aH)-dicarboxylate: compound* 36 *:* colorless liquid.

1H NMR (400 MHz, CHLOROFORM- *d* ) δ ppm 1.93 - 2.12 (m, 1 H) 2.48-2.61 (m, 1 H) 3.72 (s, 3 H) 3.74 (s, 3H) 3.75 - 3.81 (m, 1 H) 4.03 (br. s., 1 H) 5.22 (d, *J* =10.76 Hz, 1 H) 5.35 (d, *J* =17.61 Hz, 1 H) 5.60 - 5.70 (m, 2 H) 5.89 (br. s., 1 H) 6.44 (dd, *J* =17.41, 10.76 Hz, 1 H). 13C NMR (101 MHz, CHLOROFORM- *d* ) δ ppm 35.50, 45.91, 52.58, 53.16, 56.20, 69.14, 117.81, 125.81, 130.53, 130.69, 132.04, 147.93, 171.21. HRMS (ESI) *m* / *z* : calcd for C14H16NaO4 [M+Na]+ 271.0946; found 271.0938.

*(3E)-dimethyl 3,3a,7,7a-tetrahydro-3-(2-hydroxyethylidene)-2H-indene-1,1(6H)-dicarboxylate: compound 37:* colorless viscous liquid.

1H NMR (400 MHz, CHLOROFORM- *d* ) δ ppm 1.01 - 1.16 (m, 1 H) 1.21 - 1.37 (m, 2 H) 1.92 - 2.18 (m, 2 H) 2.73 - 3.01 (m, 2 H) 3.16 - 3.36 (m, 2 H) 3.70 - 3.76 (m, 7 H) 4.15 (d, *J* =6.41 Hz, 2 H) 5.35 - 5.46 (m, 1 H) 5.67 - 5.90 (m, 2 H). 13C NMR (101 MHz, CHLOROFORM- *d* ) δ ppm 21.35, 24.83, 34.67, 42.76, 43.32, 52.89, 53.14, 60.90, 62.68, 122.25, 126.27, 145.36, 170.45, 172.36. FT-IR (thin film, cm-1) = 3405, 2922, 1733, 1435, 1267, 1042. HRMS (ESI) *m* / *z* : calcd for C15H20NaO5 [M+Na]+ 303.1208; found 303.1206.

*(3E,4Z)-dimethyl 3,3a,6,7,8,8a-hexahydro-3-(2-hydroxyethyl-idene)azulene-1,1(2H)-dicarboxylate: compound 38:* colorless viscous liquid.

1H NMR (400 MHz, CHLOROFORM- *d* ) δ ppm 1.55 - 1.84 (m, 4 H) 1.98 - 2.07 (m, 2 H) 2.11 - 2.20 (m, 3 H) 2.88 (s, 2 H) 3.16 (d, *J* =6.56 Hz, 1 H) 3.74 (s, 6 H) 4.21 (br. s., 2 H) 5.57 - 5.99 (m, 2 H). 13C NMR (101 MHz, CHLOROFORM- *d* ) δ ppm 24.06, 26.25, 28.20, 30.01, 31.70, 43.41, 51.57, 52.80, 52.88, 61.05, 81.60, 81.73, 126.31, 132.50, 132.80, 151.93, 170.73. FT-IR (thin film, cm-1) = 3443, 2924, 2851, 1732, 1436, 1276, 1222, 1070, 1046. HRMS (ESI) *m* / *z* : calcd for C16H22NaO5 [M+Na]+ 317.1365; found 317.1358.

*(3E,4Z)-dimethyl 3,3a,7,8,9,9a-hexahydro-3-(2-hydroxyethyl-idene)-2H-cyclopenta[8]annulene-1,1(6H)-dicarboxylate: compound* 39 *:* pale yellow viscous liquid.

1H NMR (300 MHz, CHLOROFORM- *d* ) δ ppm 1.30 - 1.77 (m, 8 H) 1.89 - 2.37 (m, 3 H) 2.84 (d, *J* =7.57 Hz, 2 H) 3.26 (s, 1 H) 3.52 - 3.63 (m, 1 H) 3.70 - 3.88 (m, 5 H) 4.16 (d, *J* =7.57 Hz, 1 H) 4.68 (s, 2 H) 5.43 - 5.85 (m, 3 H). 13C (101 MHz, CHLOROFORM- *d* ) δ ppm 19.02, 23.42, 25.97, 26.55, 28.97, 35.14, 50.91, 53.14, 55.76, 75.95, 76.51, 77.17, 83.68, 130.08, 130.47, 154.32, 168.48. FT-IR (thin film, cm-1) = 3404, 2930, 2858, 1742, 1437, 1340, 1279, 1262, 1154, 1023, 938. HRMS (ESI) *m* / *z* : calcd for C17H24NaO5 [M+Na]+ 331.1521 found: 331.1509.

Supplementary Materials Click here for additional data file. The supplementary material is available free of charge on the website of Turkish Journal of Chemistry.
The NMR, FT-IR, and HR-MS spectral data, as well as HPLC and GC separation conditions for all new compounds (PDF).
