# Supplementary material for: Effective synthesis of bicyclodienes via palladium-catalyzed asymmetric allylic alkylation and ruthenium-catalyzed cycloisomerization
Source: Turk J Chem. 2020 Dec 16;44(6):1445–62. doi: 10.3906/kim-2004-81 (PMC7772093; doi:10.3906/kim-2004-81)

Effective synthesis of bicyclodienes via palladium-catalyzed asymmetric allylic alkylation and ruthenium-catalyzed cycloisomerization.

Nizam Havare*

*Department of Chemistry, Stanford University, Stanford, California 94305-5580, United States

Index

^{1}H-NMR, ^{13}C-NMR, FT-IR, LR-MS and HR-MS for

Compound (*R*,*R*)- **8** 3-7
Compound (*S*,*R*,*R*)-**10** 8-12
Compound (*S*,*R*,*R*)-**9** 12-16
Compound (*S*,*S*,*S*)- **9** 17-21
Compound **4** 22-25
Compound **12** 26-29
Compound **30** 30-33
Compound **29** 34-38
Compound **32** 39-42
Compound **31** 43-46
Compound **18** 47-49
Compound **13** 50-51
Compound **36** 52-54
Compound **37** 55-58
Compound **38** 59-62
Compound **39** 63-66
Compound **14** 67-68
Compound **17** 69-71
Compound **3g** 72-73
Compound **3h** 74-75
Compound **28** 76-77
Compound **33** 78-80
Compound **34** 81-83
Compound **35** 84-87

HPLC and GC data for

Table 2, entry 1: compound "*rac*-**12**" 87
Table 2, entry 3: compound "*enantioenriched* **12**" 88
Table 2, entry 4: compound "*enantioenriched* **12**" 89
Table 2, entry 2: compound "*enantioenriched* **12**" 90
Table 2, entry 5: compound "*rac*-**29**" 91
Table 2, entry 6: compound "*enantioenriched* **29**" 92
Table 2, entry 7: compound "*rac*-**32**" 93
Table 2, entry 8: compound "*enantioenriched* **32**" 94
Table 2, entry 9: compound "*rac*-**13**" 95
Table 2, entry 11: compound "*enantioenriched* **13**" 96
Table 2, entry 12: compound "*enantioenriched* **13**" 97

Table 2, entry 10: compound "*enantioenriched* **13**" 98
Table 3, entry 3: compound "*rac*-**35**" 99
Table 3, entry 4: compound "*enantioenriched* **35**" 100
Table 2, entry 13: compound "*rac*-**33**" 101
Table 2, entry 14: compound "*enantioenriched* **33**" 102

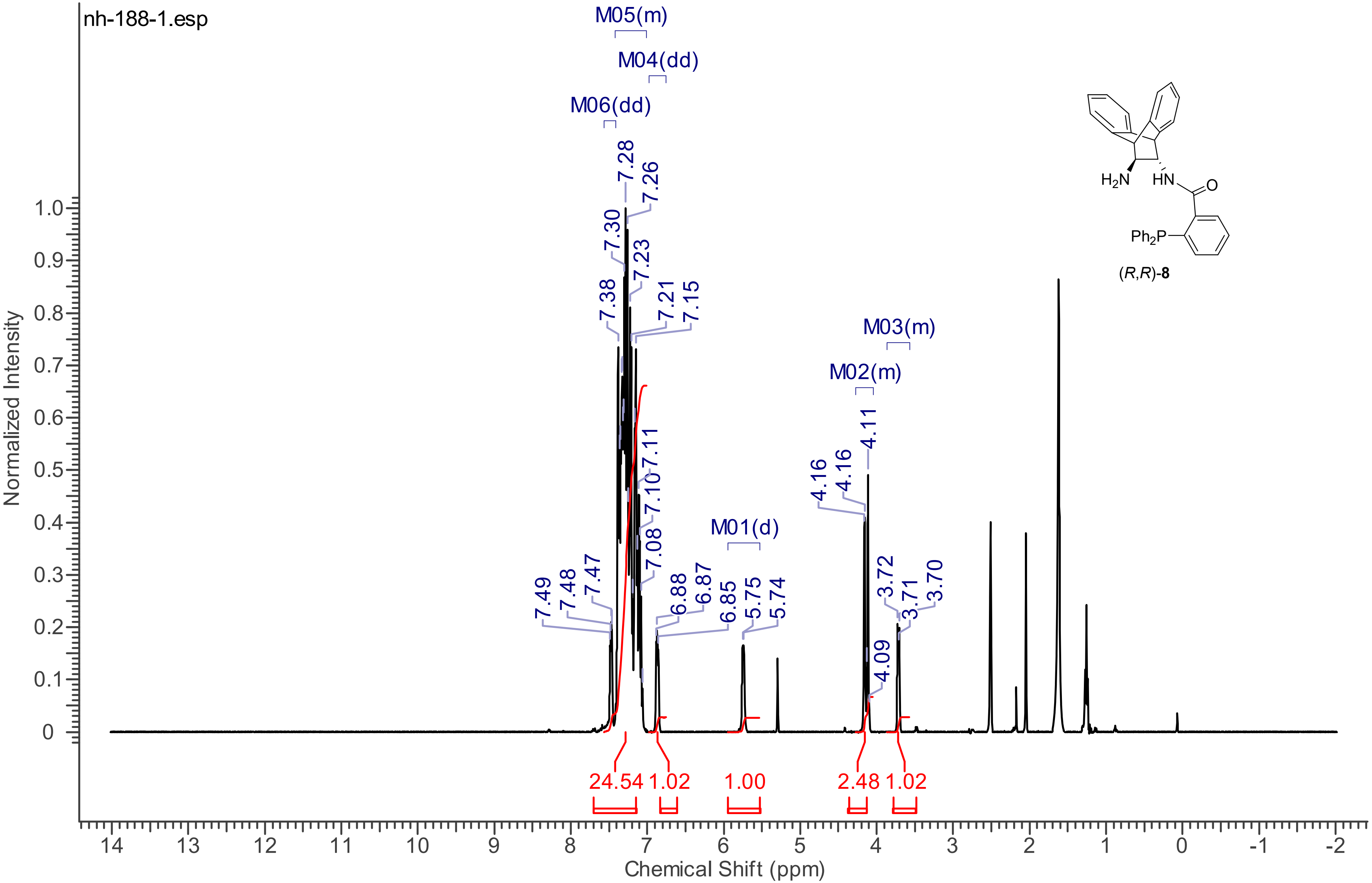
nh-188-1.esp
M05(m)
M04(dd)
M06(dd)
M01(d)
M02(m)
M03(m)
24.54 1.02 1.00 2.48 1.02
Normalized Intensity
Chemical Shift (ppm)
H2N
HN
O
Ph2P
(R,R)-8

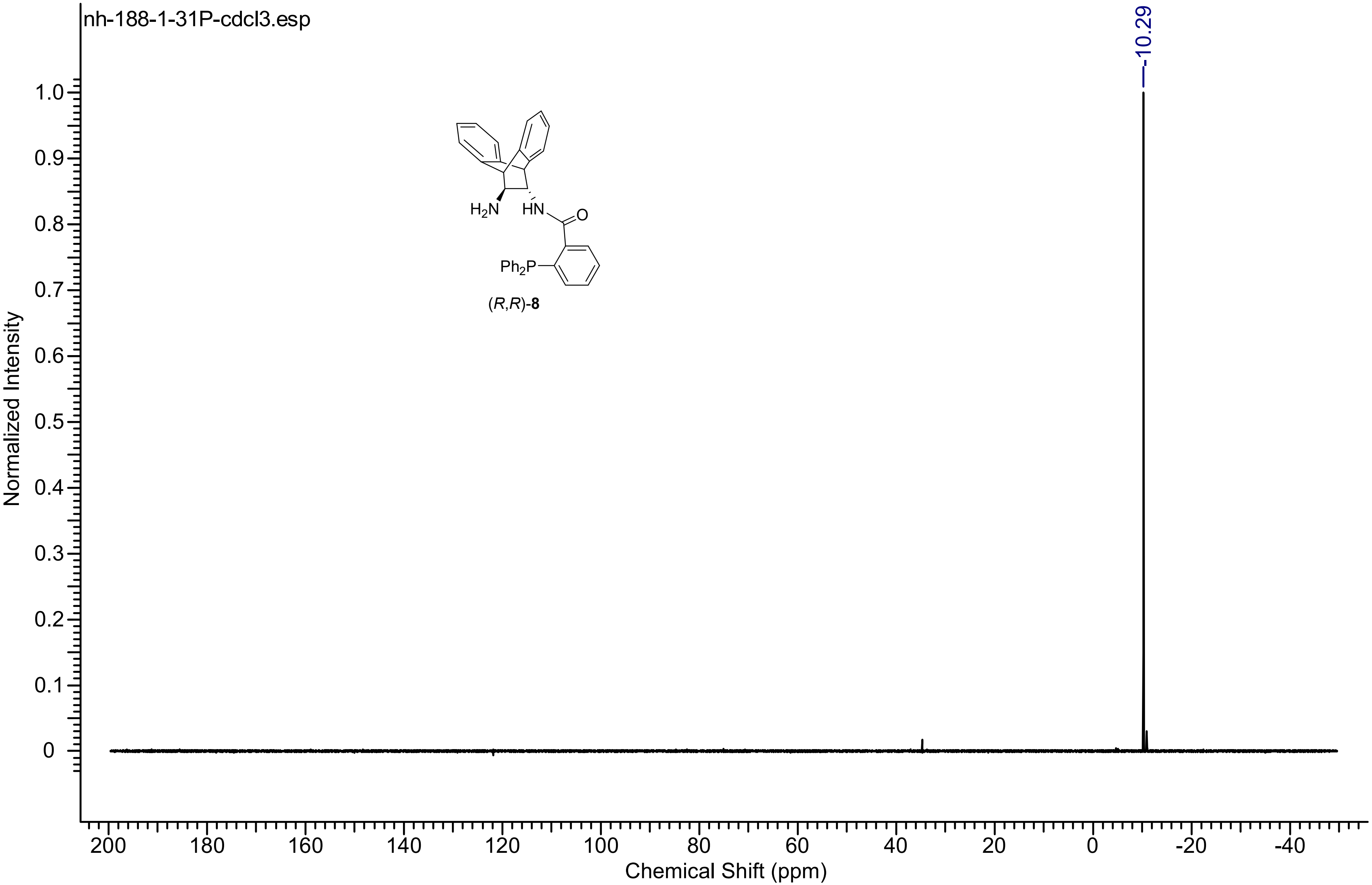
nh-188-1-31P-cdcl3.esp
-10.29
H2N
HN
O
Ph2P
(R,R)-8
Normalized Intensity
Chemical Shift (ppm)

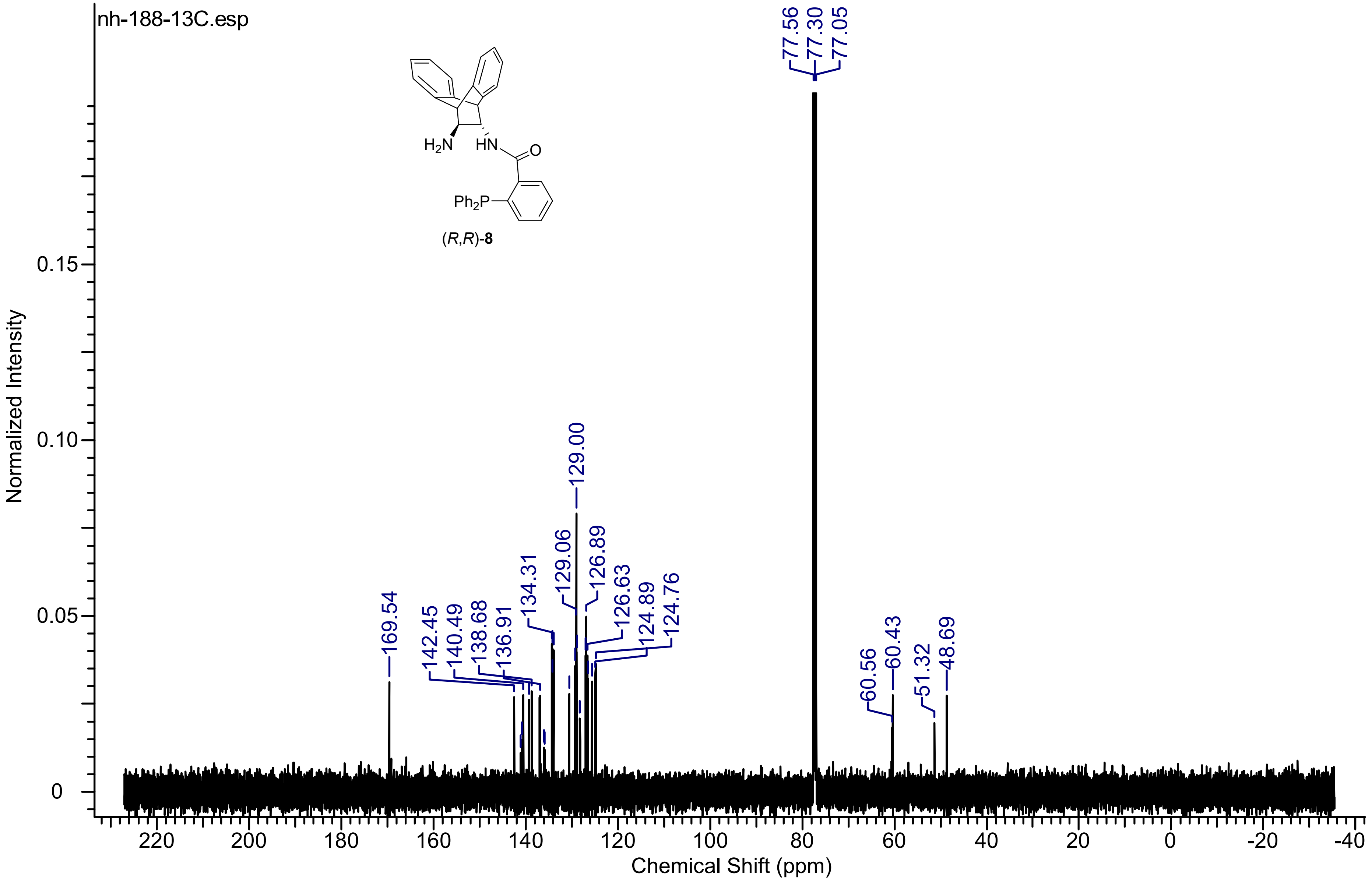
nh-188-13C.esp
H2N
HN
O
Ph2P
(R,R)-8
77.56
77.30
77.05
169.54
142.45
140.49
138.68
136.91
134.31
129.06
129.00
126.89
126.63
124.89
124.76
60.56
60.43
51.32
48.69
Normalized Intensity
0.15
0.10
0.05
0
220
200
180
160
140
120
100
80
60
40
20
0
-20
-40
Chemical Shift (ppm)

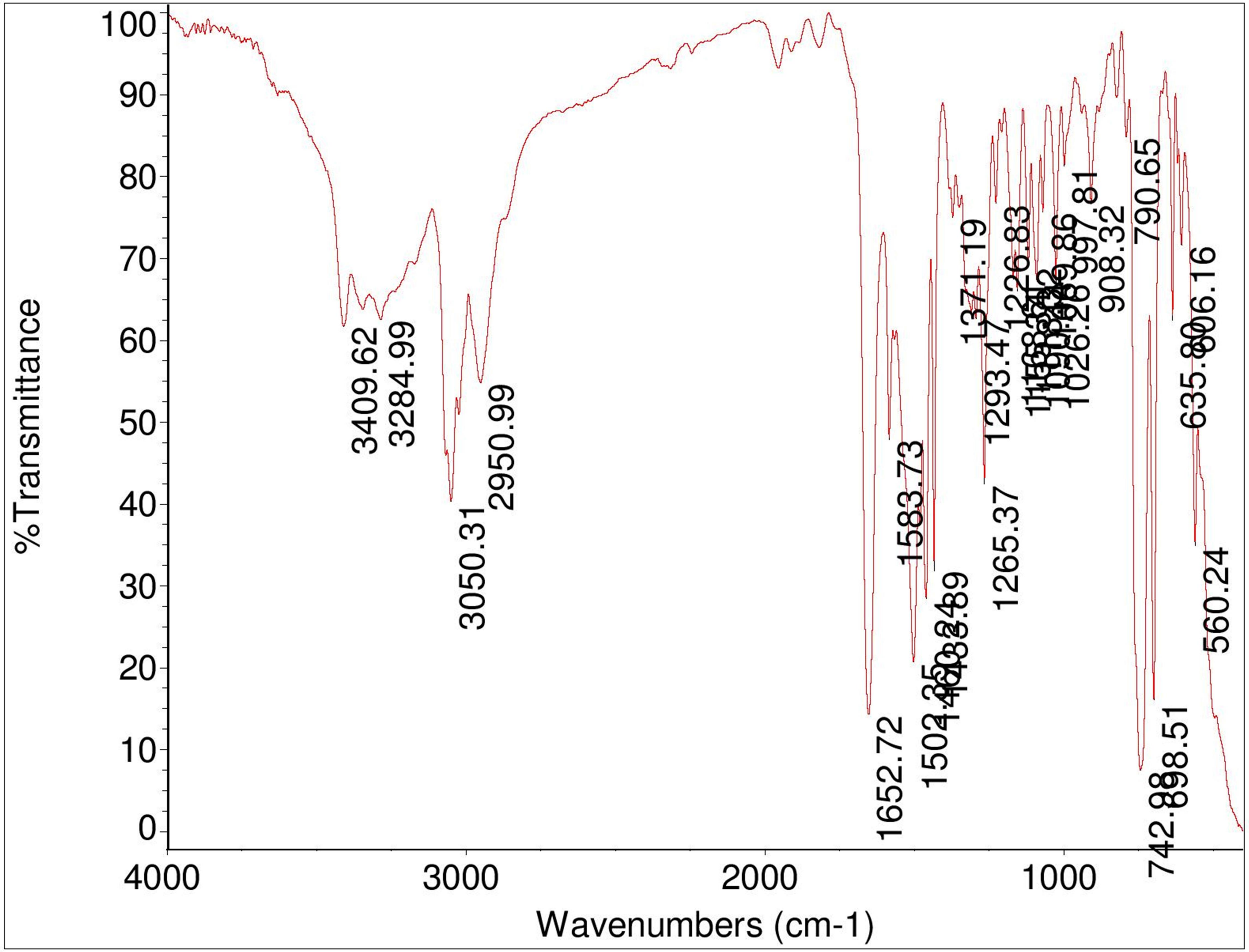
%Transmittance
Wavenumbers (cm-1)
100
90
80
70
60
50
40
30
20
10
0
4000
3000
2000
1000
3409.62
3284.99
3050.31
2950.99
1652.72
1583.73
1265.37
1371.19
1293.47
1226.83
908.32
790.65
606.16
635.80
560.24

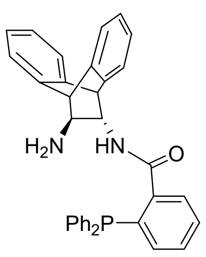
H_2N
HN
O
Ph_2P

(*R*,*R*)-**8**

LRMS [M+Na]$^+$

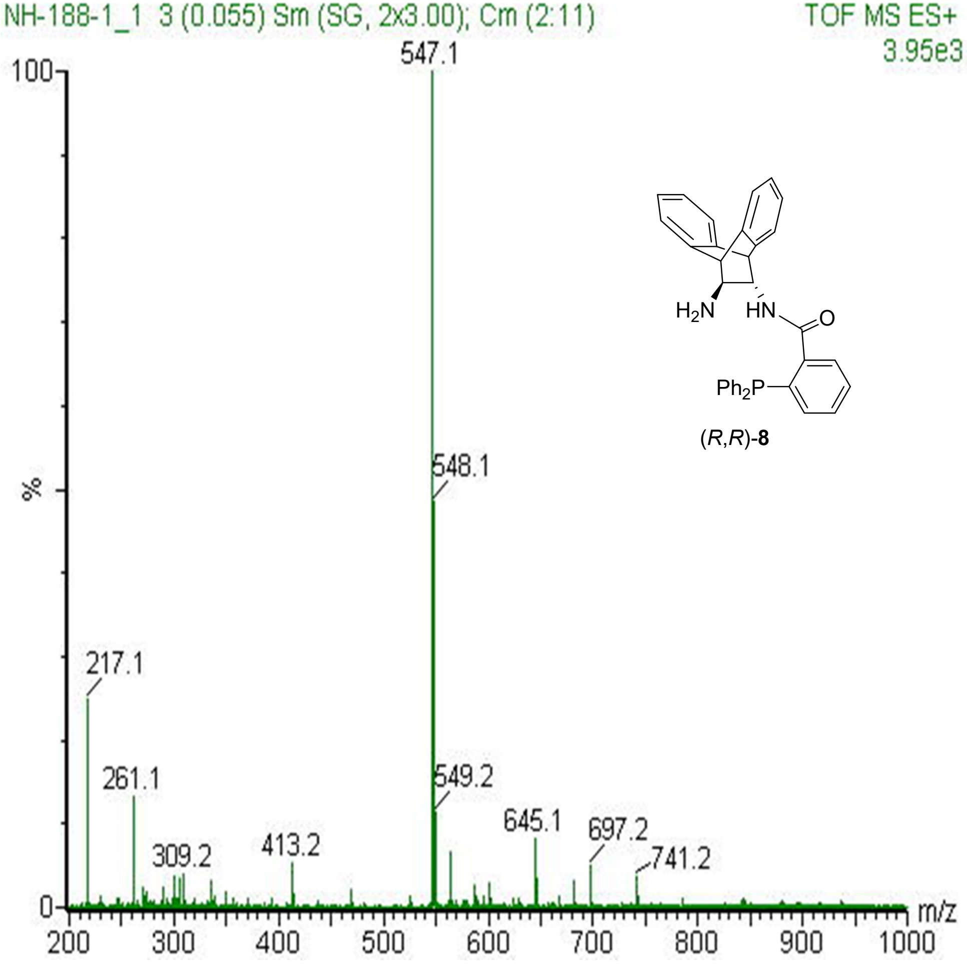

HRMS Observed∆ = 2.4 mDa
Acceptable = ± 2.7 mDa

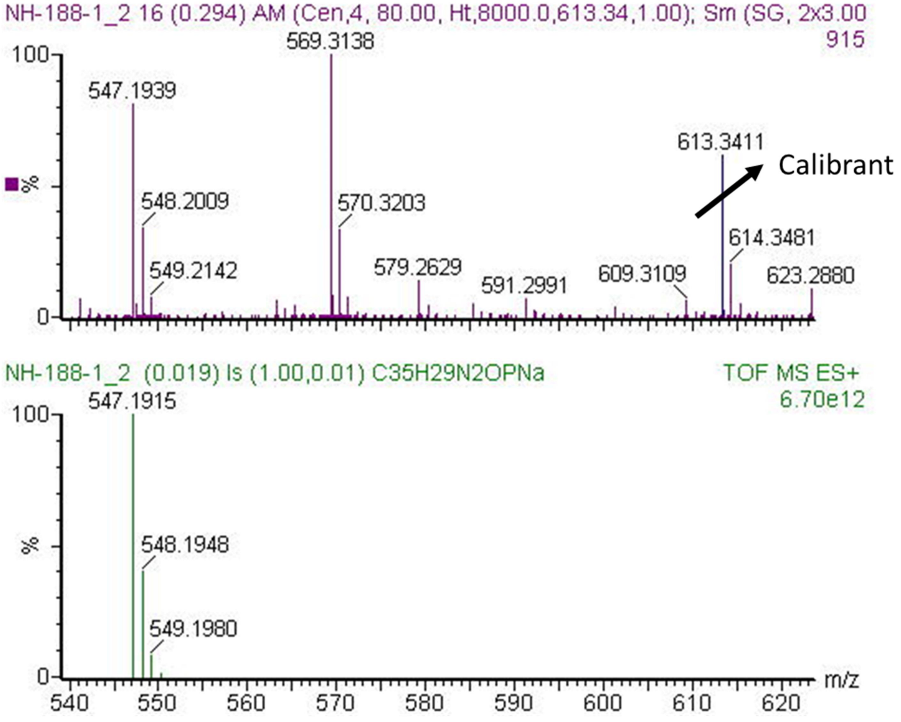

Theoretical =[M+Na]$^+$

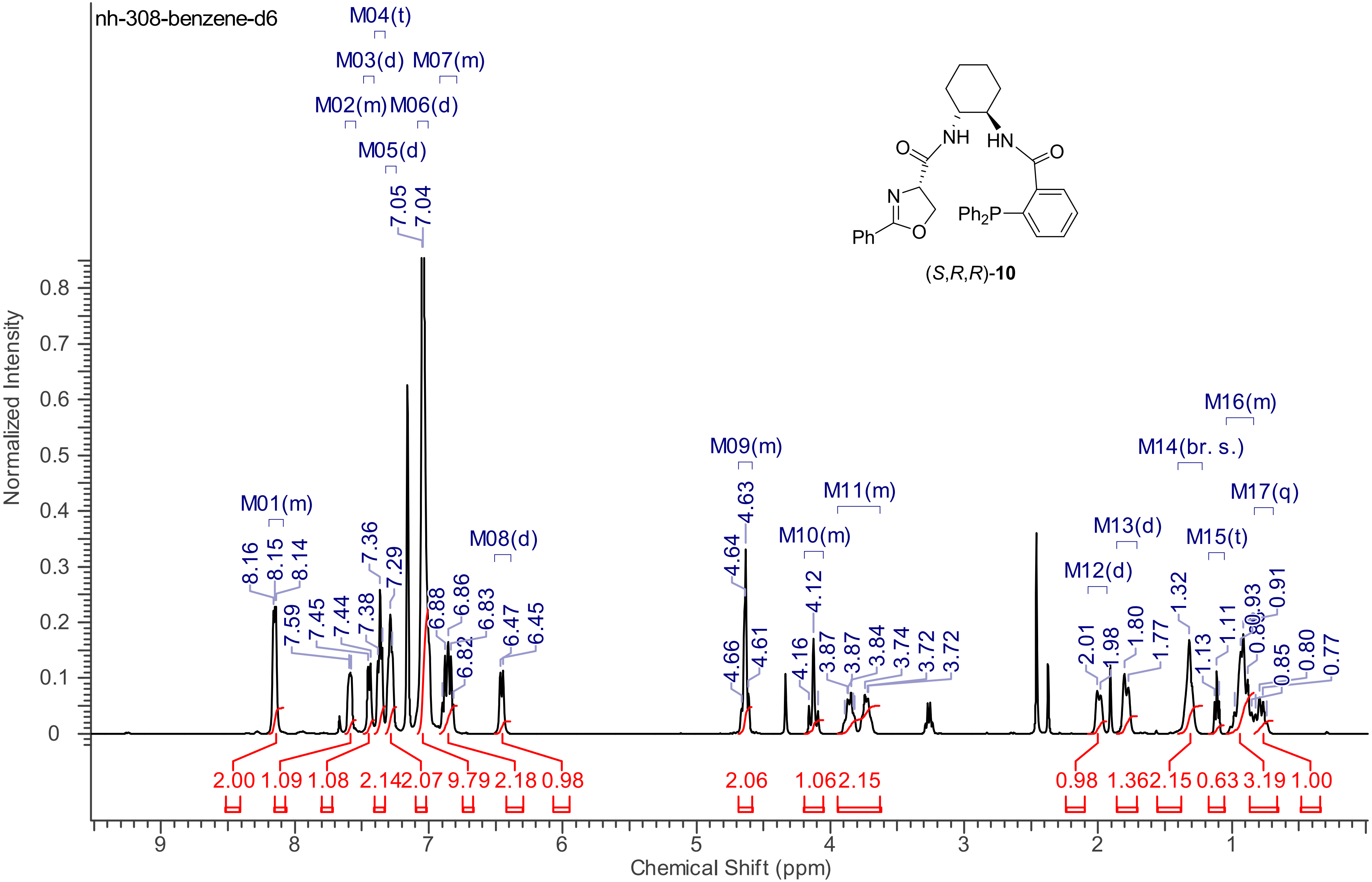
nh-308-benzene-d6
M01(m)
M02(m)
M03(d)
M04(t)
M05(d)
M06(d)
M07(m)
M08(d)
M09(m)
M10(m)
M11(m)
M12(d)
M13(d)
M14(br. s.)
M15(t)
M16(m)
M17(q)
2.00 1.09 1.08 2.14 2.07 9.79 2.18 0.98
2.06
1.06 2.15
0.98 1.36 2.15 0.63 3.19 1.00
Normalized Intensity
Chemical Shift (ppm)
NH
HN
O
N
O
Ph
Ph_2P
(*S*,*R*,*R*)-**10**

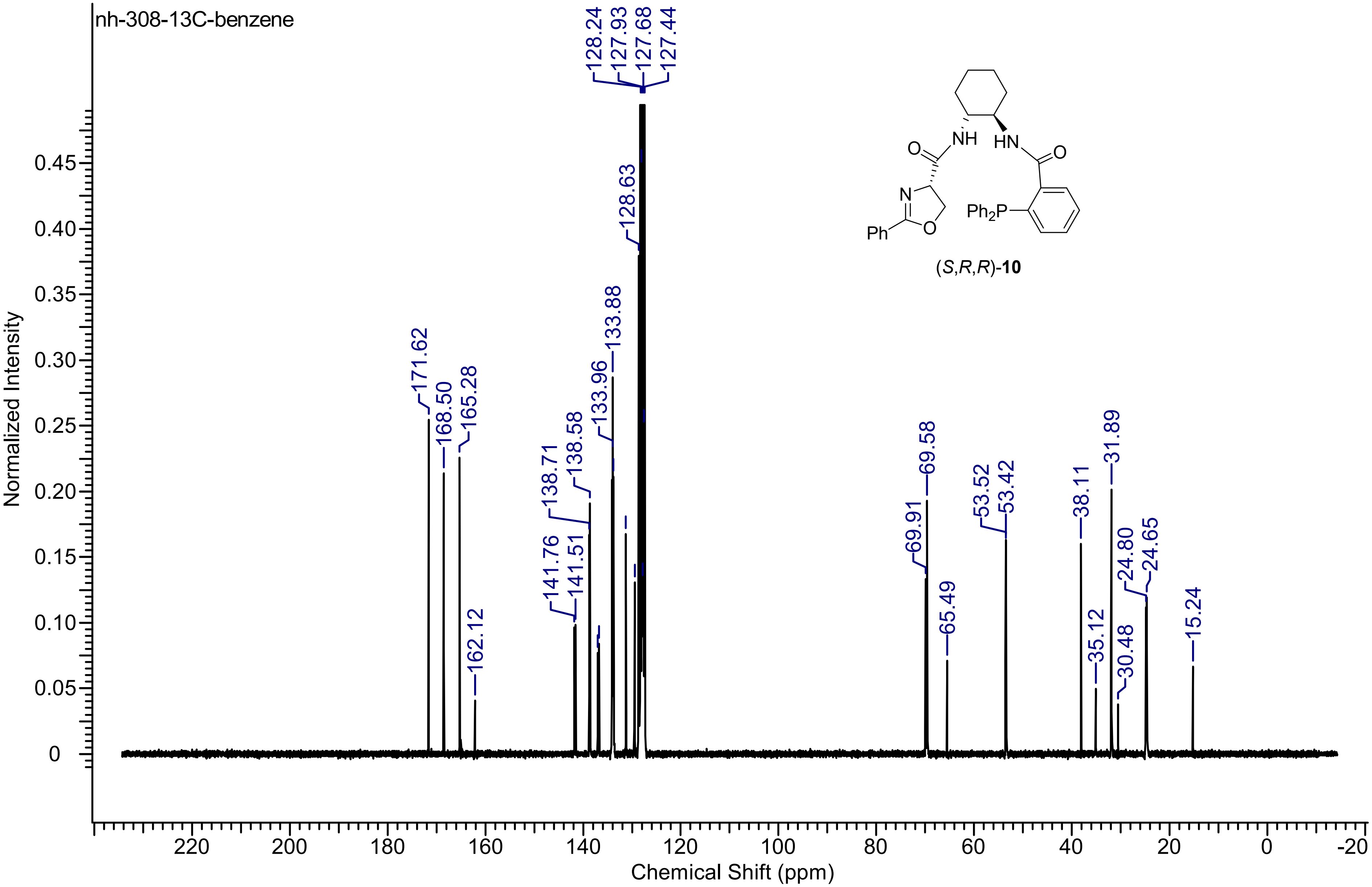
nh-308-13C-benzene
Normalized Intensity
Chemical Shift (ppm)
171.62
168.50
165.28
162.12
141.76
141.51
138.71
138.58
133.96
133.88
128.63
128.24
127.93
127.68
127.44
69.91
69.58
65.49
53.52
53.42
38.11
35.12
31.89
30.48
24.80
24.65
15.24
NH
HN
O
O
N
O
Ph
Ph2P

(*S*,*R*,*R*)-**10**

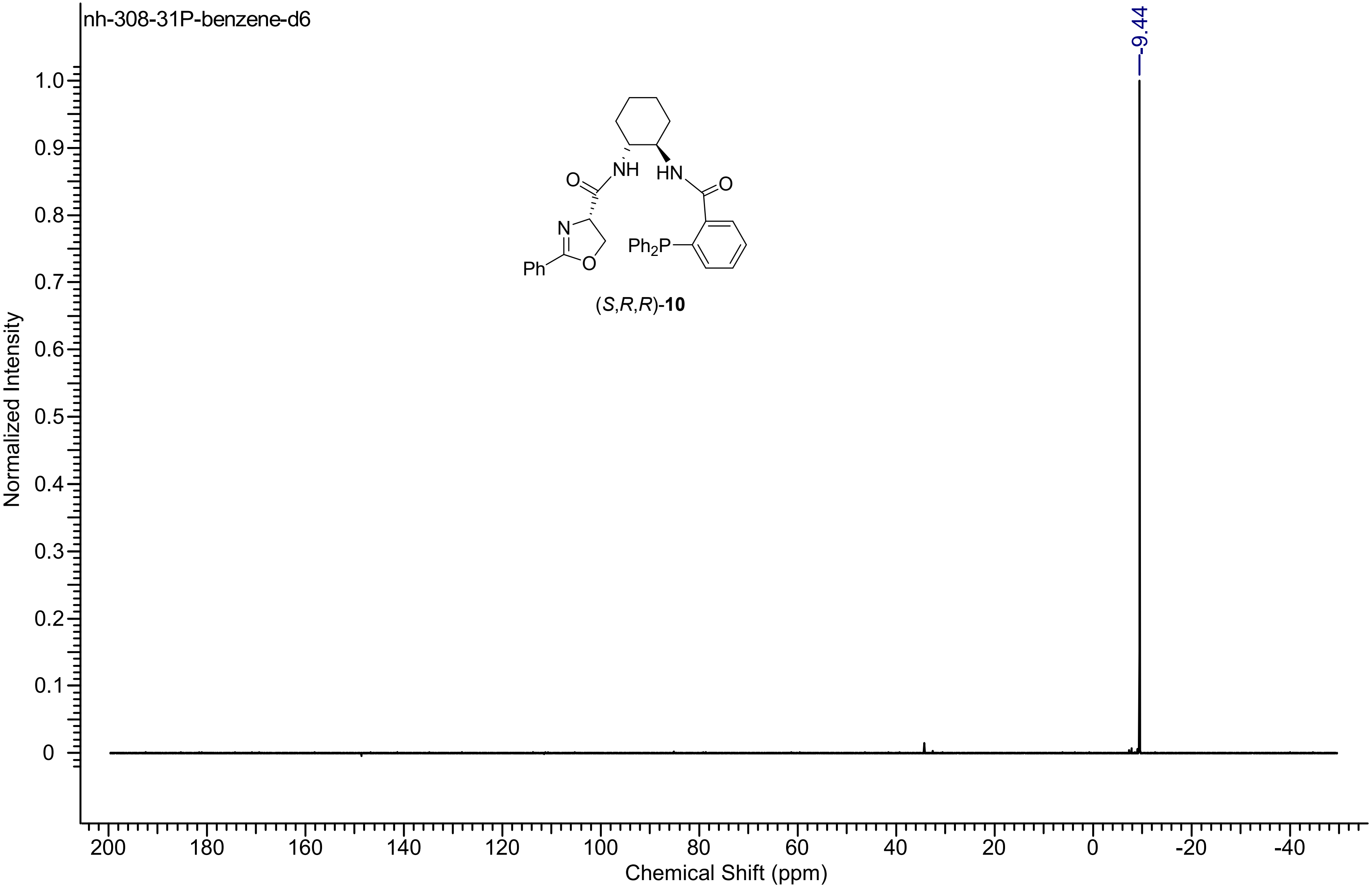
nh-308-31P-benzene-d6
-9.44
Normalized Intensity
Chemical Shift (ppm)
O
NH
HN
O
N
O
Ph
Ph2P
(S,R,R)-10

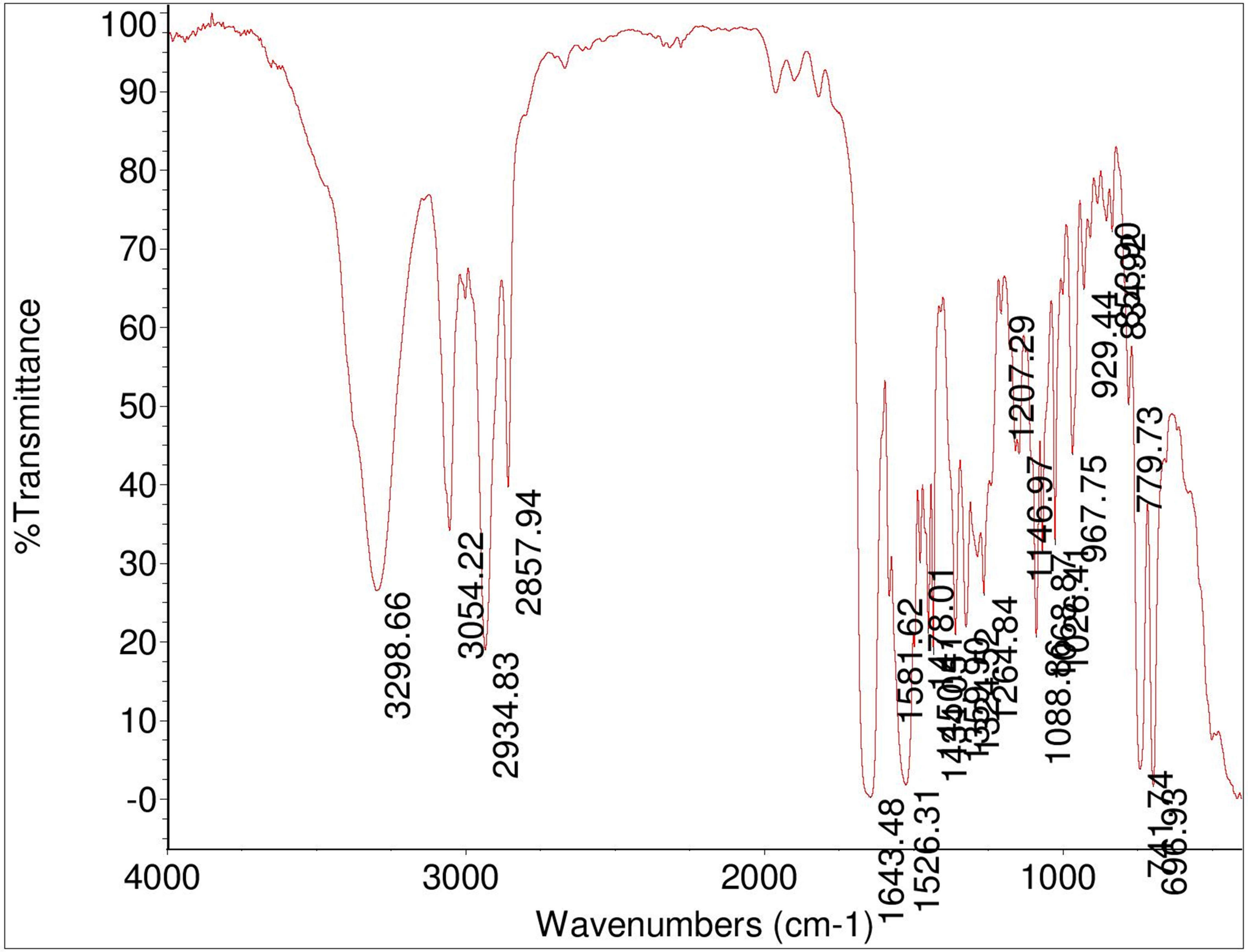
%Transmittance
Wavenumbers (cm-1)
100
90
80
70
60
50
40
30
20
10
-0
4000
3000
2000
1000
3298.66
3054.22
2934.83
2857.94
1643.48
1581.62
1526.31
1478.01
1207.29
1146.97
1088.86
1026.41
967.75
929.44
779.73
741.74
696.93

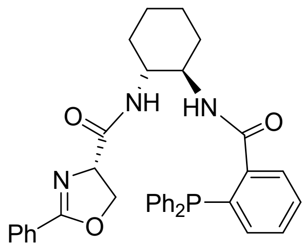
NH
HN
O
O
N
O
Ph
Ph$_2$P

(*S*,*R*,*R*)-**10**

LRMS [M+Na]$^+$

HRMS ObservedΔ = 2.2 mDa
Acceptable = ± 3.0 mDa

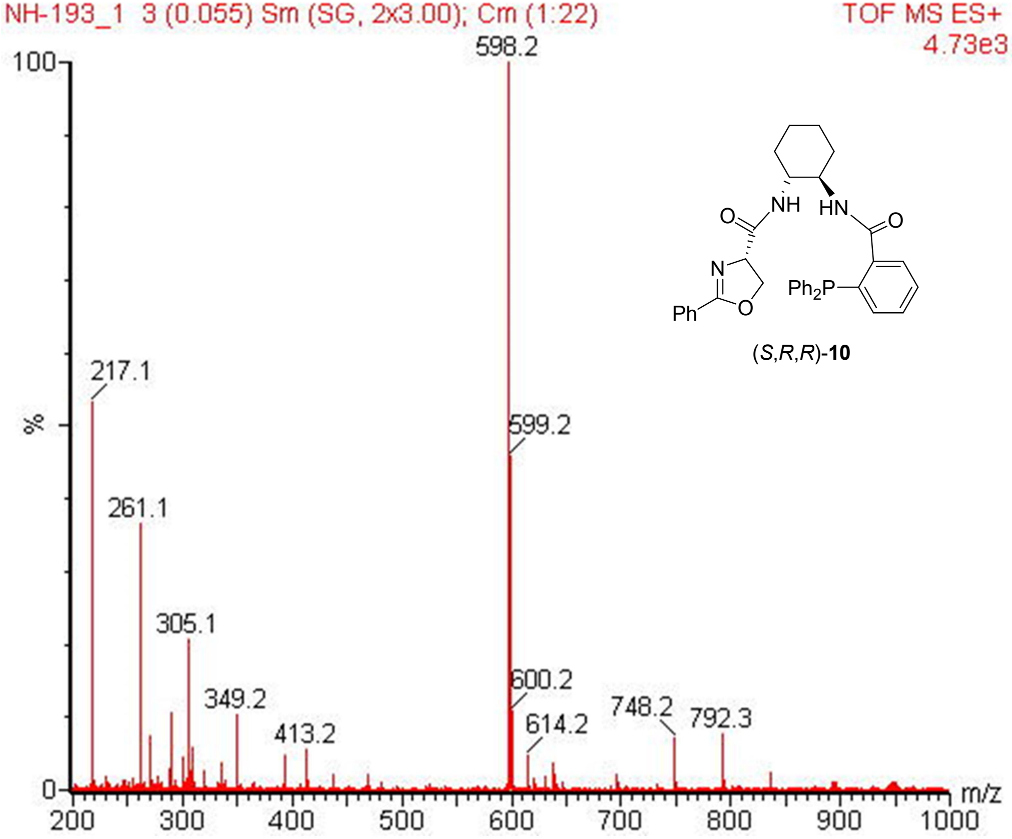

(S,R,R)-**10**

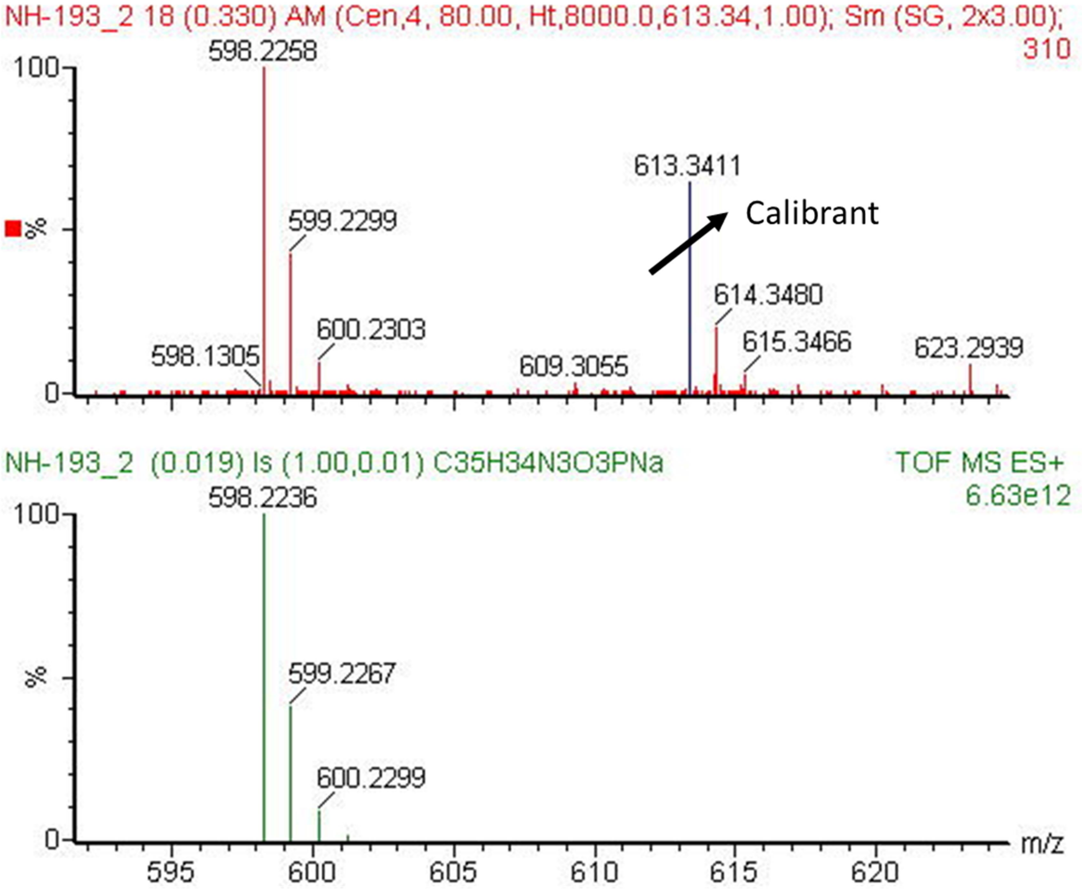

Theoretical =[M+Na]$^+$

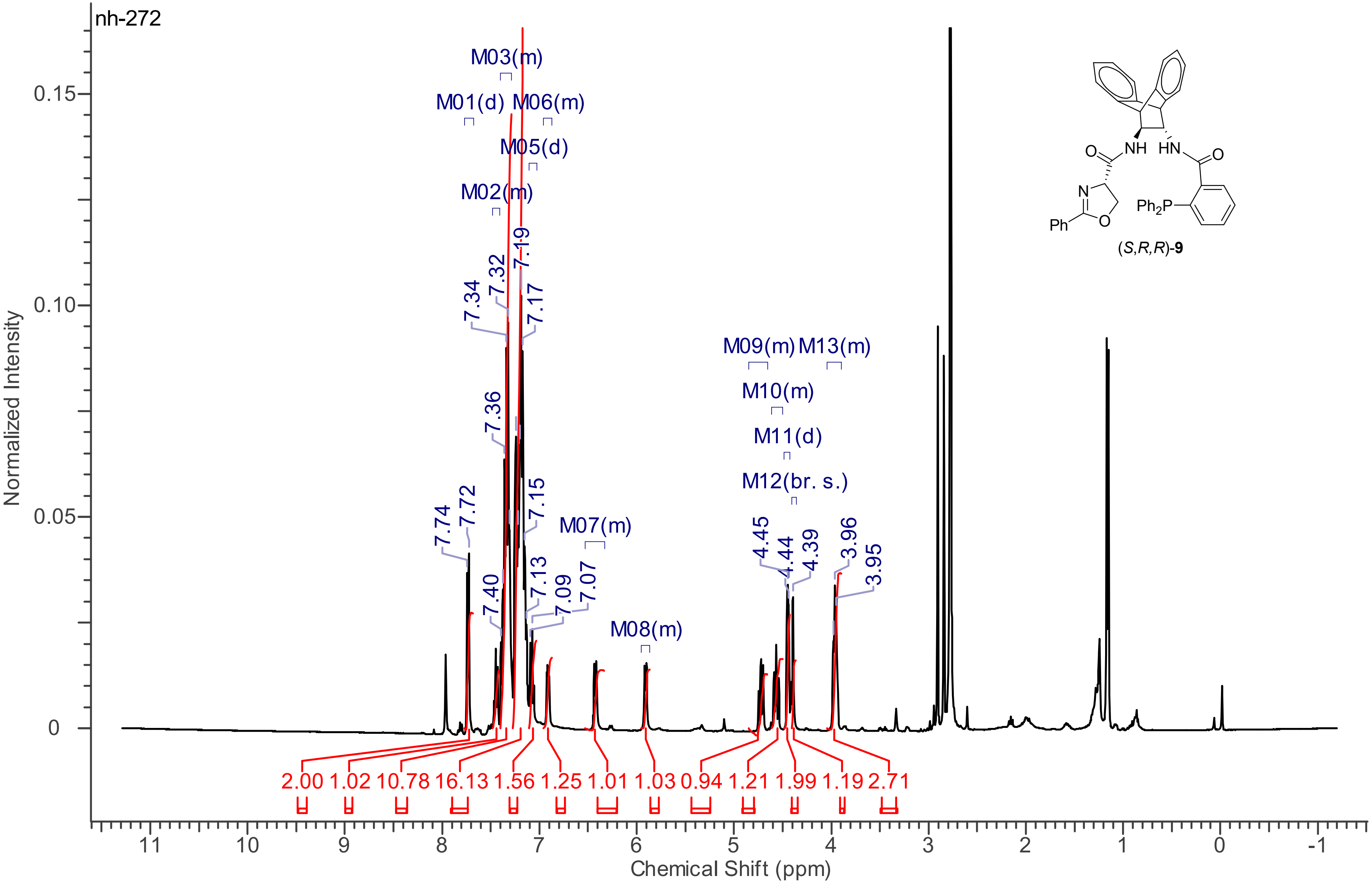

nh-272
M03(m)
M01(d) M06(m)
M05(d)
M02(m)
7.34
7.32
7.19
7.17
7.36
7.74
7.72
7.15
7.40
7.13
7.09
7.07
M07(m)
M08(m)
M09(m) M13(m)
M10(m)
M11(d)
M12(br. s.)
4.45
4.44
4.39
3.96
3.95
2.00 1.02 10.78 16.13 1.56 1.25 1.01 1.03 0.94 1.21 1.99 1.19 2.71
Normalized Intensity
0.15
0.10
0.05
0
11 10 9 8 7 6 5 4 3 2 1 0 -1
Chemical Shift (ppm)
O
NH
HN
O
N
O
Ph
Ph_2P
(*S*,*R*,*R*)-**9**

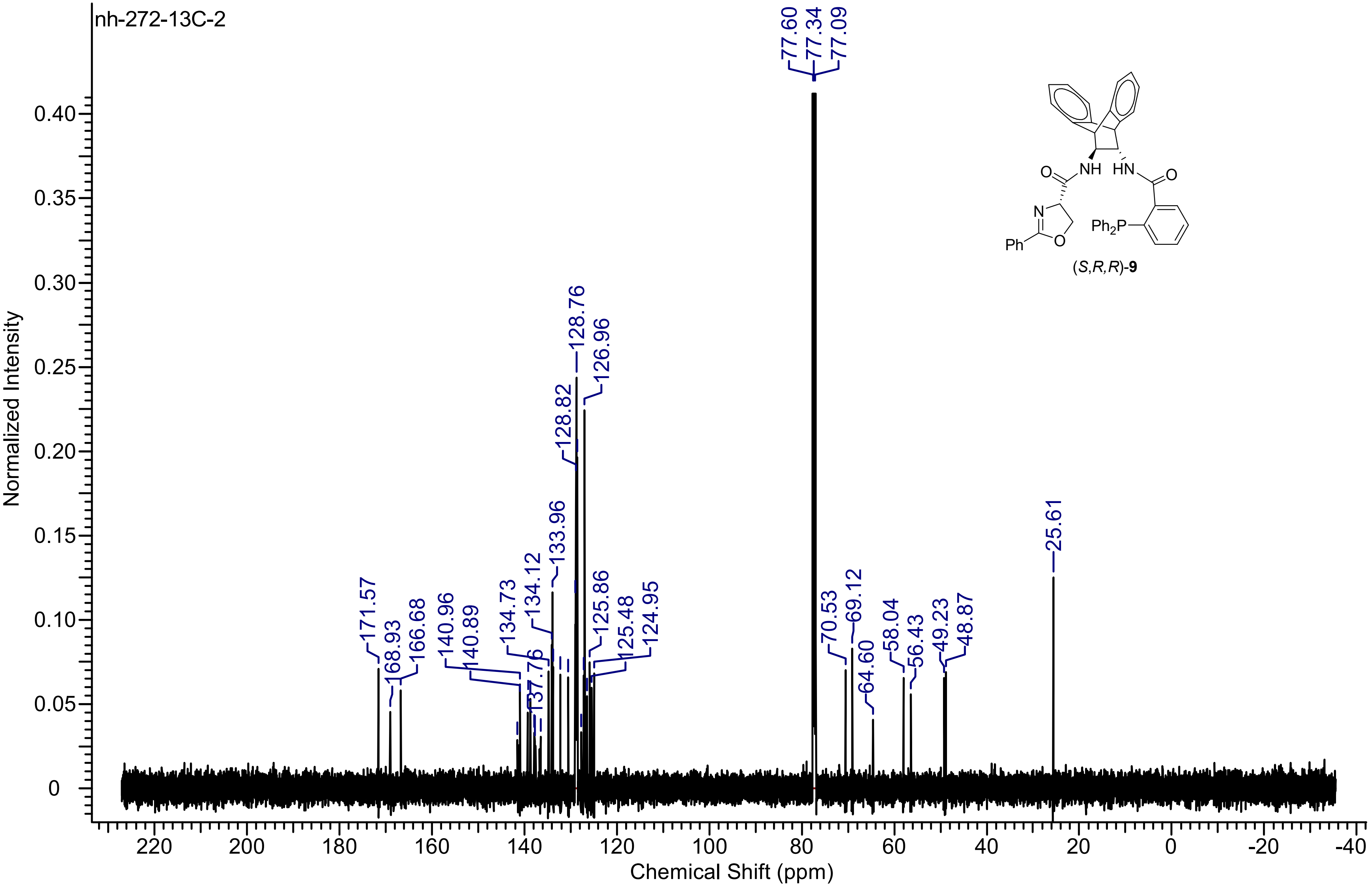
nh-272-13C-2
77.60
77.34
77.09
128.76
126.96
128.82
133.96
134.12
134.73
140.96
140.89
137.76
171.57
168.93
166.68
125.86
125.48
124.95
70.53
69.12
64.60
58.04
56.43
49.23
48.87
25.61
Normalized Intensity
Chemical Shift (ppm)
NH
HN
O
O
N
O
Ph
Ph_2P
(S,R,R)-9

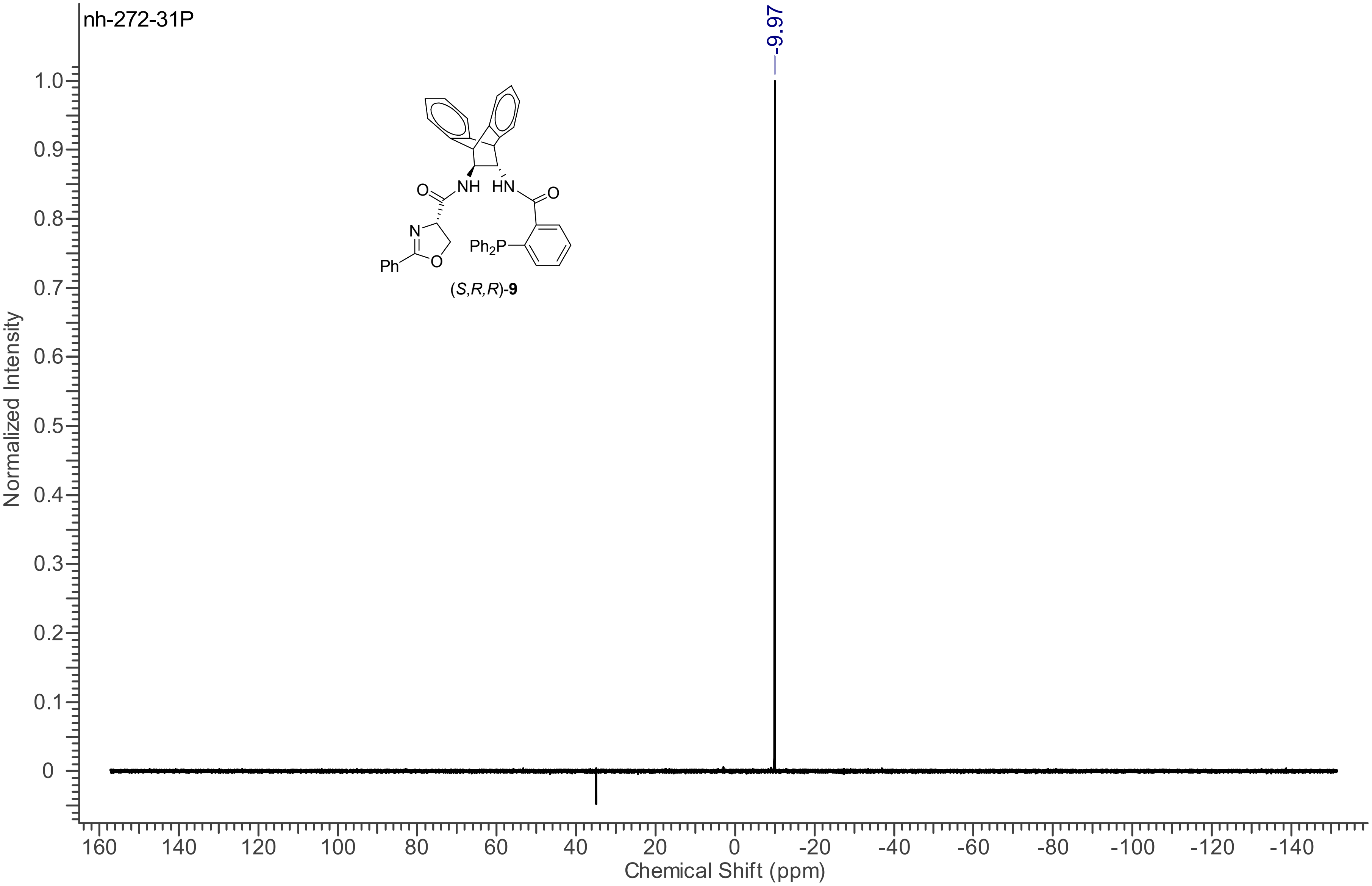
nh-272-31P
-9.97
NH
HN
O
O
N
O
Ph
Ph2P
(S,R,R)-9
Normalized Intensity
Chemical Shift (ppm)

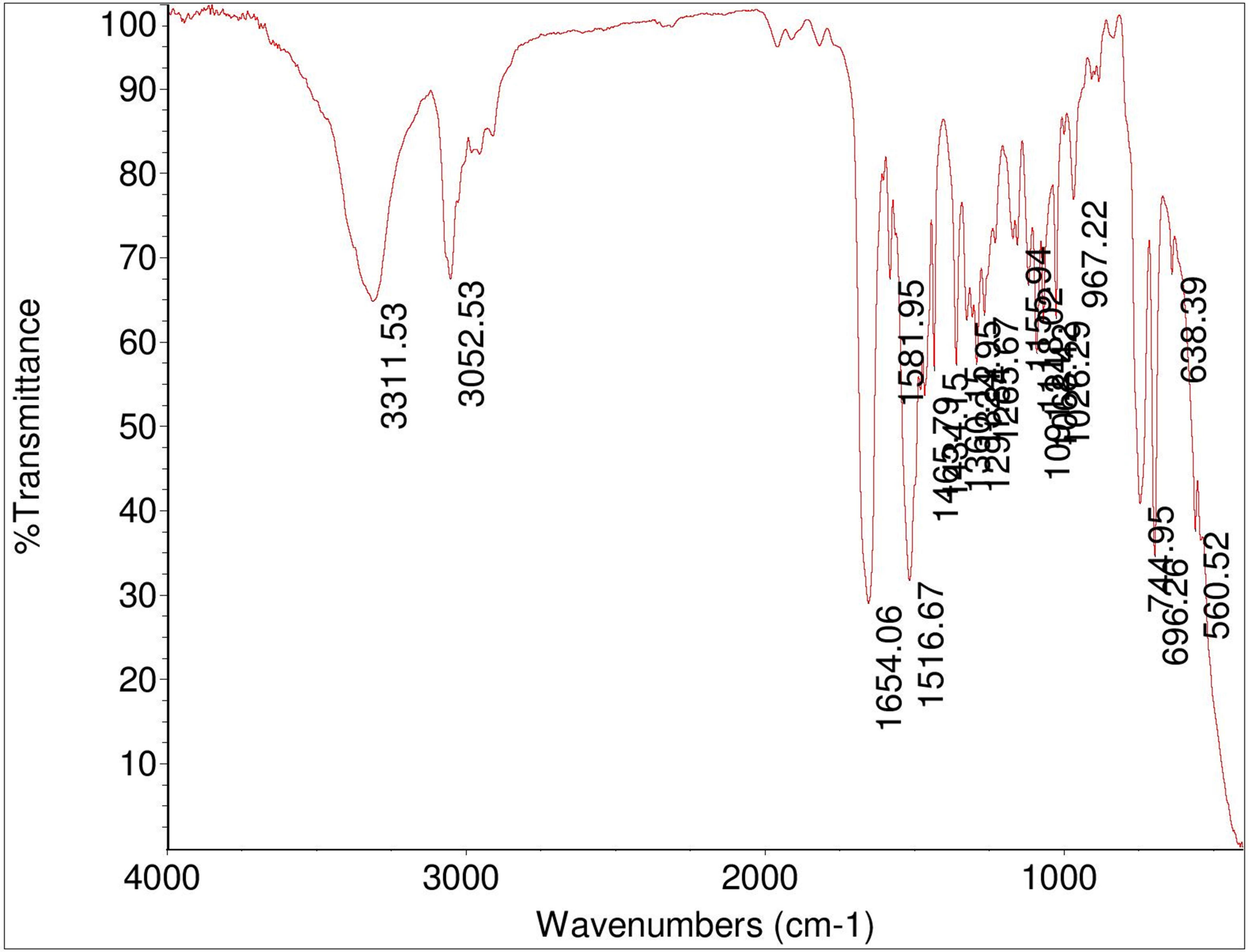
%Transmittance
Wavenumbers (cm-1)
100
90
80
70
60
50
40
30
20
10
4000
3000
2000
1000
3311.53
3052.53
1654.06
1516.67
1581.95
1265.67
1026.29
967.22
744.95
696.26
638.39
560.52

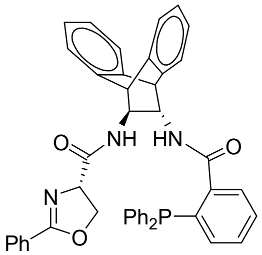
O
NH
HN
O
N
Ph
O
Ph_2P

(*S*,*R*,*R*)-**9**

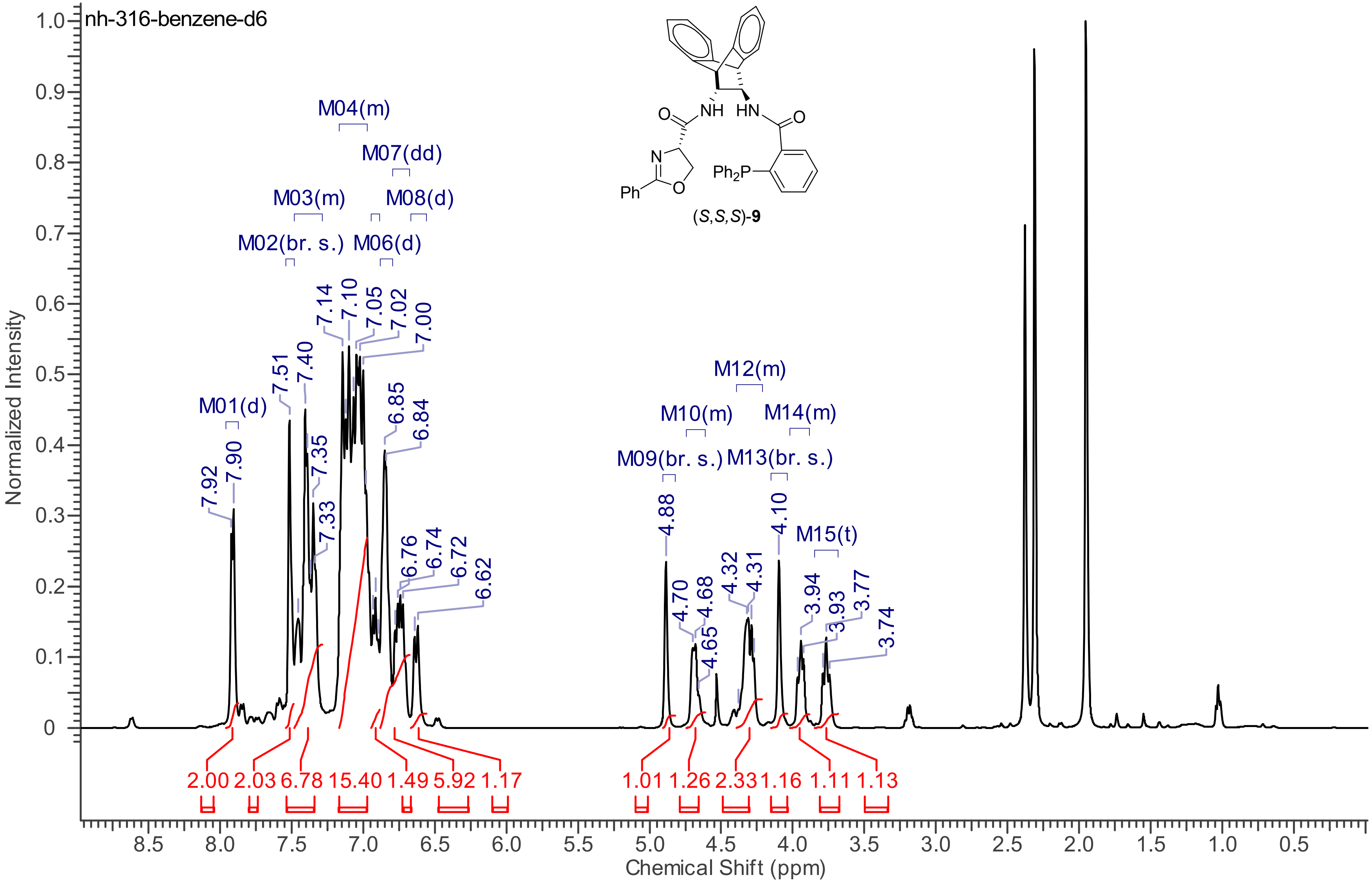

nh-316-benzene-d6
(S,S,S)-9
NH
HN
O
O
N
O
Ph
Ph_2P
M01(d)
M02(br. s.)
M03(m)
M04(m)
M06(d)
M07(dd)
M08(d)
M09(br. s.)
M10(m)
M12(m)
M13(br. s.)
M14(m)
M15(t)
7.92
7.90
7.51
7.40
7.35
7.33
7.14
7.10
7.05
7.02
7.00
6.85
6.84
6.76
6.74
6.72
6.62
4.88
4.70
4.68
4.65
4.32
4.31
4.10
3.94
3.93
3.77
3.74
2.00 2.03 6.78 15.40 1.49 5.92 1.17
1.01 1.26 2.33 1.16 1.11 1.13
Normalized Intensity
Chemical Shift (ppm)

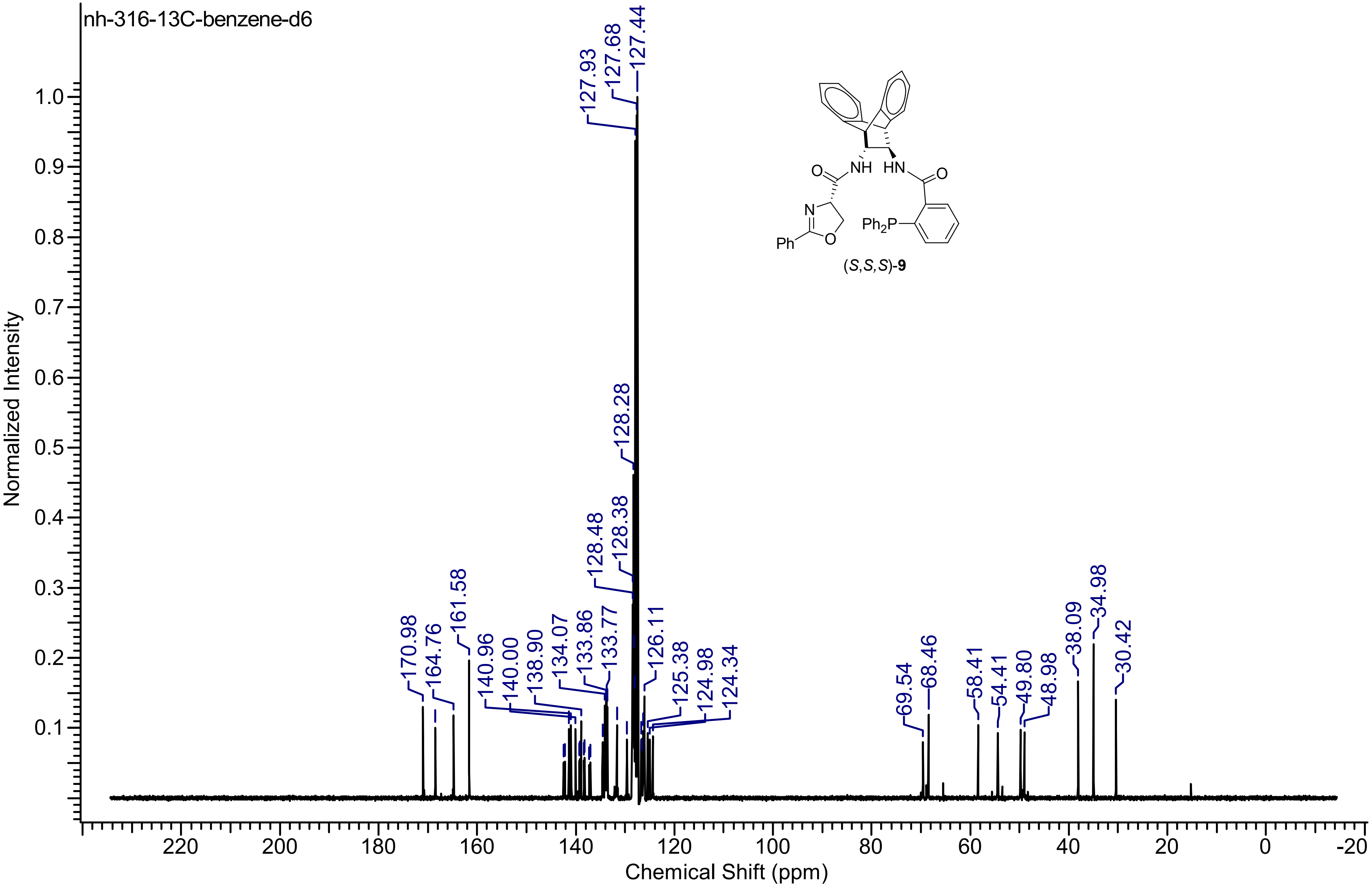
nh-316-13C-benzene-d6
Normalized Intensity
Chemical Shift (ppm)
170.98
164.76
161.58
140.96
140.00
138.90
134.07
133.86
133.77
128.48
128.38
128.28
127.93
127.68
127.44
126.11
125.38
124.98
124.34
69.54
68.46
58.41
54.41
49.80
48.98
38.09
34.98
30.42
(S,S,S)-9

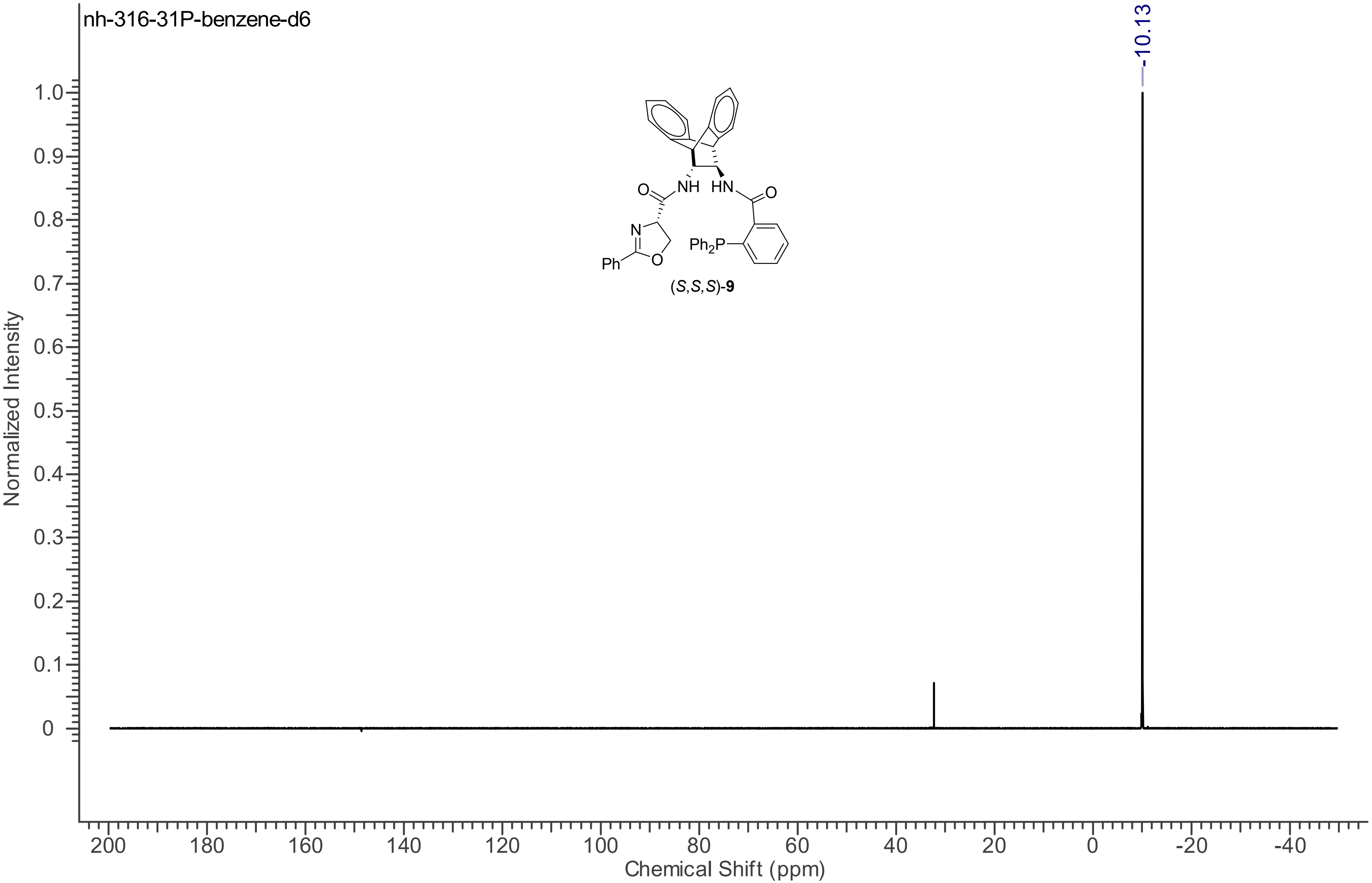

nh-316-31P-benzene-d6
-10.13
NH
HN
O
O
N
O
Ph
Ph2P
(S,S,S)-9
Normalized Intensity
1.0
0.9
0.8
0.7
0.6
0.5
0.4
0.3
0.2
0.1
0
200
180
160
140
120
100
80
60
40
20
0
-20
-40
Chemical Shift (ppm)

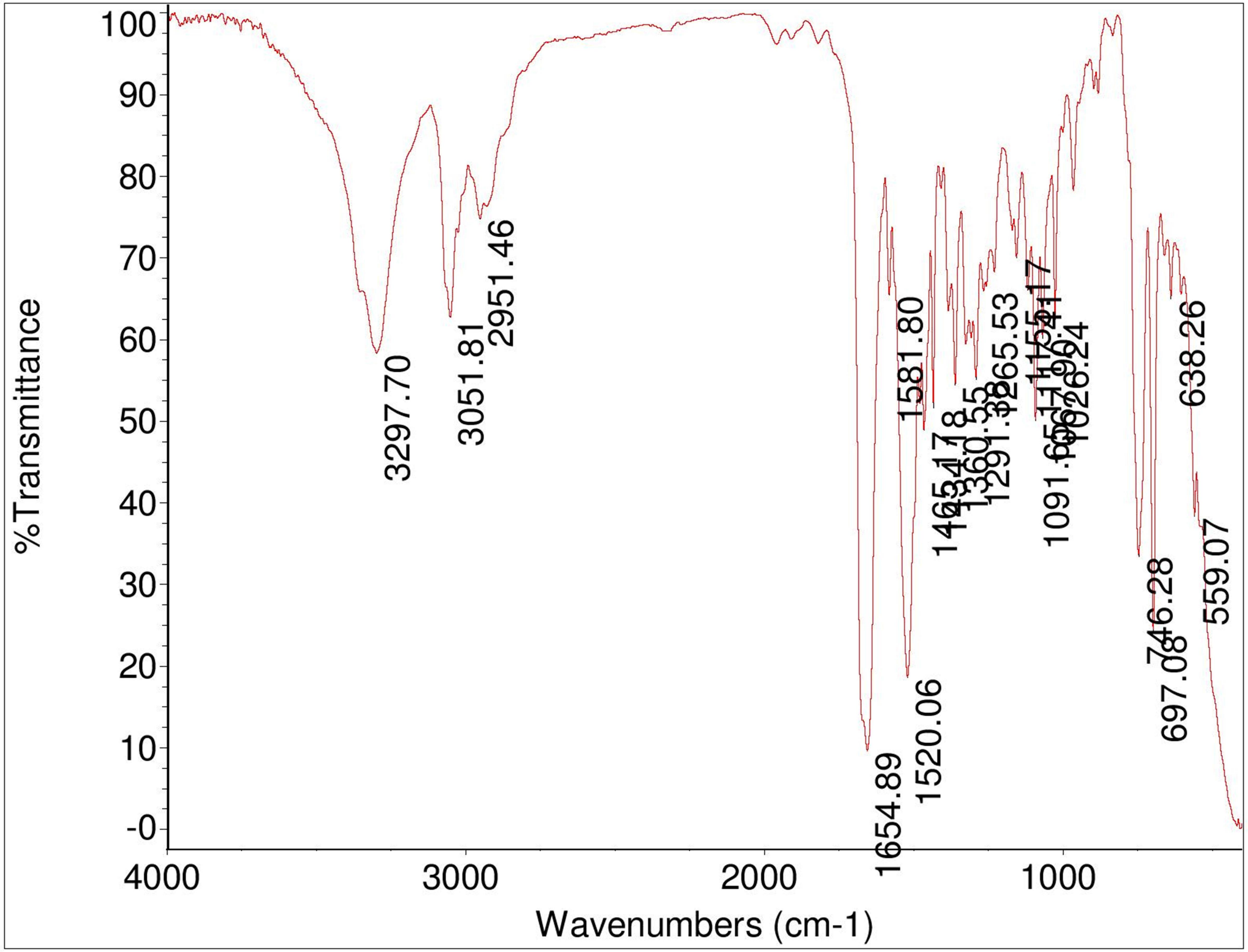
%Transmittance
Wavenumbers (cm-1)
3297.70
3051.81
2951.46
1654.89
1520.06
1581.80
1265.53
1026.24
638.26
746.28
697.08
559.07

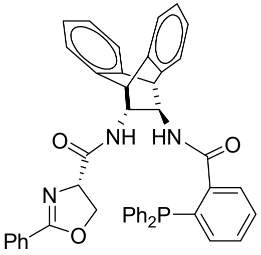
O
NH
HN
O
N
Ph
O
Ph$_2$P
(S,S,S)-**9**

ObservedΔ = 0.8 mDa
Acceptable = ± 3.0 mDa

LRMS [M+Na]$^+$

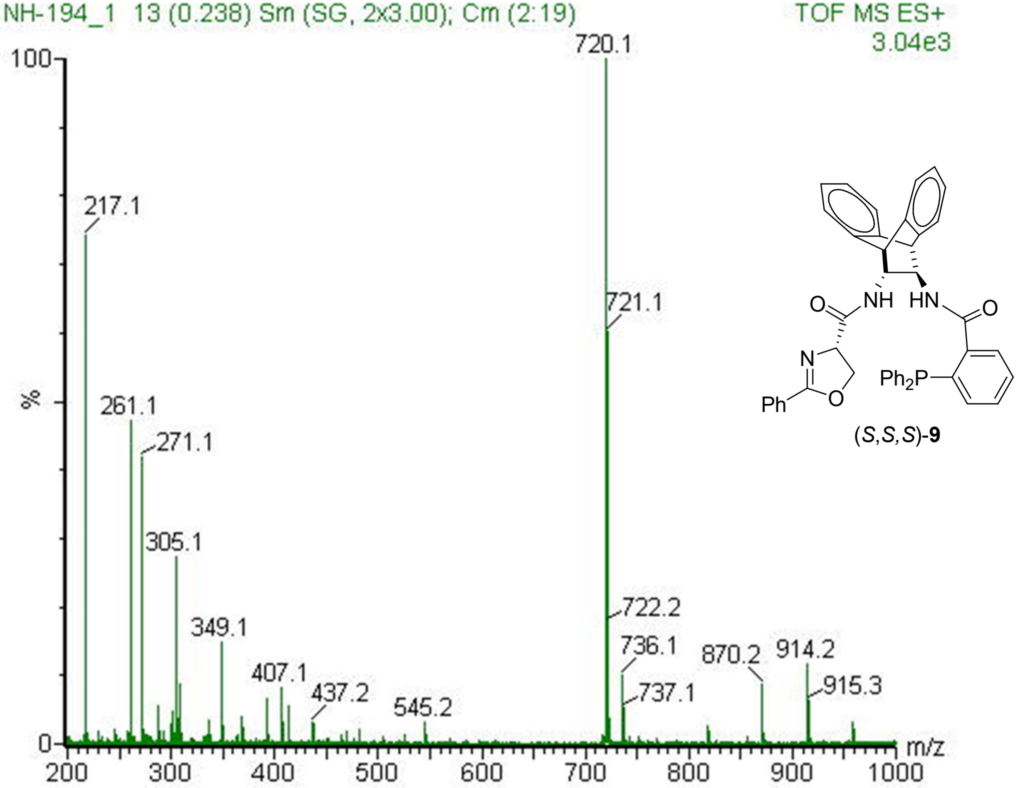

(*S*,*S*,*S*)-**9**

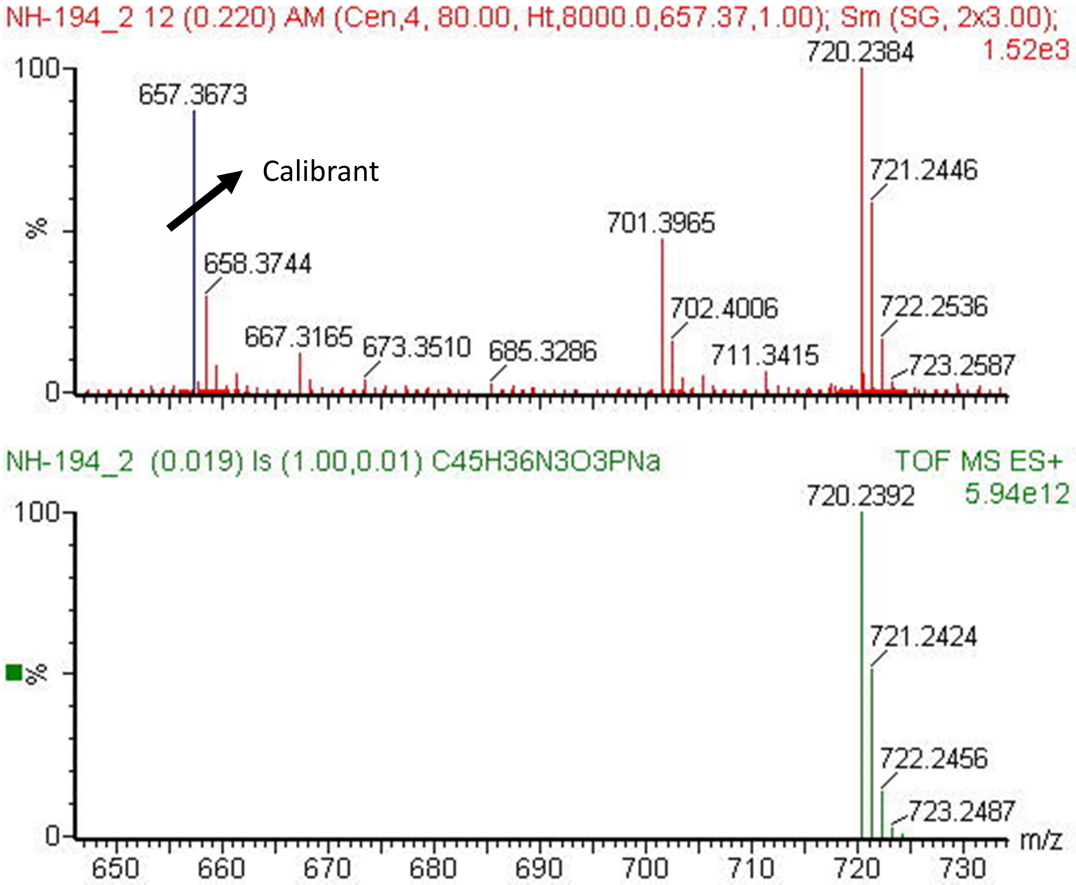

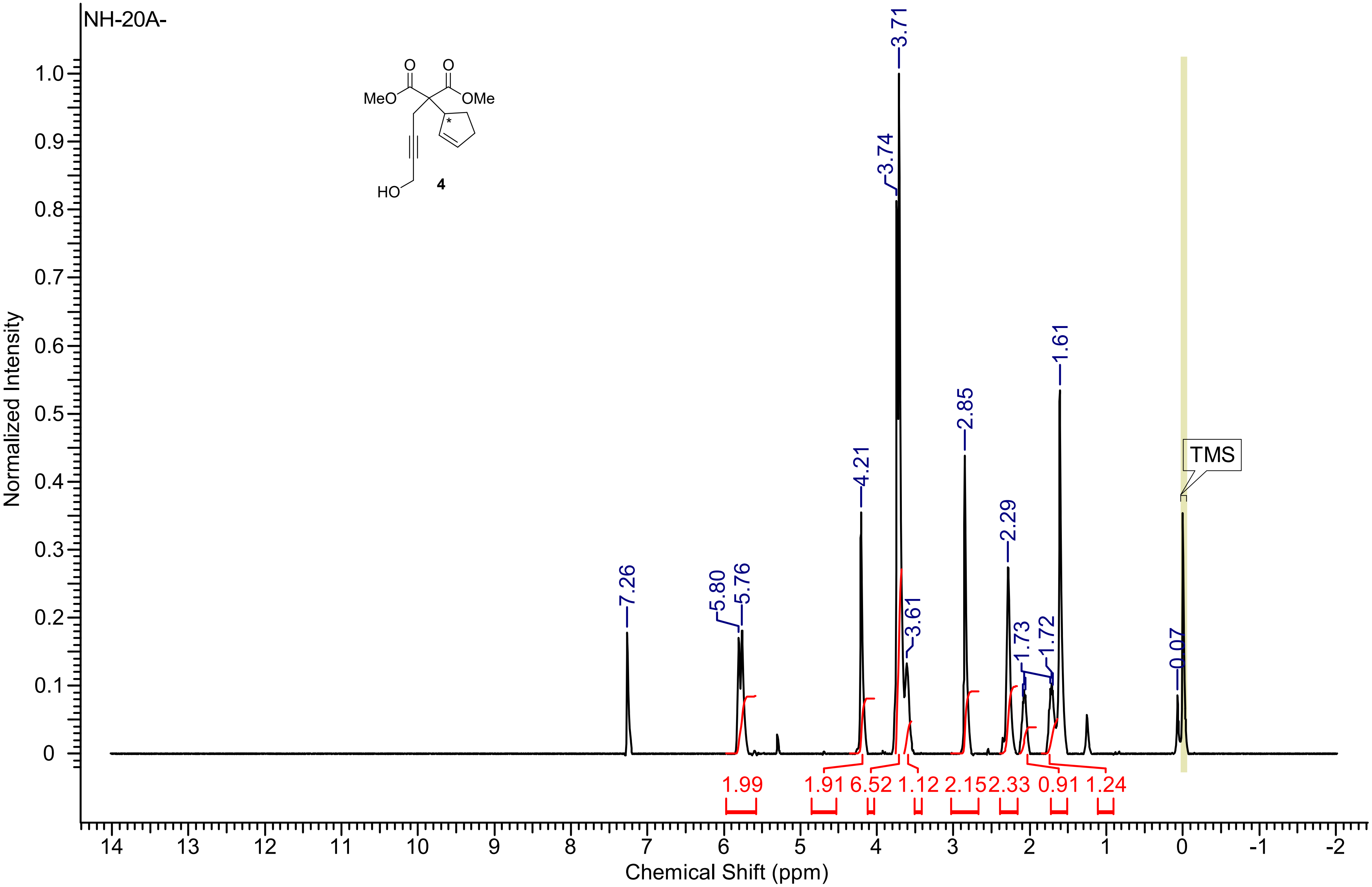

NH-20A-
MeO
OMe
O
O
HO
4
Normalized Intensity
Chemical Shift (ppm)
7.26
5.80
5.76
4.21
3.74
3.71
3.61
2.85
2.29
1.73
1.72
1.61
0.07
TMS
1.99
1.91
6.52
1.12
2.15
2.33
0.91
1.24

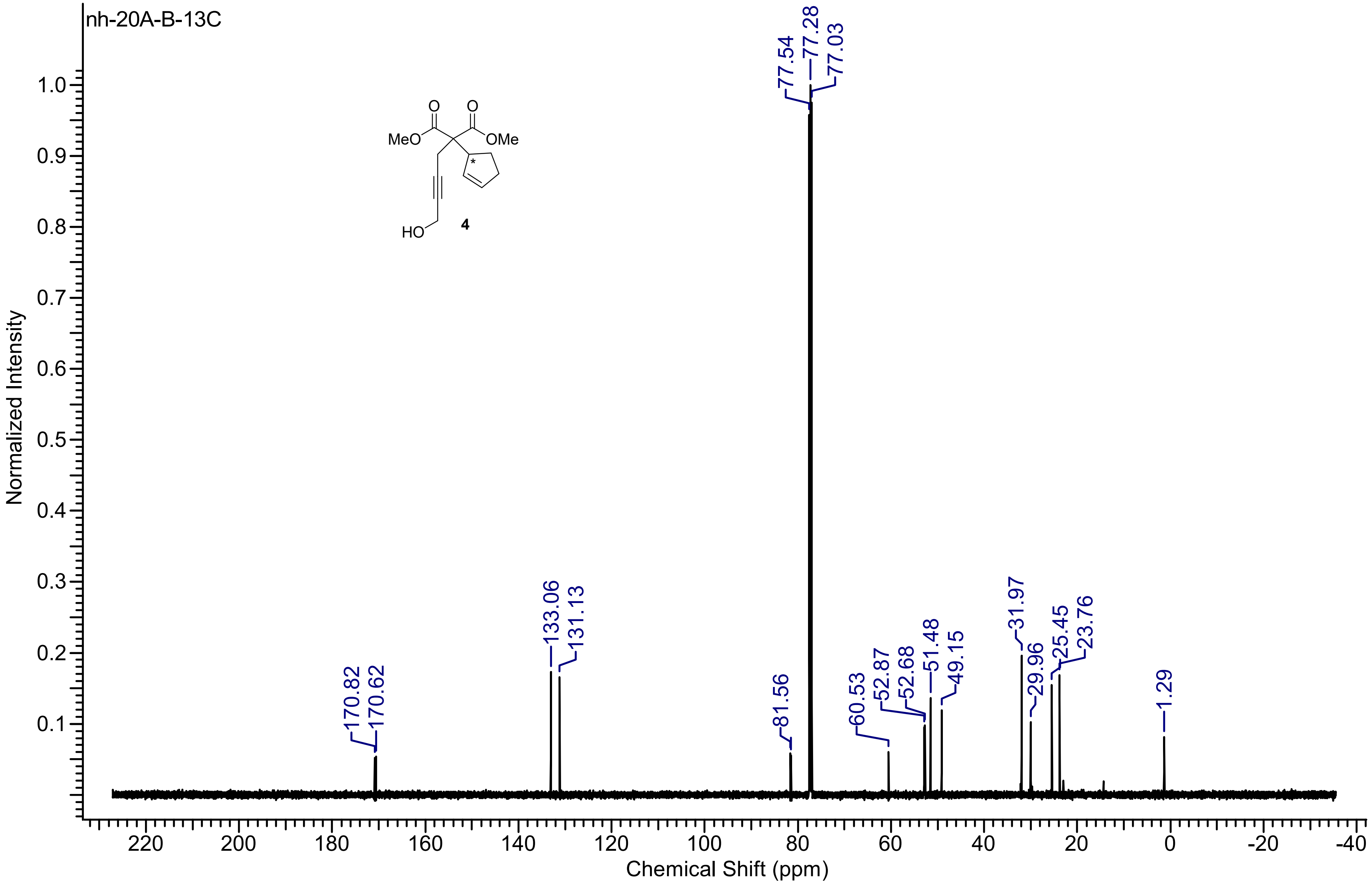
nh-20A-B-13C
MeO
O
O
OMe
*
HO
4
77.54
77.28
77.03
170.82
170.62
133.06
131.13
81.56
60.53
52.87
52.68
51.48
49.15
31.97
29.96
25.45
23.76
1.29
Normalized Intensity
Chemical Shift (ppm)

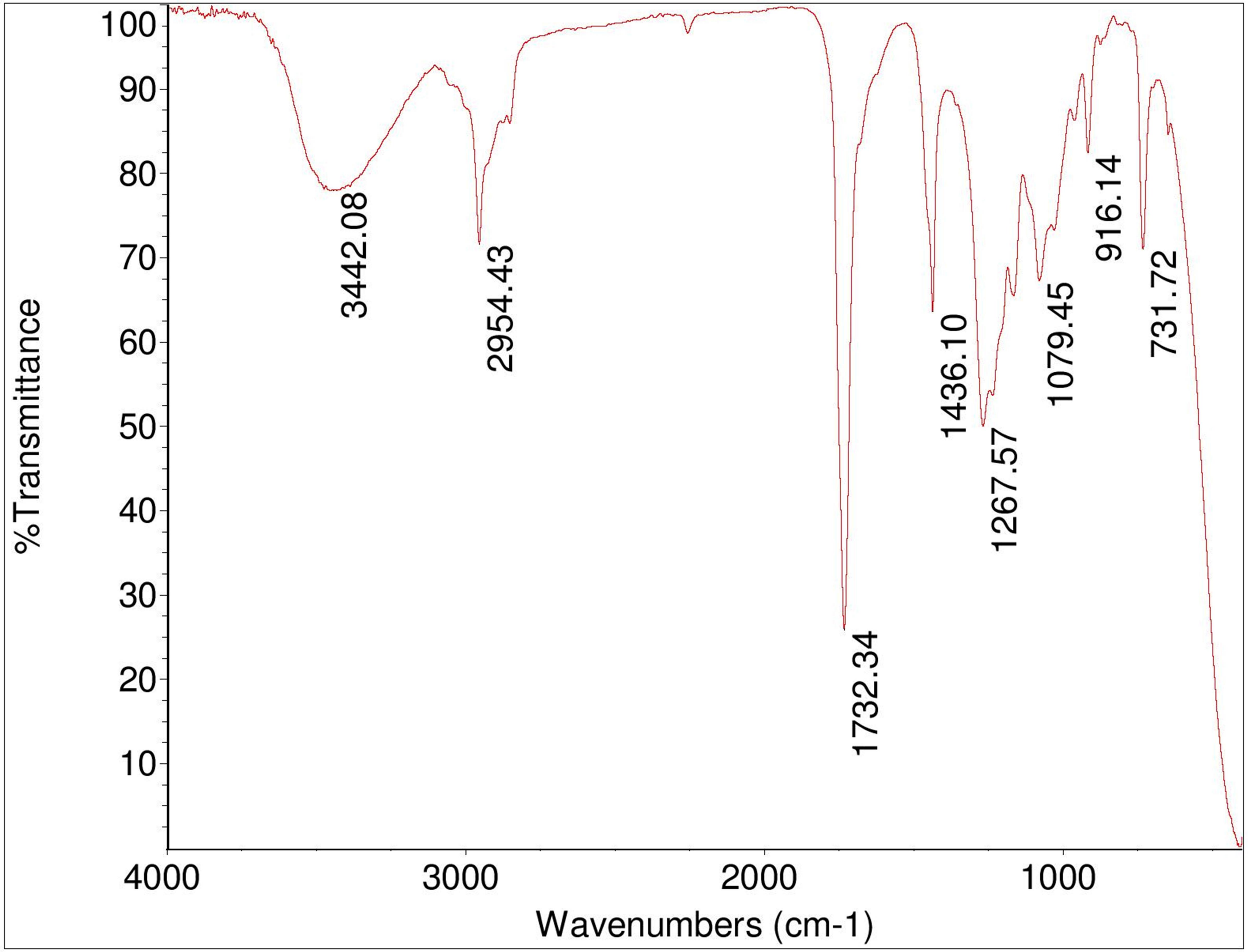
%Transmittance
Wavenumbers (cm-1)
100
90
80
70
60
50
40
30
20
10
4000
3000
2000
1000
3442.08
2954.43
1732.34
1436.10
1267.57
1079.45
916.14
731.72

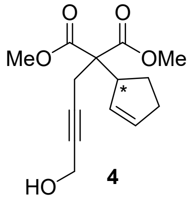
MeO
O
O
OMe
*
HO

4

LRMS [M+Na]+ [2M+Na]+

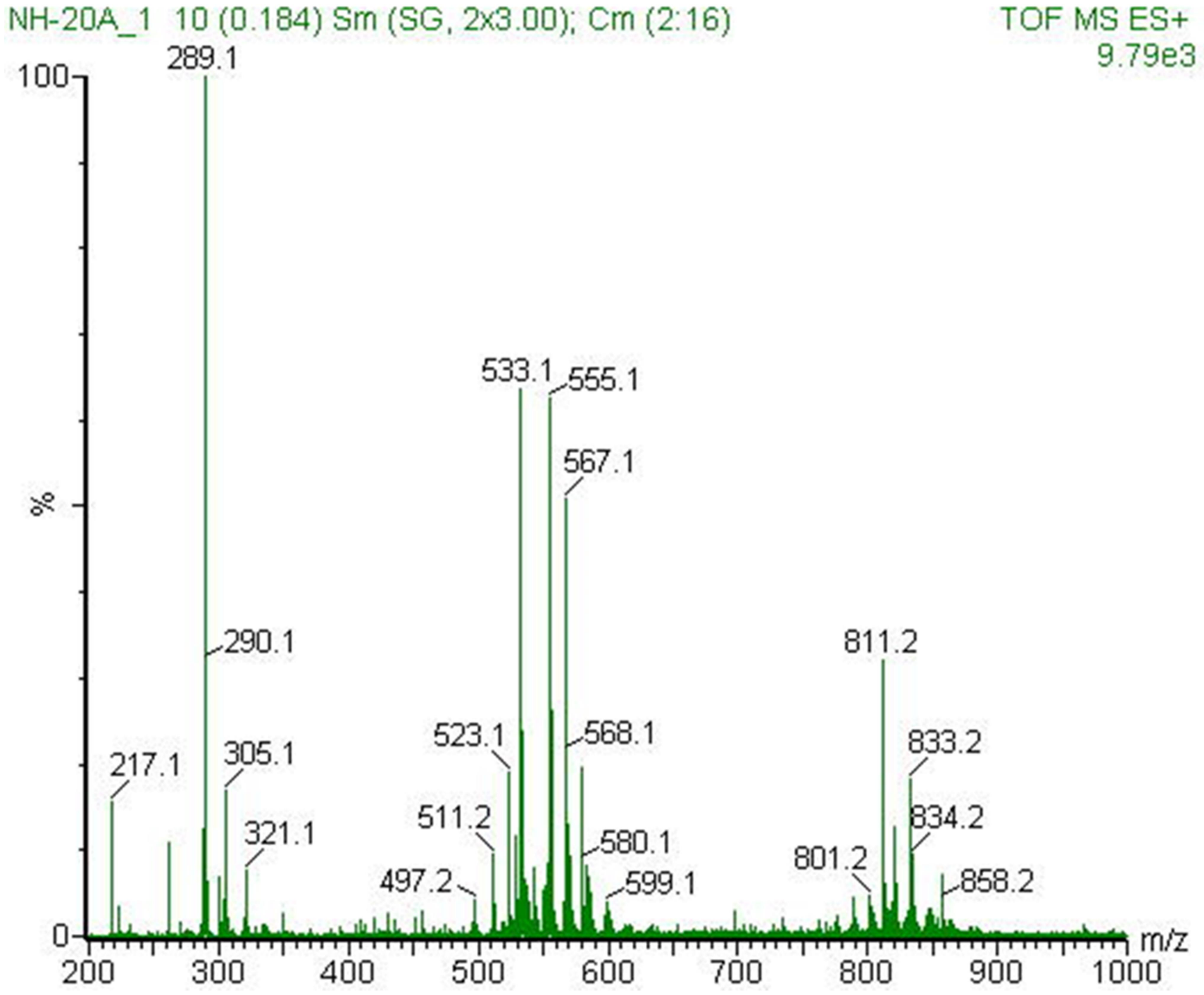

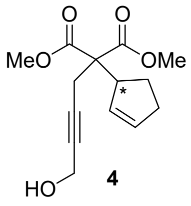

4

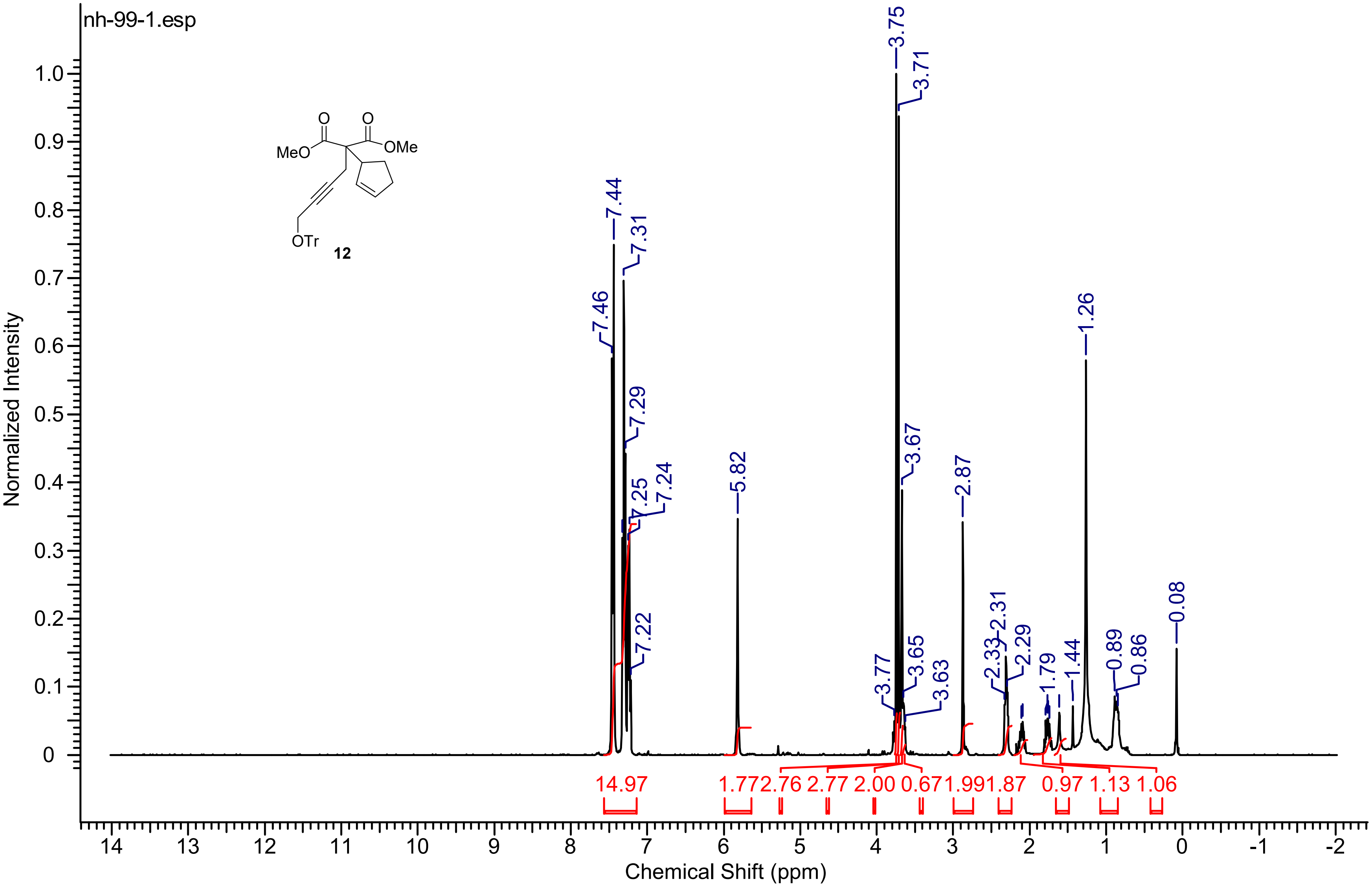
nh-99-1.esp
MeO
O
O
OMe
OTr
12
7.46
7.44
7.31
7.29
7.25
7.24
7.22
5.82
3.75
3.71
3.67
3.65
3.63
3.77
2.87
2.33
2.31
2.29
1.79
1.44
1.26
0.89
0.86
0.08
14.97
1.77
2.76
2.77
2.00
0.67
1.99
1.87
0.97
1.13
1.06
Normalized Intensity
Chemical Shift (ppm)

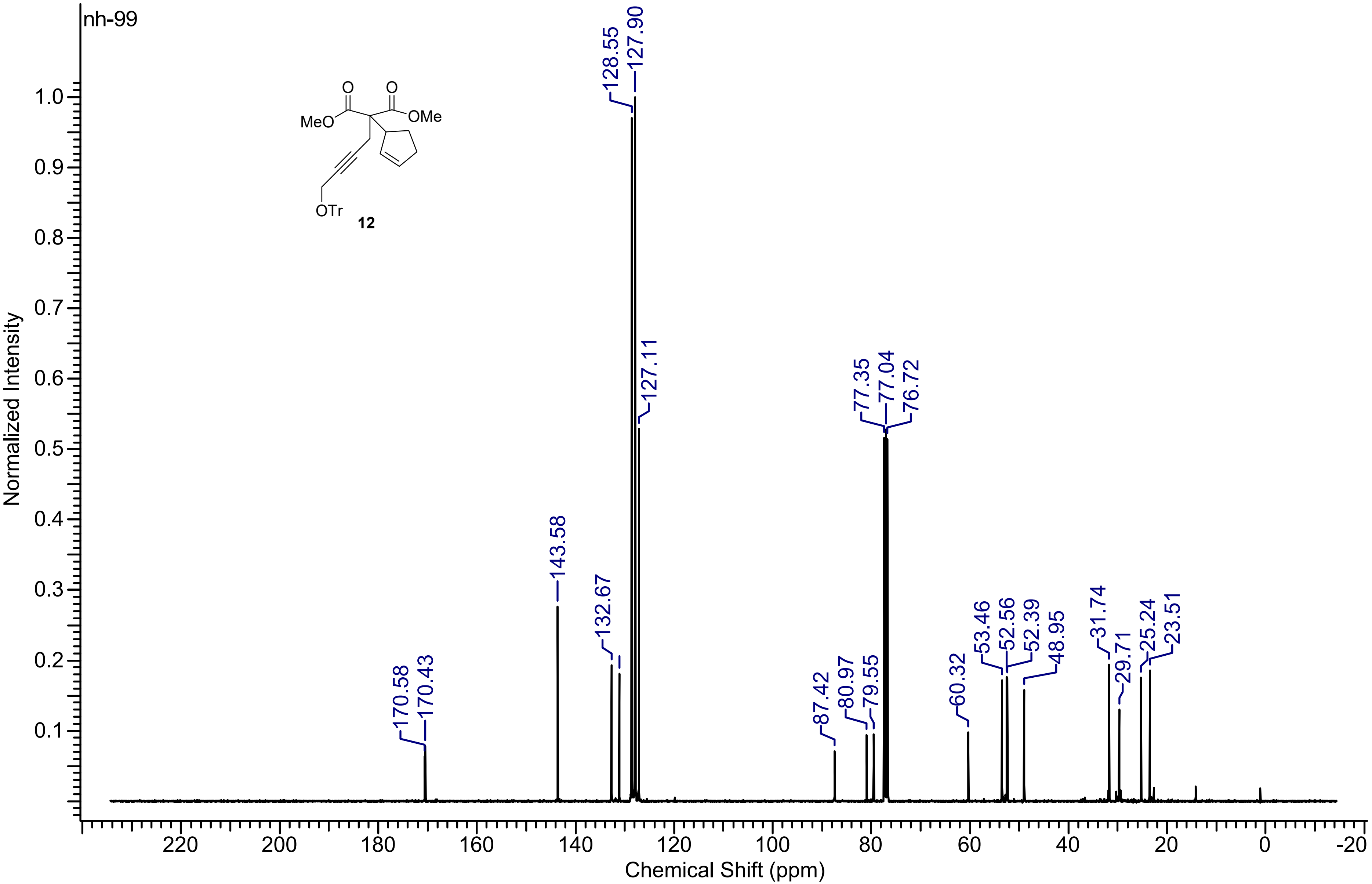
nh-99
MeO
OMe
O
O
OTr
12
170.58
170.43
143.58
132.67
128.55
127.90
127.11
87.42
80.97
79.55
77.35
77.04
76.72
60.32
53.46
52.56
52.39
48.95
31.74
29.71
25.24
23.51
Normalized Intensity
Chemical Shift (ppm)

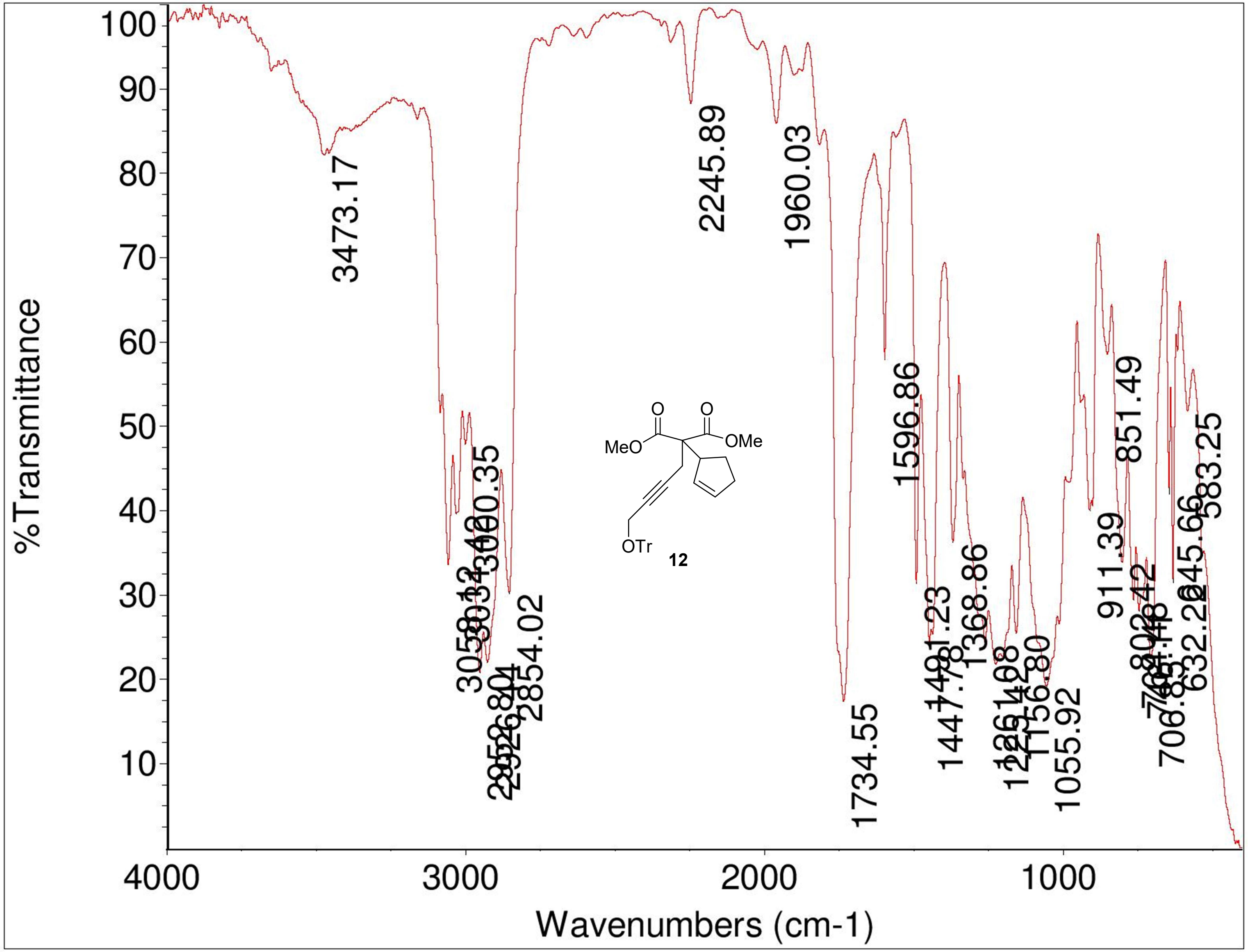
%Transmittance
Wavenumbers (cm-1)
3473.17
2245.89
1960.03
1734.55
1596.86
1368.86
1055.92
911.39
851.49
583.25
2854.02
MeO
OMe
O
O
OTr
12

LRMS [M+Na]$^+$

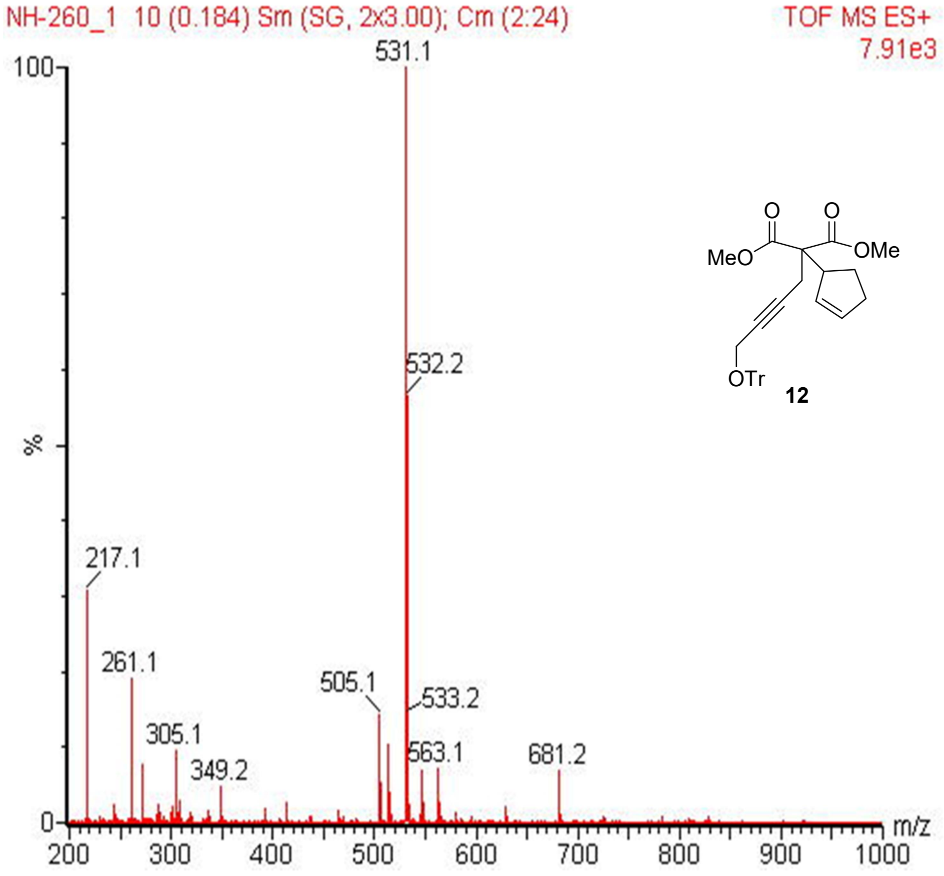

HRMS

Observed∆ = 1.4 mDa
Acceptable = ± 2.7 mDa

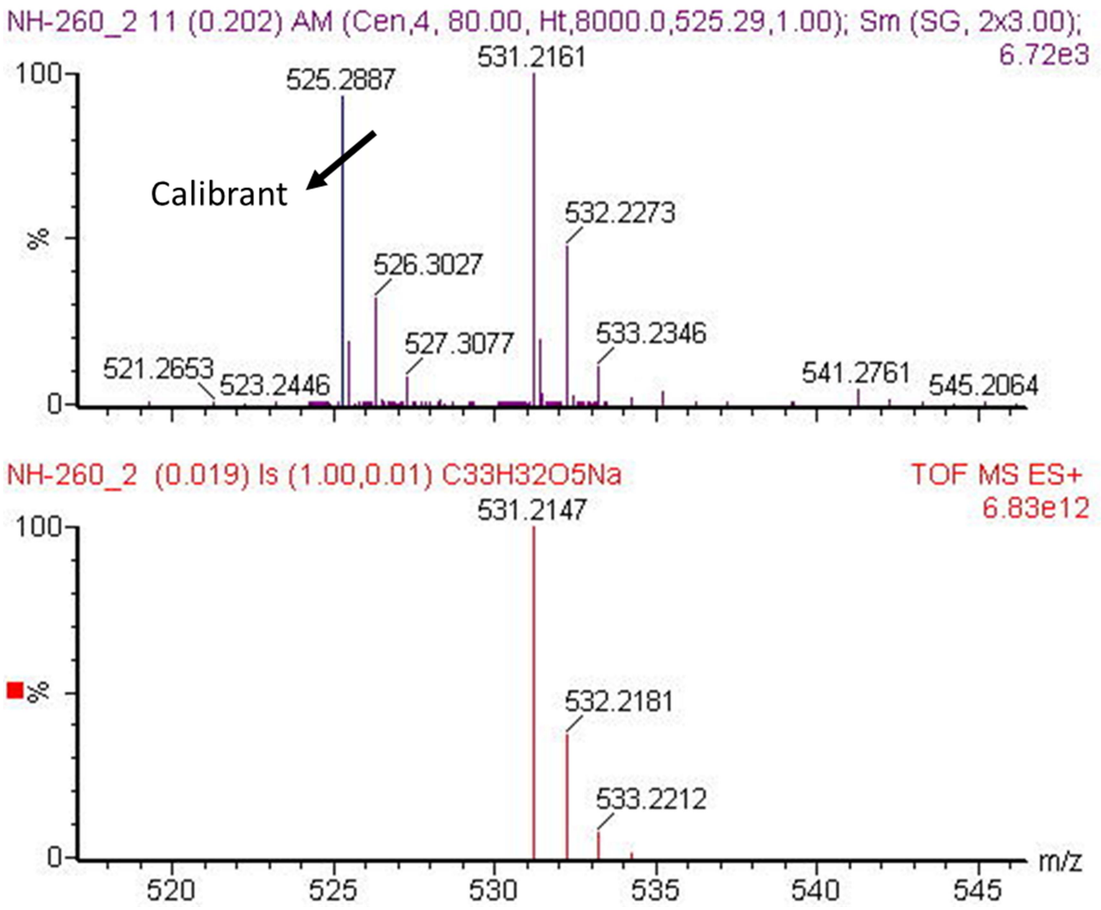

Theoretical =[M+Na]$^+$

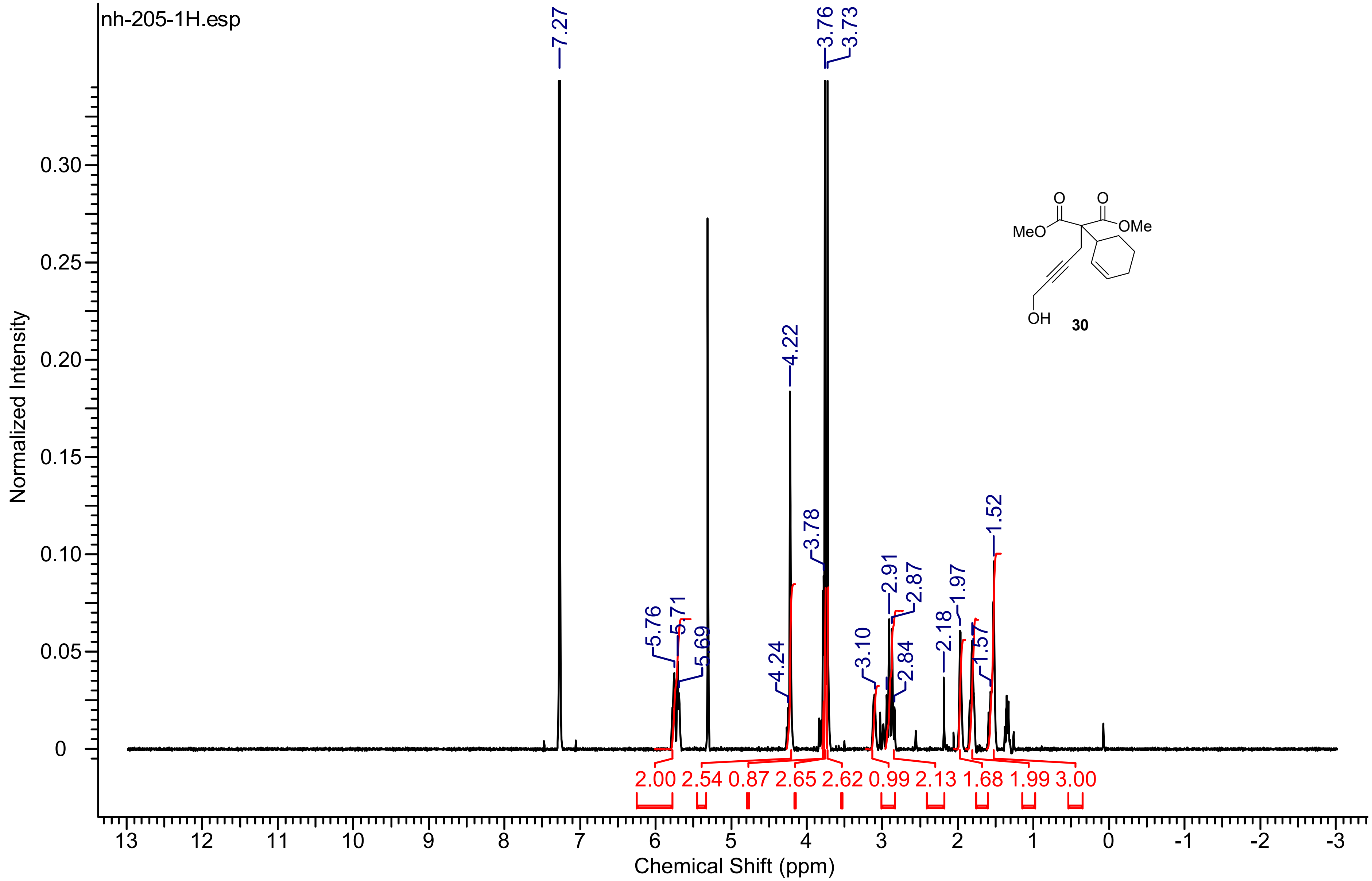
nh-205-1H.esp
Normalized Intensity
Chemical Shift (ppm)
7.27
3.76
3.73
4.22
3.78
5.76
5.71
5.69
4.24
3.10
2.91
2.87
2.84
2.18
1.97
1.57
1.52
2.00 2.54 0.87 2.65 2.62 0.99 2.13 1.68 1.99 3.00
MeO
OMe
O
O
OH

30

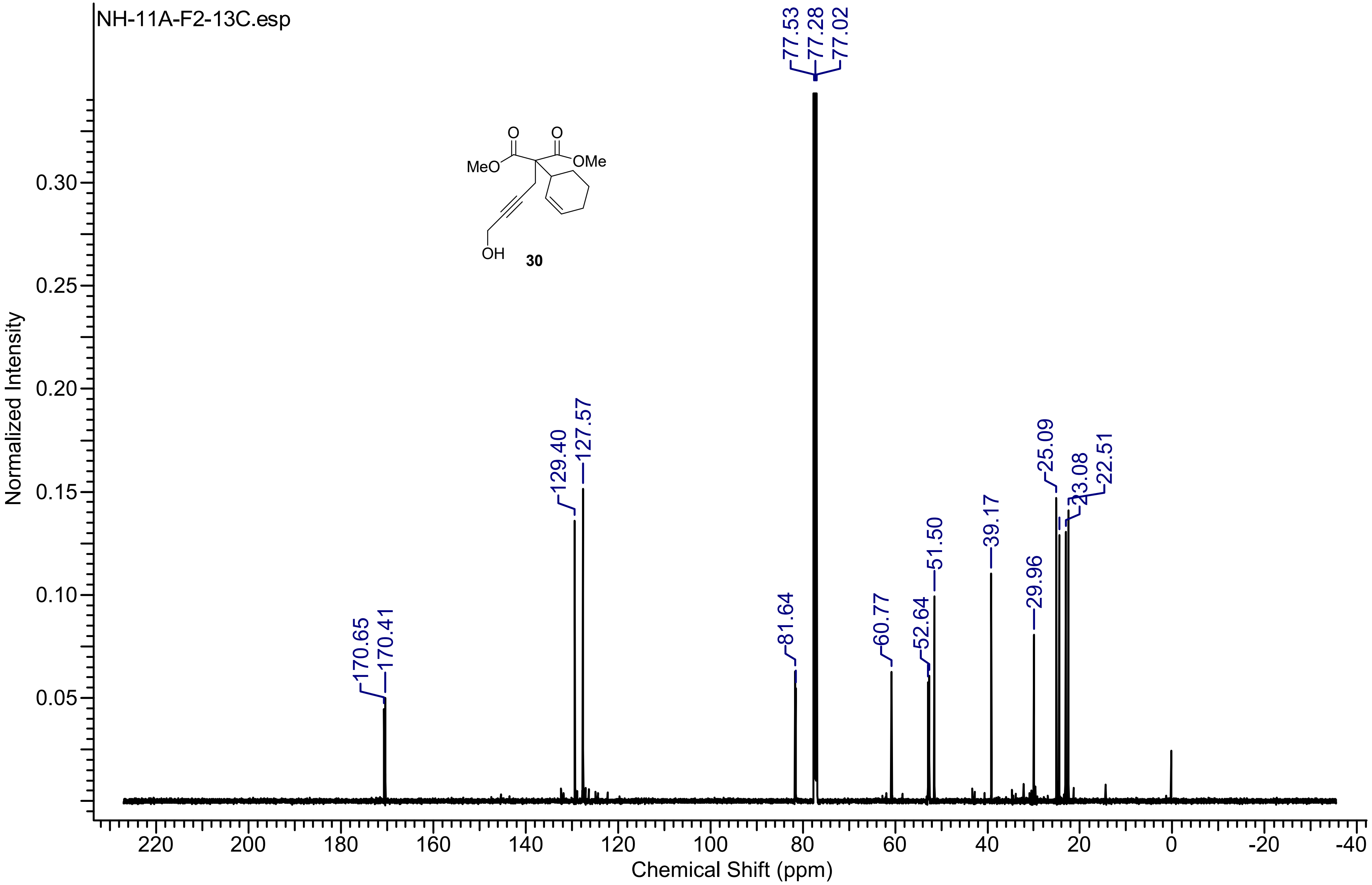
NH-11A-F2-13C.esp
MeO
O
O
OMe
OH
30
77.53
77.28
77.02
170.65
170.41
129.40
127.57
81.64
60.77
52.64
51.50
39.17
29.96
25.09
23.08
22.51
Normalized Intensity
0.30
0.25
0.20
0.15
0.10
0.05
220
200
180
160
140
120
100
80
60
40
20
0
-20
-40
Chemical Shift (ppm)

v

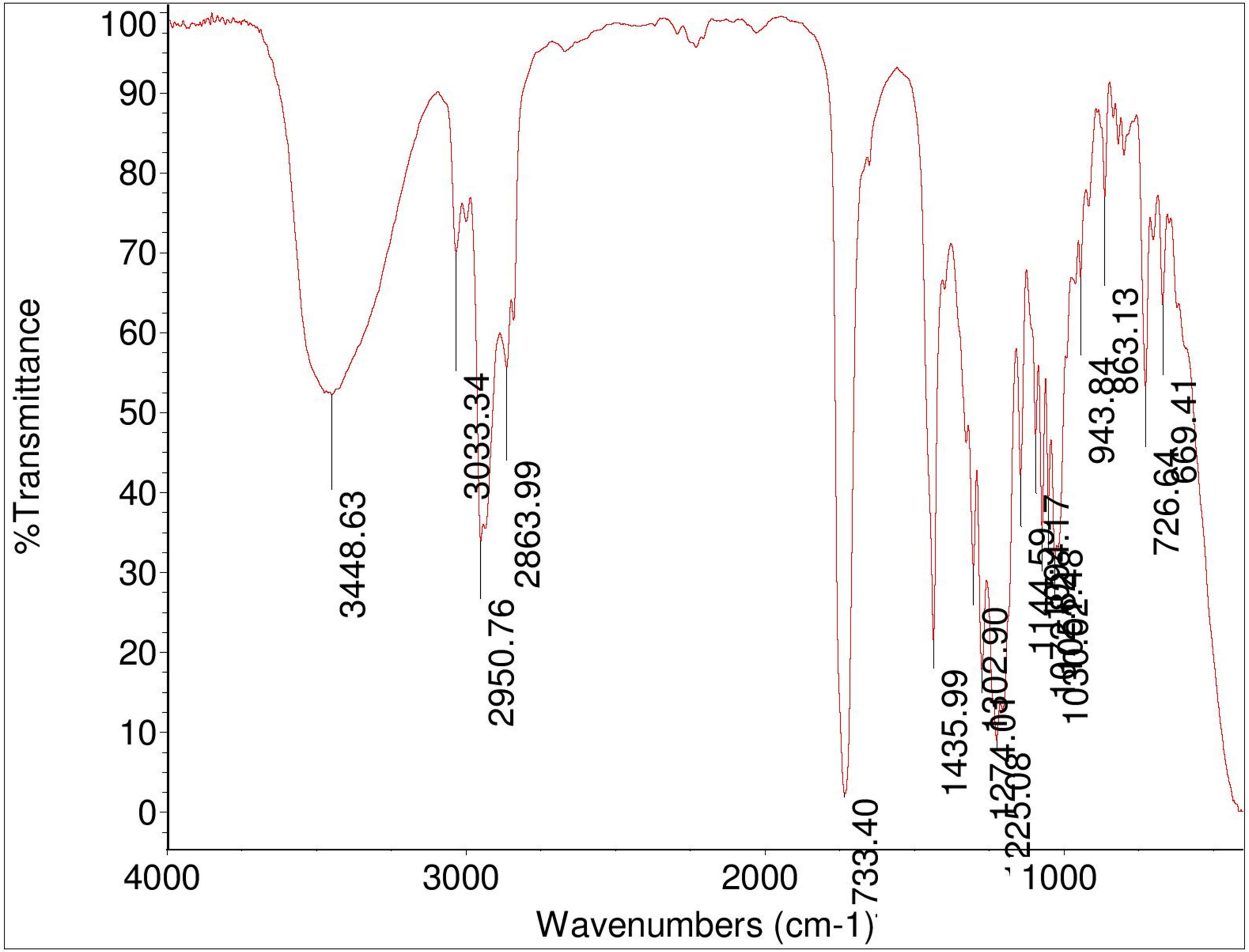
%Transmittance
Wavenumbers (cm-1)
100
90
80
70
60
50
40
30
20
10
0
4000
3000
2000
1000
3448.63
3033.34
2950.76
2863.99
733.40
1435.99
1302.90
1144.59
943.84
863.13
726.64
669.41

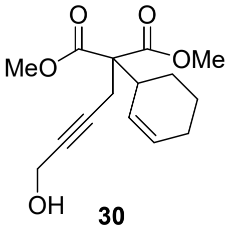
MeO
OMe
O
O
OH

30

LRMS [M+Na]

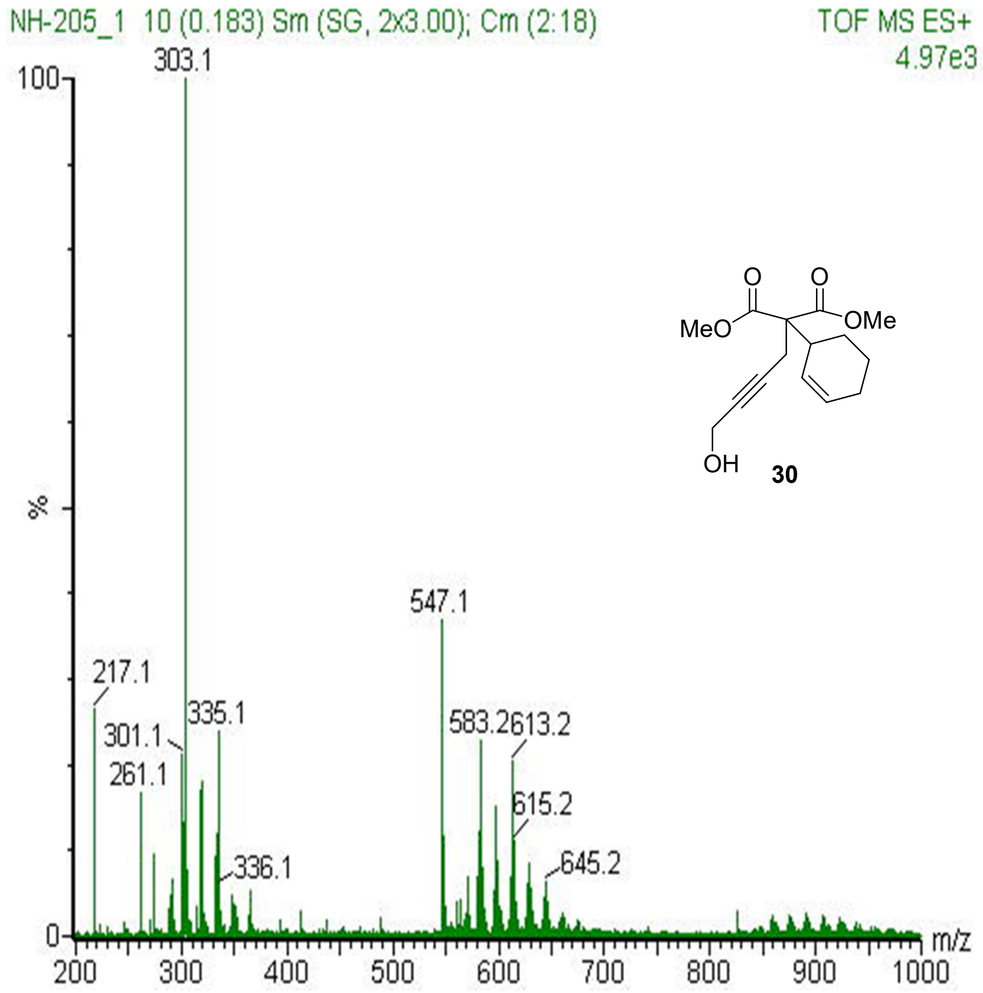

HRMS Observed∆ = 0.1 mDa
Acceptable = ± 1.5 mDa

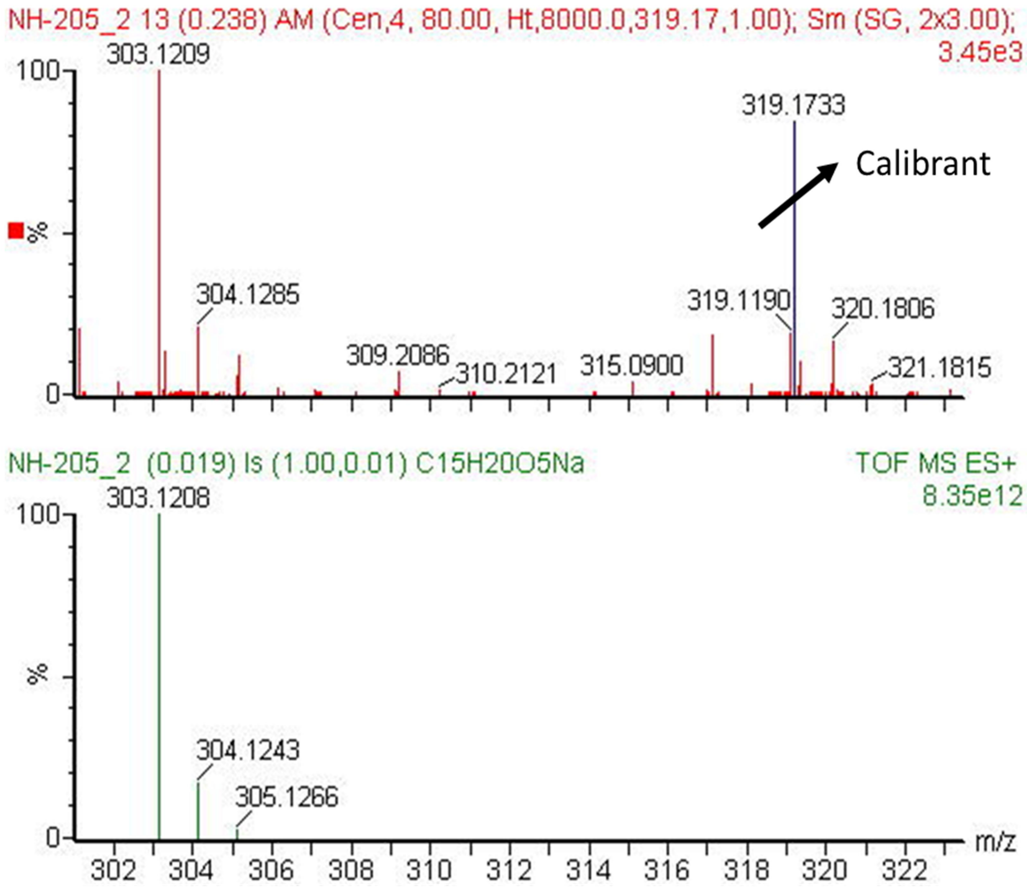

Theoretical =[M+Na]$^+$

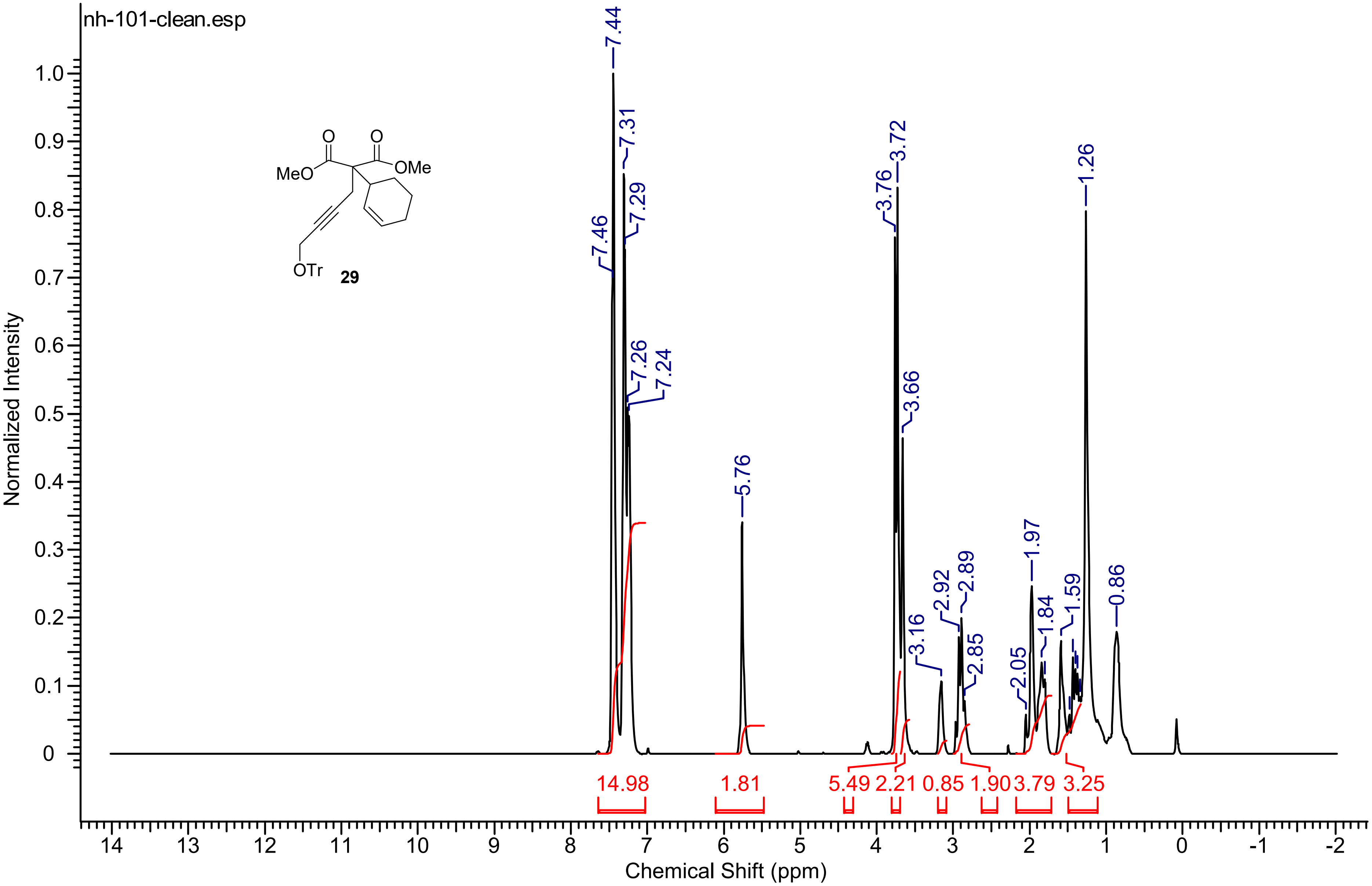

nh-101-clean.esp
MeO
O
O
OMe
OTr
29
7.44
7.31
7.29
7.46
7.26
7.24
5.76
3.76
3.72
3.66
3.16
2.92
2.89
2.85
2.05
1.97
1.84
1.59
1.26
0.86
14.98
1.81
5.49
2.21
0.85
1.90
3.79
3.25
Normalized Intensity
Chemical Shift (ppm)

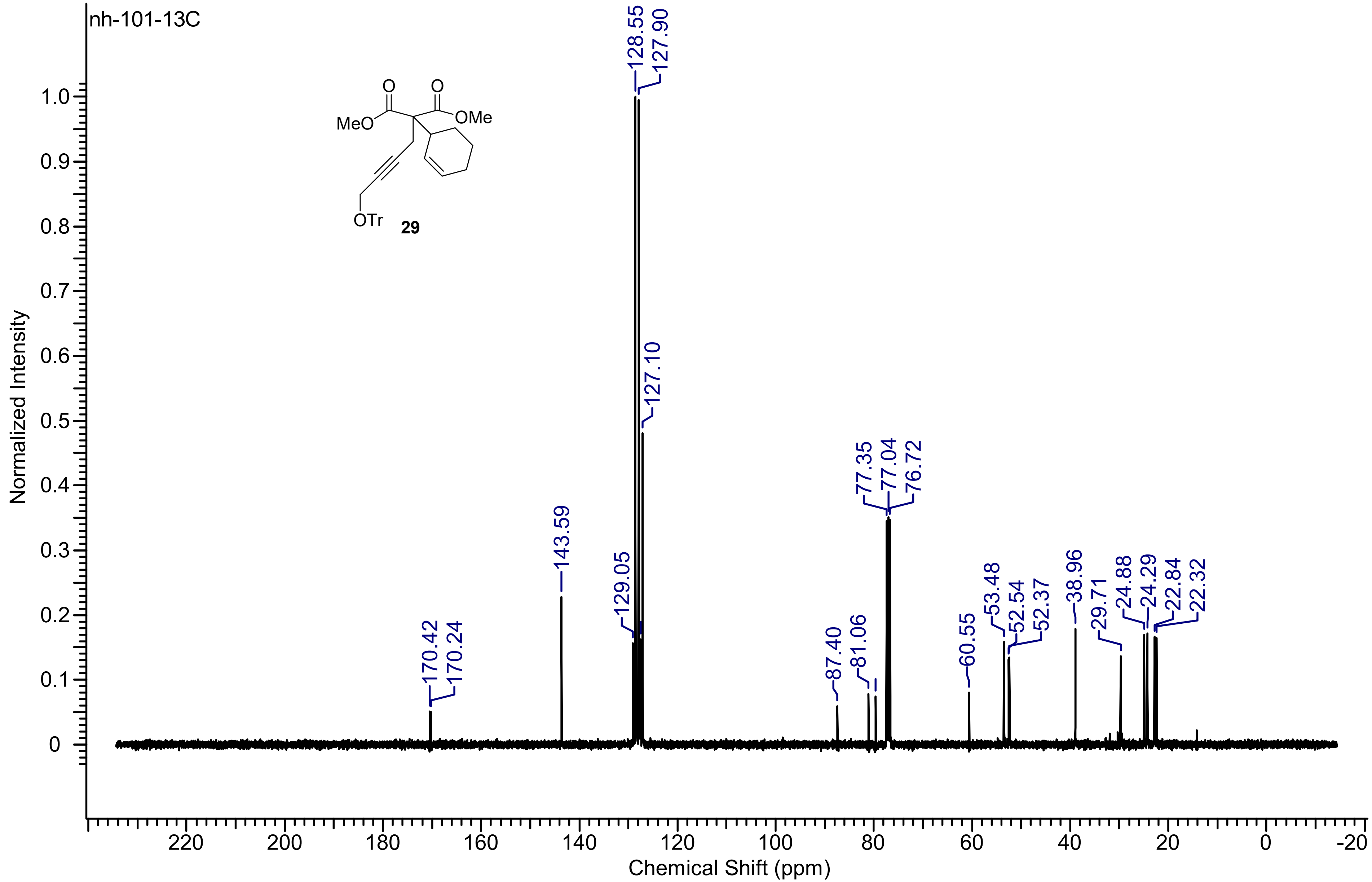
nh-101-13C
MeO
OMe
O
O
OTr
29
128.55
127.90
127.10
143.59
129.05
170.42
170.24
87.40
81.06
77.35
77.04
76.72
60.55
53.48
52.54
52.37
38.96
29.71
24.88
24.29
22.84
22.32
Normalized Intensity
Chemical Shift (ppm)

LRMS [M+Na]$^+$

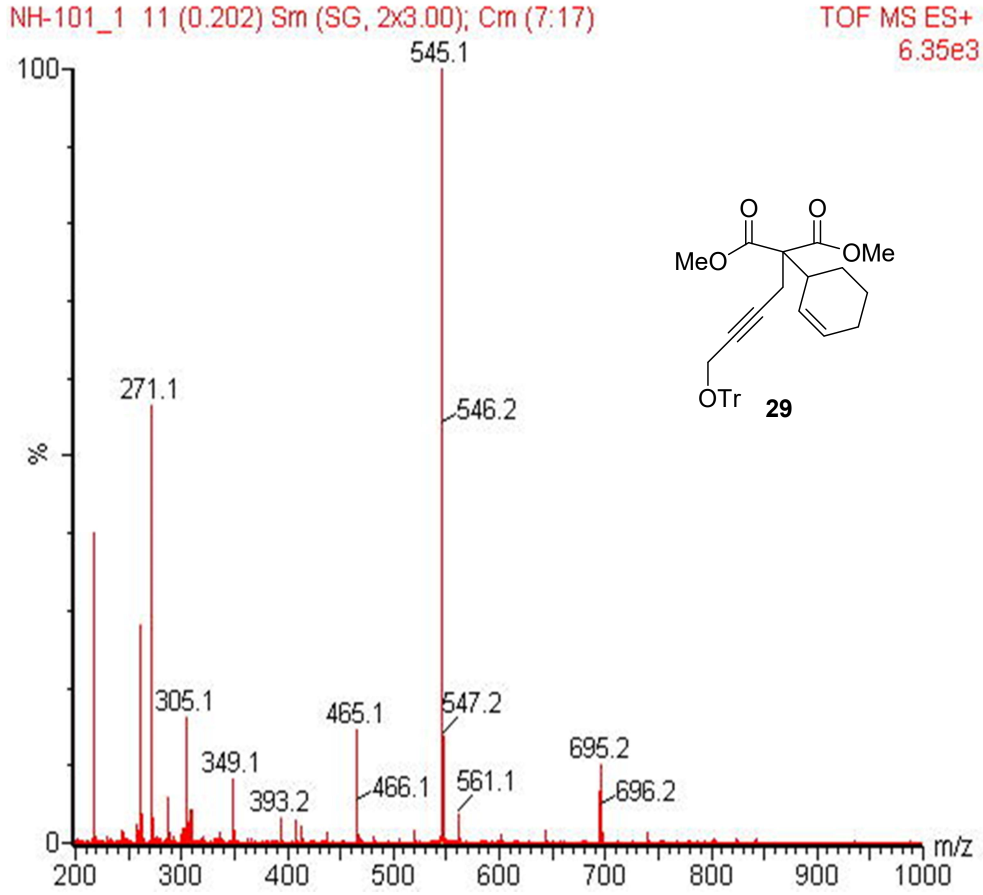

HRMS Observed∆ = 2.4 mDa
Acceptable = ± 2.7 mDa

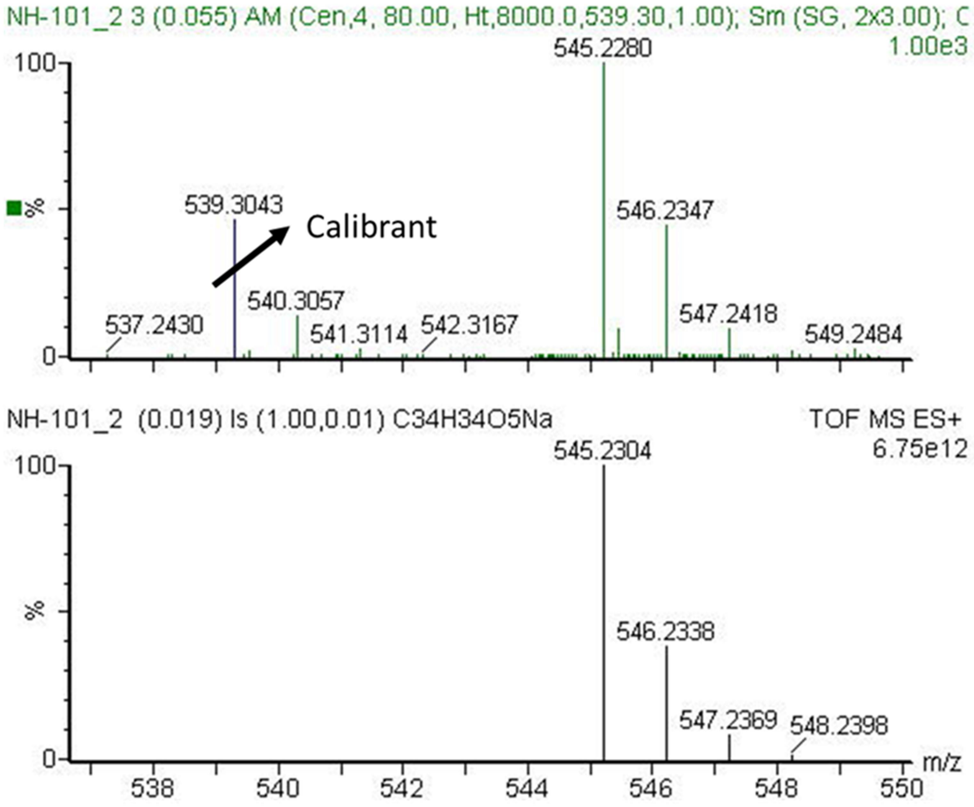

Theoretical =[M+Na]$^+$

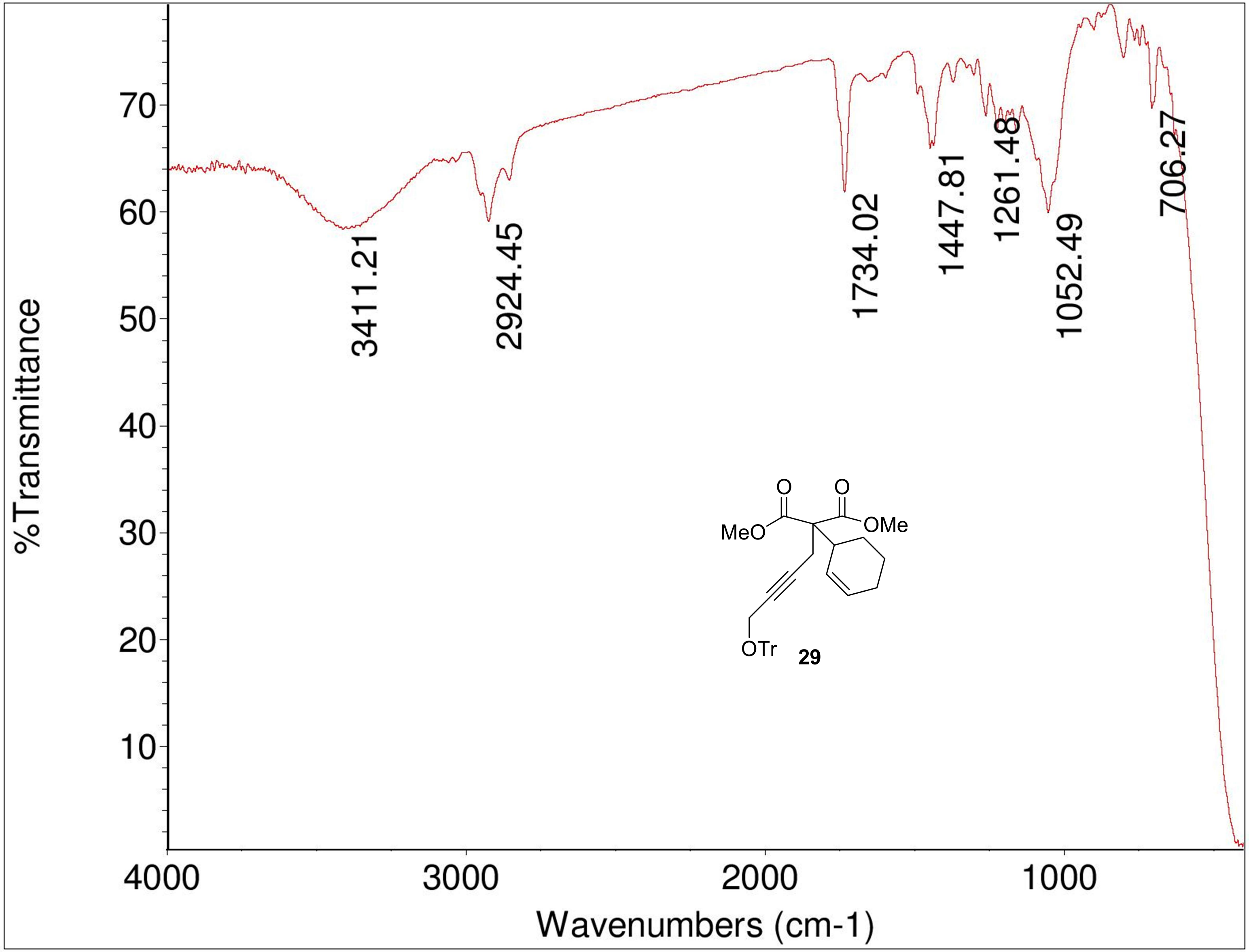

%Transmittance
Wavenumbers (cm-1)
70
60
50
40
30
20
10
4000
3000
2000
1000
3411.21
2924.45
1734.02
1447.81
1261.48
1052.49
706.27
MeO
OMe
O
O
OTr
29

HRMS ObservedΔ = 2.4 mDa
Acceptable = $\pm$ 2.7 mDa

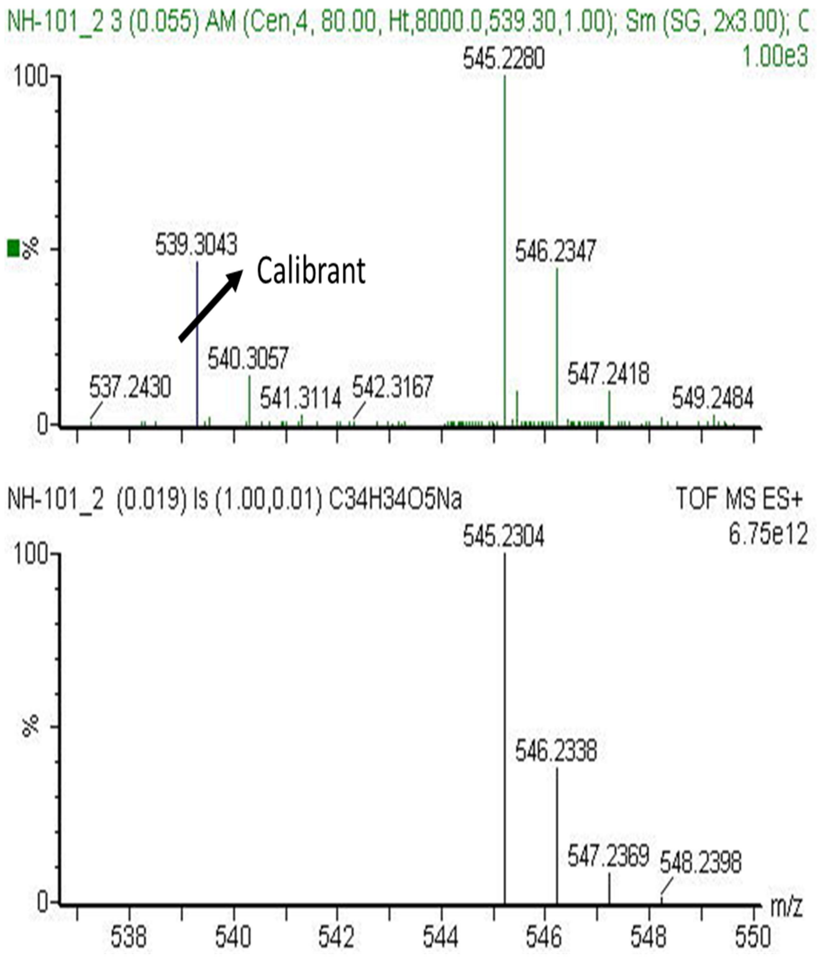

Theoretical =[M+Na]$^+$

LRMS [M+Na]$^+$

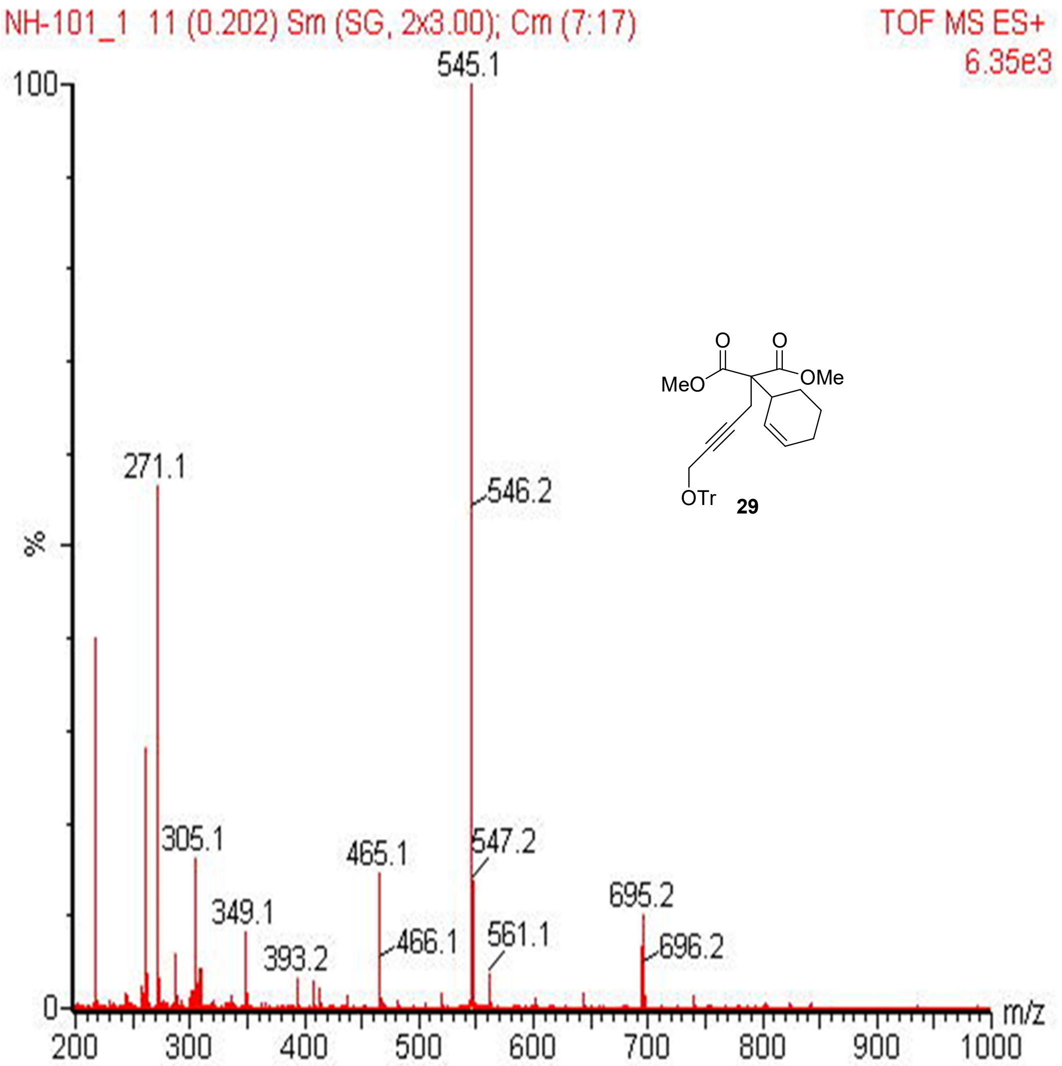

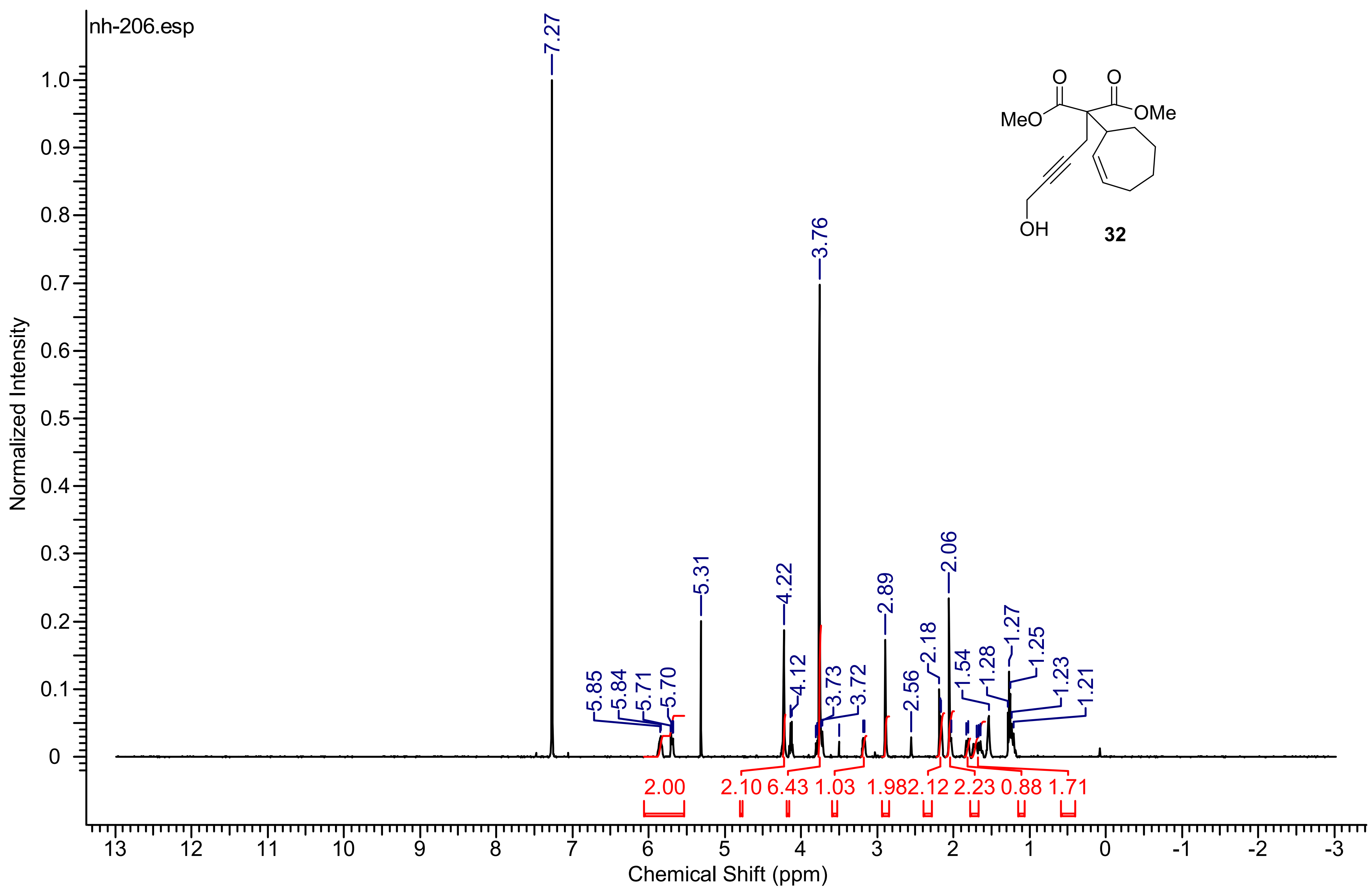
nh-206.esp
7.27
3.76
5.31
4.22
2.89
2.06
5.85
5.84
5.71
5.70
4.12
3.73
3.72
2.56
2.18
1.54
1.28
1.27
1.25
1.23
1.21
2.00
2.10 6.43 1.03 1.982.12 2.23 0.88 1.71
MeO
OMe
O
O
OH
32
Normalized Intensity
Chemical Shift (ppm)

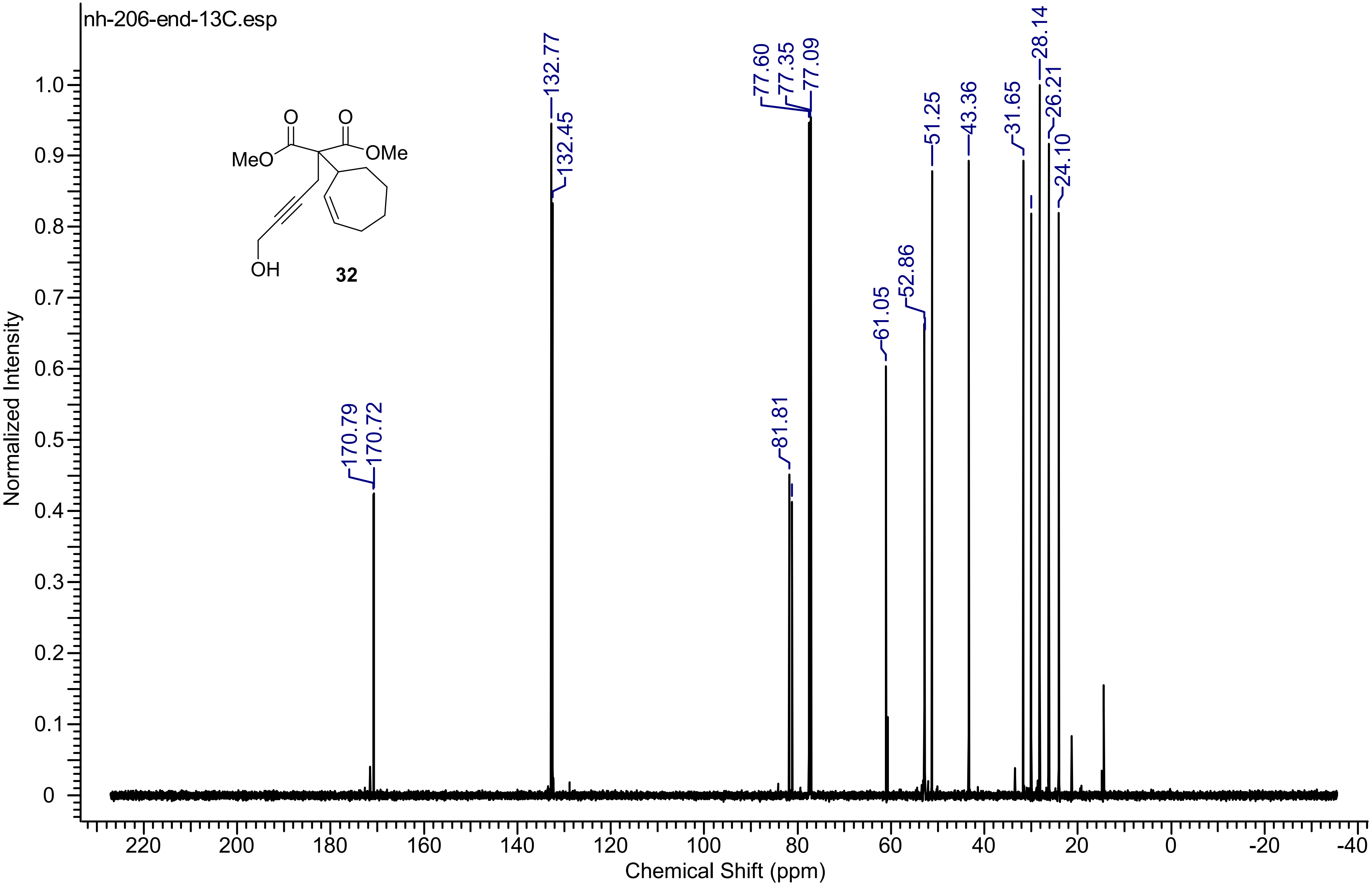
nh-206-end-13C.esp
MeO
O
O
OMe
OH
32
132.77
132.45
77.60
77.35
77.09
81.81
170.79
170.72
61.05
52.86
51.25
43.36
31.65
28.14
26.21
24.10
Normalized Intensity
Chemical Shift (ppm)

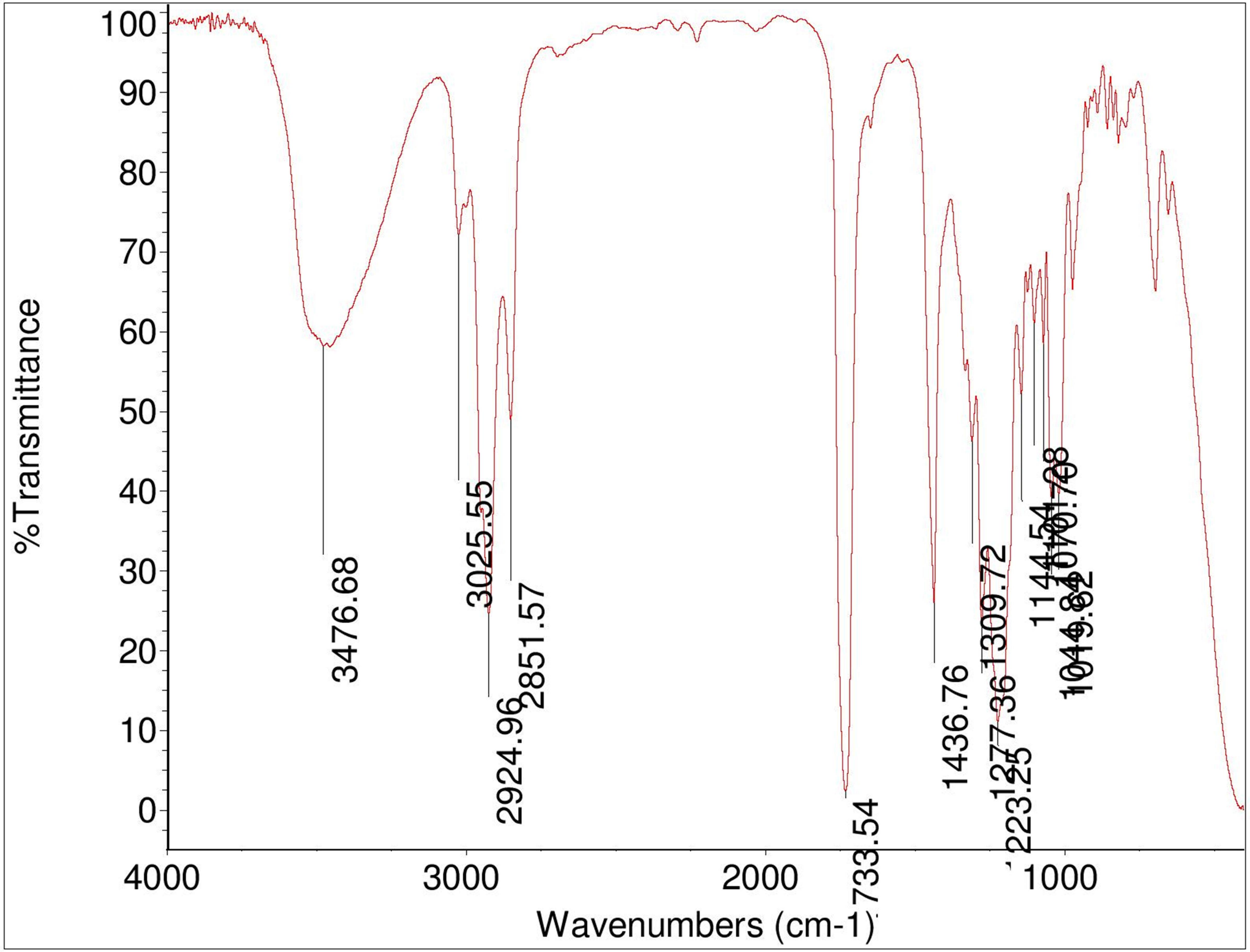
%Transmittance
Wavenumbers (cm-1)
3476.68
3025.55
2924.96
2851.57
1733.54
1436.76
1309.72
1277.36
1223.25
1144.54
1070.73
1044.84
1019.62

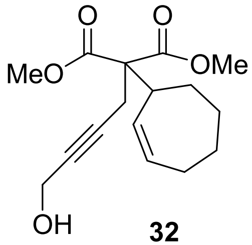
MeO
OMe
O
O
OH

32

HRMS

ObservedΔ = 0.7 mDa

Acceptable = ± 1.6 mDa

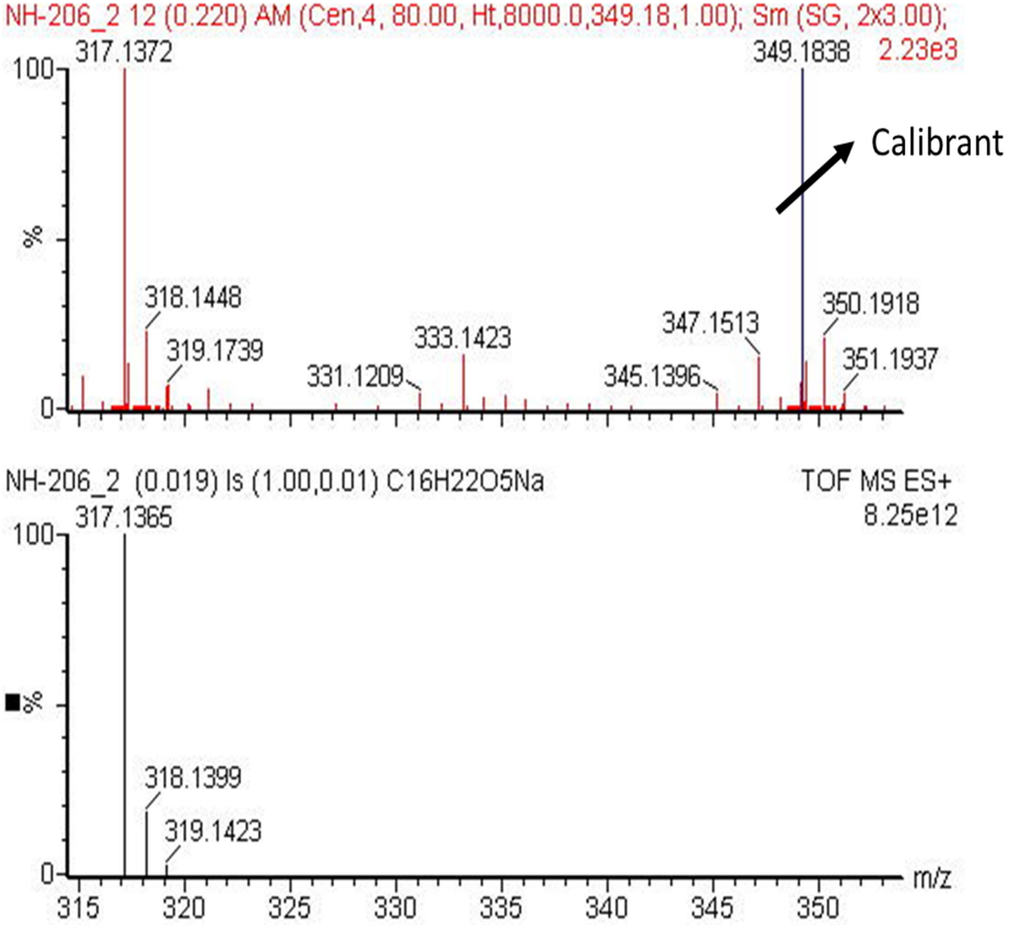

Theoretical =[M+Na]$^{+}$

LRMS [M+Na]$^{+}$

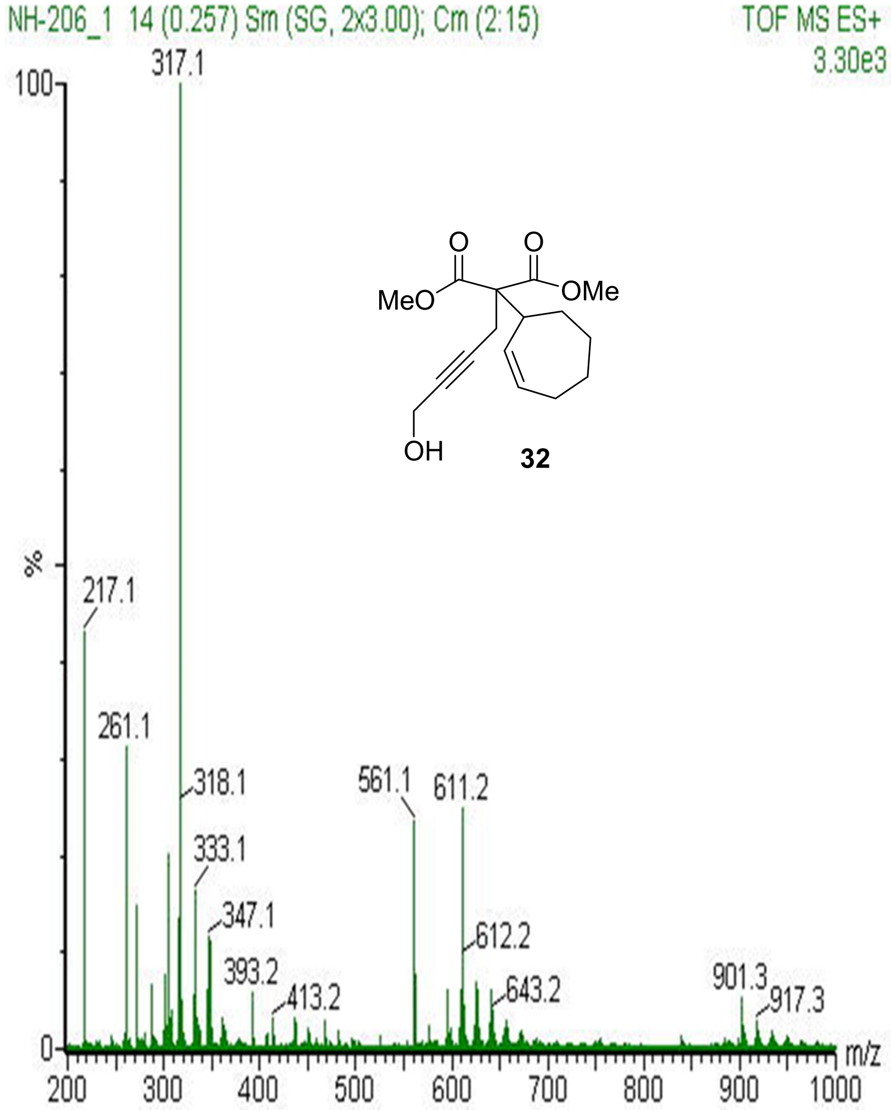

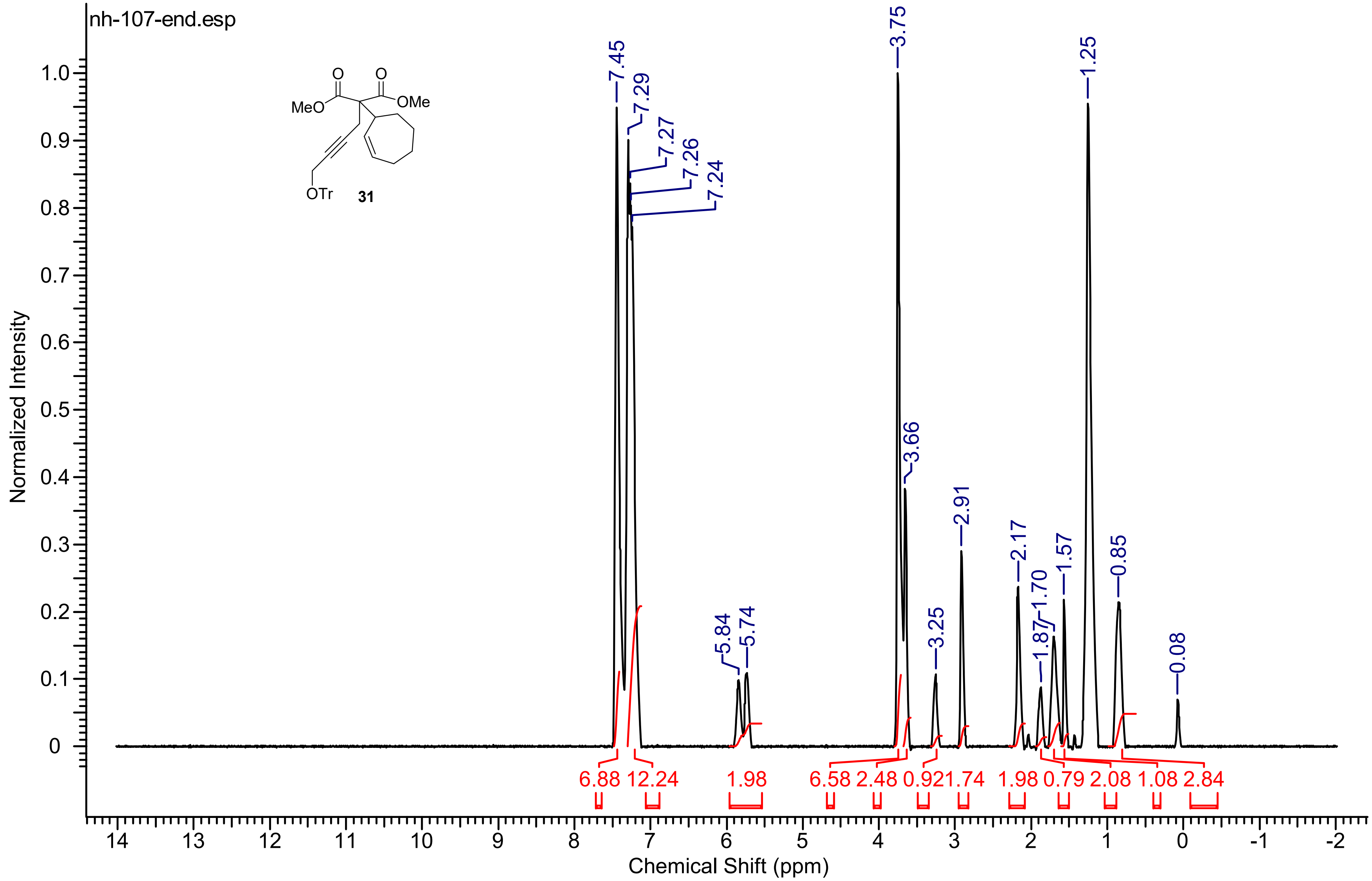
nh-107-end.esp
MeO
OMe
OTr
31
7.45
7.29
7.27
7.26
7.24
5.84
5.74
3.75
3.66
3.25
2.91
2.17
1.87
1.70
1.57
1.25
0.85
0.08
6.88
12.24
1.98
6.58
2.48
0.92
1.74
1.98
0.79
2.08
1.08
2.84
Normalized Intensity
Chemical Shift (ppm)

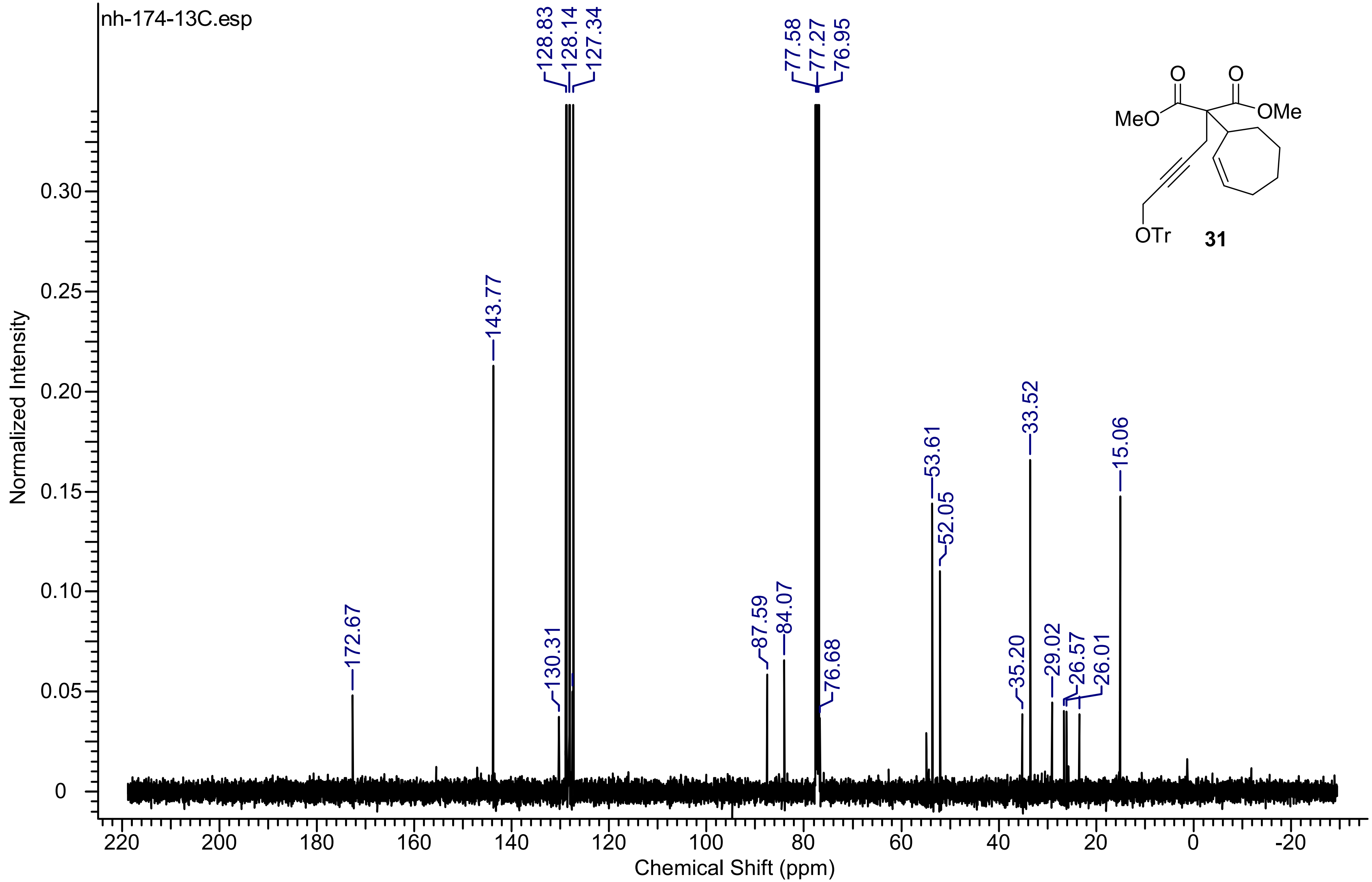
nh-174-13C.esp
128.83
128.14
127.34
77.58
77.27
76.95
143.77
172.67
130.31
87.59
84.07
76.68
53.61
52.05
33.52
35.20
29.02
26.57
26.01
15.06
MeO
OMe
O
O
OTr
31
Normalized Intensity
Chemical Shift (ppm)
0.30
0.25
0.20
0.15
0.10
0.05
0
220
200
180
160
140
120
100
80
60
40
20
0
-20

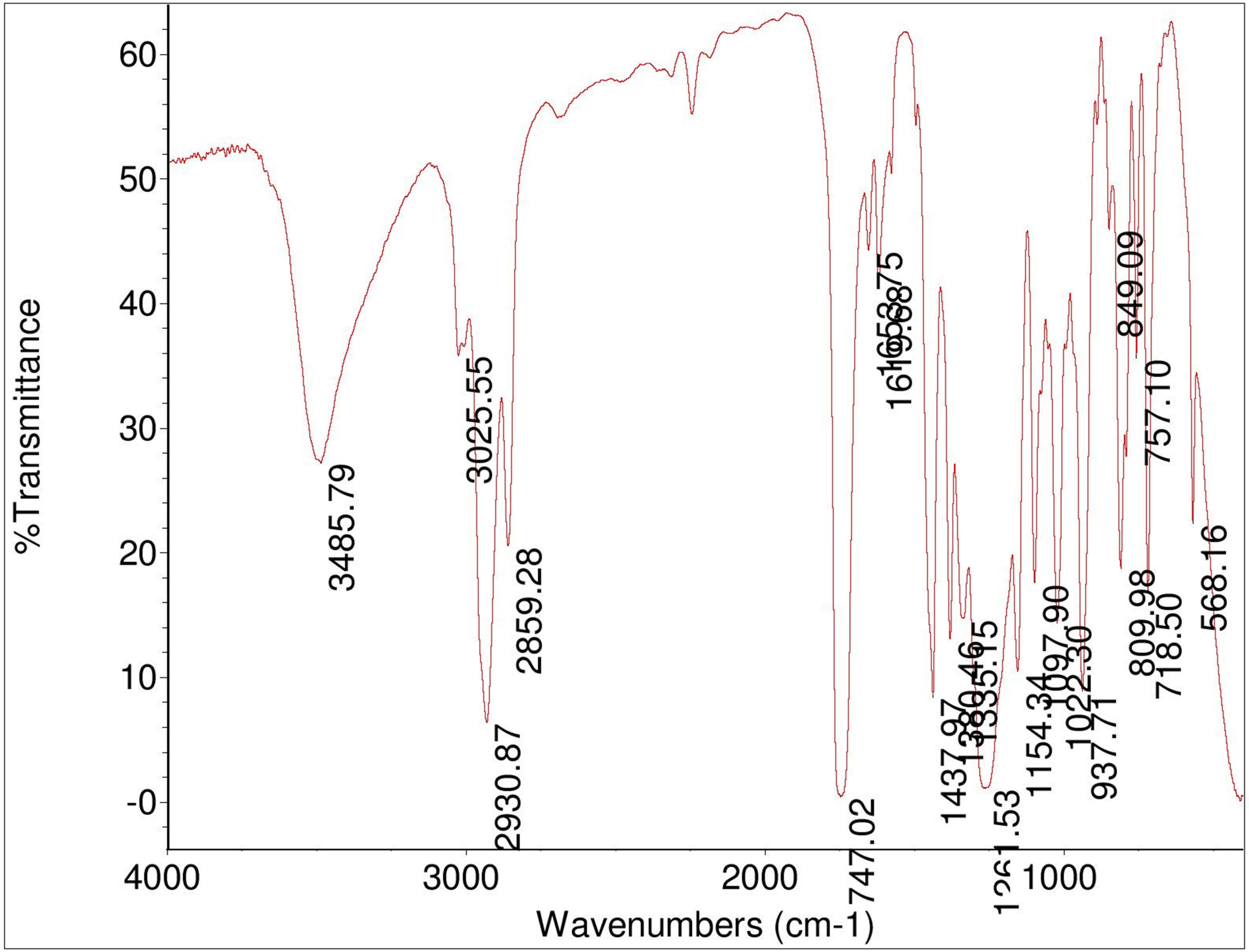
%Transmittance
Wavenumbers (cm-1)
60
50
40
30
20
10
-0
4000
3000
2000
1000
3485.79
3025.55
2930.87
2859.28
747.02
1653.75
1619.68
1437.97
1380.46
1335.15
1261.53
1154.34
1097.90
1022.30
937.71
849.09
809.98
757.10
718.50
568.16

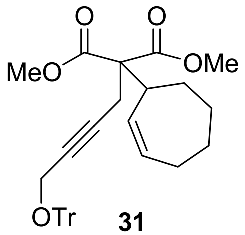
MeO
OMe
O
O
OTr

31

LRMS [M+Na]$^+$

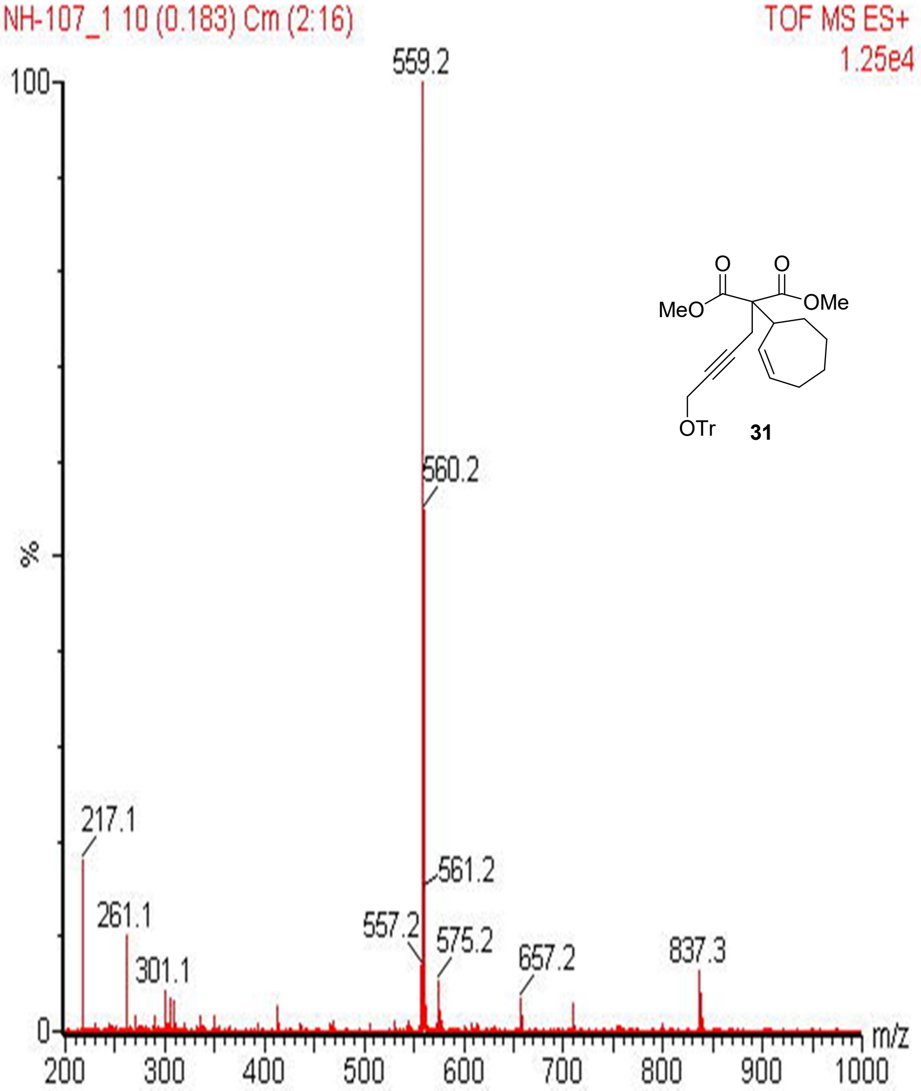

31

HRMS

ObservedΔ = 2.6 mDa
Acceptable = ± 2.8 mDa

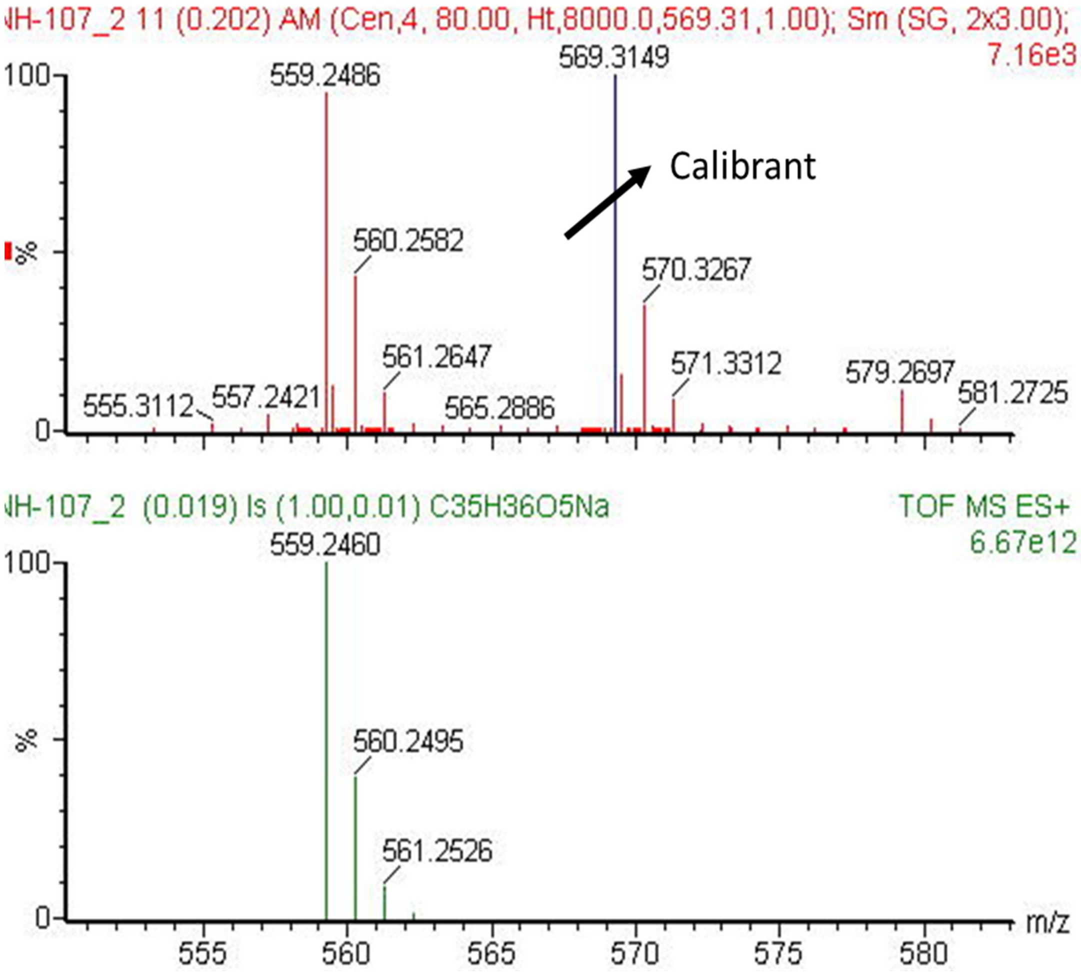

Theoretical =[M+Na]$^+$

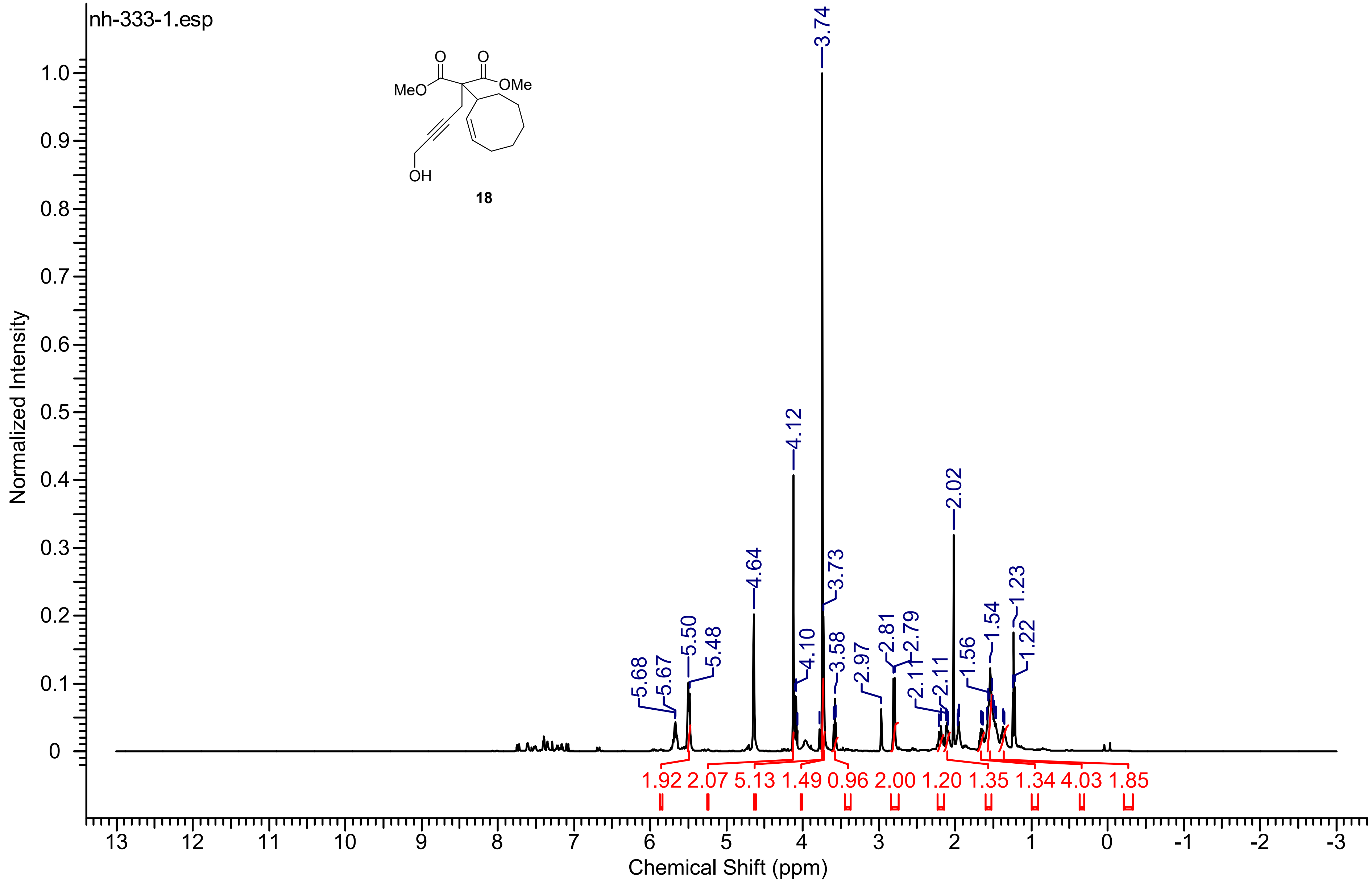
nh-333-1.esp
MeO
O
O
OMe
OH
18
Normalized Intensity
Chemical Shift (ppm)
5.68
5.67
5.50
5.48
4.64
4.12
4.10
3.74
3.73
3.58
2.97
2.81
2.79
2.11
2.11
2.02
1.56
1.54
1.23
1.22
1.92 2.07 5.13 1.49 0.96 2.00 1.20 1.35 1.34 4.03 1.85

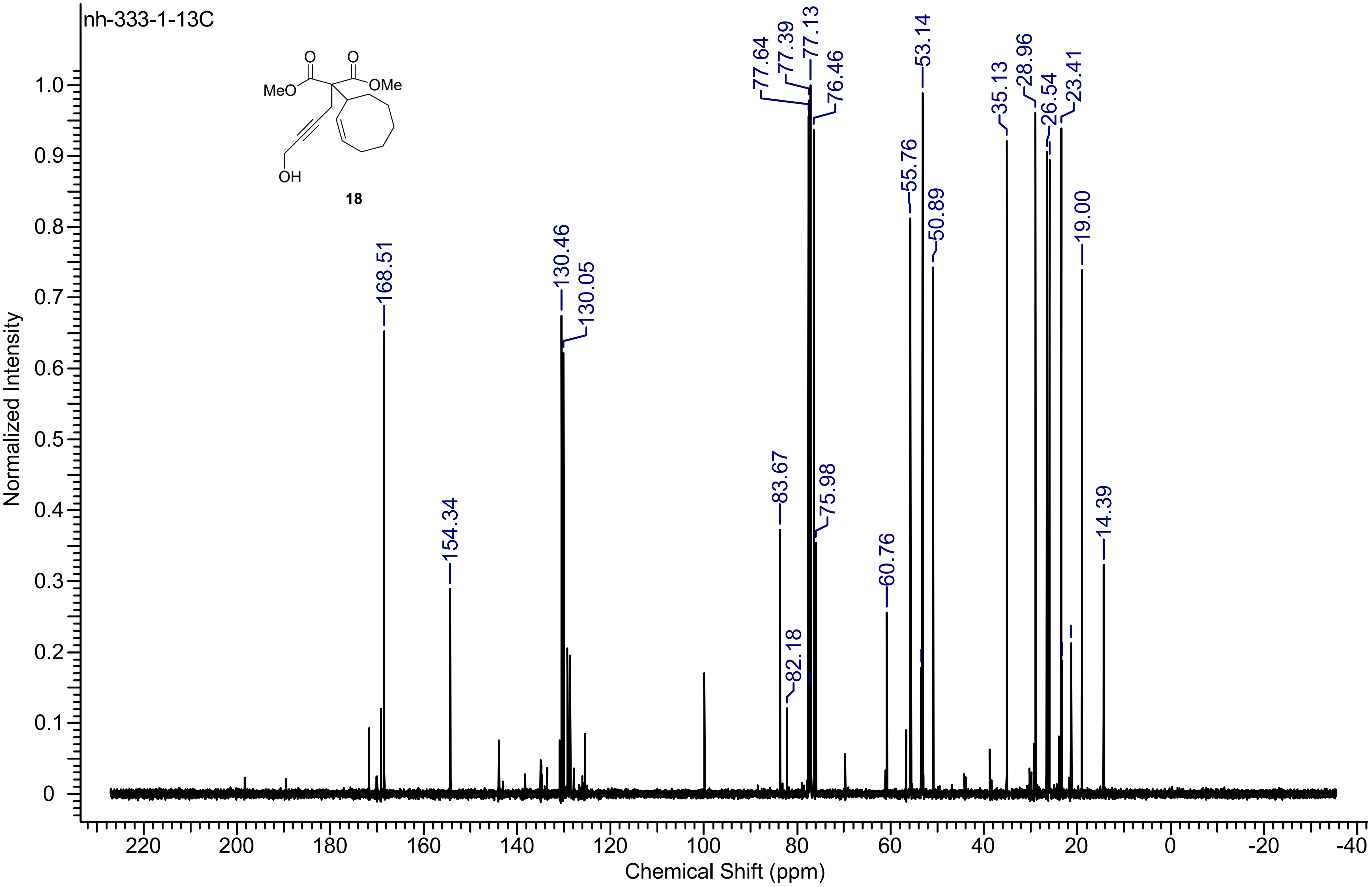
nh-333-1-13C
MeO
OMe
O
O
OH
18
168.51
154.34
130.46
130.05
83.67
82.18
77.64
77.39
77.13
76.46
75.98
60.76
55.76
53.14
50.89
35.13
28.96
26.54
23.41
19.00
14.39
Normalized Intensity
Chemical Shift (ppm)

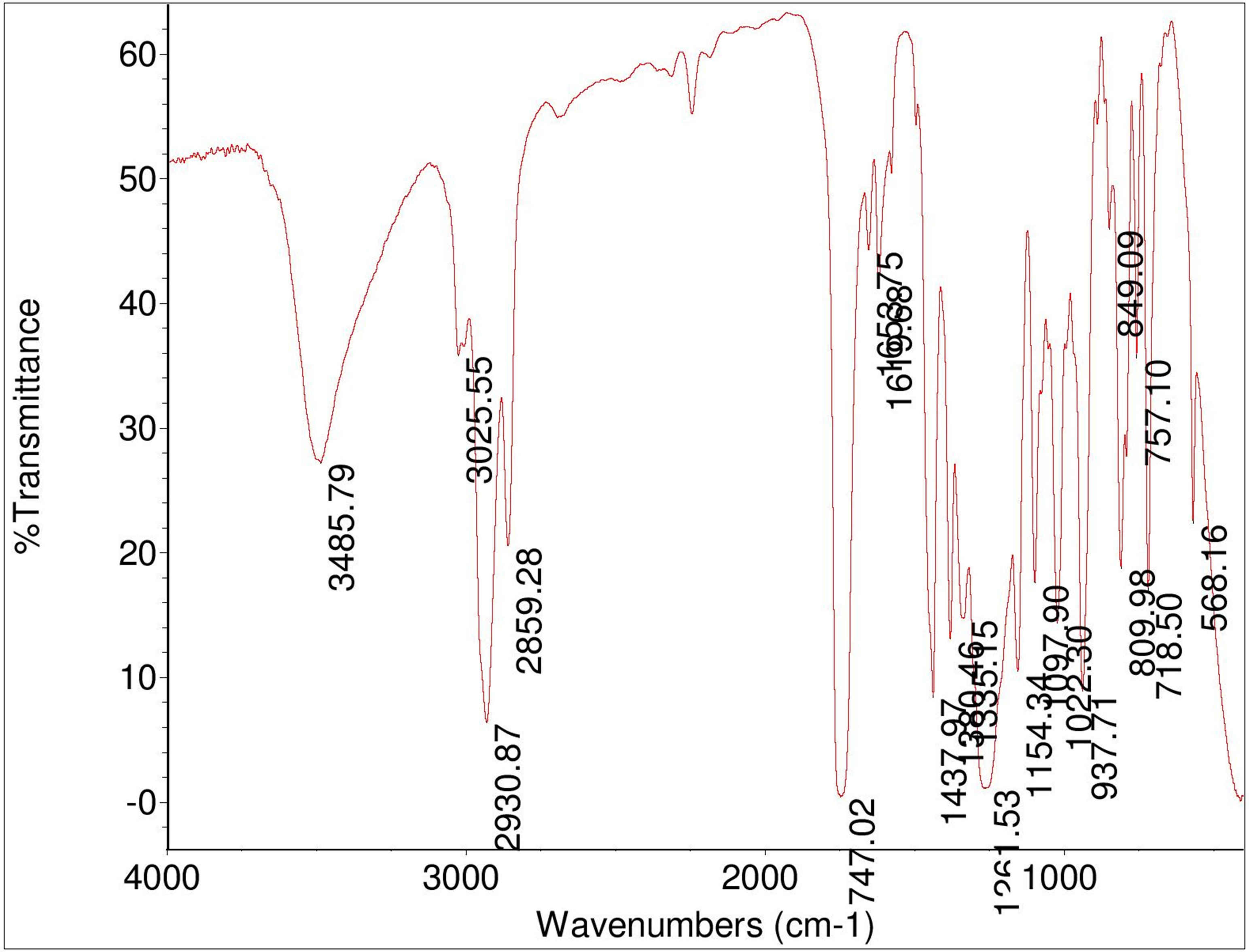
%Transmittance
Wavenumbers (cm-1)
3485.79
3025.55
2930.87
2859.28
1653.75
1619.68
1437.97
1154.34
1097.90
937.71
849.09
809.98
757.10
718.50
568.16

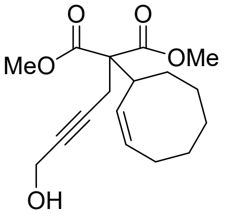
O
O
MeO
OMe
OH

18

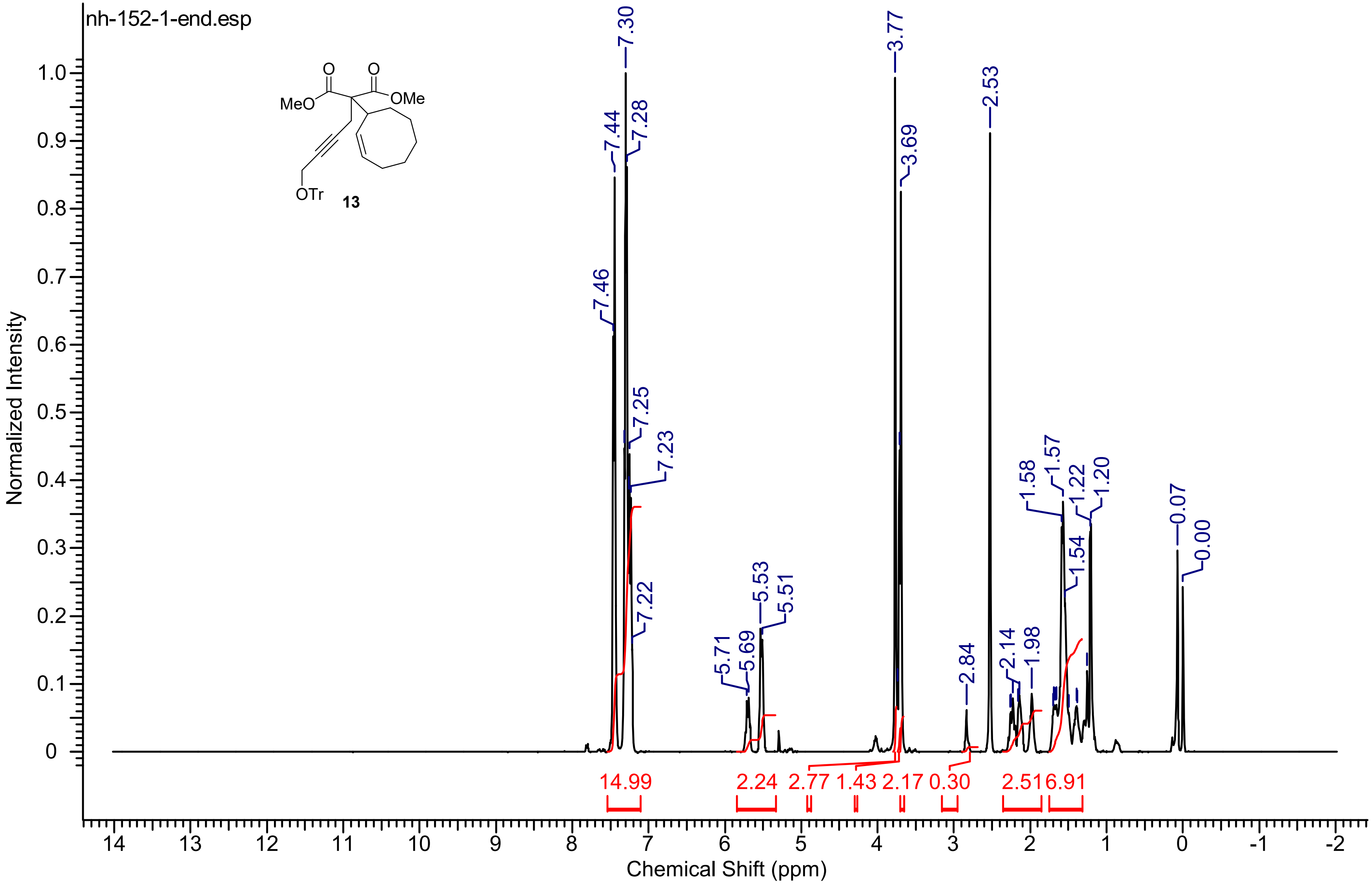
nh-152-1-end.esp
MeO
OMe
OTr
13
Normalized Intensity
Chemical Shift (ppm)
7.46
7.44
7.30
7.28
7.25
7.23
7.22
5.71
5.69
5.53
5.51
3.77
3.69
2.84
2.53
2.14
1.98
1.58
1.57
1.54
1.22
1.20
0.07
0.00
14.99
2.24
2.77
1.43
2.17
0.30
2.51
6.91

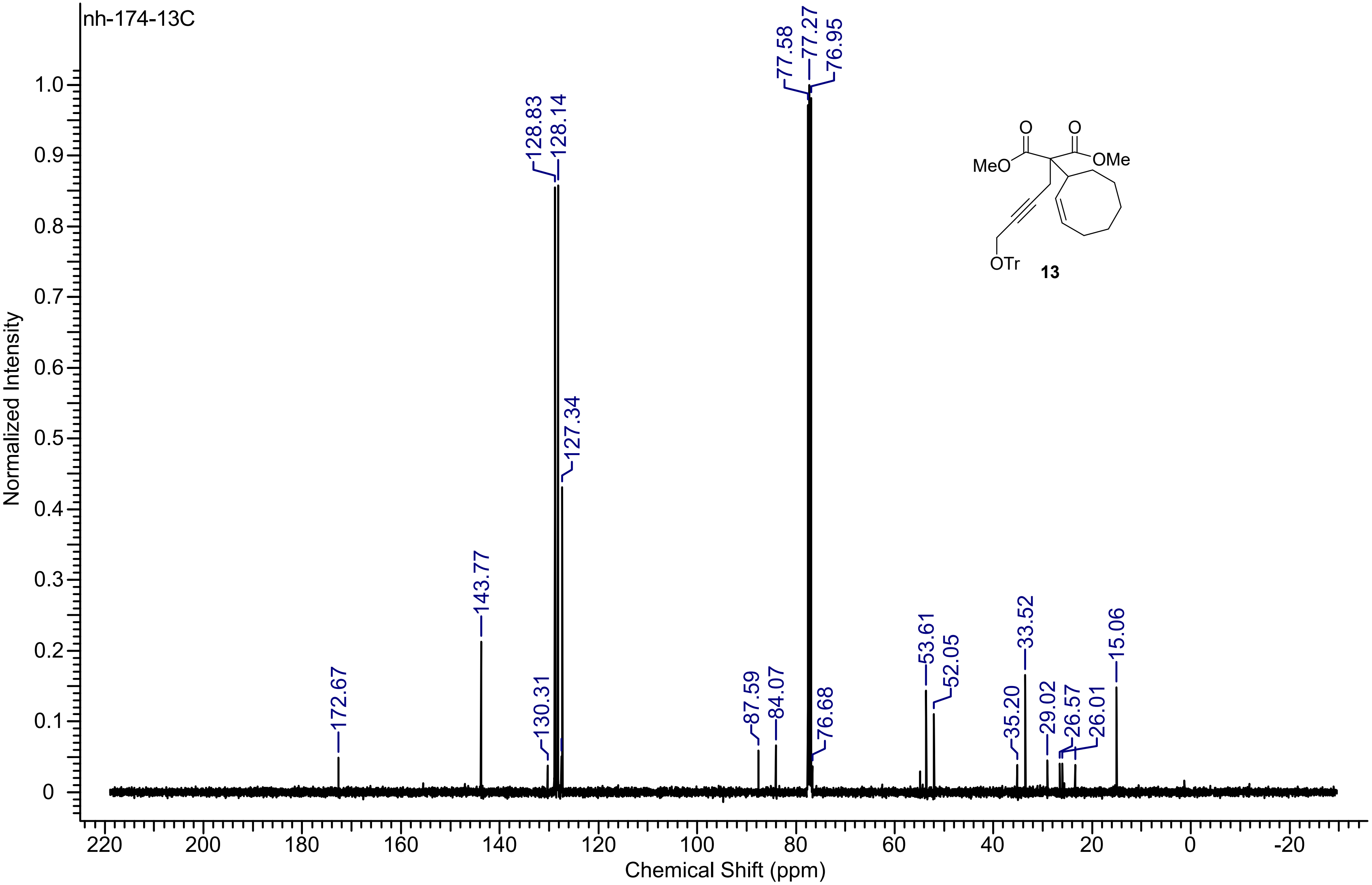
nh-174-13C
172.67
143.77
130.31
128.83
128.14
127.34
87.59
84.07
77.58
77.27
76.95
76.68
53.61
52.05
35.20
33.52
29.02
26.57
26.01
15.06
MeO
OMe
O
O
OTr
13
Normalized Intensity
Chemical Shift (ppm)

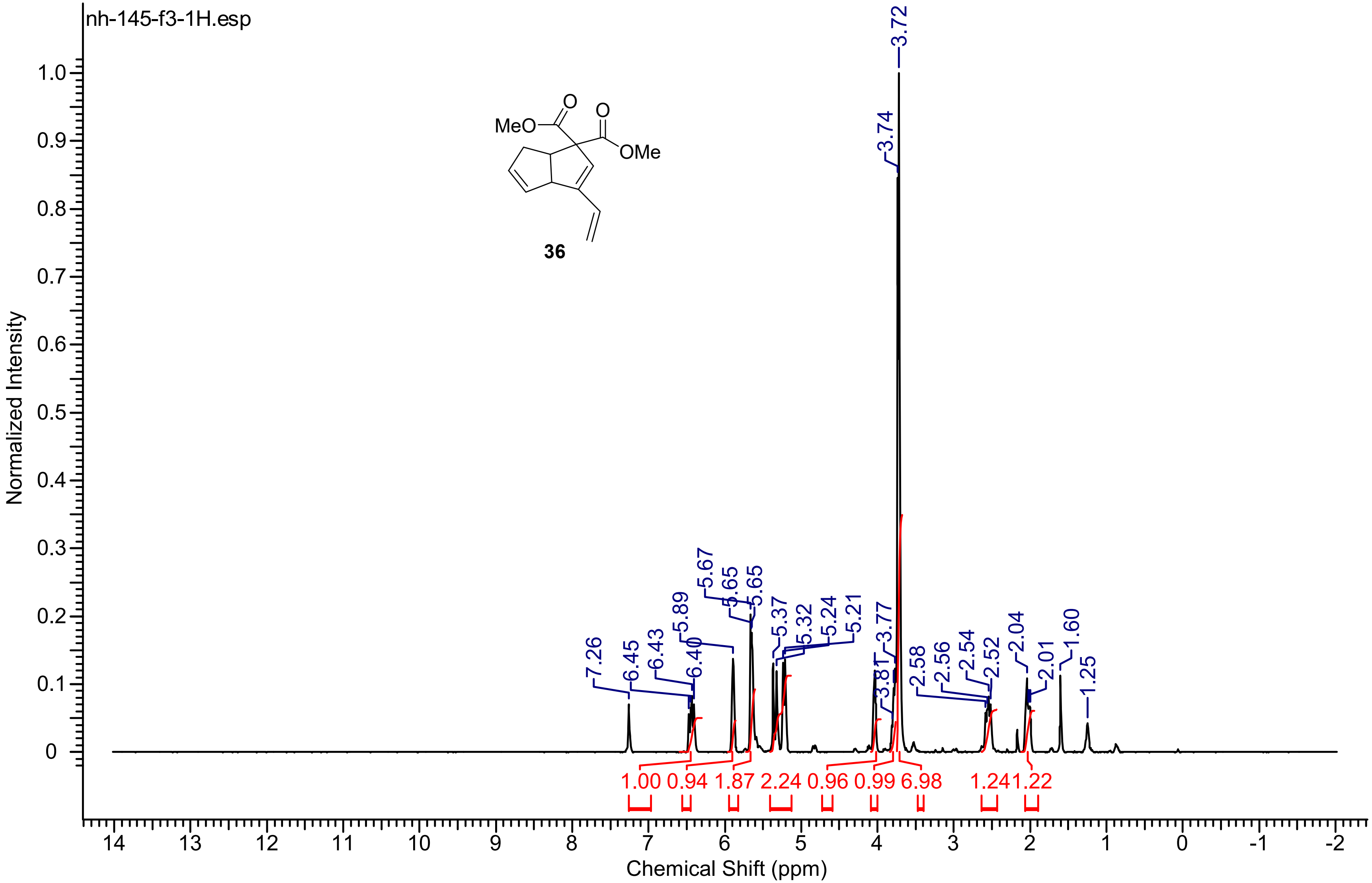
nh-145-f3-1H.esp
MeO
O
O
OMe
36
Normalized Intensity
Chemical Shift (ppm)
7.26
6.45
6.43
6.40
5.89
5.67
5.65
5.65
5.37
5.32
5.24
5.21
3.81
3.77
3.74
3.72
2.58
2.56
2.54
2.52
2.04
2.01
1.60
1.25
1.00
0.94
1.87
2.24
0.96
0.99
6.98
1.24
1.22

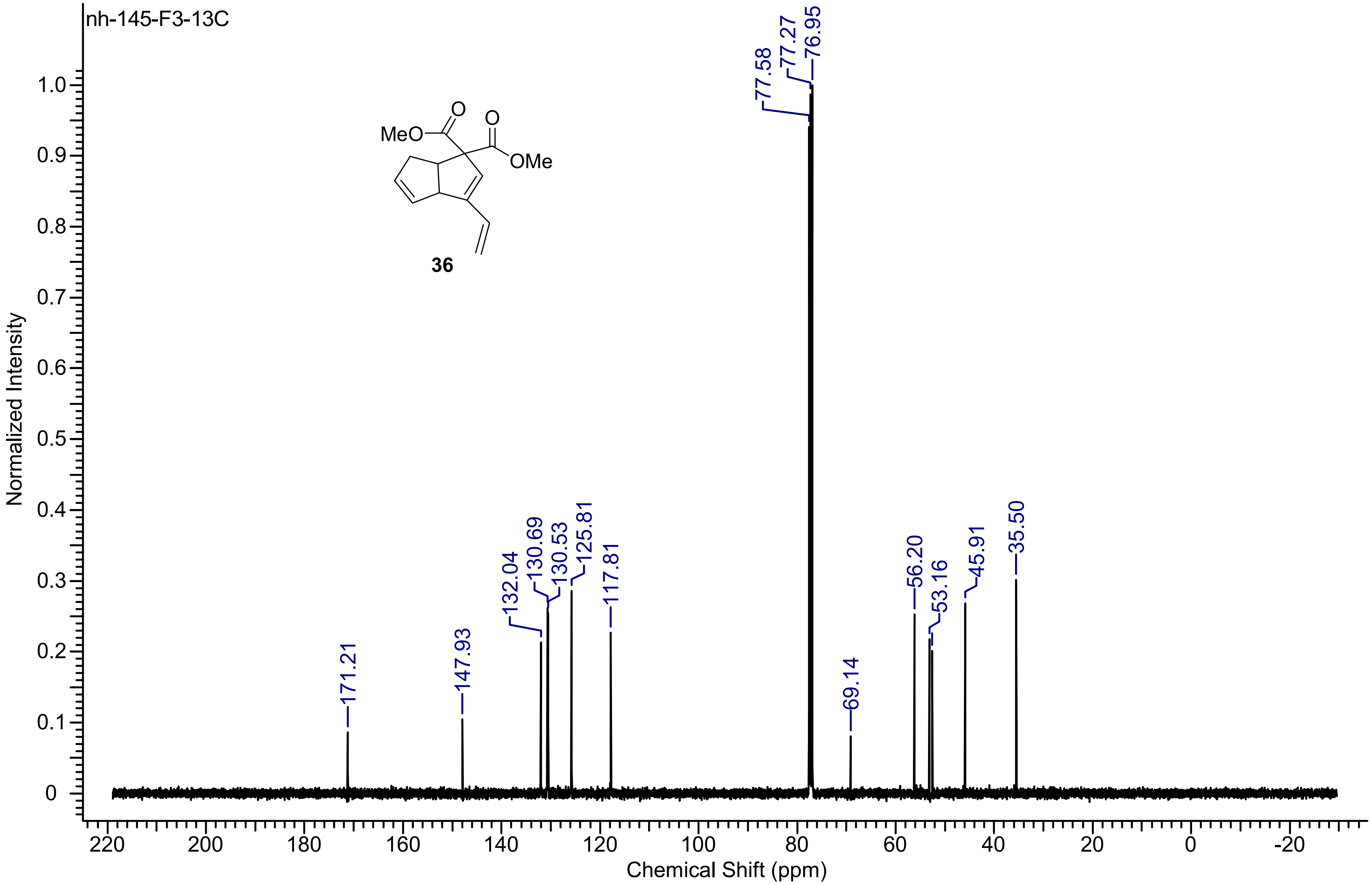

nh-145-F3-13C
MeO
O
O
OMe
36
171.21
147.93
132.04
130.69
130.53
125.81
117.81
77.58
77.27
76.95
69.14
56.20
53.16
45.91
35.50
Normalized Intensity
Chemical Shift (ppm)

HRMS ObservedΔ = 0.8 mDa
Acceptable = ± 1.4 mDa

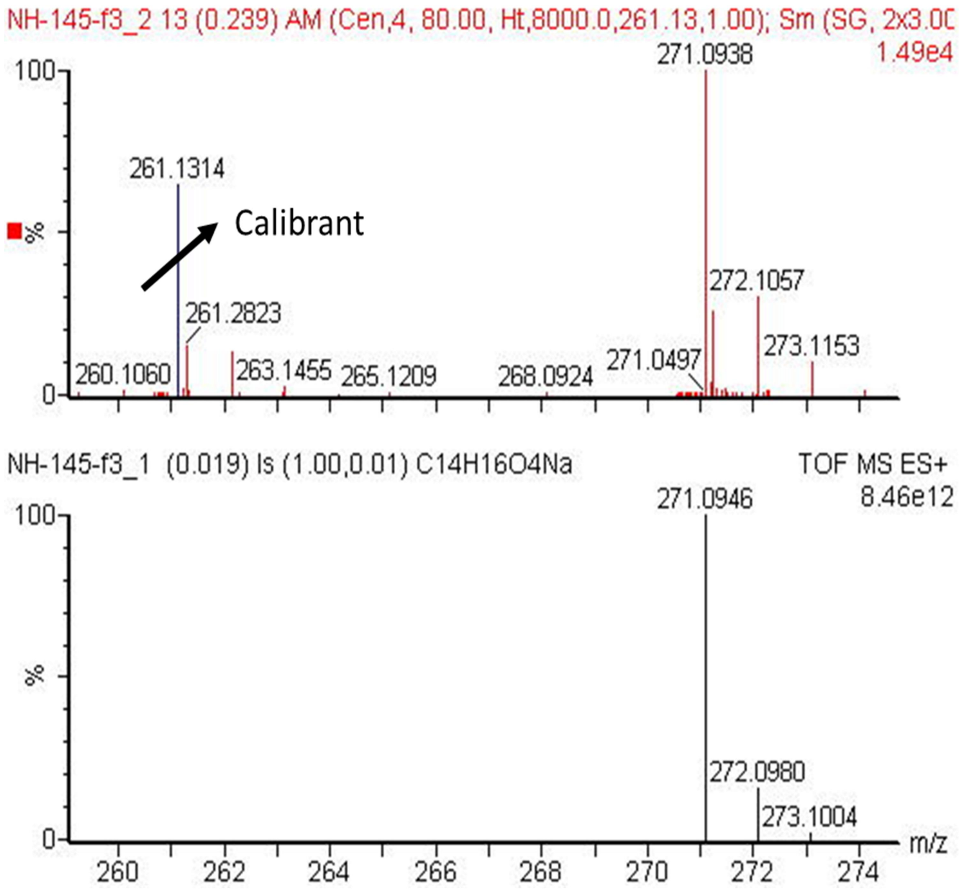

Theoretical =[M+Na]$^{+}$

LRMS [M+Na]$^{+}$ [2M+Na]$^{+}$

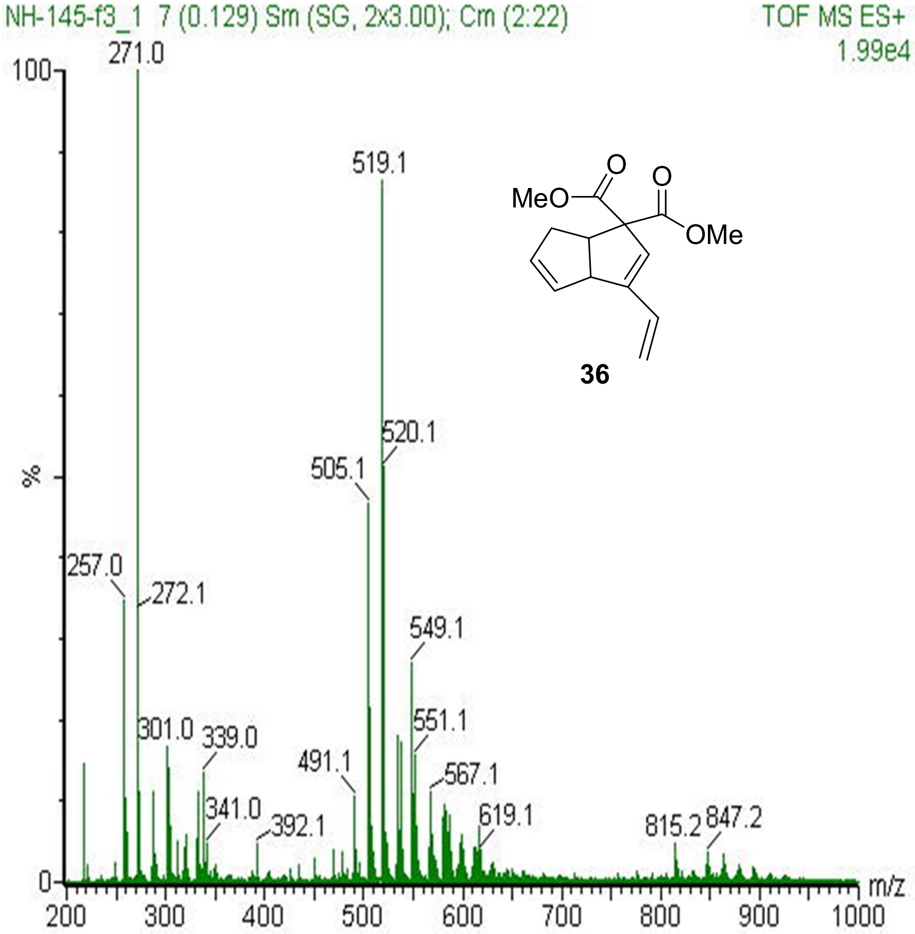

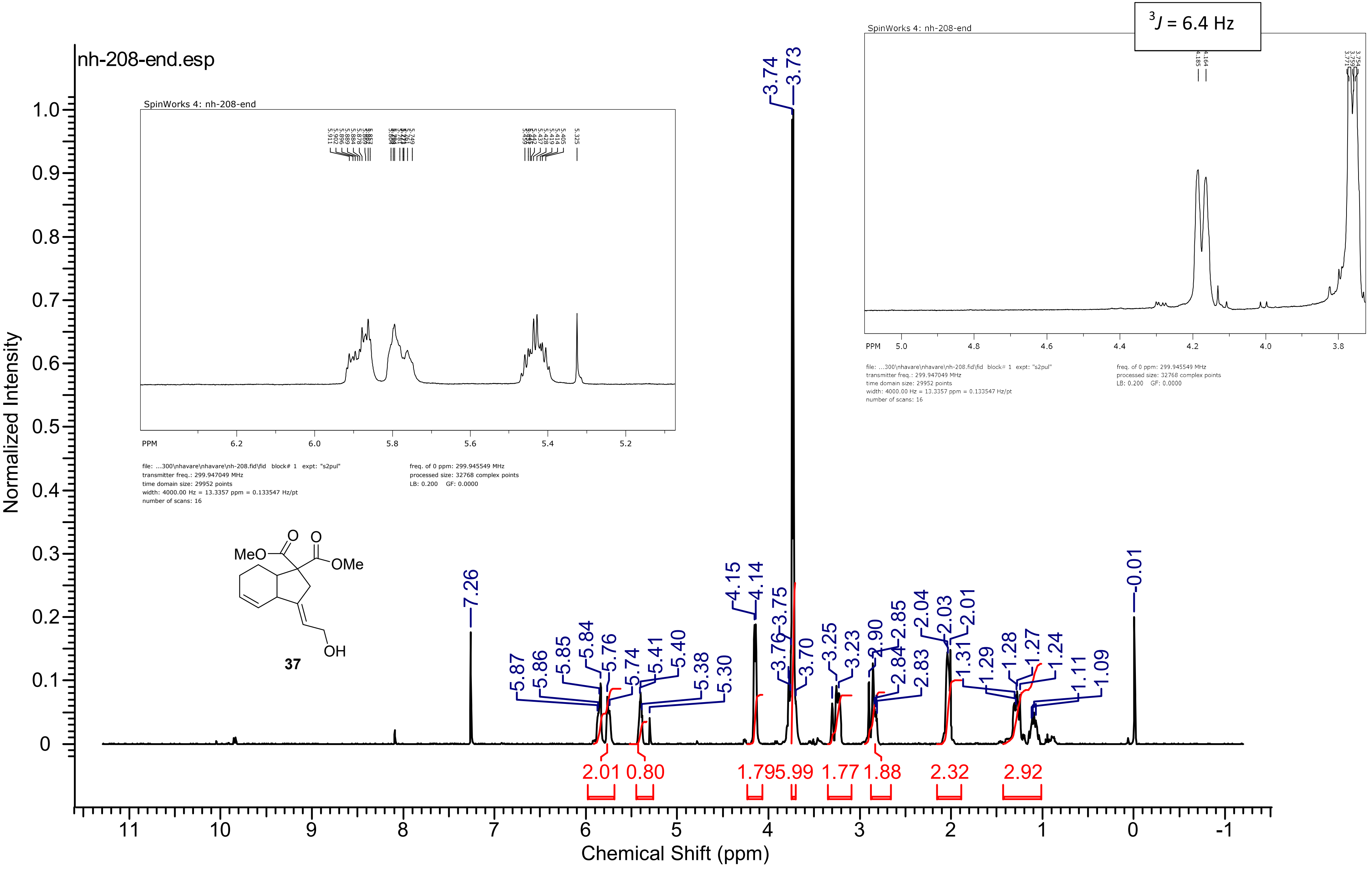

nh-208-end.esp
SpinWorks 4: nh-208-end
^3J = 6.4 Hz
MeO
O
O
OMe
OH
37
Normalized Intensity
Chemical Shift (ppm)
7.26
4.15
4.14
3.74
3.73
-0.01
2.01
0.80
1.79
5.99
1.77
1.88
2.32
2.92

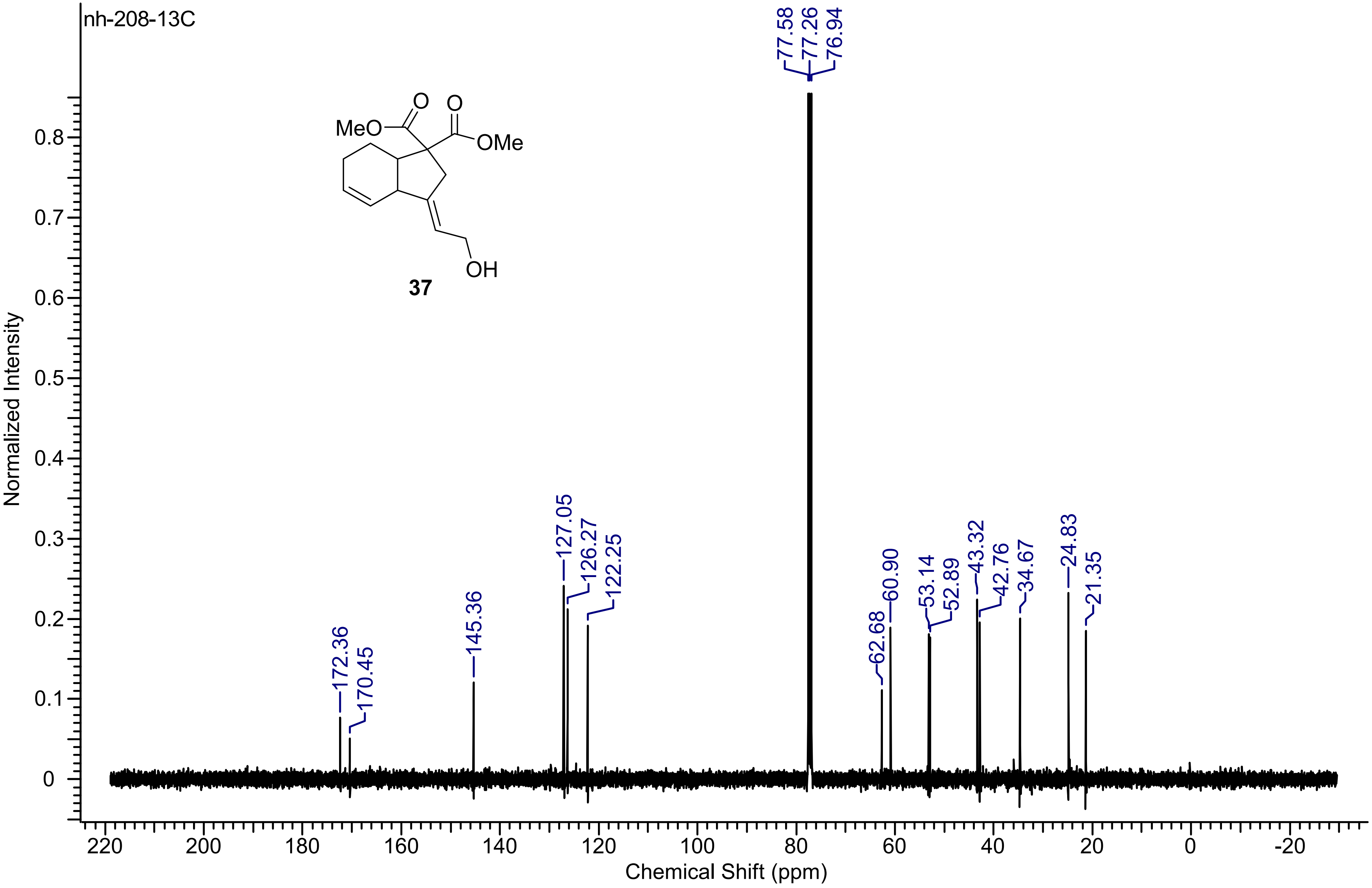
nh-208-13C
MeO
O
O
OMe
OH
37
77.58
77.26
76.94
172.36
170.45
145.36
127.05
126.27
122.25
62.68
60.90
53.14
52.89
43.32
42.76
34.67
24.83
21.35
Normalized Intensity
Chemical Shift (ppm)

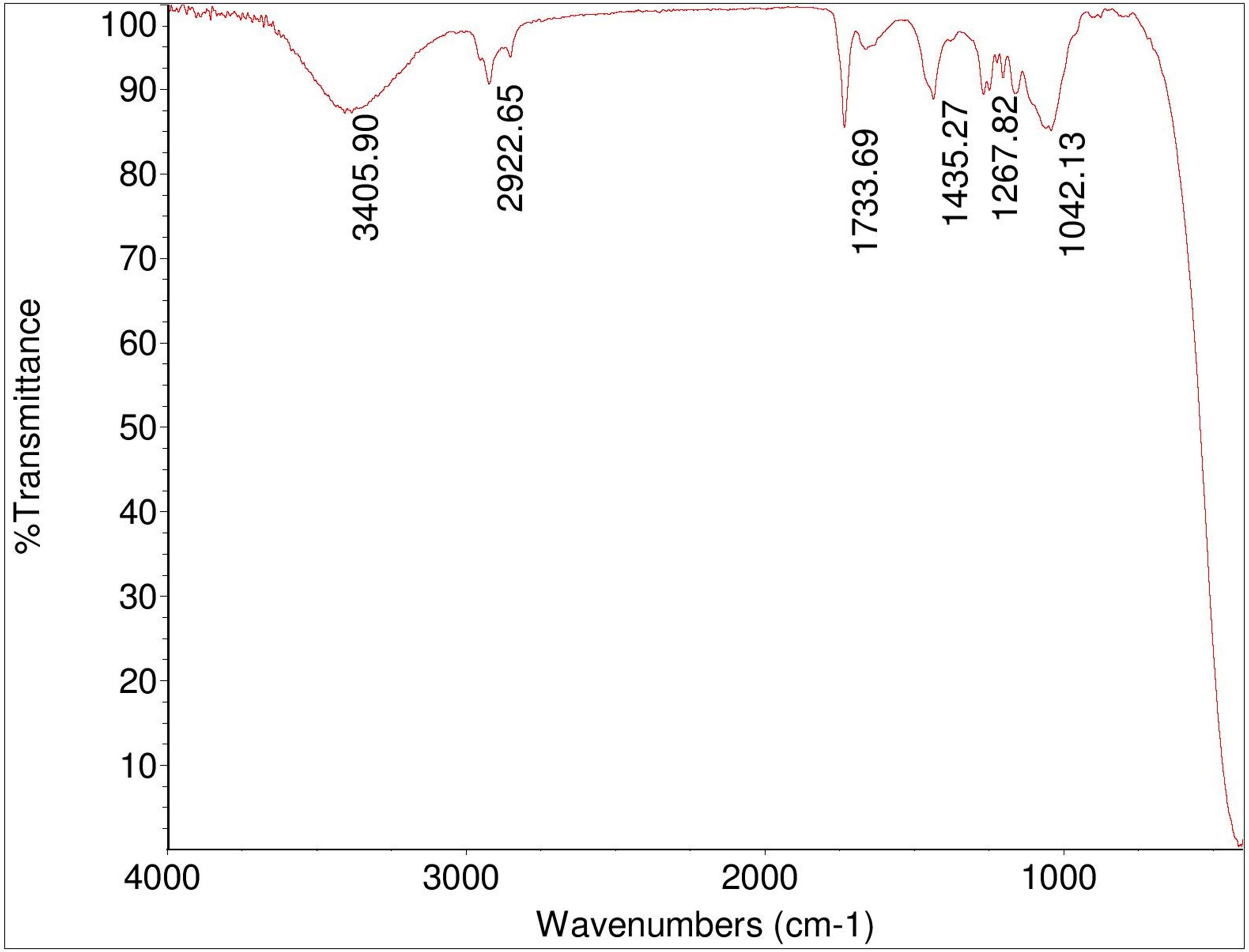

100
90
80
70
60
50
40
30
20
10
%Transmittance
3405.90
2922.65
1733.69
1435.27
1267.82
1042.13
4000
3000
2000
1000
Wavenumbers (cm-1)

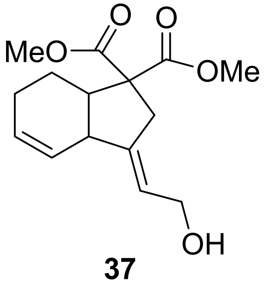

MeO
O
O
OMe
OH

37

LRMS [M+Na]$^+$

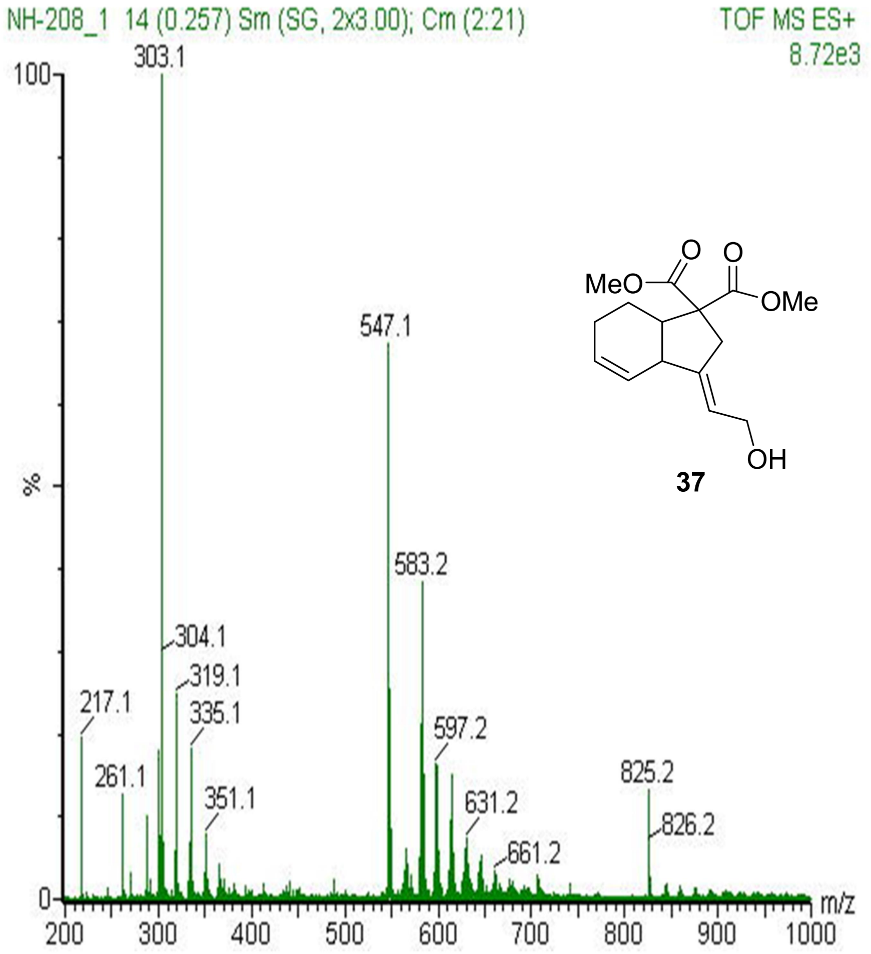

37

HRMS

Observed Δ = 0.2 mDa
Acceptable = ± 1.5 mDa

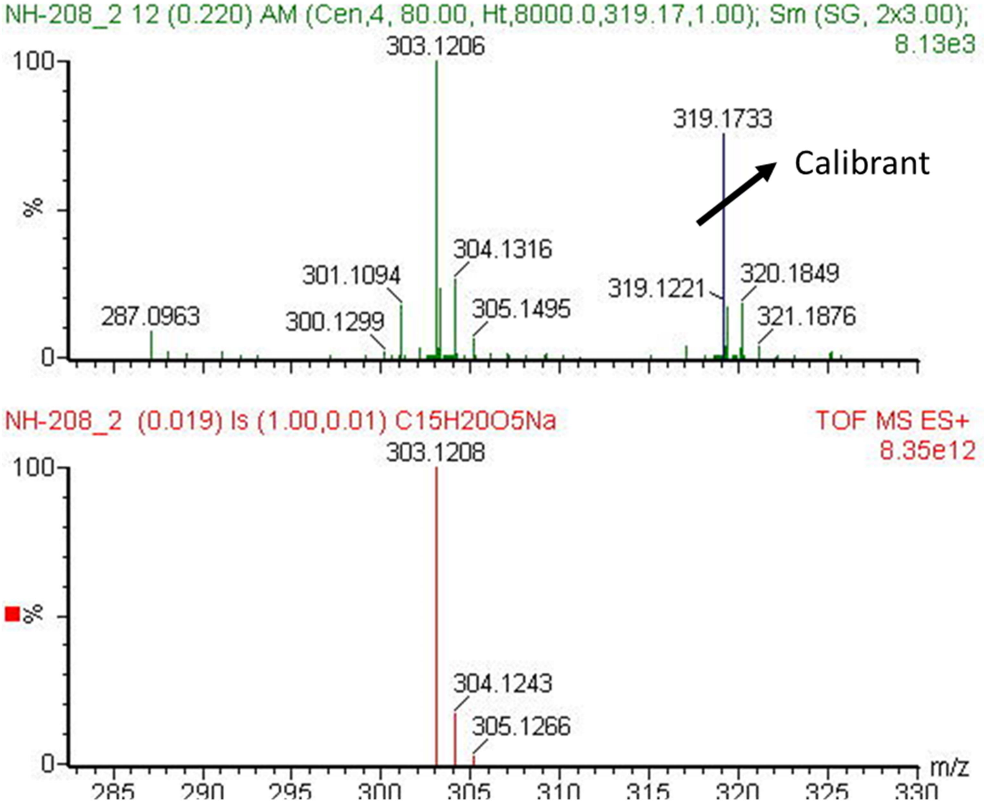

Theoretical =[M+Na]$^+$

SpinWorks 4: nh-335-b

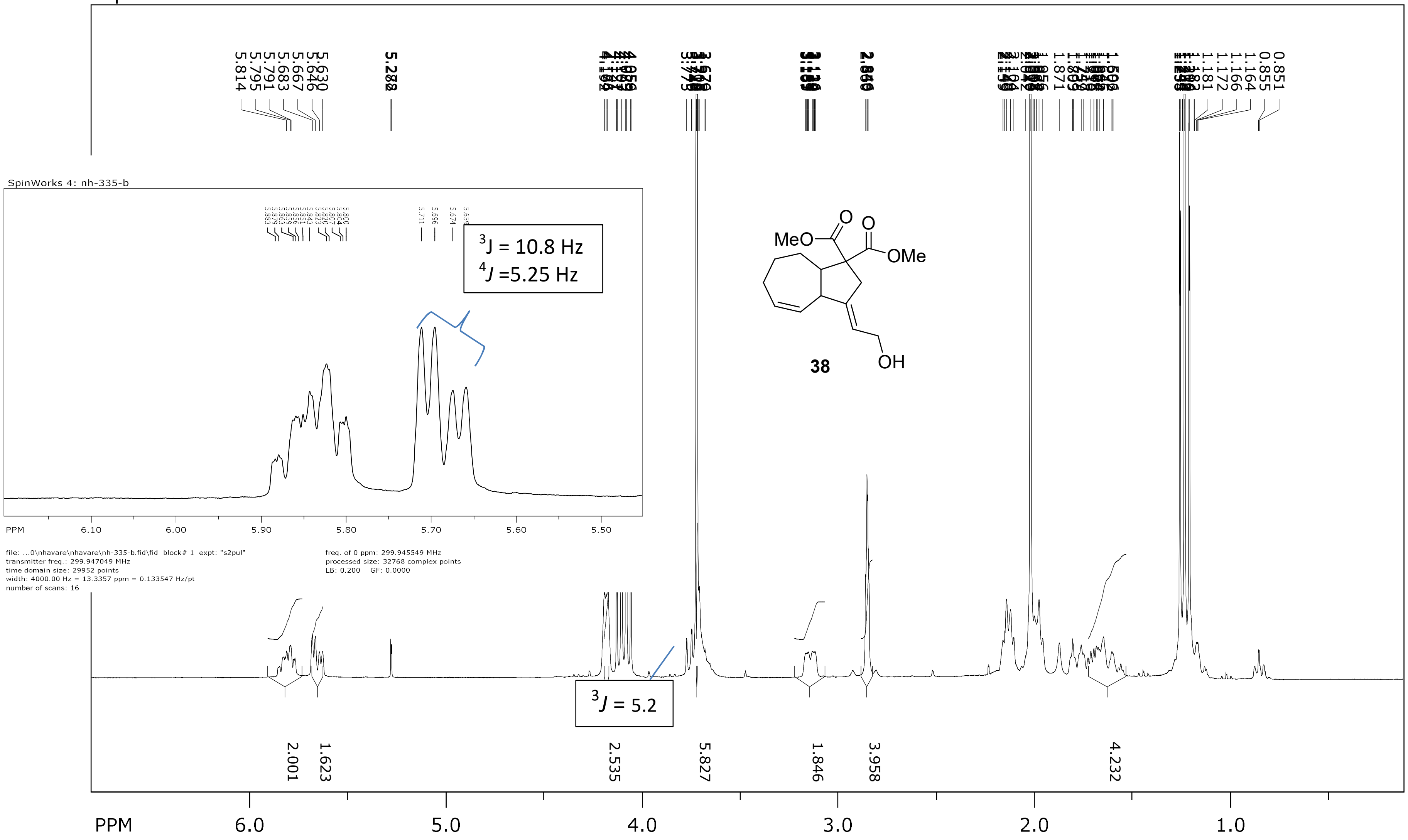

file: ...0\nhavare\nhavare\nh-335-b.fid\fid block# 1 expt: "s2pul"
transmitter freq.: 299.947049 MHz
time domain size: 29952 points
width: 4000.00 Hz = 13.3357 ppm = 0.133547 Hz/pt
number of scans: 16

freq. of 0 ppm: 299.945557 MHz
processed size: 32768 complex points
LB: 0.200 GF: 0.0000

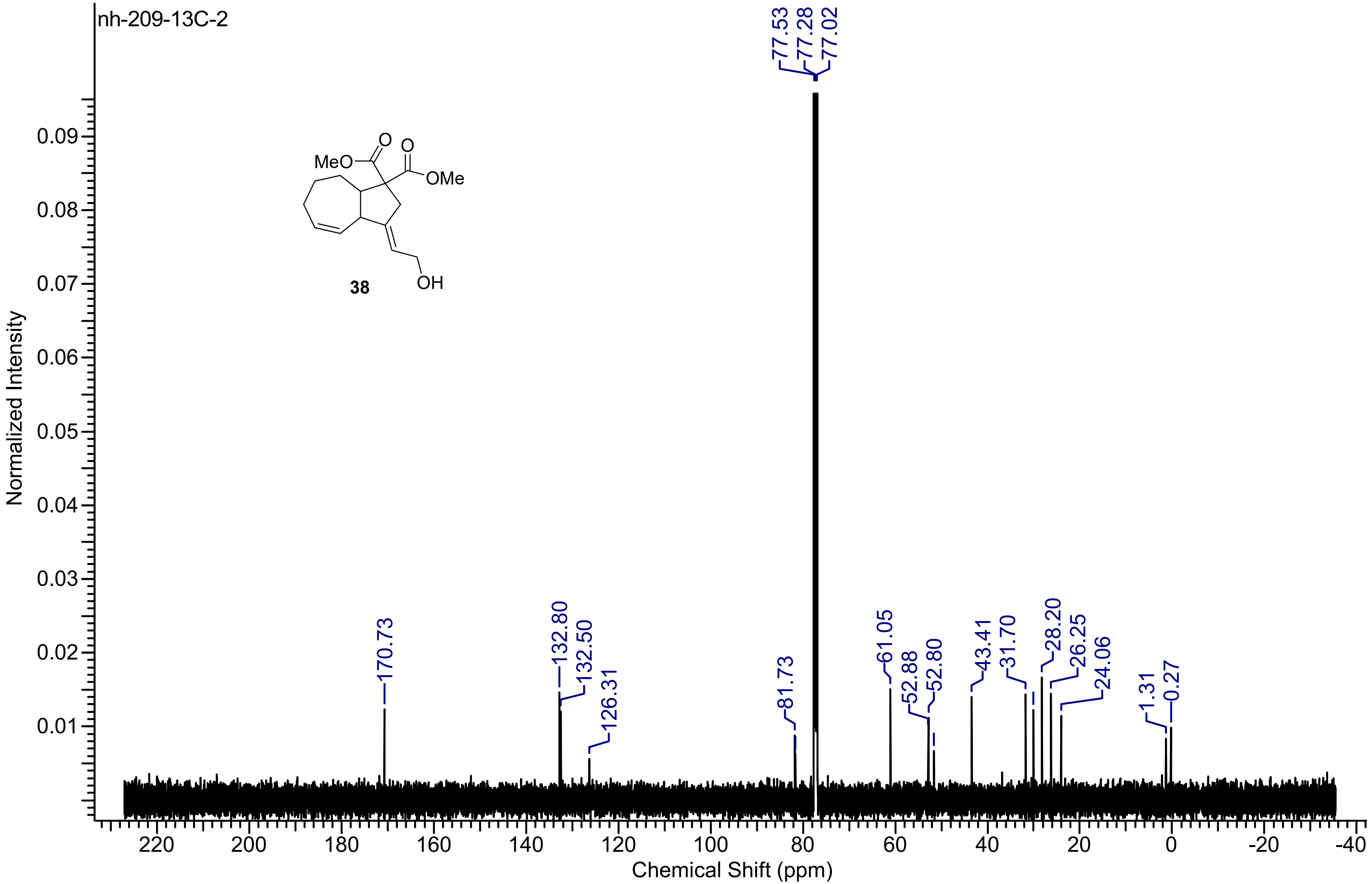

nh-209-13C-2
77.53
77.28
77.02
MeO
O
O
OMe
38
OH
170.73
132.80
132.50
126.31
81.73
61.05
52.88
52.80
43.41
31.70
28.20
26.25
24.06
1.31
0.27
Normalized Intensity
Chemical Shift (ppm)

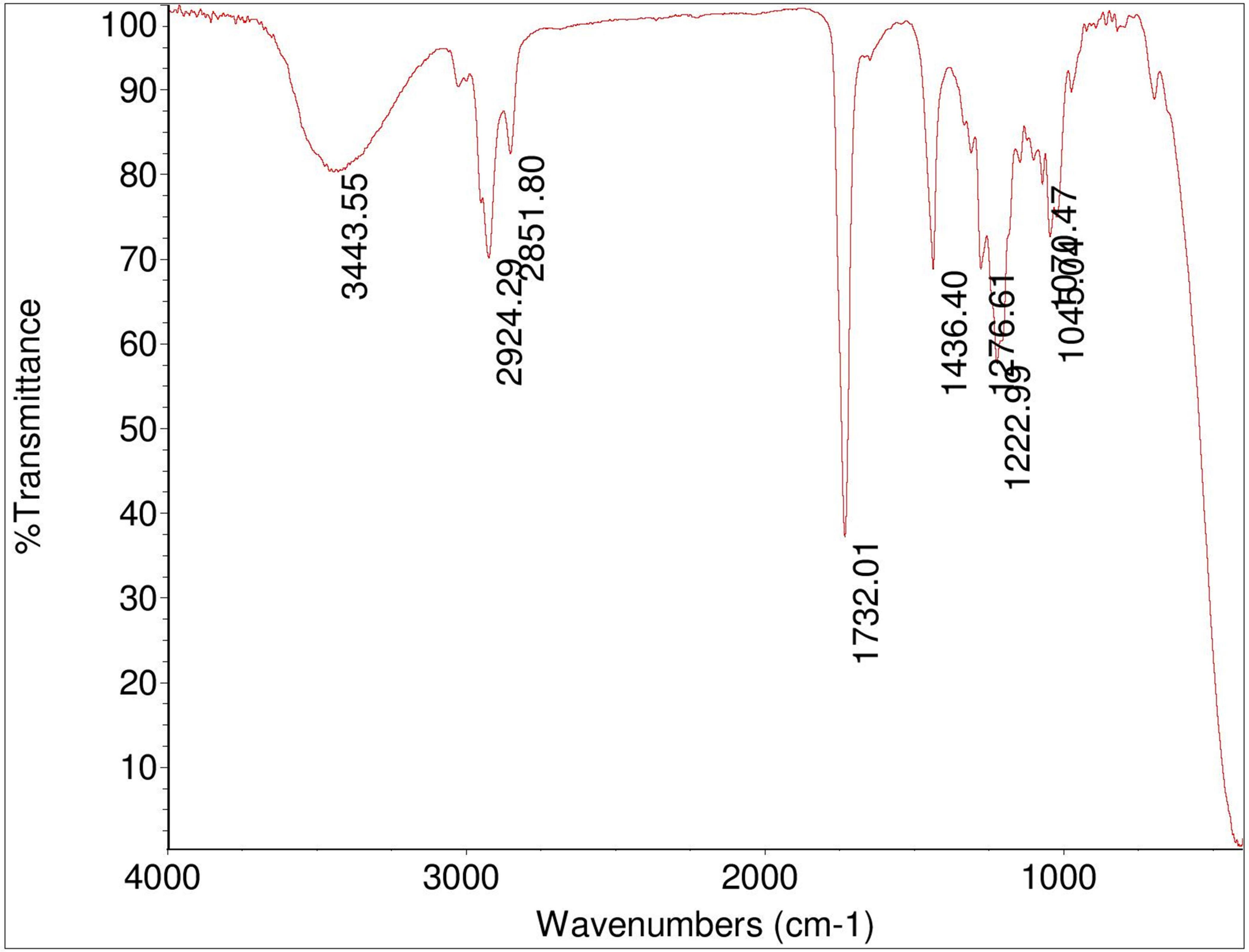

%Transmittance
Wavenumbers (cm-1)
3443.55
2924.29
2851.80
1732.01
1436.40
1276.61
1222.99
1070.47
1045.04

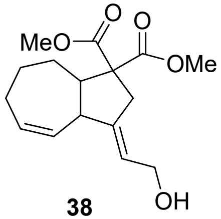

MeO
O
O
OMe
OH

38

LRMS [M+Na]$^+$

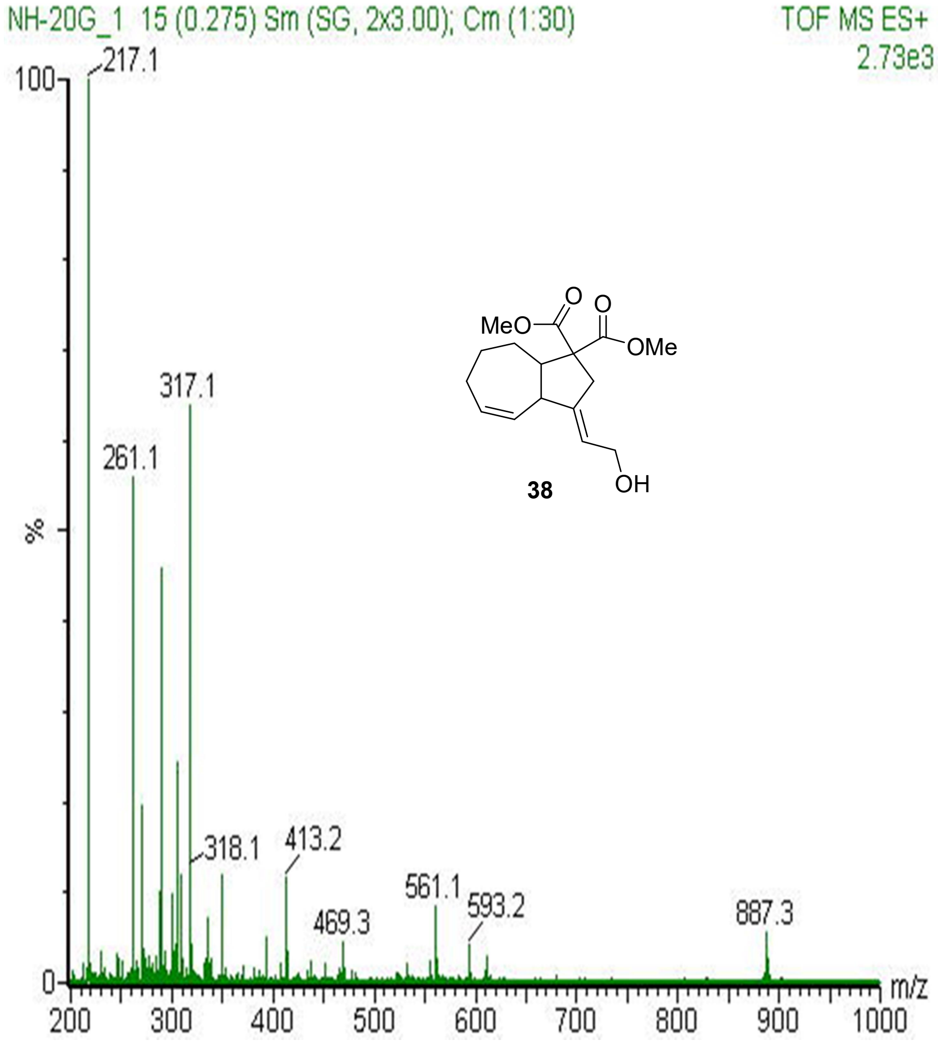

LRMS [M+Na]$^+$

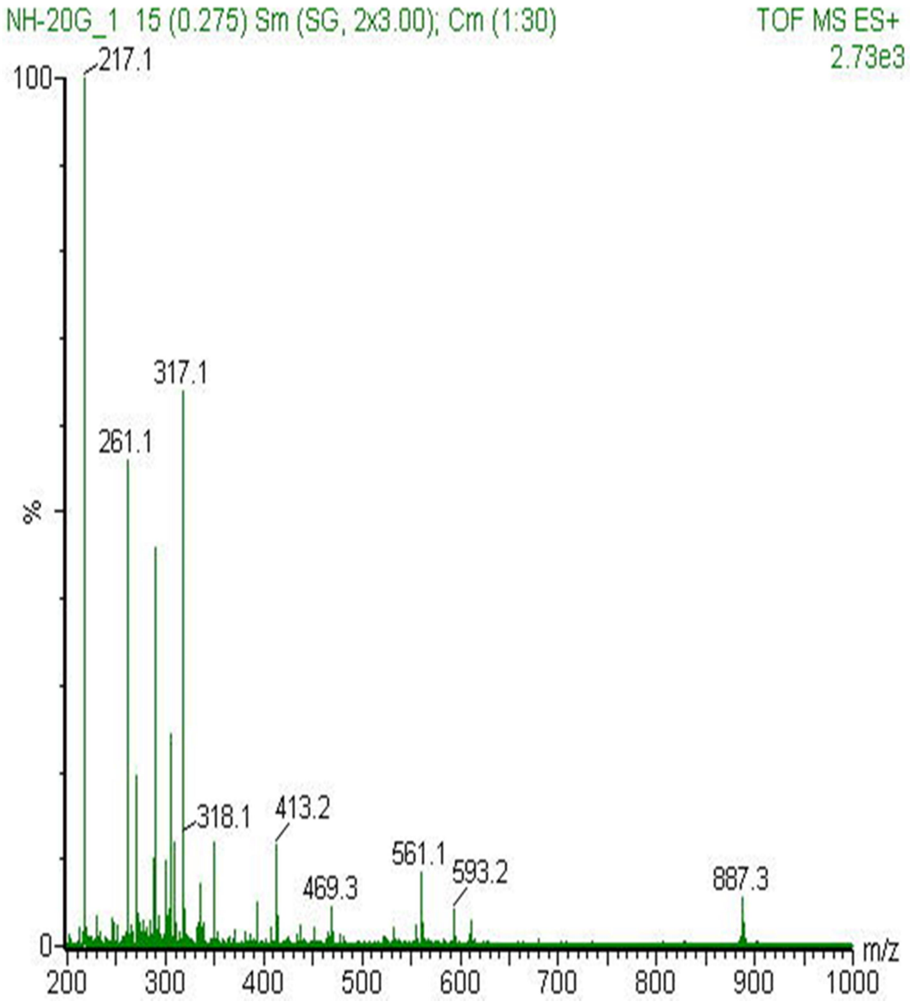

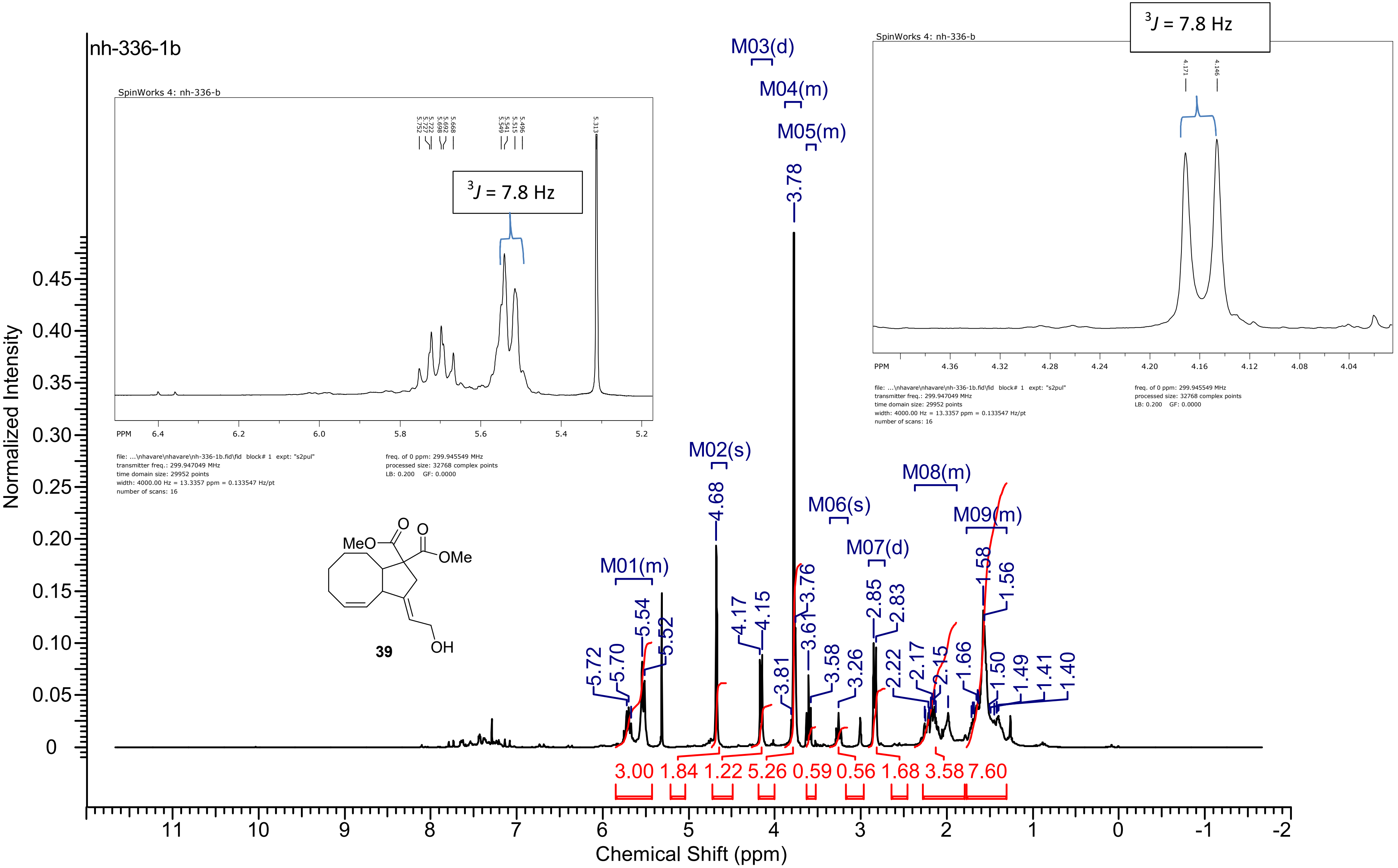
nh-336-1b
Normalized Intensity
Chemical Shift (ppm)
SpinWorks 4: nh-336-b
^3J = 7.8 Hz
MeO
OMe
OH
39
M01(m)
M02(s)
M03(d)
M04(m)
M05(m)
M06(s)
M07(d)
M08(m)
M09(m)
3.00 1.84 1.22 5.26 0.59 0.56 1.68 3.58 7.60

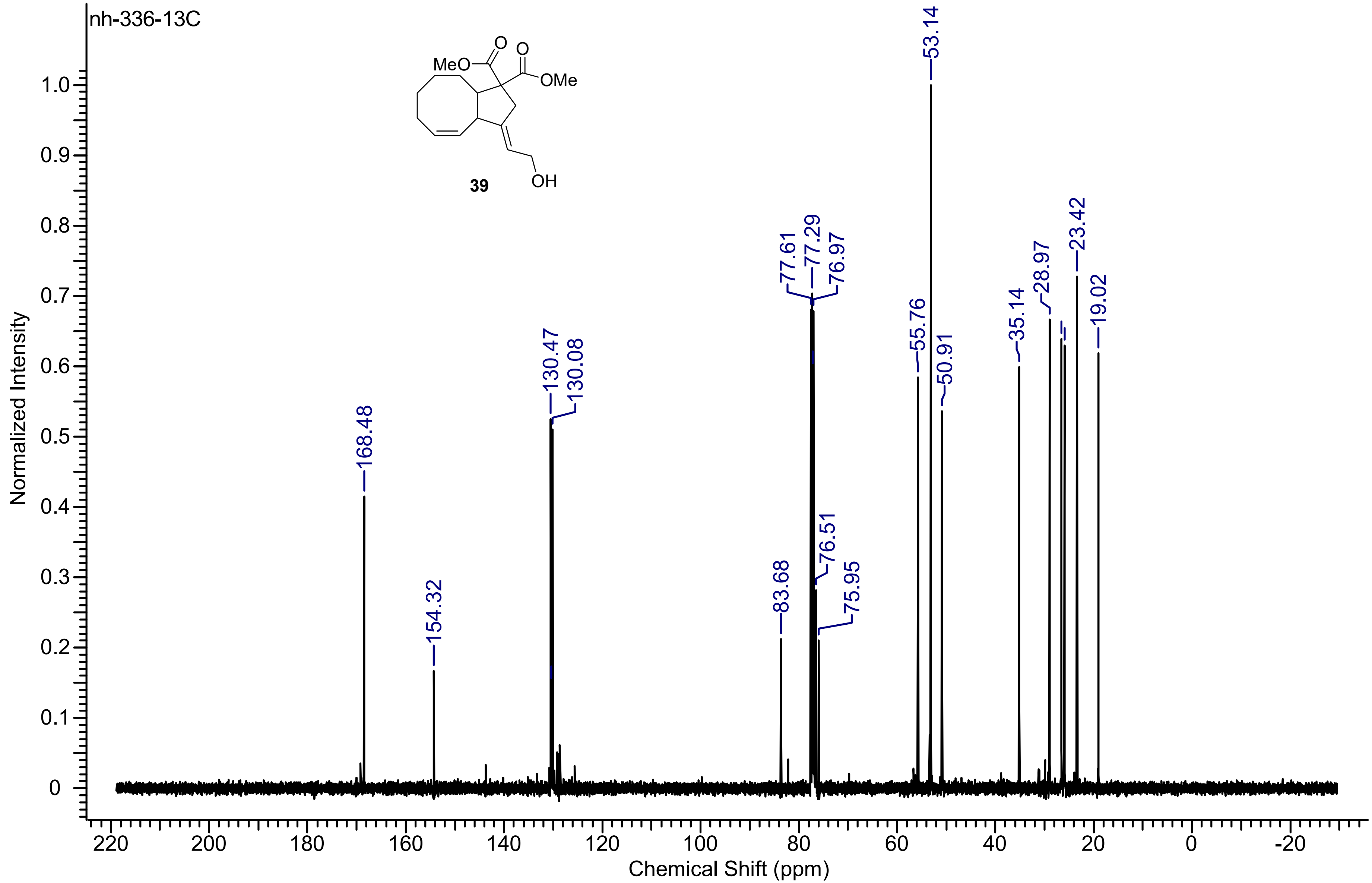
nh-336-13C
MeO
O
O
OMe
39
OH
168.48
154.32
130.47
130.08
83.68
77.61
77.29
76.97
76.51
75.95
55.76
53.14
50.91
35.14
28.97
23.42
19.02
Normalized Intensity
Chemical Shift (ppm)

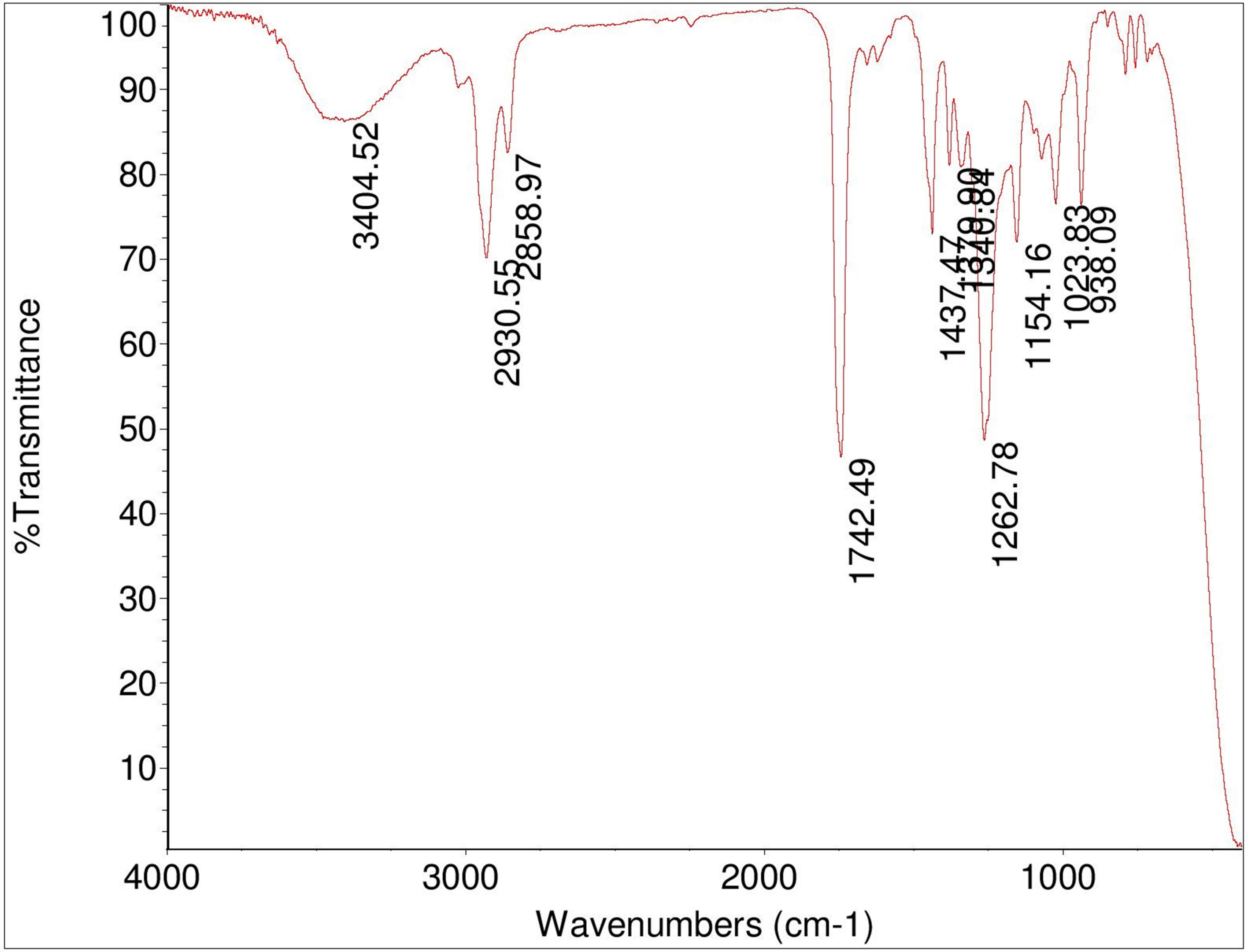

%Transmittance
Wavenumbers (cm-1)
100
90
80
70
60
50
40
30
20
10
4000
3000
2000
1000
3404.52
2930.55
2858.97
1742.49
1437.47
1379.80
1340.84
1262.78
1154.16
1023.83
938.09

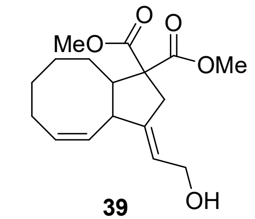

MeO
O
O
OMe
OH

39

Mass Spectrum SmartFormula and Mass List Report

Analysis Info

Analysis Name	D:\Data\HRAM\170216\data\HavareN_i6574_nh-336_L1_12_01_18731.d	Acquisition Date	2/28/2017 9:47:25 PM
Method	lc_nodiv_15mins_pos_50-1000.m	Operator	BDAL@DE
Sample Name	HavareN_i6574_nh-336_L1	Instrument / Ser#	micrOTOF-Q II 10292
Comment			

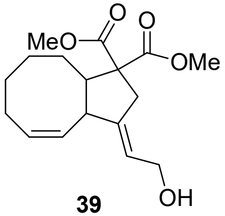

39

Acquisition Parameter

Source Type	ESI	Ion Polarity	Positive	Set Nebulizer	2.5 Bar
Focus	Not active	Set Capillary	4500 V	Set Dry Heater	200 °C
Scan Begin	50 m/z	Set End Plate Offset	-500 V	Set Dry Gas	8.0 l/min
Scan End	1000 m/z	Set Collision Cell RF	60.0 Vpp	Set Divert Valve	Source

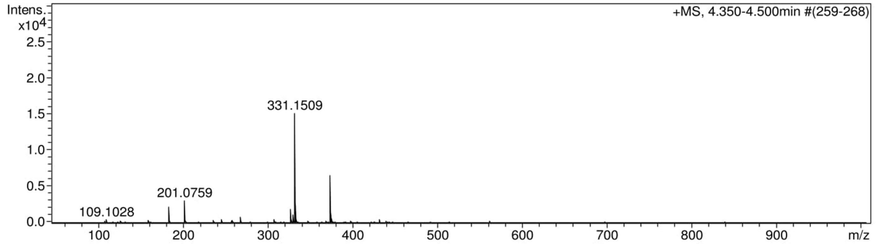

Meas. m/z	#	Formula	m/z	mSigma	err [ppm]	Score	err [mDa]	rdb	e⁻ Conf	N-Rule
331.1509	1	C 17 H 24 Na O 5	331.1516	6.3	2.1	89.34	0.7	5.5	even	ok
	2	C 15 H 19 N 6 O 3	331.1513	7.8	1.3	100.00	0.4	9.5	even	ok
	3	C 14 H 23 N 2 O 7	331.1500	8.9	-2.7	76.88	-0.9	4.5	even	ok

#	m/z	I	I %	Area	Res.
1	107.0866	150	1.0	3	5013
2	109.1028	379	2.5	9	4871
3	126.1285	163	1.1	4	5339
4	158.9642	349	2.3	10	5866
5	183.0650	2182	14.4	69	6161
6	184.0695	201	1.3	7	6083
7	201.0759	3067	20.3	107	6056
8	202.0782	328	2.2	11	6698
9	235.1121	271	1.8	11	6443
10	245.0665	405	2.7	17	6625
11	257.0939	332	2.2	13	7321
12	267.0479	725	4.8	32	6722
13	307.1676	410	2.7	18	7796
14	309.1692	143	0.9	7	6709
15	326.1950	1886	12.5	88	7472
16	327.1994	418	2.8	20	7652
17	329.1445	1020	6.7	54	6955
18	330.1479	232	1.5	12	7050
19	331.1509	15140	100.0	705	7652
20	332.1542	2628	17.4	127	7599
21	333.1544	456	3.0	22	8097
22	347.1236	231	1.5	11	7871
23	368.1677	223	1.5	11	8246
24	373.1246	6511	43.0	334	7925
25	374.1280	1299	8.6	67	7913
26	375.1360	553	3.7	29	8021
27	397.1456	164	1.1	10	7513
28	430.9123	469	3.1	25	8505
29	439.1189	187	1.2	12	8040

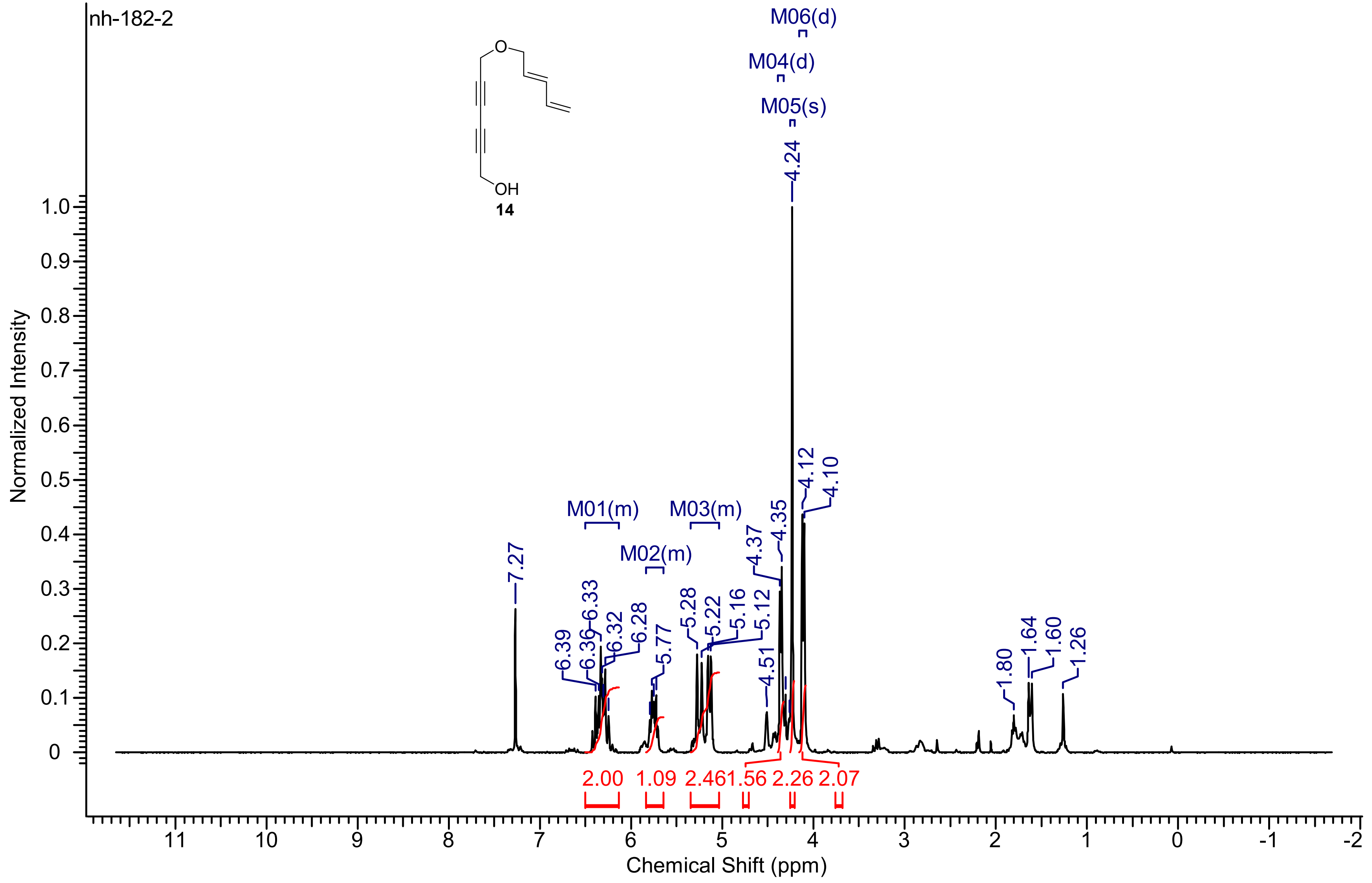
nh-182-2
OH
14
M06(d)
M04(d)
M05(s)
M01(m)
M02(m)
M03(m)
7.27
6.39
6.36
6.33
6.32
6.28
5.77
5.28
5.22
5.16
5.12
4.51
4.37
4.35
4.24
4.12
4.10
1.80
1.64
1.60
1.26
2.00
1.09
2.46
1.56
2.26
2.07
Normalized Intensity
Chemical Shift (ppm)

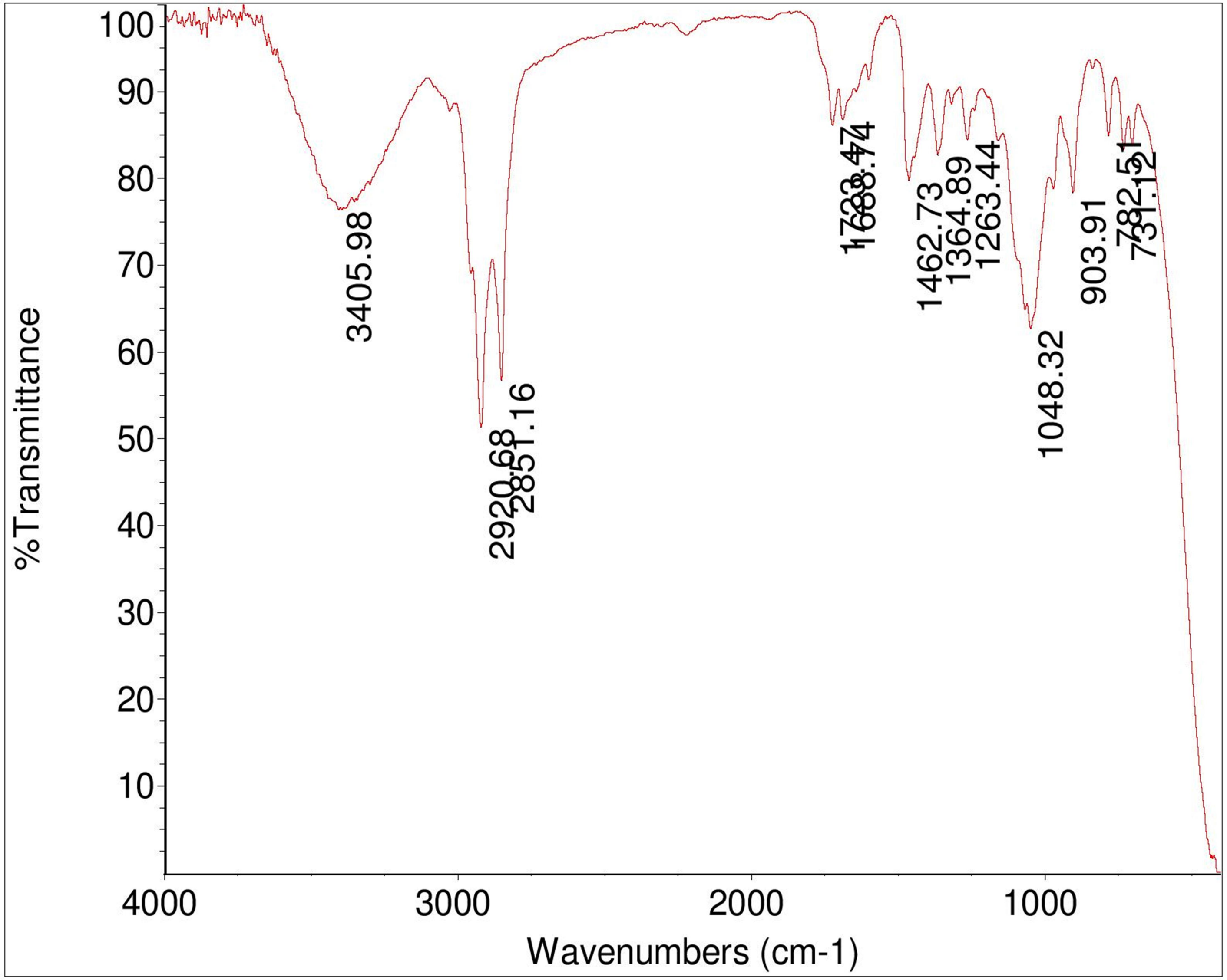
%Transmittance
Wavenumbers (cm-1)
100
90
80
70
60
50
40
30
20
10
4000
3000
2000
1000
3405.98
2920.68
2851.16
1733.47
1688.74
1462.73
1364.89
1263.44
1048.32
903.91
782.51
731.12

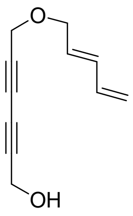
O
OH
14

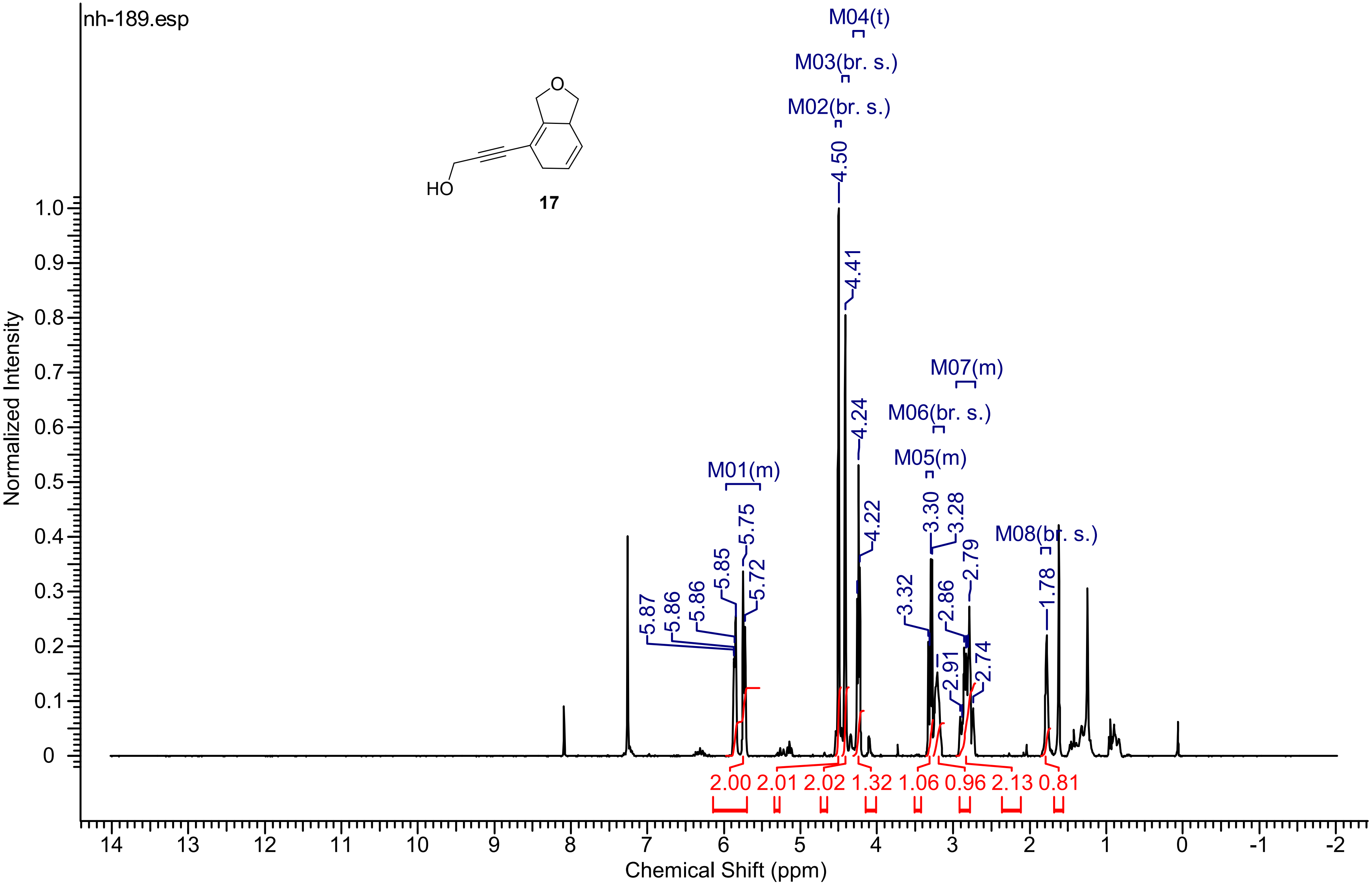
nh-189.esp
HO
17
M01(m)
M02(br. s.)
M03(br. s.)
M04(t)
M05(m)
M06(br. s.)
M07(m)
M08(br. s.)
5.87
5.86
5.86
5.85
5.75
5.72
4.50
4.41
4.24
4.22
3.32
3.30
3.28
2.91
2.86
2.79
2.74
1.78
2.00 2.01 2.02 1.32 1.06 0.96 2.13 0.81
Normalized Intensity
Chemical Shift (ppm)

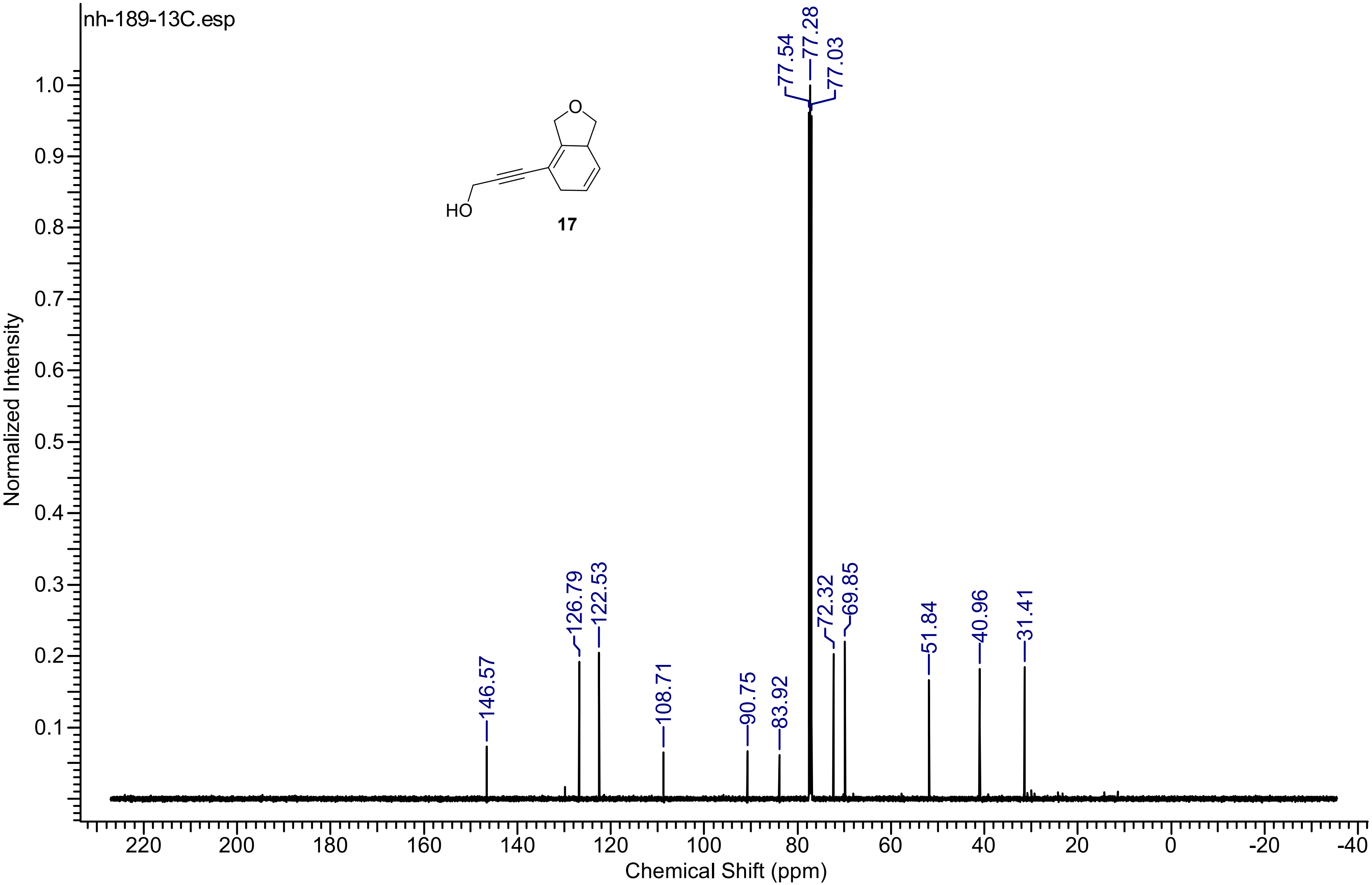

nh-189-13C.esp
HO
O
17
77.54
77.28
77.03
146.57
126.79
122.53
108.71
90.75
83.92
72.32
69.85
51.84
40.96
31.41
Normalized Intensity
1.0
0.9
0.8
0.7
0.6
0.5
0.4
0.3
0.2
0.1
220
200
180
160
140
120
100
80
60
40
20
0
-20
-40
Chemical Shift (ppm)

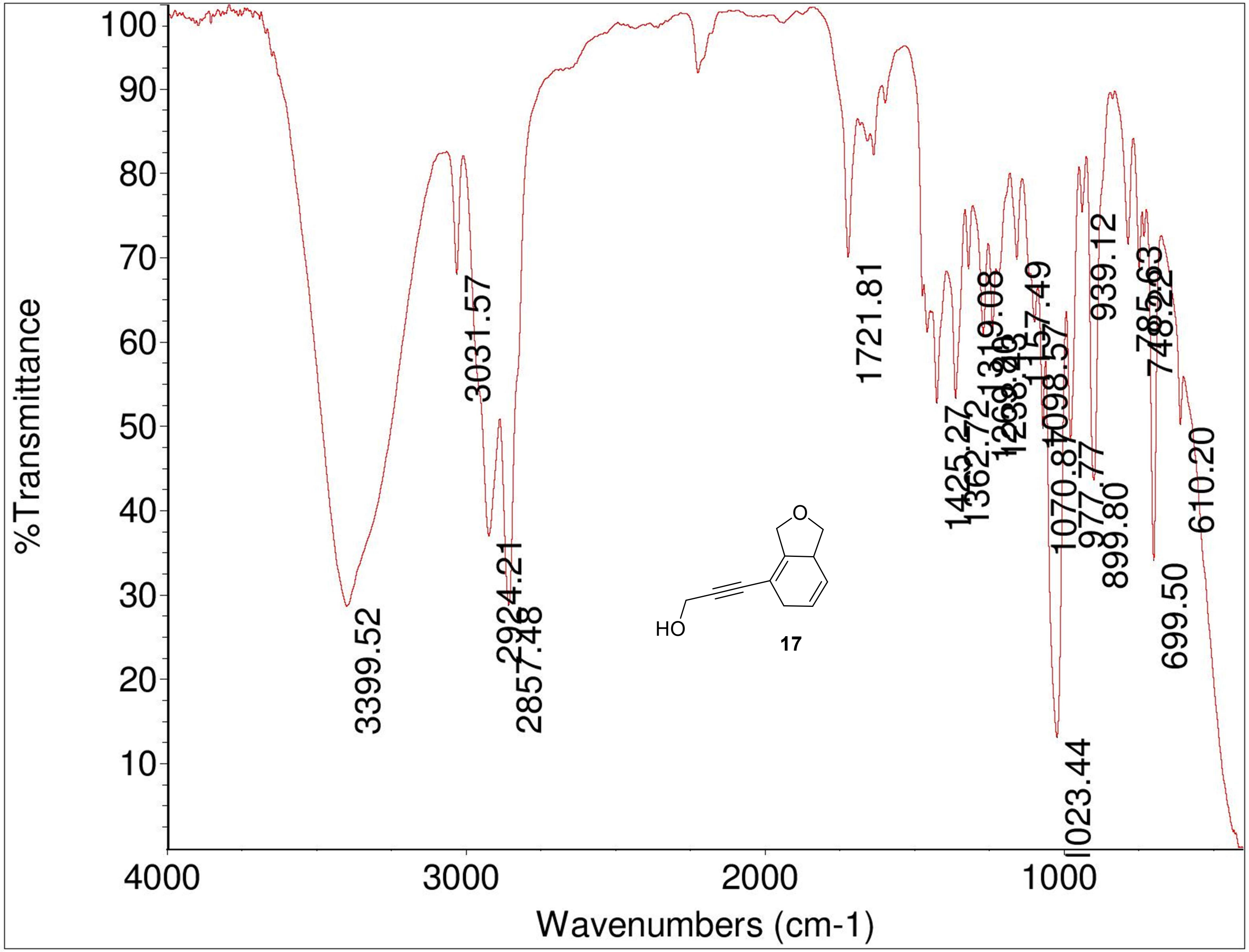

%Transmittance
Wavenumbers (cm-1)
100
90
80
70
60
50
40
30
20
10
4000
3000
2000
1000
3399.52
3031.57
2924.21
2857.48
1721.81
1425.27
1362.72
1319.08
1157.49
1098.57
1070.81
1023.44
977.77
939.12
899.80
785.63
748.22
699.50
610.20
O
HO
17

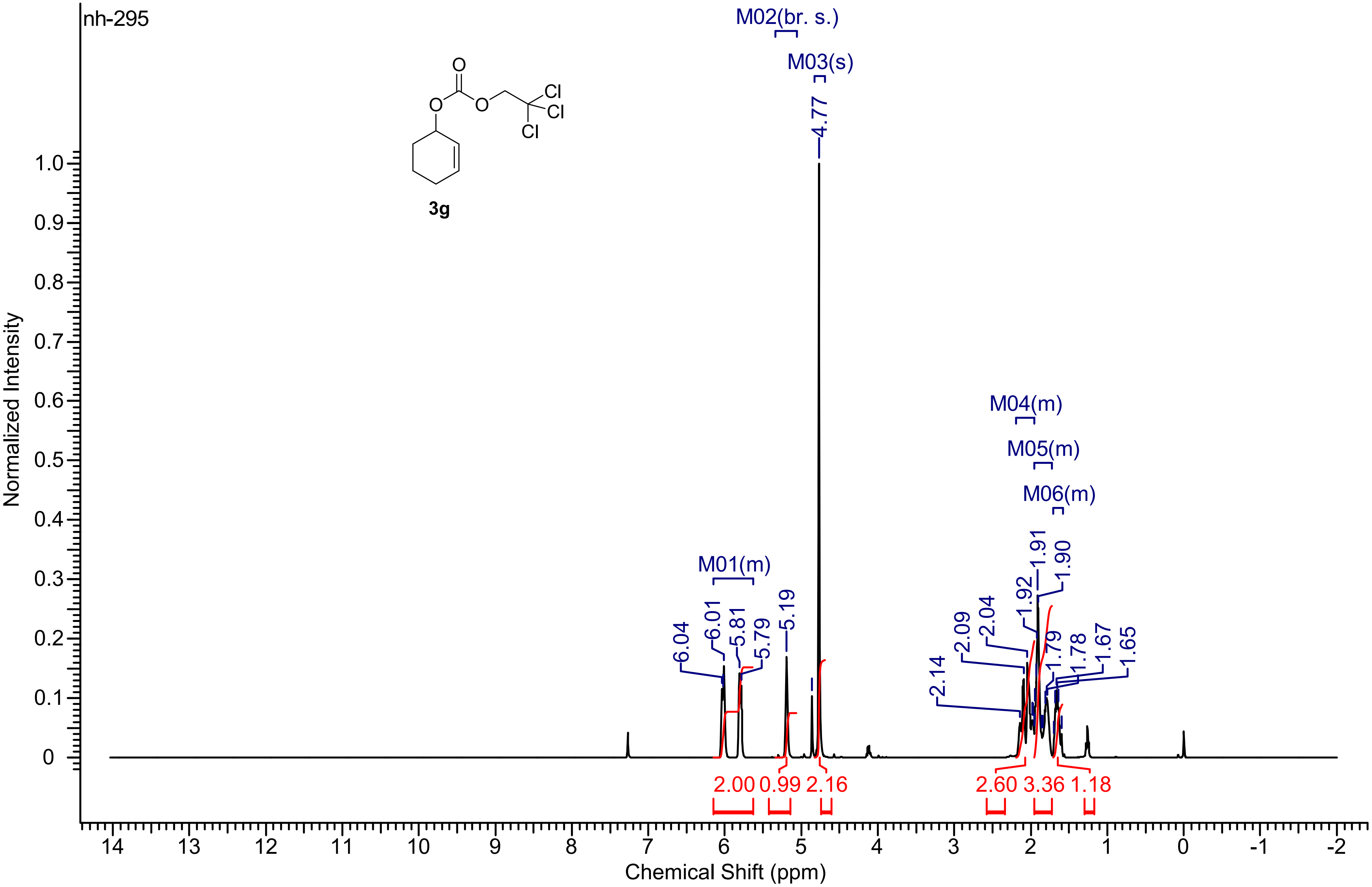

nh-295
3g
M01(m)
M02(br. s.)
M03(s)
M04(m)
M05(m)
M06(m)
6.04
6.01
5.81
5.79
5.19
4.77
2.14
2.09
2.04
1.92
1.91
1.90
1.79
1.78
1.67
1.65
2.00
0.99
2.16
2.60
3.36
1.18
Normalized Intensity
Chemical Shift (ppm)

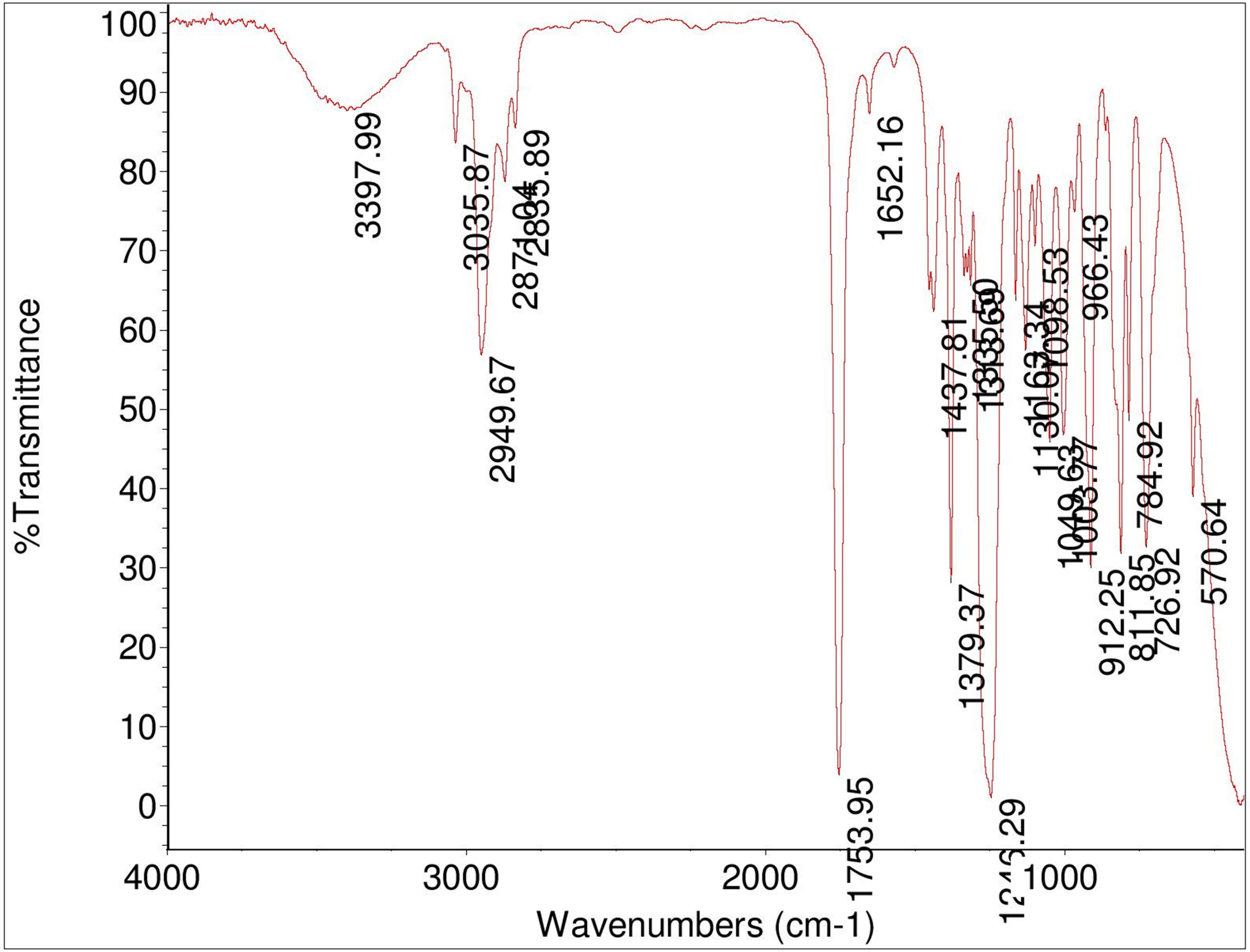
%Transmittance
Wavenumbers (cm-1)
100
90
80
70
60
50
40
30
20
10
0
4000
3000
2000
1000
3397.99
3035.87
2949.67
1652.16
1753.95
1437.81
1379.37
1098.53
1049.63
1003.77
966.43
912.25
811.85
784.92
726.92
570.64

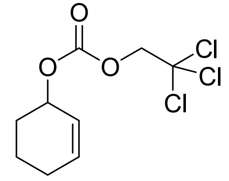
O
O
O
Cl
Cl
Cl

3g

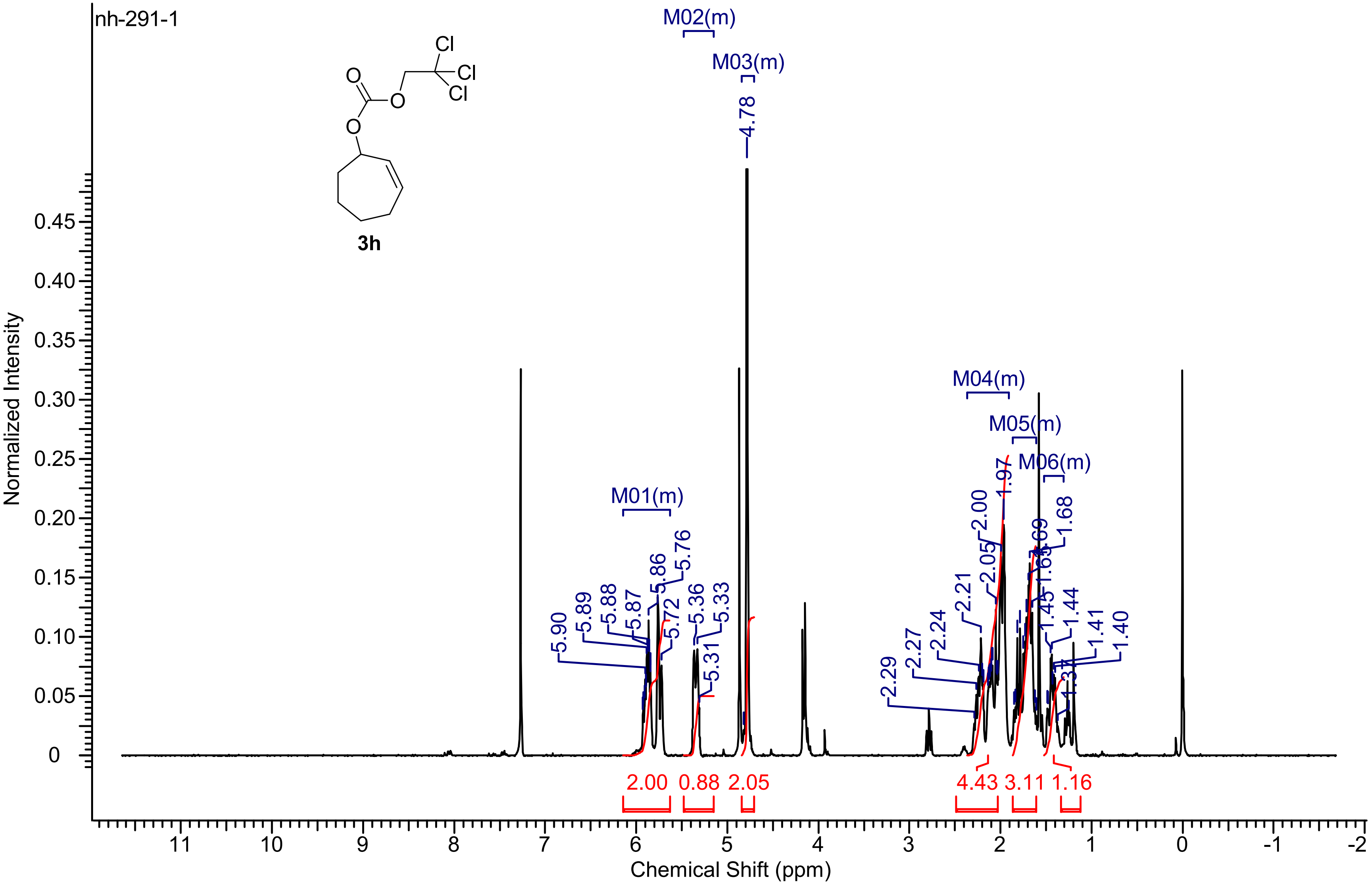
nh-291-1
O
O
O
Cl
Cl
Cl
3h
M01(m)
M02(m)
M03(m)
M04(m)
M05(m)
M06(m)
4.78
5.90
5.89
5.88
5.87
5.86
5.76
5.72
5.36
5.33
5.31
2.29
2.27
2.24
2.21
2.05
2.00
1.97
1.69
1.68
1.65
1.45
1.44
1.41
1.40
2.00
0.88
2.05
4.43
3.11
1.16
Normalized Intensity
Chemical Shift (ppm)

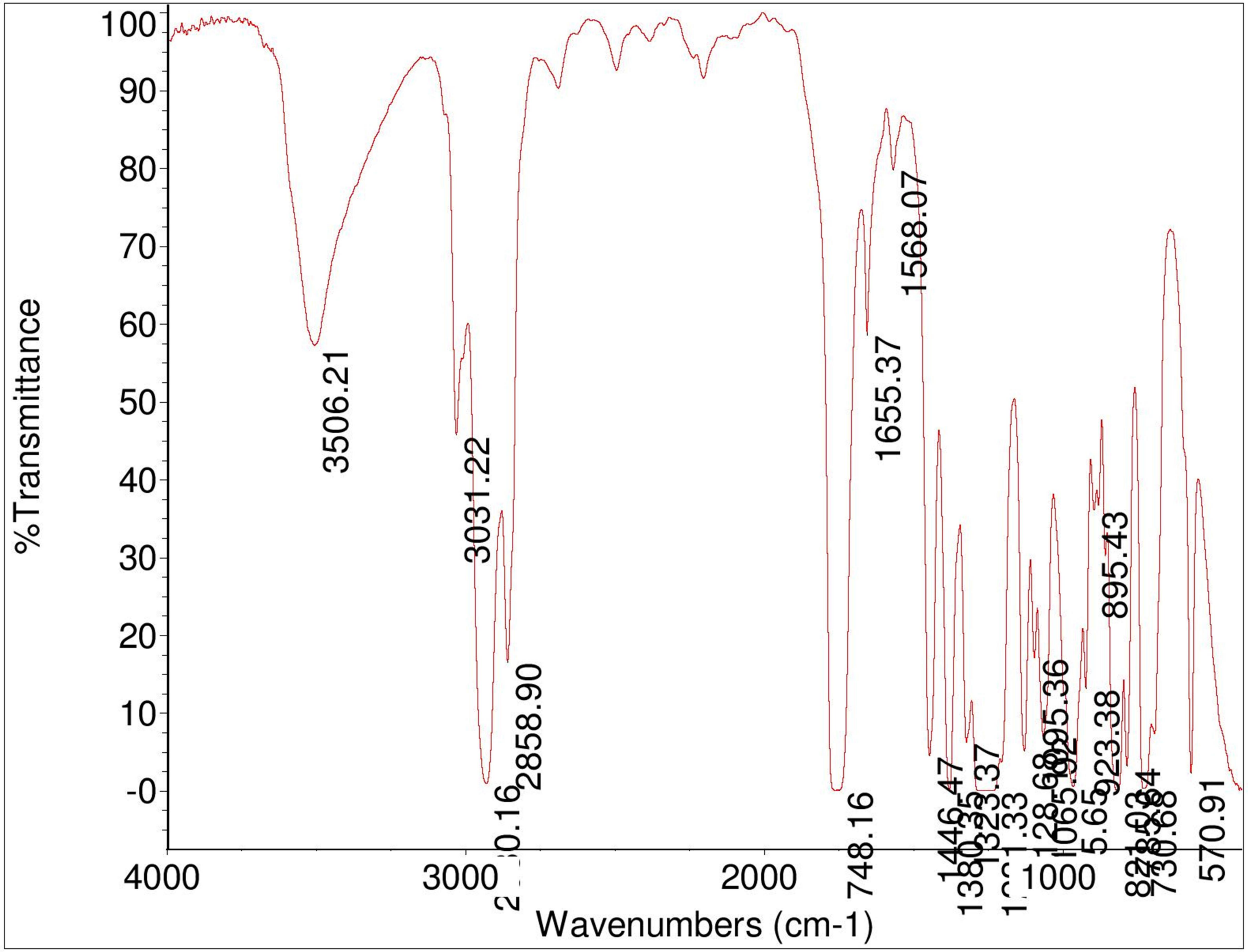

%Transmittance
100
90
80
70
60
50
40
30
20
10
-0
4000
3000
2000
1000
Wavenumbers (cm-1)
3506.21
3031.22
2858.90
748.16
1655.37
1568.07
1446.47
895.43
923.38
570.91

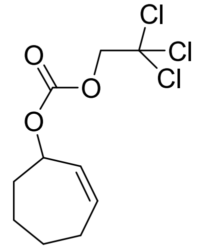

Cl
Cl
Cl
O
O
O

3h

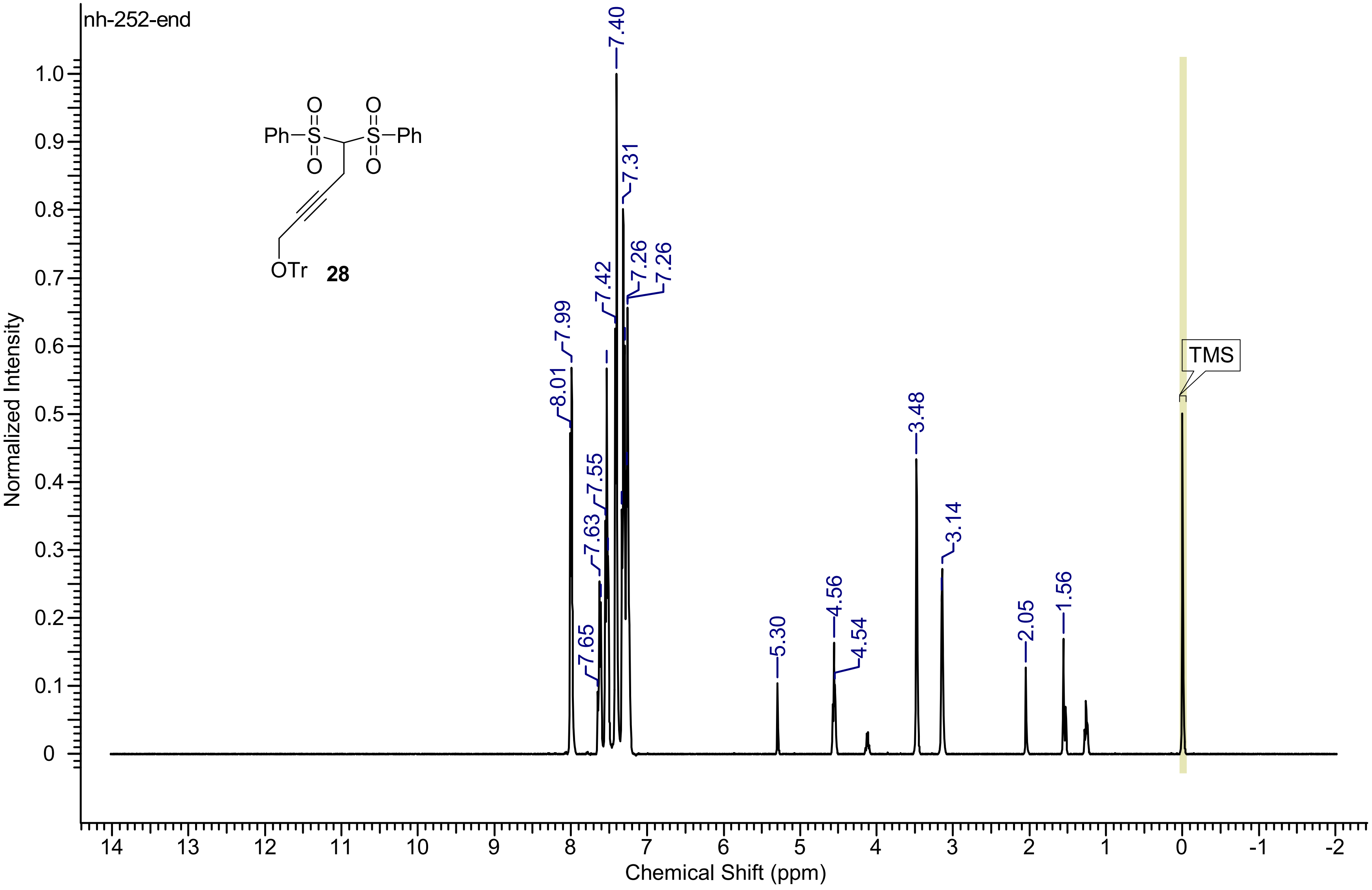
nh-252-end
Ph
S
O
O
S
O
O
Ph
OTr
28
8.01
7.99
7.65
7.63
7.55
7.42
7.40
7.31
7.26
7.26
5.30
4.56
4.54
3.48
3.14
2.05
1.56
TMS
Normalized Intensity
1.0
0.9
0.8
0.7
0.6
0.5
0.4
0.3
0.2
0.1
0
14
13
12
11
10
9
8
7
6
5
4
3
2
1
0
-1
-2
Chemical Shift (ppm)

SpinWorks 4: nh-252-13C

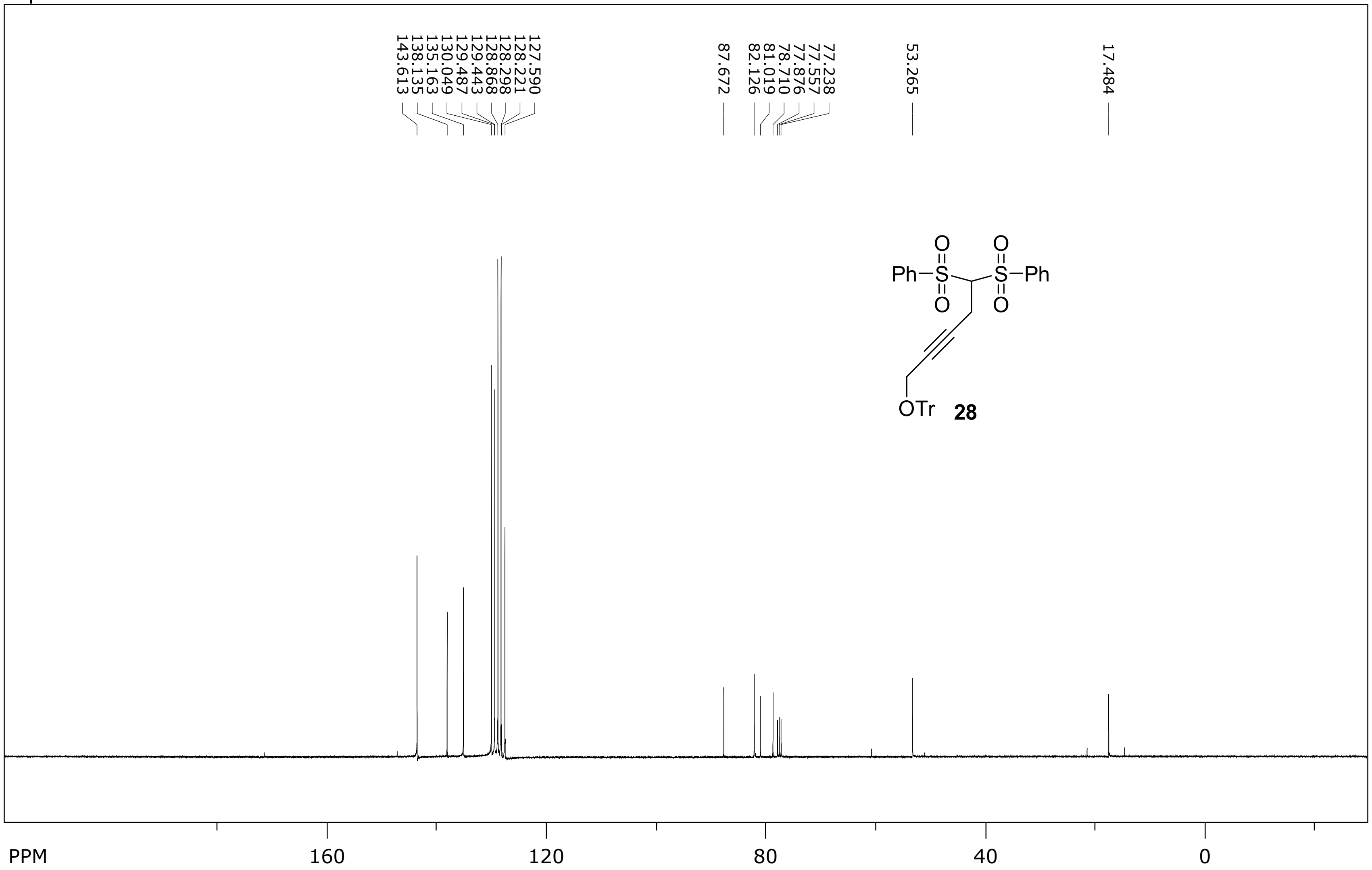

file: ...nhavare\nhavare\nh-252-13C.fid\fid block# 1 expt: "s2pul"
transmitter freq.: 100.617627 MHz
time domain size: 59968 points
width: 25000.00 Hz = 248.4654 ppm = 0.416889 Hz/pt
number of scans: 624

freq. of 0 ppm: 100.608102 MHz
processed size: 65536 complex points
LB: 0.500 GF: 0.0000

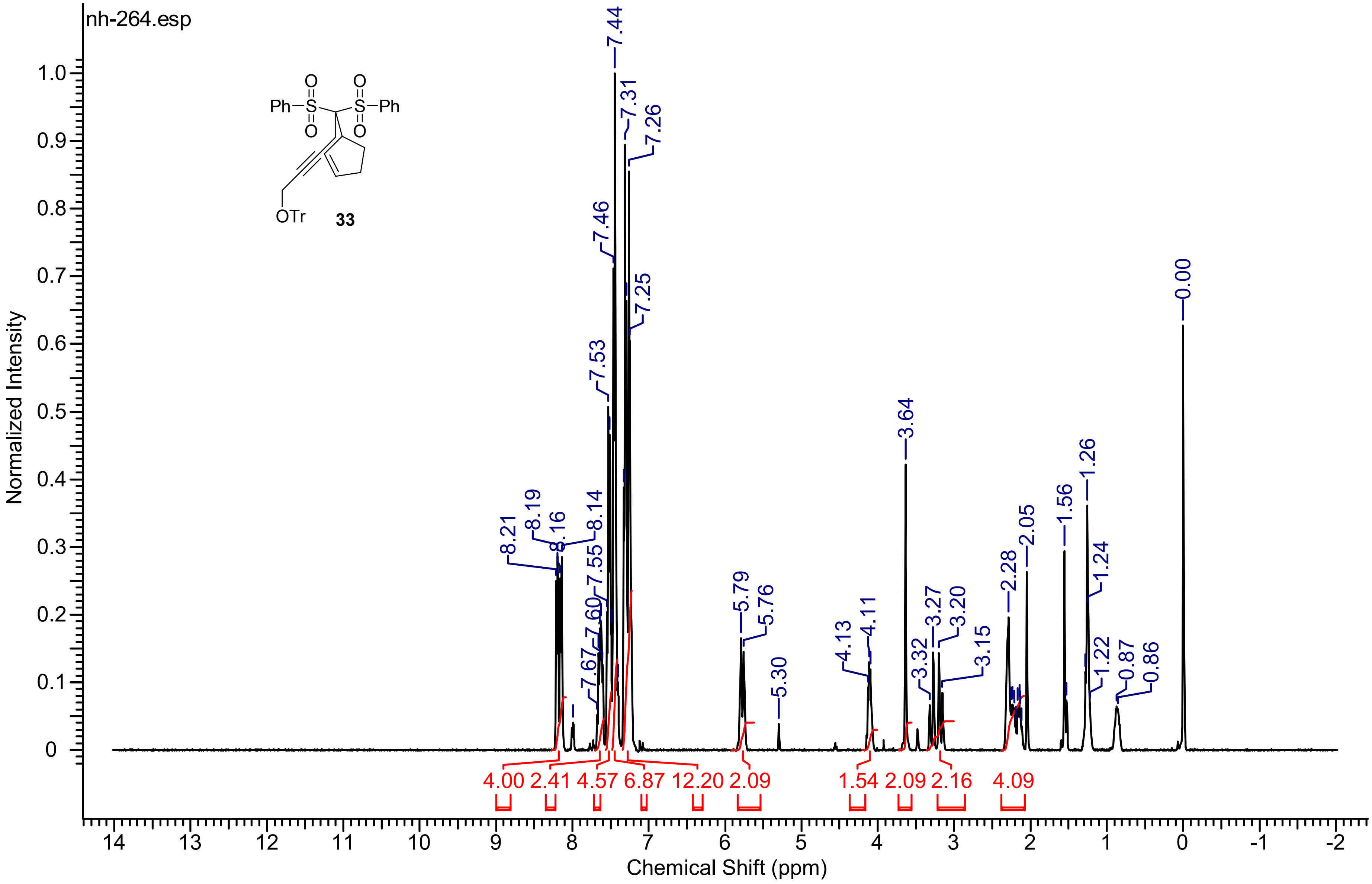

nh-264.esp
Normalized Intensity
Chemical Shift (ppm)
Ph
O
S
O
Ph
OTr
33
8.21
8.19
8.16
8.14
7.67
7.60
7.55
7.53
7.46
7.44
7.31
7.26
7.25
5.79
5.76
5.30
4.13
4.11
3.64
3.32
3.27
3.20
3.15
2.28
2.05
1.56
1.26
1.24
1.22
0.87
0.86
0.00
4.00
2.41
4.57
6.87
12.20
2.09
1.54
2.09
2.16
4.09

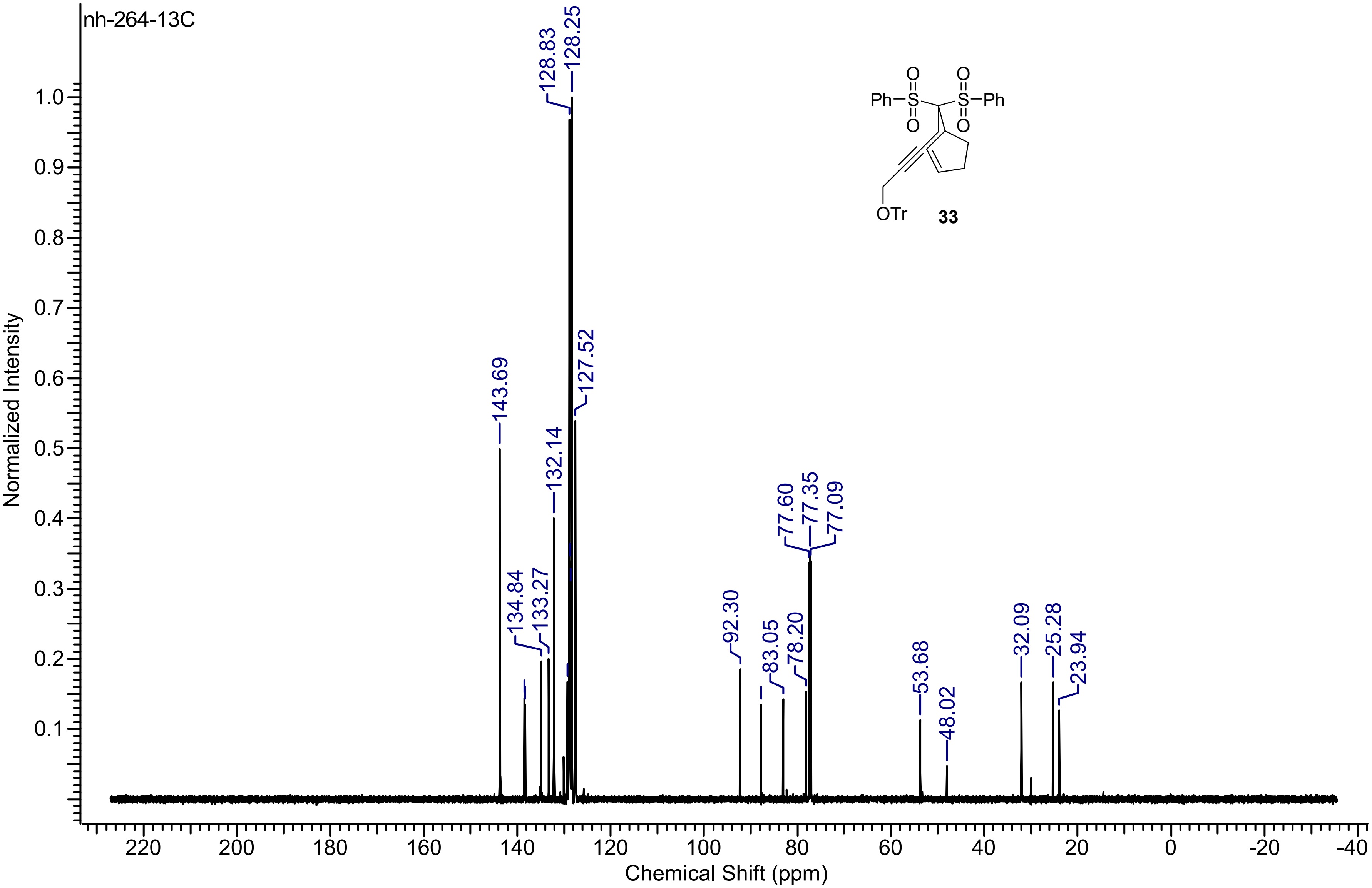
nh-264-13C
143.69
134.84
133.27
132.14
128.83
128.25
127.52
92.30
83.05
78.20
77.60
77.35
77.09
53.68
48.02
32.09
25.28
23.94
Normalized Intensity
Chemical Shift (ppm)
Ph
S
O
OTr
33

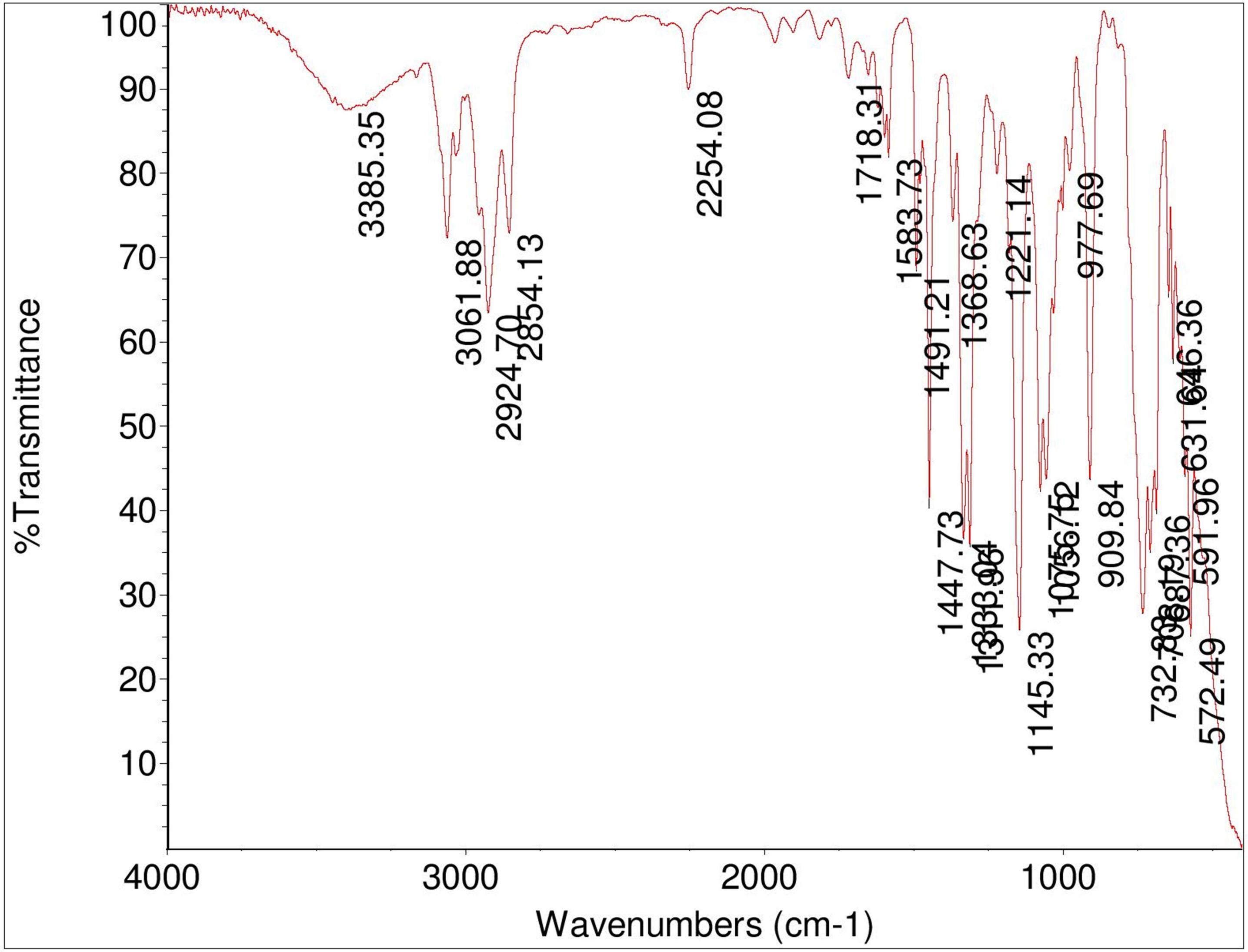
%Transmittance
Wavenumbers (cm-1)
3385.35
3061.88
2924.70
2854.13
2254.08
1718.31
1583.73
1491.21
1447.73
1368.63
1221.14
1145.33
977.69
909.84
591.96
572.49

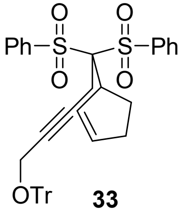

33

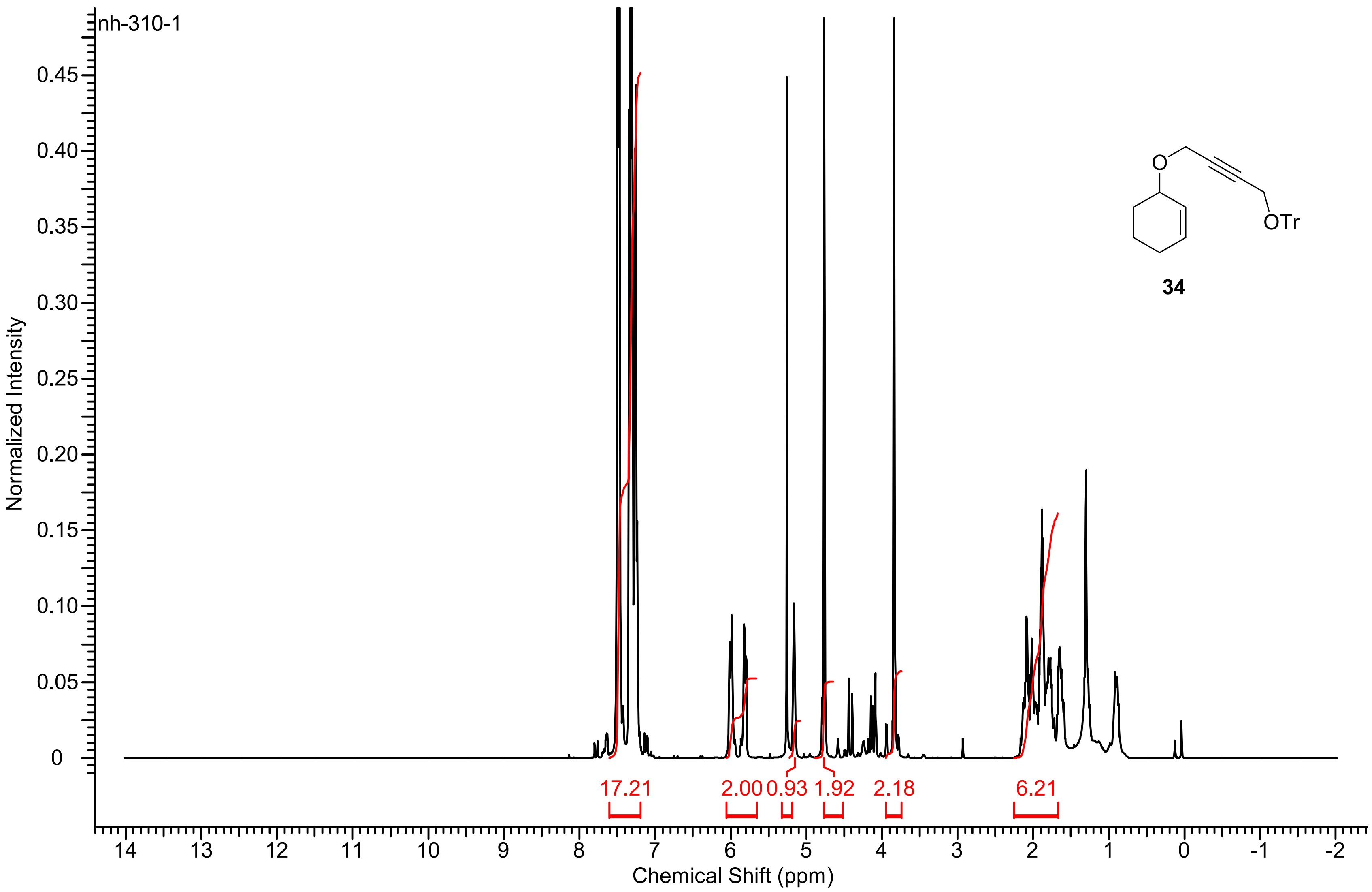
nh-310-1
OTr
34
Normalized Intensity
0.45
0.40
0.35
0.30
0.25
0.20
0.15
0.10
0.05
0
17.21
2.00
0.93
1.92
2.18
6.21
14 13 12 11 10 9 8 7 6 5 4 3 2 1 0 -1 -2
Chemical Shift (ppm)

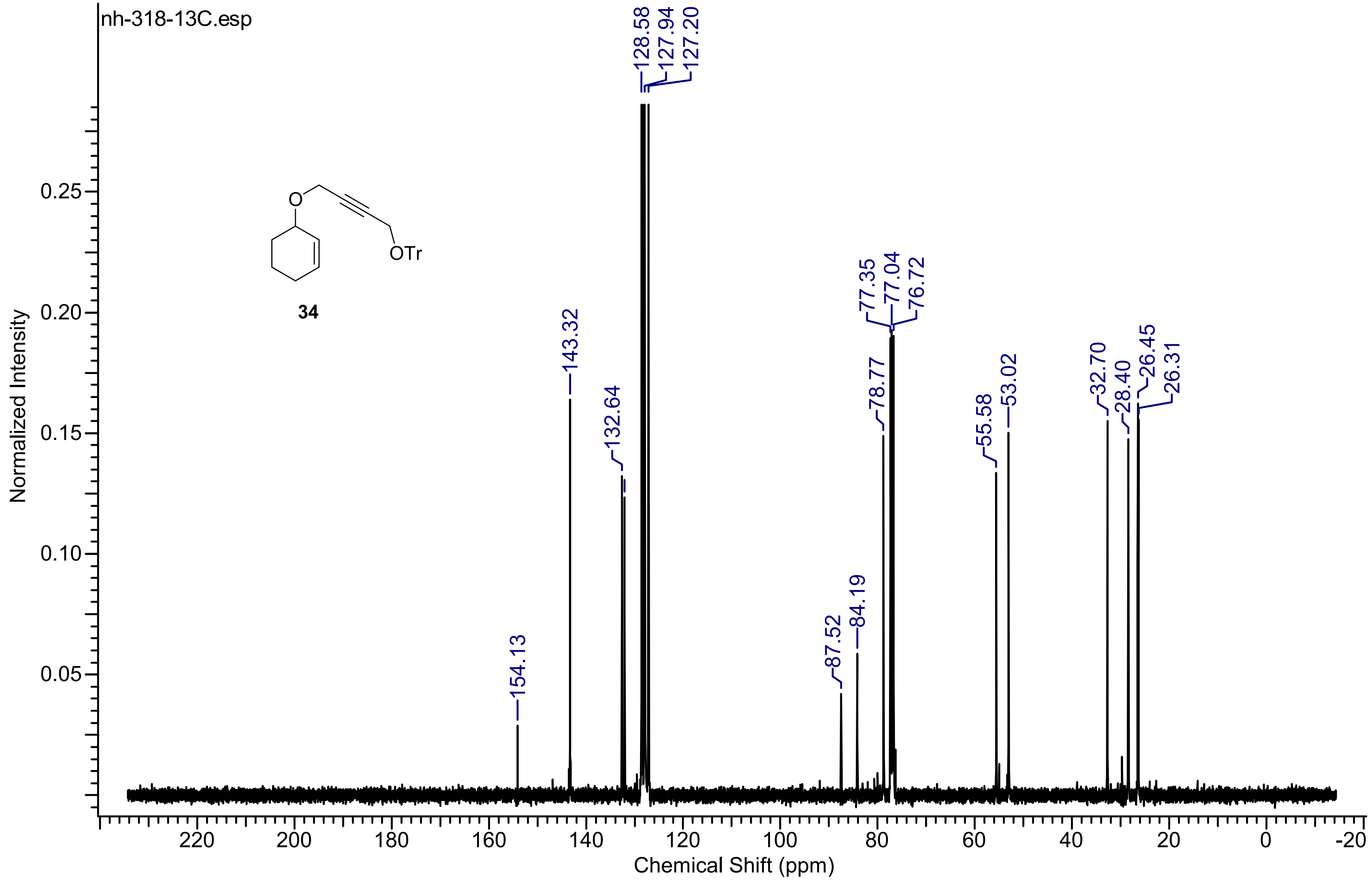

nh-318-13C.esp
OTr
O
34
154.13
143.32
132.64
128.58
127.94
127.20
87.52
84.19
78.77
77.35
77.04
76.72
55.58
53.02
32.70
28.40
26.45
26.31
Normalized Intensity
0.25
0.20
0.15
0.10
0.05
220
200
180
160
140
120
100
80
60
40
20
0
-20
Chemical Shift (ppm)

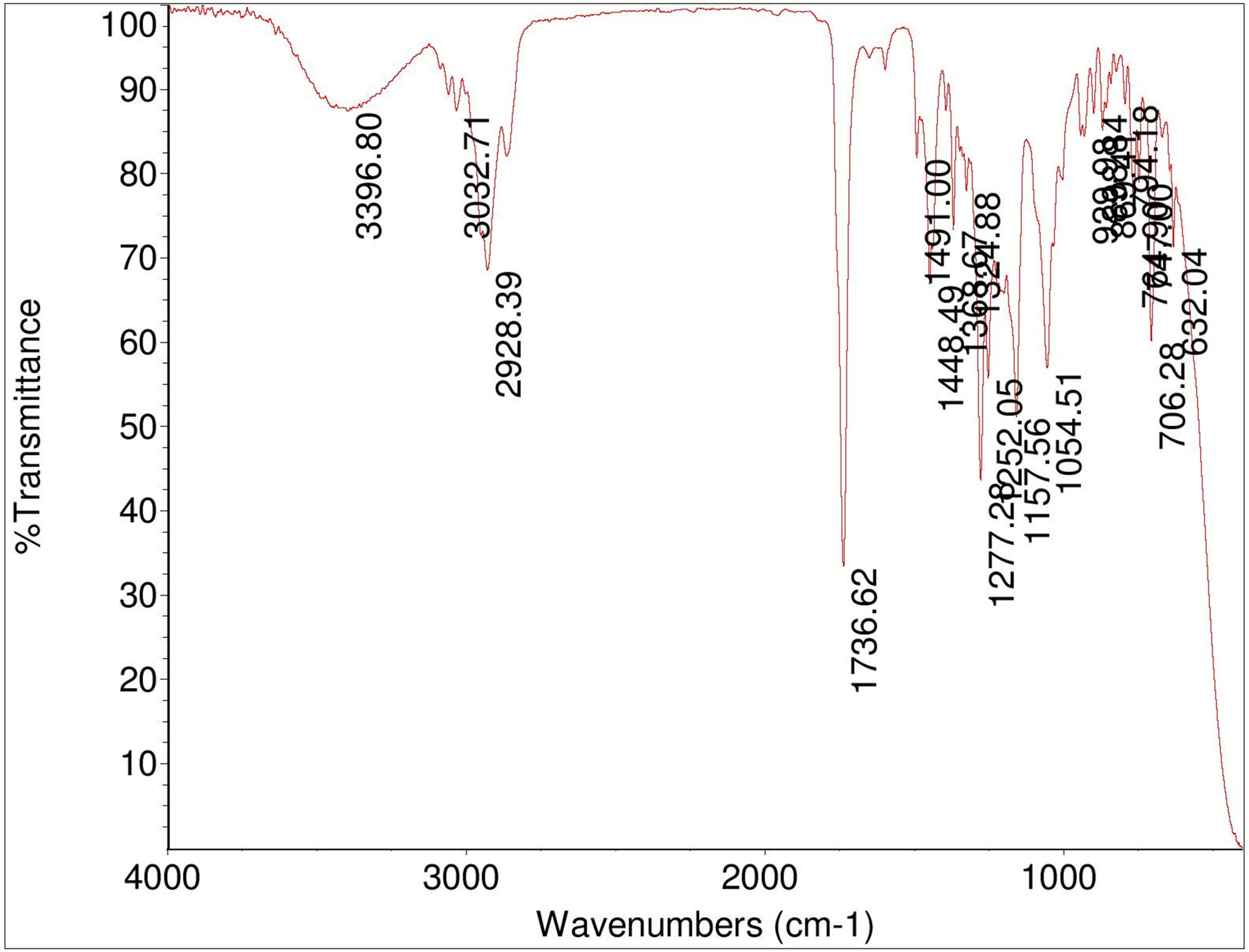
%Transmittance
Wavenumbers (cm-1)
100
90
80
70
60
50
40
30
20
10
4000
3000
2000
1000
3396.80
3032.71
2928.39
1736.62
1491.00
1448.49
1277.28
1252.05
1157.56
1054.51
706.28
632.04

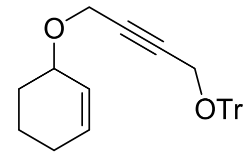
O
OTr

34

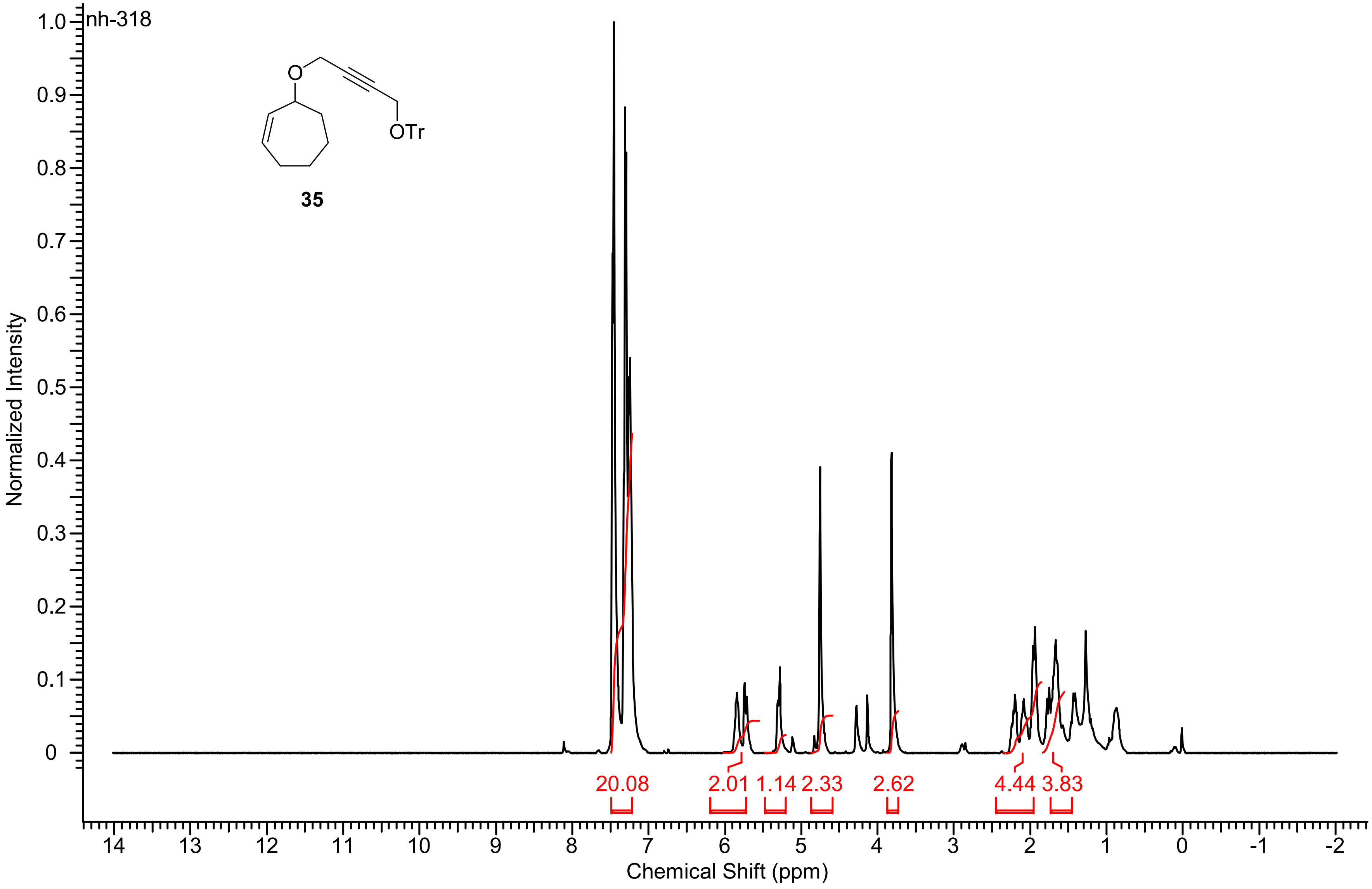
nh-318
OTr
35
20.08
2.01
1.14
2.33
2.62
4.44
3.83
Normalized Intensity
Chemical Shift (ppm)

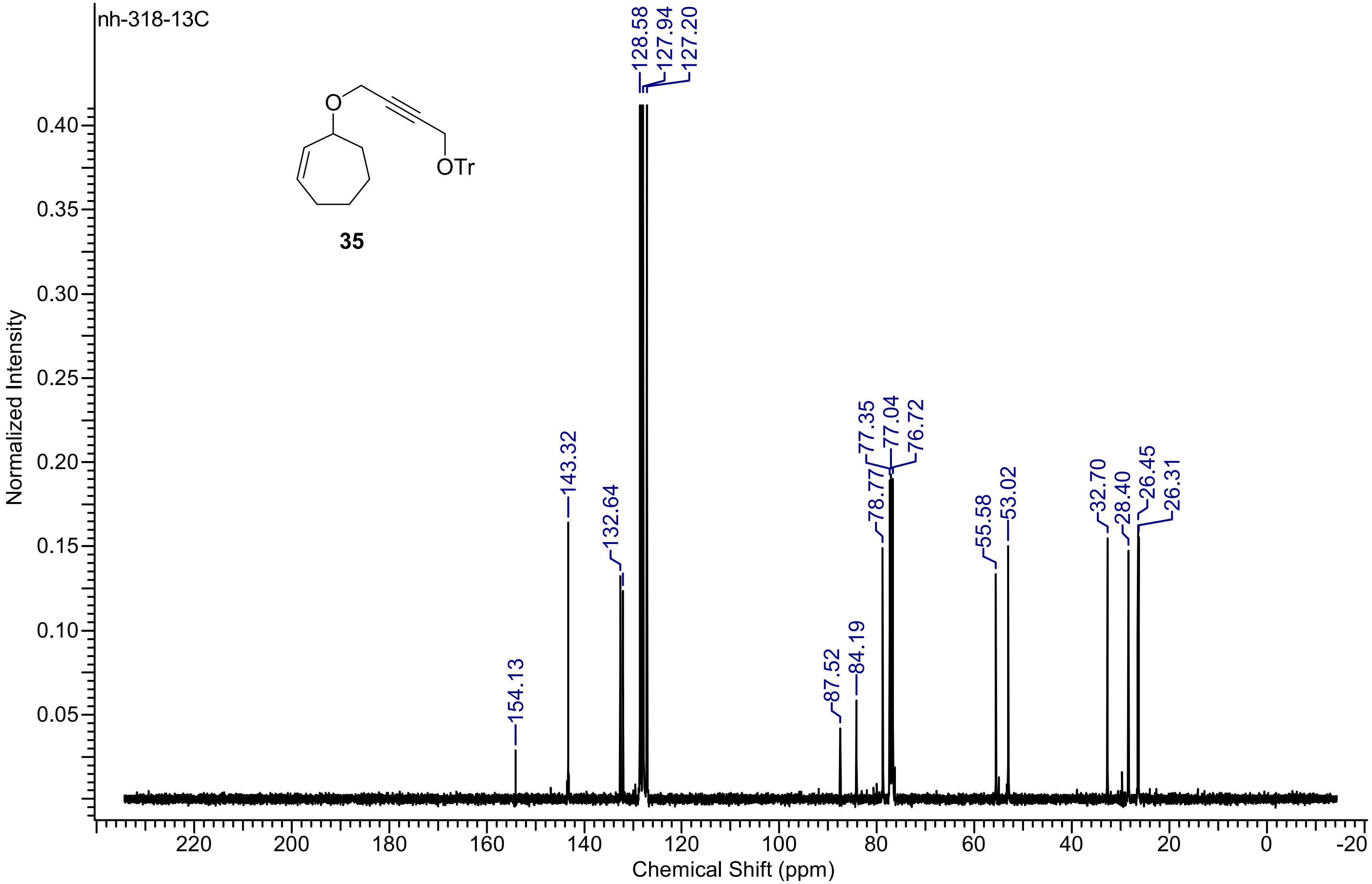
nh-318-13C
OTr
35
128.58
127.94
127.20
143.32
132.64
154.13
77.35
77.04
76.72
78.77
87.52
84.19
55.58
53.02
32.70
28.40
26.45
26.31
Normalized Intensity
Chemical Shift (ppm)

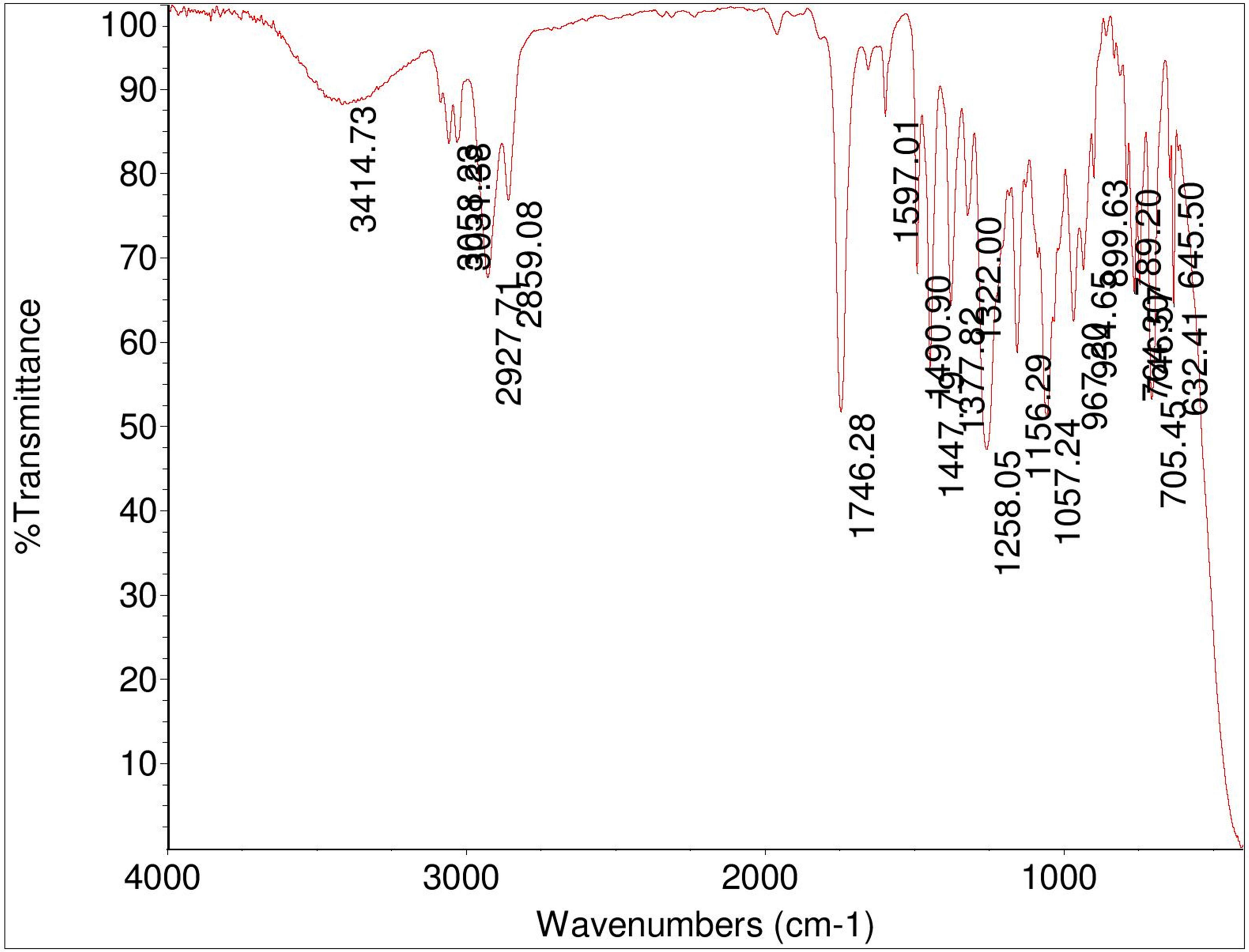
%Transmittance
Wavenumbers (cm-1)
3414.73
2927.71
2859.08
1746.28
1597.01
1490.90
1447.74
1377.82
1322.00
1258.05
1156.29
1057.24
899.63
789.20
705.45
645.50
632.41

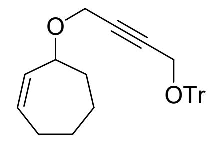
O
OTr

35

Table 2, **Entry 1**

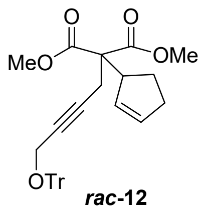

***rac*-12**

Data File C:\CHEM32\1\DATA\NIZAM\DEF_LC_CORRECT 2016-07-21 16-19-31\NH-98.D
Sample Name: NH-98

===

Acq. Operator : NIZAM　　Seq. Line : 2
Acq. Instrument : Instrument 1 Classic　　Location : Vial 11
Injection Date : 7/21/2016 4:39:21 PM　　Inj : 1
Inj Volume : 5.0 µl
Acq. Method : C:\CHEM32\1\DATA\NIZAM\DEF_LC_CORRECT 2016-07-21 16-19-31\IA, 90-10 HEPT-IPA, 0,8 ML-MIN,254NM,30M.M
Last changed : 10/15/2012 4:26:35 PM by Fred
Analysis Method : C:\CHEM32\1\DATA\MARIUS\DEF_LC_CORRECT 2017-01-21 23-13-46\IA, 98-2 HEPT-IPA, 0,8 ML-MIN,254NM,30M.M
Last changed : 5/23/2015 5:47:14 PM by gnanam
Method Info : IA, 98/2 heptane/isopropanol, 0.8 ml/min, 254 nm, 30 min

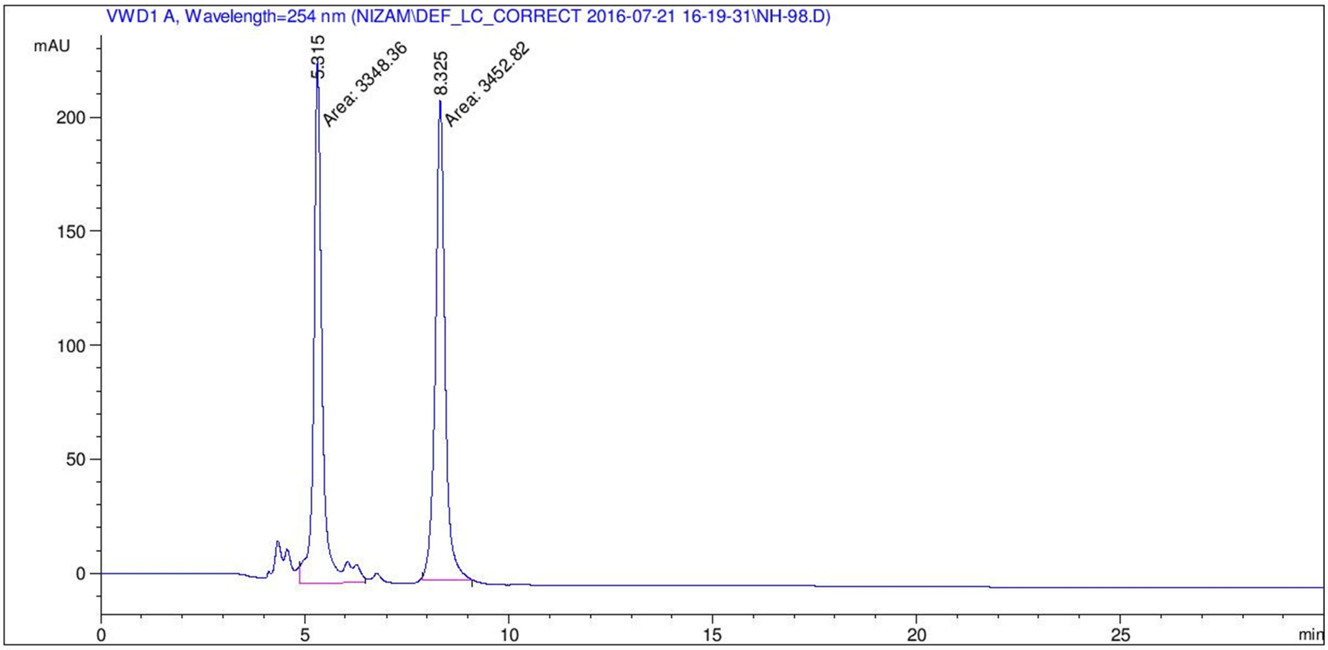

===

Area Percent Report

===

Sorted By : Signal
Multiplier: : 1.0000
Dilution: : 1.0000
Use Multiplier & Dilution Factor with ISTDs

Signal 1: VWD1 A, Wavelength=254 nm

Peak #	RetTime [min]	Type	Width [min]	Area mAU *s	Height [mAU]	Area %
1	5.315	MM	0.2439	3348.35986	228.79910	49.2320
2	8.325	MM	0.2735	3452.82373	210.39929	50.7680

Table 2, Entry 3

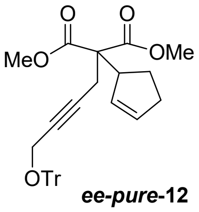

ee-pure-**12**

Data File C:\CHEM32\1\DATA\NIZAM\DEF_LC_CORRECT 2016-07-26 17-25-03\NH-104-C.D
Sample Name: NH-104-C

===
Acq. Operator : NIZAM Seq. Line : 6
Acq. Instrument : Instrument 1 Classic Location : Vial 21
Injection Date : 7/26/2016 7:48:09 PM Inj : 1
Inj Volume : 5.0 µl
Acq. Method : C:\CHEM32\1\DATA\NIZAM\DEF_LC_CORRECT 2016-07-26 17-25-03\IA, 90-10 HEPT-IPA, 0,8 ML-MIN,254NM,30M.M
Last changed : 10/15/2012 4:26:35 PM by Fred
Analysis Method : C:\CHEM32\1\DATA\MARIUS\DEF_LC_CORRECT 2017-01-21 23-13-46\IA, 98-2 HEPT-IPA, 0,8 ML-MIN,254NM,30M.M
Last changed : 5/23/2015 5:47:14 PM by gnanam
Method Info : IA, 98/2 heptane/isopropanol, 0.8 ml/min, 254 nm, 30 min

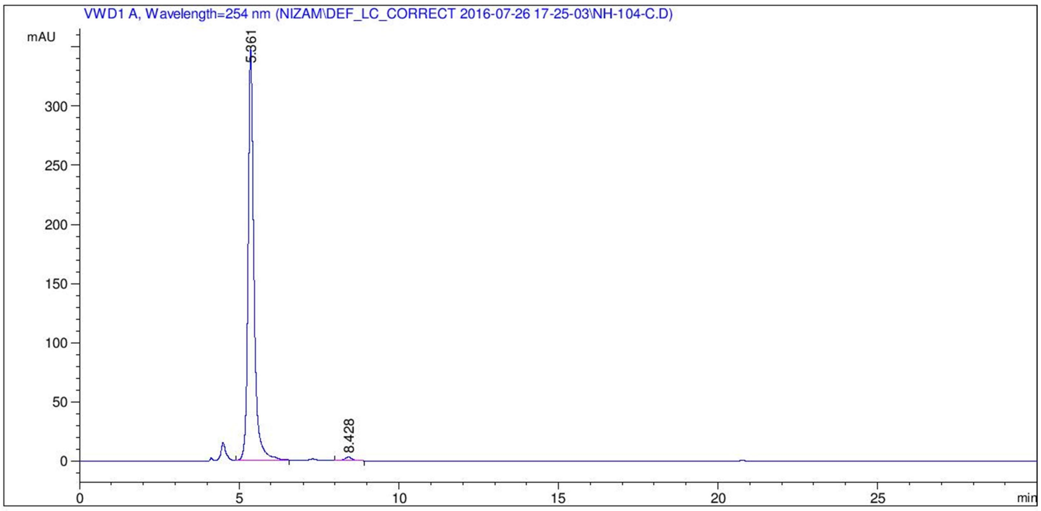

===
Area Percent Report
===

Sorted By : Signal
Multiplier: : 1.0000
Dilution: : 1.0000
Use Multiplier & Dilution Factor with ISTDs

Signal 1: VWD1 A, Wavelength=254 nm

Peak #	RetTime [min]	Type	Width [min]	Area mAU *s	Height [mAU]	Area %
1	5.361	BB	0.1888	4451.47021	347.36212	98.8490
2	8.428	BB	0.2423	51.83323	3.13620	1.1510

Table 2, Entry 4: Results using ligand (*S,S,S*)-**9**

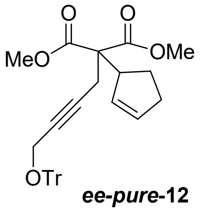

***ee-pure*-12**

Data File C:\CHEM32\1\DATA\NIZAM\DEF_LC_CORRECT 2016-11-27 00-39-19\NH-260.D
Sample Name: NH-260

===

Acq. Operator : NIZAM Seq. Line : 20
Acq. Instrument : Instrument 1 Classic Location : Vial 21
Injection Date : 11/27/2016 9:30:21 AM Inj : 1
Inj Volume : 5.0 µl
Acq. Method : C:\CHEM32\1\DATA\NIZAM\DEF_LC_CORRECT 2016-11-27 00-39-19\IA, 90-10 HEPT-IPA, 0,8 ML-MIN,254NM,30M.M
Last changed : 10/15/2012 4:26:35 PM by Fred
Analysis Method : C:\CHEM32\1\DATA\MARIUS\DEF_LC_CORRECT 2017-01-21 23-13-46\IA, 98-2 HEPT-IPA, 0,8 ML-MIN,254NM,30M.M
Last changed : 5/23/2015 5:47:14 PM by gnanam
Method Info : IA, 98/2 heptane/isopropanol, 0.8 ml/min, 254 nm, 30 min

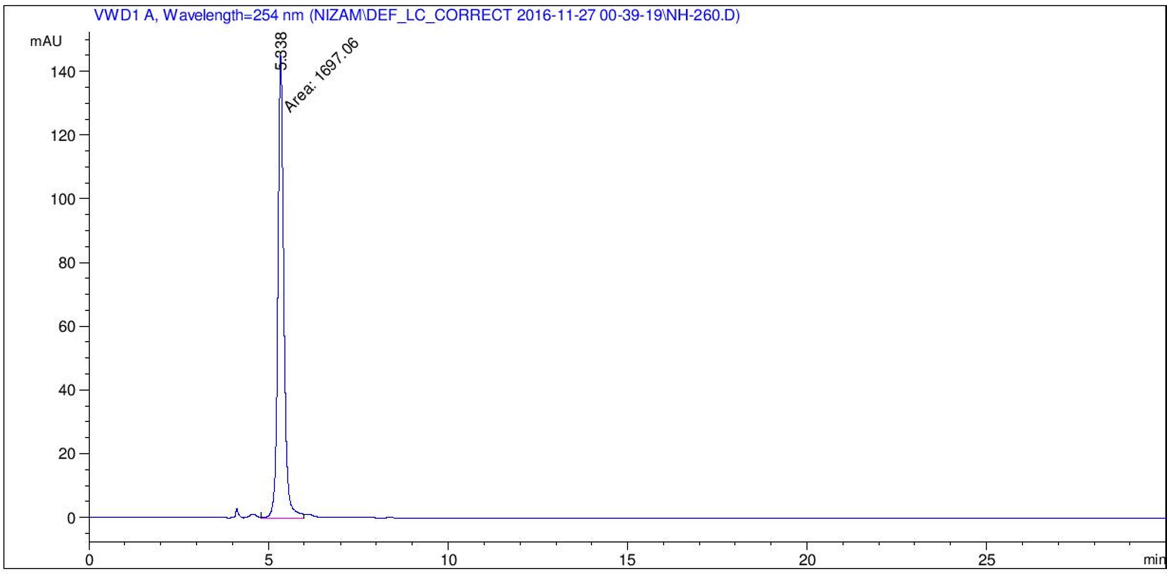

===

Area Percent Report

===

Sorted By : Signal
Multiplier: : 1.0000
Dilution: : 1.0000
Use Multiplier & Dilution Factor with ISTDs

Signal 1: VWD1 A, Wavelength=254 nm

Peak #	RetTime [min]	Type	Width [min]	Area mAU *s	Height [mAU]	Area %
1	5.338	MM	0.1946	1697.05640	145.37408	100.0000

Table 2, Entry 2: Results using ligand (*S*,*R*,*R*)-**10**

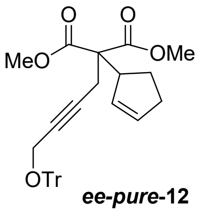

***ee-pure*-12**

Data File C:\CHEM32\1\DATA\NIZAM\DEF_LC_CORRECT 2016-11-27 00-39-19\NH-261.D
Sample Name: NH-261

===
Acq. Operator : NIZAM Seq. Line : 21
Acq. Instrument : Instrument 1 Classic Location : Vial 31
Injection Date : 11/27/2016 10:01:58 AM Inj : 1
Inj Volume : 5.0 µl
Acq. Method : C:\CHEM32\1\DATA\NIZAM\DEF_LC_CORRECT 2016-11-27 00-39-19\IA, 90-10 HEPT-IPA, 0,8 ML-MIN,254NM,30M.M
Last changed : 10/15/2012 4:26:35 PM by Fred
Analysis Method : C:\CHEM32\1\DATA\MARIUS\DEF_LC_CORRECT 2017-01-21 23-13-46\IA, 98-2 HEPT-IPA, 0,8 ML-MIN,254NM,30M.M
Last changed : 5/23/2015 5:47:14 PM by gnanam
Method Info : IA, 98/2 heptane/isopropanol, 0.8 ml/min, 254 nm, 30 min

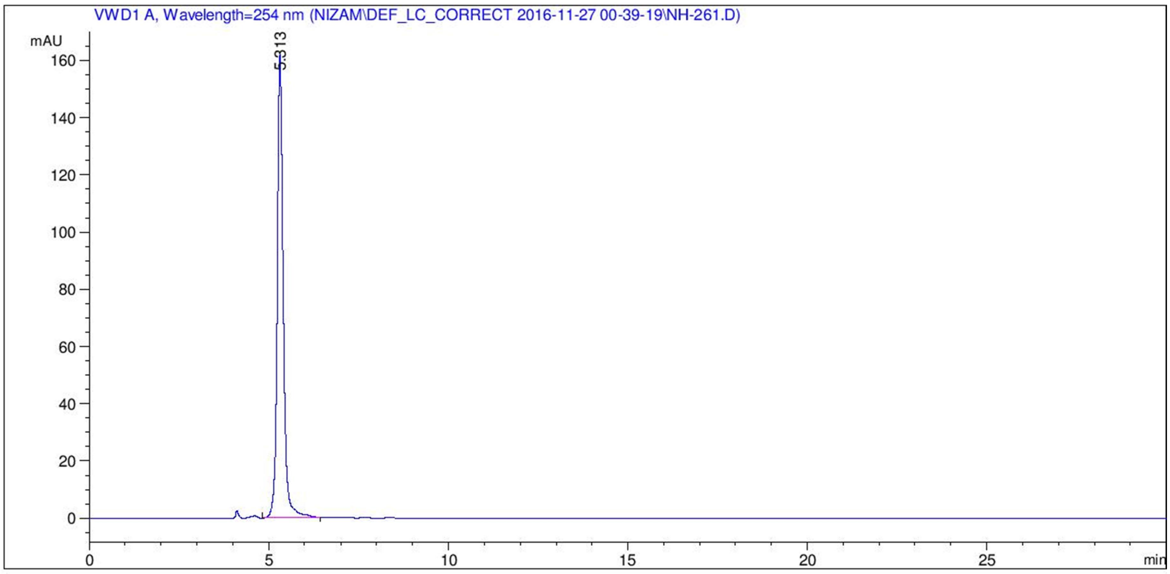

===
Area Percent Report
===

Sorted By : Signal
Multiplier: : 1.0000
Dilution: : 1.0000
Use Multiplier & Dilution Factor with ISTDs

Signal 1: VWD1 A, Wavelength=254 nm

Peak #	RetTime [min]	Type	Width [min]	Area mAU *s	Height [mAU]	Area %
1	5.313	VB	0.1738	1886.14941	161.88745	100.0000

Table 2, Entry 5

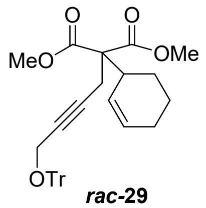

***rac*-29**

```
Title        :
Run File     : c:\star\data\nizam\nh-101-b-hpt-ipa-99-1-254  nm 0.8 ml- ad.run
Method File  : c:\star\data\nizam\nh-101-b-hpt-ipa-99-1-254  nm 0.8 ml- ad-1.mth
Sample ID    : Default Sample

Injection Date: 7/27/2016 4:50 PM     Calculation Date: 12/23/2016 3:28 PM

Operator    : Operator                Detector Type: 0800 (1 Volt)
Workstation: TROST-HPLC-LEFý HÚp"     Bus Address   : 80
Instrument  : Instrument #1           Sample Rate   : 50.00 Hz
Channel     : 1 = 1                   Run Time      : 60.000 min

** LC Workstation Multi Instrument (Demo) Version 6.41 ** 05000-31c8-fa9-30a1 **

Chart Speed =    0.33 cm/min   Attenuation = 93         Zero Offset = 26%
Start Time  =   0.000  min      End Time    = 60.000  min   Min / Tick  = 1.00
```

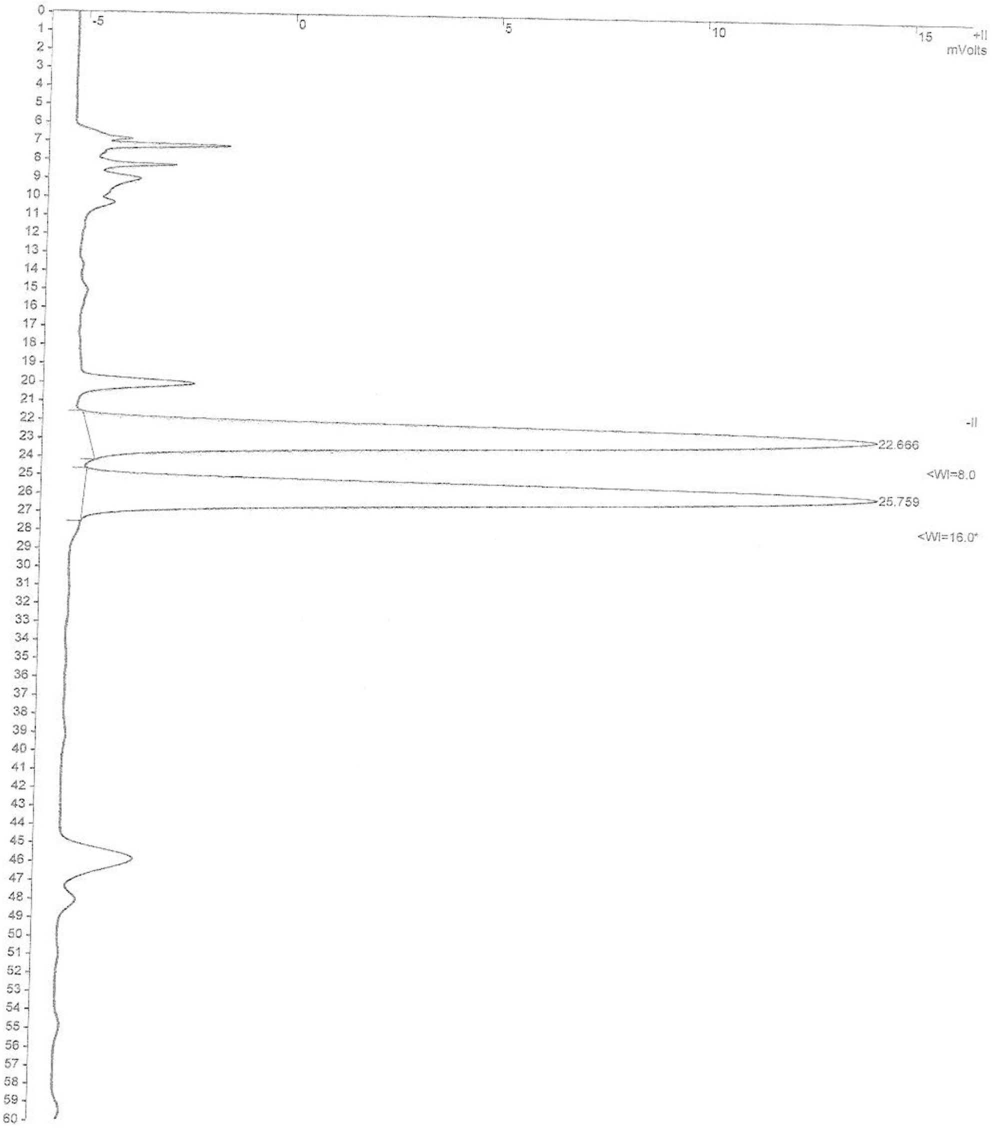

Table 2, Entry 6

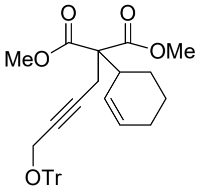

***enantioenriched*-29**

```
Title        :
Run File     : c:\star\data\nizam\nh-112-hpt-ipa-99-1-254  nm 0.8 ml- ad.run
Method File  : c:\star\data\nizam\nh-112-hpt-ipa-99-1-254  nm 0.8 ml- ad-1.mth
Sample ID    : Default Sample

Injection Date: 8/6/2016 8:19 PM     Calculation Date: 8/6/2016 9:19 PM

Operator    : Operator               Detector Type: 0800 (1 Volt)
Workstation: TROST-HPLC-LEFý HÚp"    Bus Address  : 80
Instrument  : Instrument #1          Sample Rate  : 50.00 Hz
Channel     : 1 = 1                  Run Time     : 58.000 min

** LC Workstation Multi Instrument (Demo) Version 6.41 ** 05000-31c8-fa9-30a1 **

Chart Speed =   0.34 cm/min   Attenuation = 144        Zero Offset = 9%
Start Time  =  0.000   min     End Time    = 58.000  min   Min / Tick  = 1.00
```

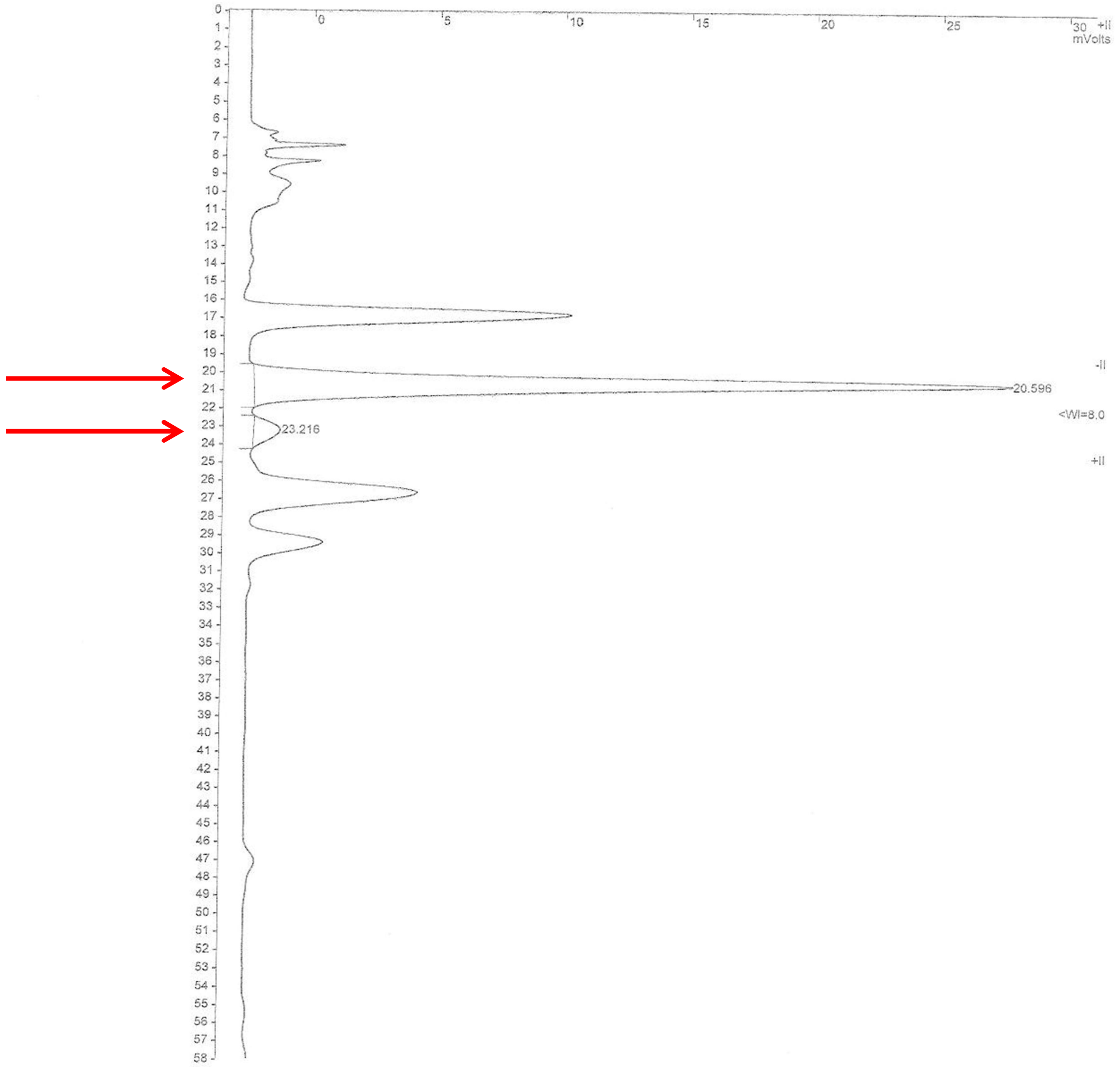

Table 2, Entry 7

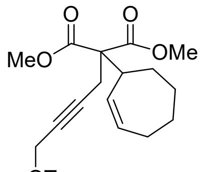

***rac*-31**

After deprotection of trityl group, the racemic compound was separated using chiral GC-colomun Cyclosil B: with following method: Init Temp: 50; init time: 30; rate 1: 10; final temp. 1: 200; final time-1: 100; rate 2: 10; final-temp. 2: 50.

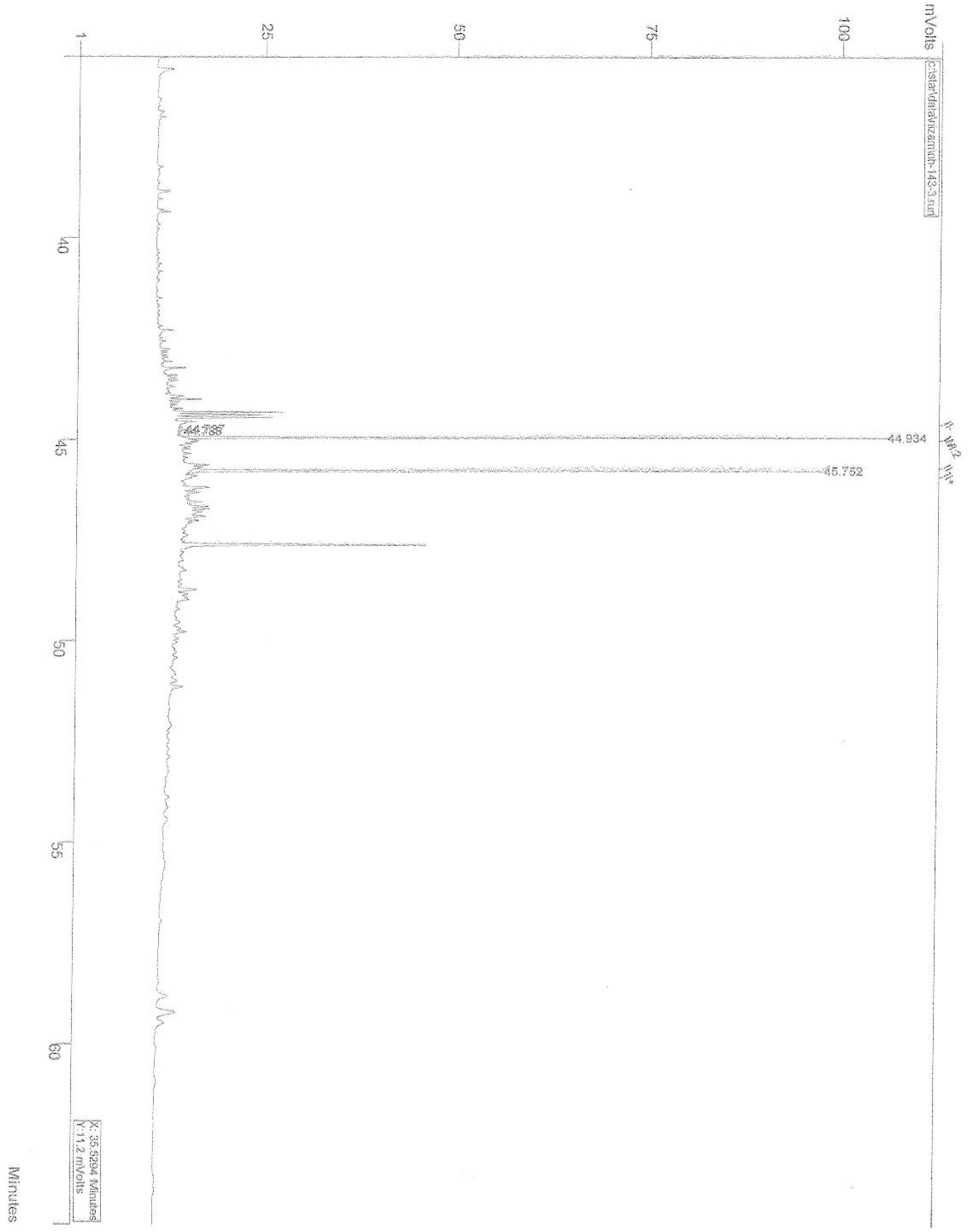

Table 2, Entry 8

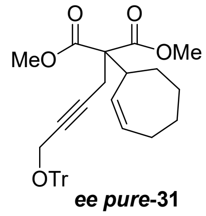

ee pure-**31**

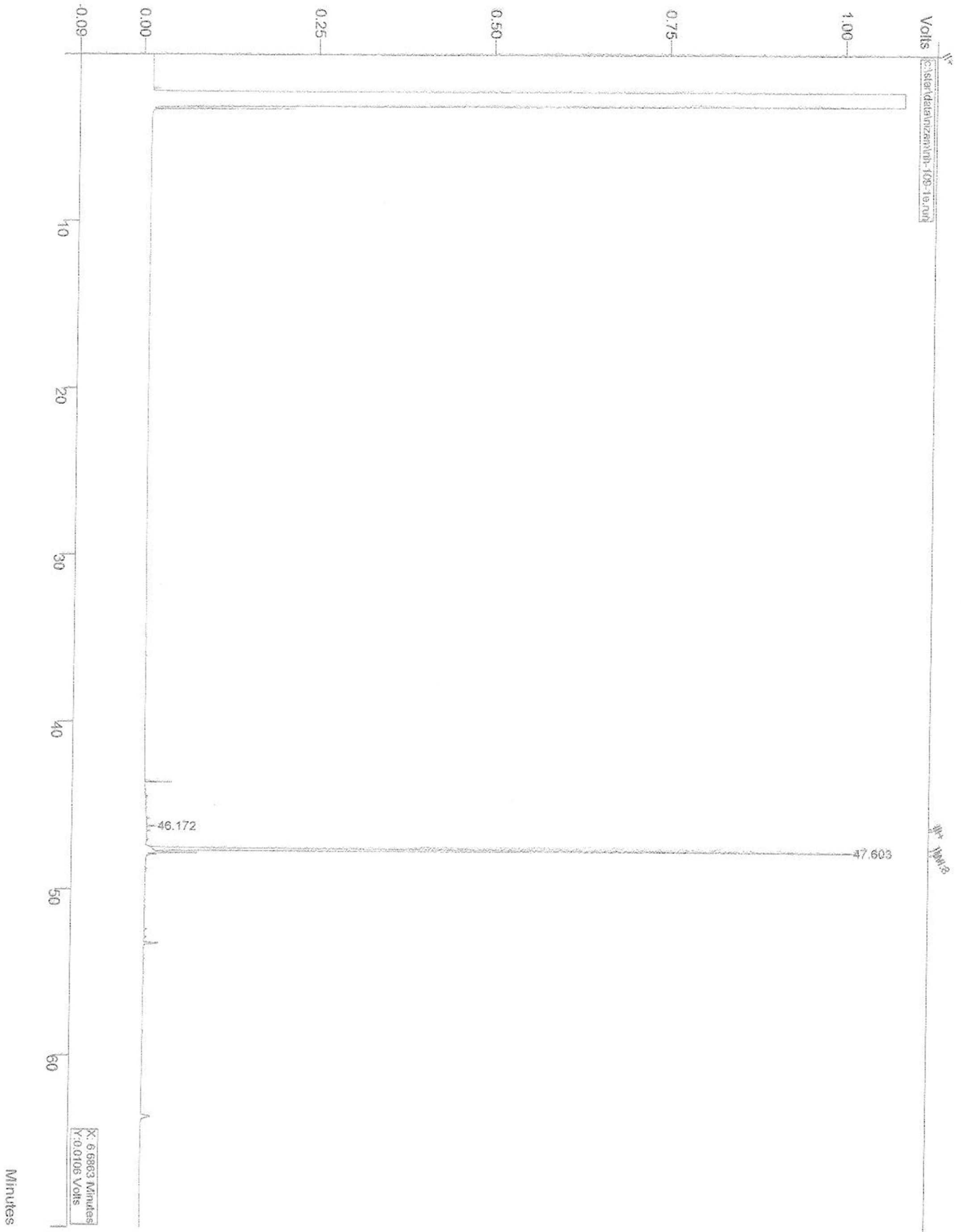

Table 2, Entry 9

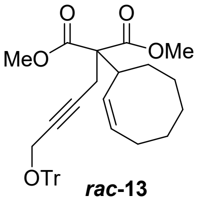

rac-**13**

```
Data File C:\CHEM32\1\DATA\NIZAM\DEF_LC_CORRECT 2016-09-06 18-20-40\NH-152-1C-MIX.D
Sample Name: nh-152-1c-mix

    =====================================================================
    Acq. Operator   : nizam                          Seq. Line :   8
    Acq. Instrument : Instrument 1 Classic            Location : Vial 11
    Injection Date  : 9/6/2016 9:19:17 PM                  Inj :   1
                                                    Inj Volume : 5.0 µl
    Acq. Method     : C:\CHEM32\1\DATA\NIZAM\DEF_LC_CORRECT 2016-09-06 18-20-40\IC, 90-10 HEPT-IPA,
                      0,8 ML-MIN,254NM,30M.M
    Last changed    : 3/7/2011 2:58:51 PM
    Analysis Method : C:\CHEM32\1\DATA\MARIUS\DEF_LC_CORRECT 2017-01-21 23-13-46\IA, 98-2 HEPT-IPA,
                      0,8 ML-MIN,254NM,30M.M
    Last changed    : 5/23/2015 5:47:14 PM by gnanam
    Method Info     : IA, 98/2 heptane/isopropanol, 0.8 ml/min, 254 nm, 30 min
```

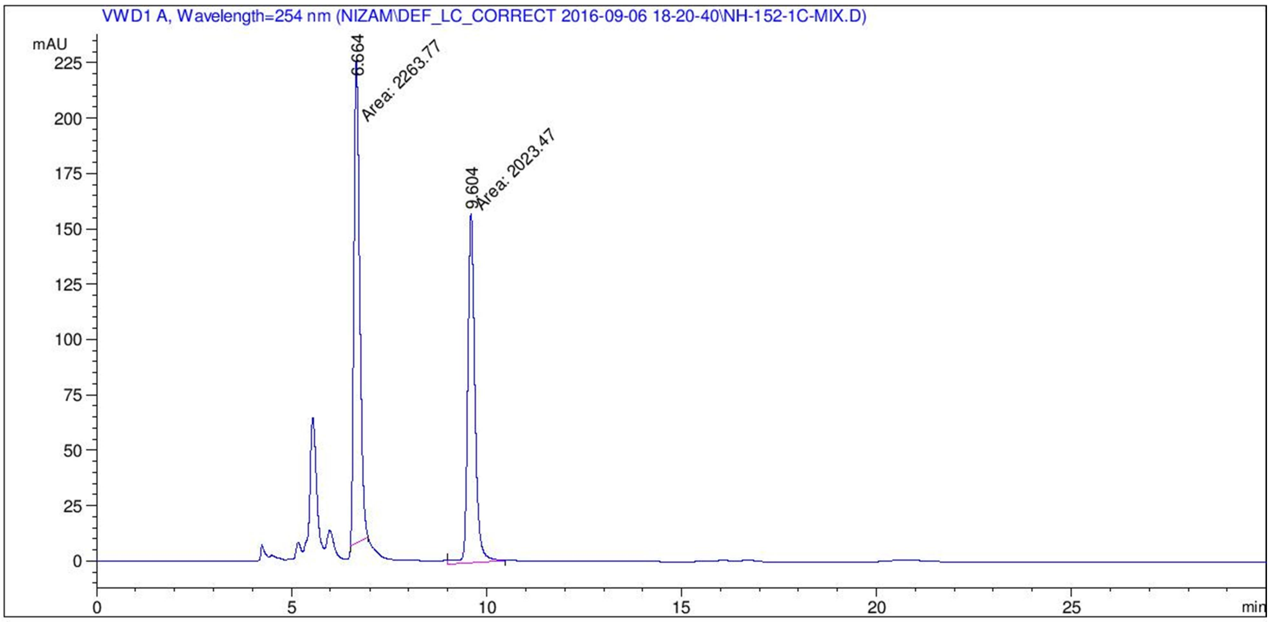


```
    =====================================================================
                             Area Percent Report
    =====================================================================

    Sorted By             :      Signal
    Multiplier:                    :      1.0000
    Dilution:                      :      1.0000
    Use Multiplier & Dilution Factor with ISTDs


    Signal 1: VWD1 A, Wavelength=254 nm

    Peak RetTime Type  Width     Area      Height     Area
      #   [min]        [min]  mAU    *s   [mAU    ]     %
    ----|-------|----|-------|----------|----------|--------|
       1   6.664 MM    0.1727 2263.77393  218.43097  52.8026
       2   9.604 MM    0.2140 2023.46863  157.60985  47.1974
```

Table 2, Entry 11

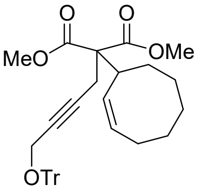

***enantioenriched*-13**

Data File C:\CHEM32\1\DATA\NIZAM\DEF_LC_CORRECT 2016-10-04 13-10-01\NH-174.D
Sample Name: NH-174

===

Acq. Operator : NIZAM Seq. Line : 5
Acq. Instrument : Instrument 1 Classic Location : Vial 41
Injection Date : 10/4/2016 4:44:01 PM Inj : 1
Inj Volume : 5.0 µl
Acq. Method : C:\CHEM32\1\DATA\NIZAM\DEF_LC_CORRECT 2016-10-04 13-10-01\IC, 90-10 HEPT-IPA, 0,8 ML-MIN,254NM,30M.M
Last changed : 3/7/2011 2:58:51 PM
Analysis Method : C:\CHEM32\1\DATA\MARIUS\DEF_LC_CORRECT 2017-01-21 23-13-46\IA, 98-2 HEPT-IPA, 0,8 ML-MIN,254NM,30M.M
Last changed : 5/23/2015 5:47:14 PM by gnanam
Method Info : IA, 98/2 heptane/isopropanol, 0.8 ml/min, 254 nm, 30 min

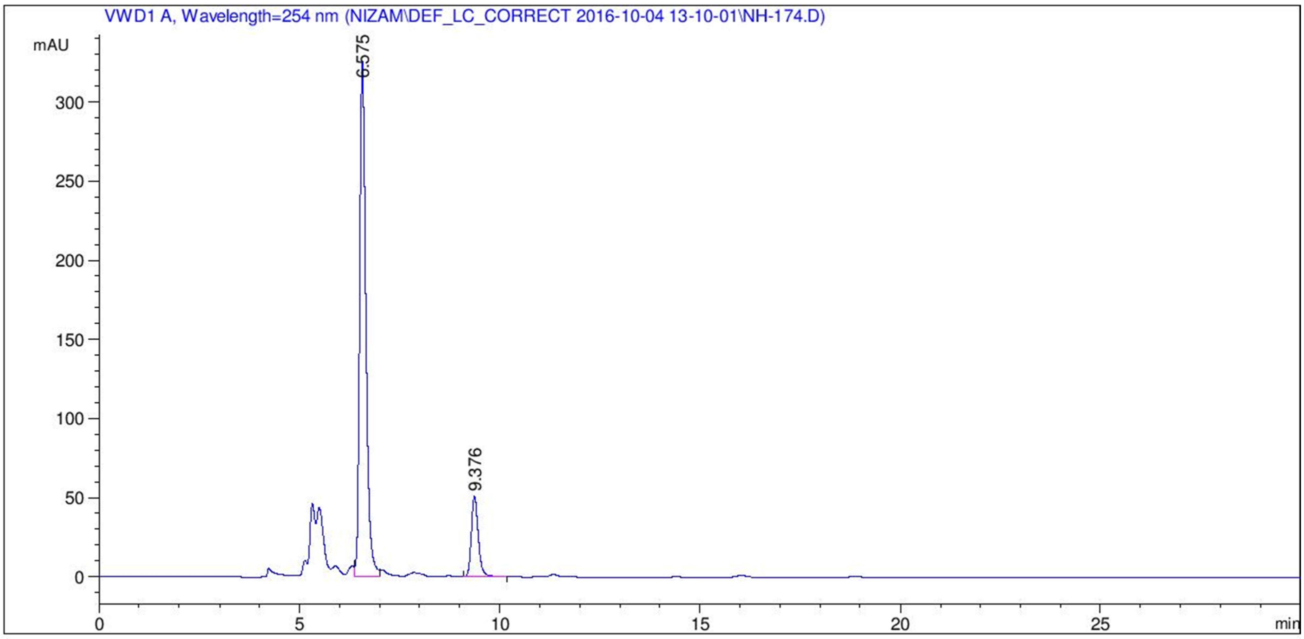

===

Area Percent Report

===

Sorted By : Signal
Multiplier: : 1.0000
Dilution: : 1.0000
Use Multiplier & Dilution Factor with ISTDs

Signal 1: VWD1 A, Wavelength=254 nm

Peak #	RetTime [min]	Type	Width [min]	Area mAU *s	Height [mAU]	Area %
1	6.575	VV	0.1663	3549.53418	326.18906	85.1267
2	9.376	VB	0.1854	620.17120	51.05568	14.8733

Table 2, Entry 12

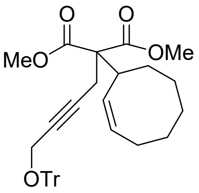

***ee pure*-13**

```
Data File C:\CHEM32\1\DATA\NIZAM\DEF_LC_CORRECT 2016-10-03 22-21-54\NH-186.D
Sample Name: NH-186

=====================================================================
Acq. Operator   : NIZAM                          Seq. Line :   4
Acq. Instrument : Instrument 1 Classic            Location : Vial 31
Injection Date  : 10/4/2016 12:14:27 AM                Inj :   1
                                               Inj Volume : 5.0 µl
Acq. Method     : C:\CHEM32\1\DATA\NIZAM\DEF_LC_CORRECT 2016-10-03 22-21-54\IC, 90-10 HEPT-IPA,
                  0,8 ML-MIN,254NM,30M.M
Last changed    : 3/7/2011 2:58:51 PM
Analysis Method : C:\CHEM32\1\DATA\MARIUS\DEF_LC_CORRECT 2017-01-21 23-13-46\IA, 98-2 HEPT-IPA,
                  0,8 ML-MIN,254NM,30M.M
Last changed    : 5/23/2015 5:47:14 PM by gnanam
Method Info     : IA, 98/2 heptane/isopropanol, 0.8 ml/min, 254 nm, 30 min
```

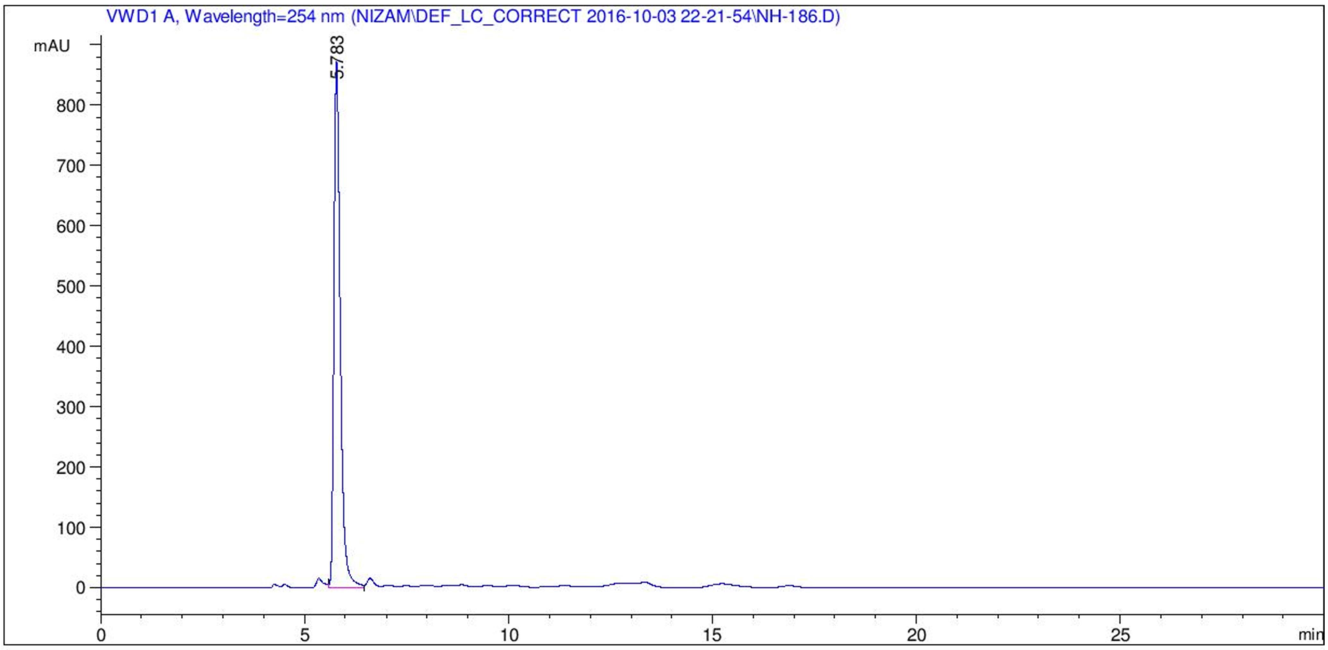


```
=====================================================================
                          Area Percent Report
=====================================================================

Sorted By             :      Signal
Multiplier:                   :      1.0000
Dilution:                     :      1.0000
Use Multiplier & Dilution Factor with ISTDs


Signal 1: VWD1 A, Wavelength=254 nm

Peak RetTime Type  Width     Area      Height     Area
  #   [min]        [min]  mAU   *s   [mAU   ]       %
----|-------|----|-------|----------|----------|--------|
   1   5.783 VV    0.1660 9468.98926  871.83289 100.0000
```

Table 2, Entry 10

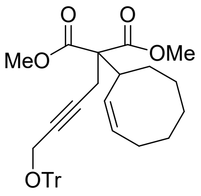

***ee pure*-13**

```
Data File C:\CHEM32\1\DATA\NIZAM\DEF_LC_CORRECT 2016-12-20 11-28-45\NH-299-B.D
Sample Name: NH-299-B

=====================================================================
Acq. Operator   : NIZAM                          Seq. Line :   2
Acq. Instrument : Instrument 1 Classic            Location : Vial 11
Injection Date  : 12/20/2016 11:48:45 AM               Inj :   1
                                                Inj Volume : 5.0 µl
Different Inj Volume from Sequence !     Actual Inj Volume : 15.0 µl
Acq. Method     : C:\CHEM32\1\DATA\NIZAM\DEF_LC_CORRECT 2016-12-20 11-28-45\IC, 90-10 HEPT-IPA,
                  0,8 ML-MIN,254NM,30M.M
Last changed    : 3/7/2011 2:58:51 PM
Analysis Method : C:\CHEM32\1\DATA\MARIUS\DEF_LC_CORRECT 2017-01-21 23-13-46\IA, 98-2 HEPT-IPA,
                  0,8 ML-MIN,254NM,30M.M
Last changed    : 5/23/2015 5:47:14 PM by gnanam
Method Info     : IA, 98/2 heptane/isopropanol, 0.8 ml/min, 254 nm, 30 min
```

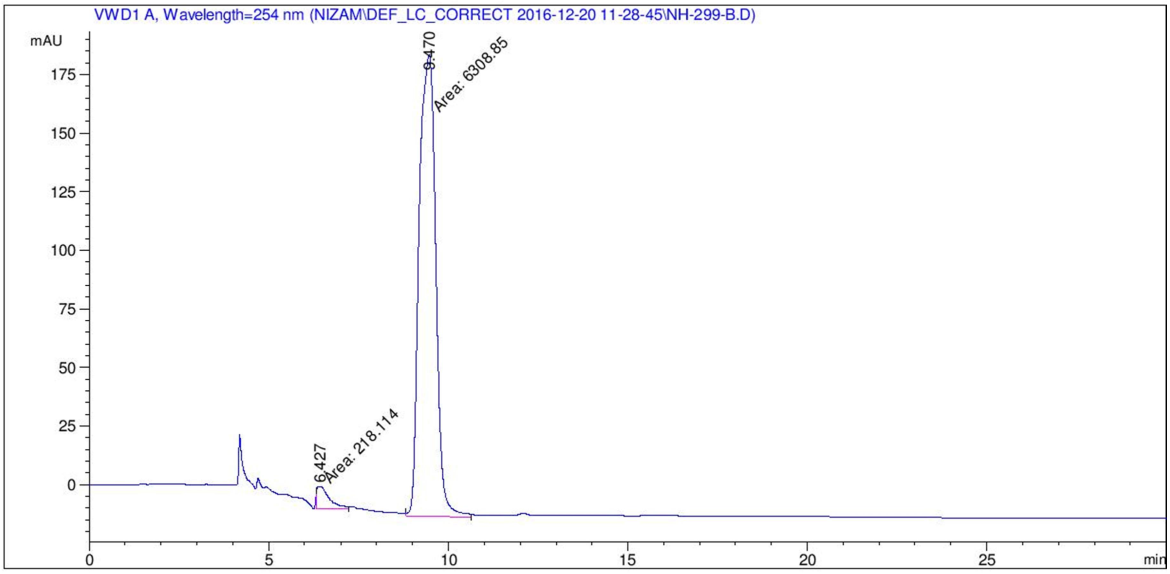


```
=====================================================================
                     Area Percent Report
=====================================================================

Sorted By             :      Signal
Multiplier:                  :      1.0000
Dilution:                    :      1.0000
Use Multiplier & Dilution Factor with ISTDs


Signal 1: VWD1 A, Wavelength=254 nm
```

Table 3, Entry 3

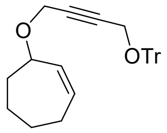

rac-**35**

```
Title        :
Run File     : c:\star\data\nizam\nh-312-1-hep-ipa-200-1- 254-nm-0.8ml ad.run
Method File  : c:\star\data\nizam\nh-312-1-hep-ipa-200-1- 254-nm-0.8ml ad-1.mth
Sample ID    : Default Sample

Injection Date: 12/30/2016 7:52 PM    Calculation Date: 1/24/2017 3:02 PM

Operator    : Operator                 Detector Type: 0800 (1 Volt)
Workstation: TROST-HPLC-LEFÿ HÚþ"      Bus Address  : 80
Instrument : Instrument #1             Sample Rate  : 50.00 Hz
Channel     : 1 = 1                    Run Time     : 35.000 min

** LC Workstation Multi Instrument (Demo) Version 6.41 ** 05000-31c8-fa9-30a1 **

Chart Speed =    0.57 cm/min   Attenuation = 133          Zero Offset = 3%
Start Time  =   0.000   min     End Time    = 35.000  min   Min / Tick  = 1.00
```

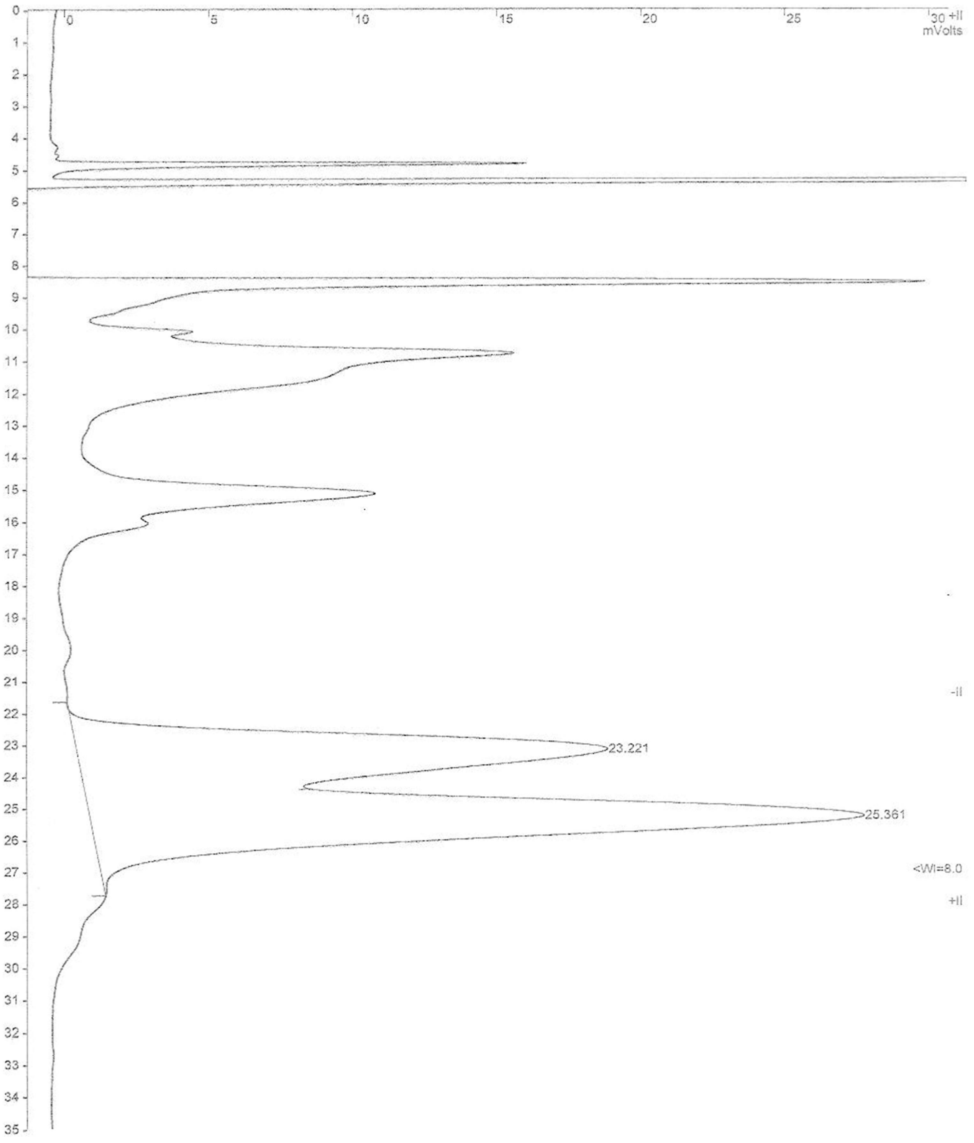

Table 3, Entry 4

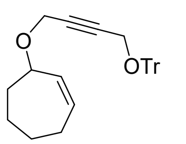

enantioenriched-**35**

```
Title        :
Run File     : c:\star\data\nizam\nh-318-1-hep-ipa-200-1- 254-nm-0.8ml ad.run
Method File  : c:\star\data\nizam\nh-318-1-hep-ipa-200-1- 254-nm-0.8ml ad-1.mth
Sample ID    : Default Sample

Injection Date: 12/31/2016 2:03 PM    Calculation Date: 1/12/2017 10:28 PM

Operator    : Operator                Detector Type: 0800 (1 Volt)
Workstation: TROST-HPLC-LEFÿ HÚþ"     Bus Address  : 80
Instrument  : Instrument #1           Sample Rate  : 50.00 Hz
Channel     : 1 = 1                   Run Time     : 60.000 min

** LC Workstation Multi Instrument (Demo) Version 6.41 ** 05000-31c8-fa9-30a1 **

Chart Speed =    0.33 cm/min   Attenuation = 343          Zero Offset = 2%
Start Time  =   0.000   min     End Time    = 60.000  min   Min / Tick  = 1.00
```

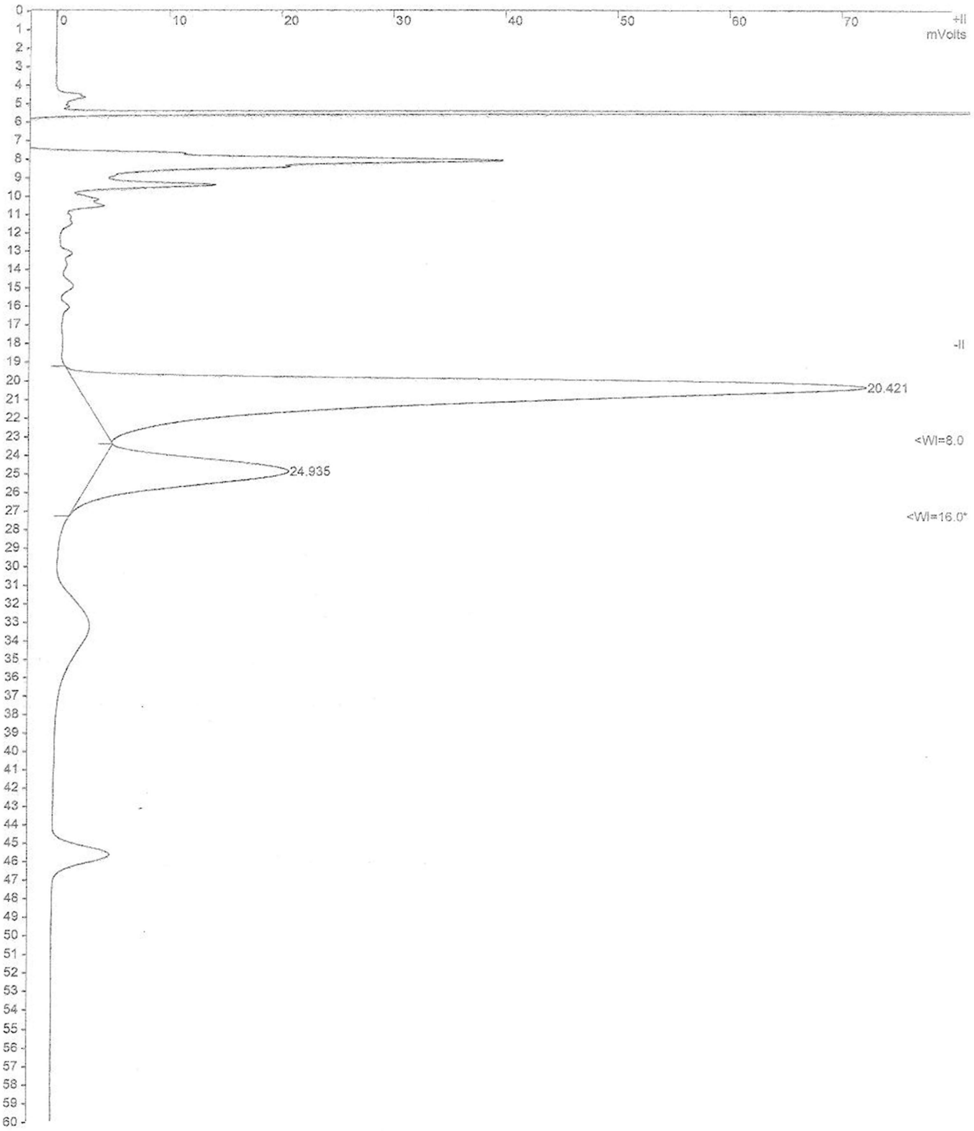

Table 2, Entry 13

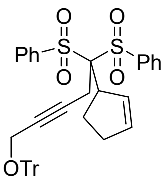

rac-**33**

```
Title        :
Run File     : c:\star\data\nizam\nh-264-6-hep-ipa-90 10 254-nm-0.8ml ad.run
Method File  : c:\star\data\nizam\nh-264-6-hep-ipa-90 10 254-nm-0.8ml ad-1.mth
Sample ID    : Default Sample

Injection Date: 12/16/2016 6:46 PM    Calculation Date: 1/23/2017 5:54 PM

Operator    : Operator                   Detector Type: 0800 (1 Volt)
Workstation: TROST-HPLC-LEFý HÚþ"        Bus Address  : 80
Instrument : Instrument #1               Sample Rate  : 50.00 Hz
Channel    : 1 = 1                       Run Time     : 56.000 min

** LC Workstation Multi Instrument (Demo) Version 6.41 ** 05000-31c8-fa9-30a1 **

Chart Speed =    0.35 cm/min   Attenuation = 282          Zero Offset = 15%
Start Time  =   0.000  min      End Time    = 56.000  min   Min / Tick  = 1.00
```

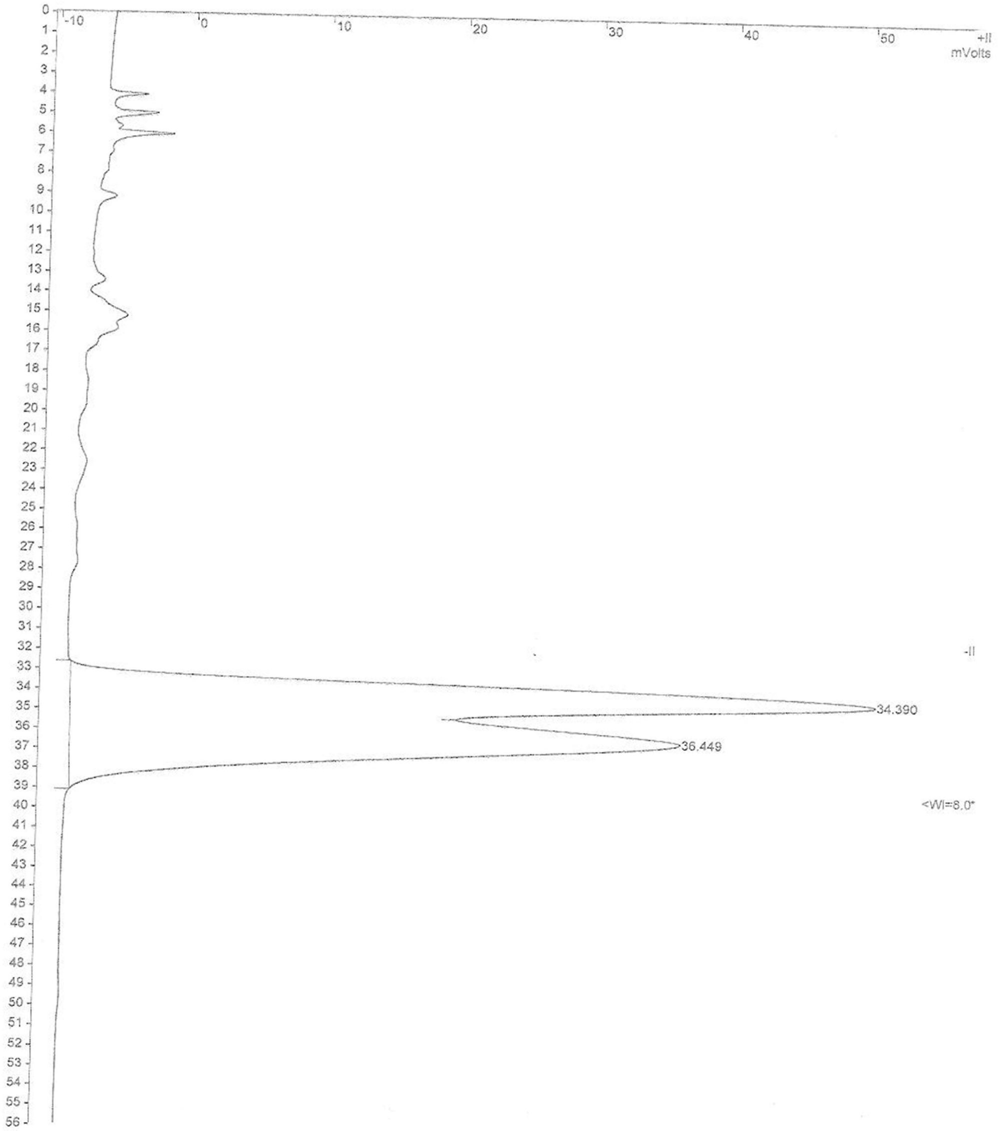

Table 2, Entry 14

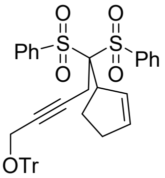

enantioenriched-**33**

Title :
Run File : c:\star\data\nizam\nh-281-hep-ipa-95 5 254-nm-0.8ml ad.run
Method File : c:\star\data\nizam\nh-281-hep-ipa-95 5 254-nm-0.8ml ad-1.mth
Sample ID : Default Sample

Injection Date: 12/19/2016 7:10 PM Calculation Date: 1/23/2017 5:51 PM

Operator : Operator
Workstation: TROST-HPLC-LEFÿ HÚþ"
Instrument : Instrument #1
Channel : 1 = 1

Detector Type: 0800 (1 Volt)
Bus Address : 80
Sample Rate : 50.00 Hz
Run Time : 100.000 min

** LC Workstation Multi Instrument (Demo) Version 6.41 ** 05000-31c8-fa9-30a1 **

Chart Speed = 0.20 cm/min Attenuation = 1217 Zero Offset = 2%
Start Time = 0.000 min End Time = 100.000 min Min / Tick = 1.00

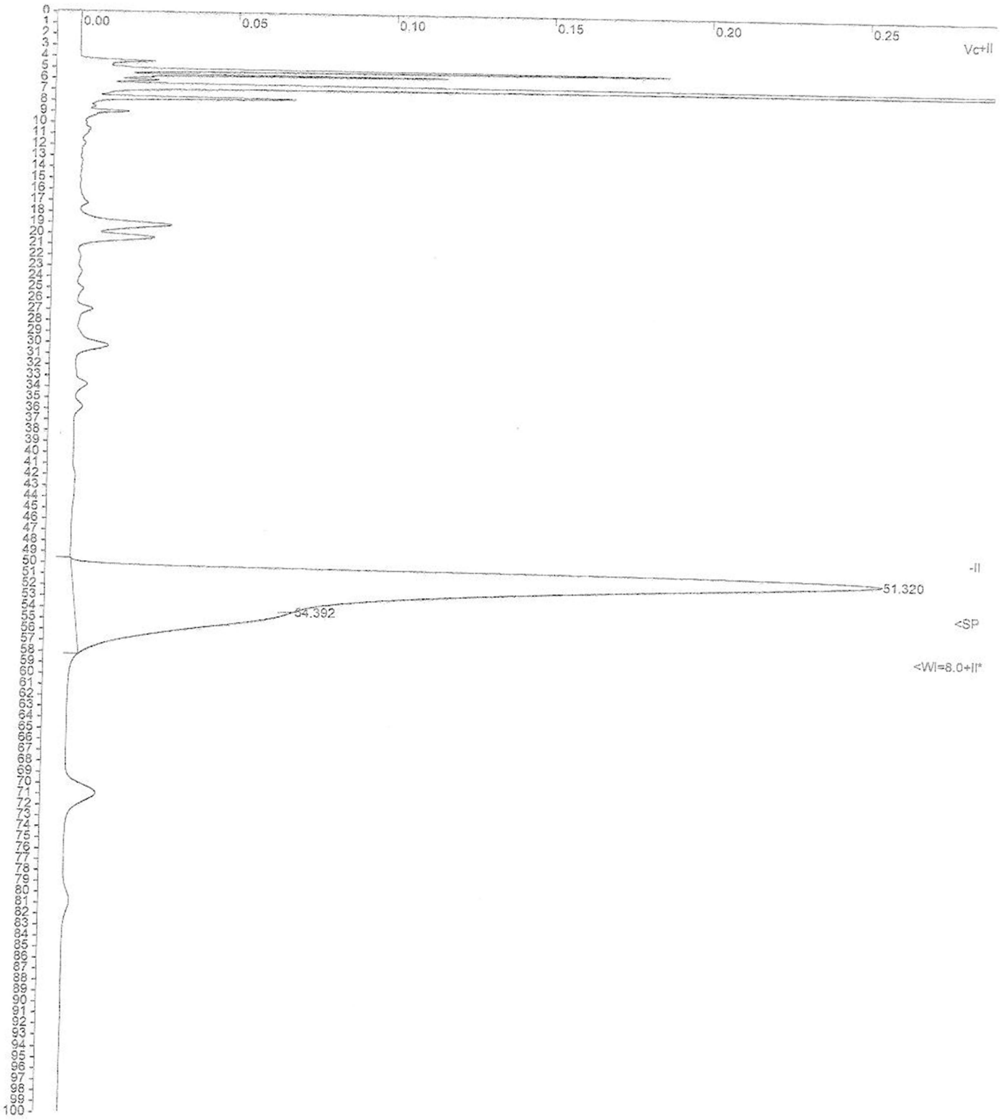

Supplement: Supplementary file 1 — Supplementary Materials [file turkjchem-44-1445-sup001.pdf]
